# Synthesis, Molecular Docking, and Antimycotic Evaluation of Some 3-Acyl Imidazo[1,2-*a*]pyrimidines

**DOI:** 10.3390/molecules23030599

**Published:** 2018-03-07

**Authors:** Omar Gómez-García, Dulce Andrade-Pavón, Elena Campos-Aldrete, Ricardo Ballinas-Indilí, Alfonso Méndez-Tenorio, Lourdes Villa-Tanaca, Cecilio Álvarez-Toledano

**Affiliations:** 1 Departamento de Química Orgánica-Laboratorio de Síntesis de Fármacos Heterocíclicos, Escuela Nacional de Ciencias Biológicas-IPN, Prolongación de Carpio y Plan de Ayala S/N, Colonia Santo Tomás, C.P. 11340 Ciudad de México, Mexico; camesol22@gmail.com; 2 Departamento de Microbiología-Laboratorio de Biología Molecular de Bacterias y Levaduras, Escuela Nacional de Ciencias Biológicas-IPN, Prolongación de Carpio y Plan de Ayala S/N, Colonia Santo Tomás, C.P. 11340 Ciudad de México, Mexico; lourdesvilla@hotmail.com; 3 Instituto de Química-UNAM, Circuito Exterior, Ciudad Universitaria, Coyoacán, C.P. 04510 Ciudad de México, Mexico; ricardo_snf.49.ers@hotmail.com; 4 Departamento de Bioquímica-Laboratorio de Biotecnología y Bioinformática Genómica, Escuela Nacional de Ciencias Biológicas-IPN, Prolongación de Carpio y Plan de Ayala S/N, Colonia Santo Tomás, C.P. 11340 Ciudad de México, Mexico; amendezt@ipn.mx

**Keywords:** 3-benzoyl imidazo[1,2-*a*]pyrimidines, molecular docking, antimycotic activity, Lanosterol 14α-demethylase, *Candida* spp.

## Abstract

A series of 3-benzoyl imidazo[1,2-*a*]pyrimidines, obtained from *N*-heteroarylformamidines in good yields, was tested in silico and in vitro for binding and inhibition of seven *Candida* species (*Candida albicans* (ATCC 10231), *Candida dubliniensis* (CD36), *Candida glabrata* (CBS138), *Candida guilliermondii* (ATCC 6260), *Candida kefyr*, *Candida krusei* (ATCC 6358) and *Candida tropicalis* (MYA-3404)). To predict binding mode and energy, each compound was docked in the active site of the lanosterol 14α-demethylase enzyme (CYP51), essential for fungal growth of *Candida* species. Antimycotic activity was evaluated as the 50% minimum inhibitory concentration (MIC50) for the test compounds and two reference drugs, ketoconazole and fluconazole. All test compounds had a better binding energy (range: −6.11 to −9.43 kcal/mol) than that found for the reference drugs (range: 48.93 to −6.16 kcal/mol). In general, the test compounds showed greater inhibitory activity of yeast growth than the reference drugs. Compounds **4j** and **4f** were the most active, indicating an important role in biological activity for the benzene ring with electron-withdrawing substituents. These compounds show the best MIC50 against *C. guilliermondii and* C. *glabrata,* respectively. The current findings suggest that the 3-benzoyl imidazo[1,2-*a*]pyrimidine derivatives, herein synthesized by an accessible methodology, are potential antifungal drugs.

## 1. Introduction

Imidazo[1,2-*a*]pyrimidine is an important core with a wide range of biological activity, including antitumor [1,2,3], antihyperglycemic [4], antitubercular [5], antiviral [6,7], anti-HIV [8], and antiatherosclerosis [9]. There are reports on the application of imidazo[1,2-*a*]pyridine derivatives as antifungal agents [10,11,12].

Fungal infections have become a growing problem for patient health, implying great economic losses in hospitals [13,14]. Among the main fungal infections is invasive candidiasis, caused by species belonging to the genus *Candida*, such as *C. albicans*, *C. glabrata*, *C. tropicalis*, and *C. parapsilosis* [15,16]. A common treatment for these infections is the application of a broad-spectrum antifungal agent. Prominent among the drugs administered for this purpose are different azoles (triazoles and imidazoles), which act by inhibiting the lanosterol 14α-demethylase (CYP51) enzyme, product of the (*CYP51/ERG11*) gene. Each of the CYP51 proteins has heme as its prosthetic group in the active site. This group is dependent on cytochrome P450, a key enzyme in the synthesis of sterols. The latter compounds are an important component of the fungal membrane [17].

Previous studies have demonstrated the efficacy of azoles and their derivatives on various strains and clinical isolates of *Candida* ssp. However, a large number of strains have shown resistance to azoles [18,19,20], making it necessary to seek alternatives for treatment of fungal infections. Although there are reports on the application of imidazo[1,2-*a*]pyrimidine derivatives as antifungal agents [21,22], they still do not display the pharmacological profile sought. Hence, it is necessary to design and synthesize new compounds with a better pharmacological profile.

The most widely used method of synthesis for the imidazo[1,2-*a*]pyrimidine nucleus was developed by Tschichibabin, based on the reaction between 2-aminopyrimidine and an alpha haloketone [23,24,25,26]. Despite the fact that imidazo[1,2-*a*]pyrimidines have been prepared with diverse variations in methodology, synthesis of 3-benzoyl-substituted imidazo[1,2-*a*]pyrimidines without a substituent in position 2 are scarce [27,28]. The known methods for the latter compounds employ conventional heating and microwave irradiation, such as the synthesis of 3-benzoyl imidazo[1,2-*a*]pyridine analogs previously described by our group [29]. However, no reports exist, to our knowledge, of the synthesis of 3-benzoyl-substituted imidazo[1,2-*a*]pyrimidines without using conventional heating or microwave irradiation.

The modern design and synthesis of new drugs has become more efficient by analyzing potential drugs with computational tools in order to explore the functional groups that can improve biological activity. Nevertheless, we were unable to find any reports on molecular docking studies carried out to establish the binding mode of 3-benzoyl imidazo[1,2-*a*]pyrimidine derivatives to the active site of the CYP51 enzyme. Such information would be advantageous for the evaluation of these compounds as antifungal agents against *Candida* species.

The aim of the present study was to synthesize a series of 3-benzoyl imidazo[1,2-*a*]pyrimidine derivatives without conventional heating or microwave irradiation, and analyze these compounds by molecular docking studies to determine their binding mode and energy in relation to the active site of the CYP51 enzyme. In addition, the antifungal activity of the test compounds was ascertained in relation to seven different *Candida* species, and these data were compared to the inhibitory activity of two reference drugs (fluconazole and ketoconazole).

## 2. Results

### 2.1. Synthesis

**Scheme 1:**
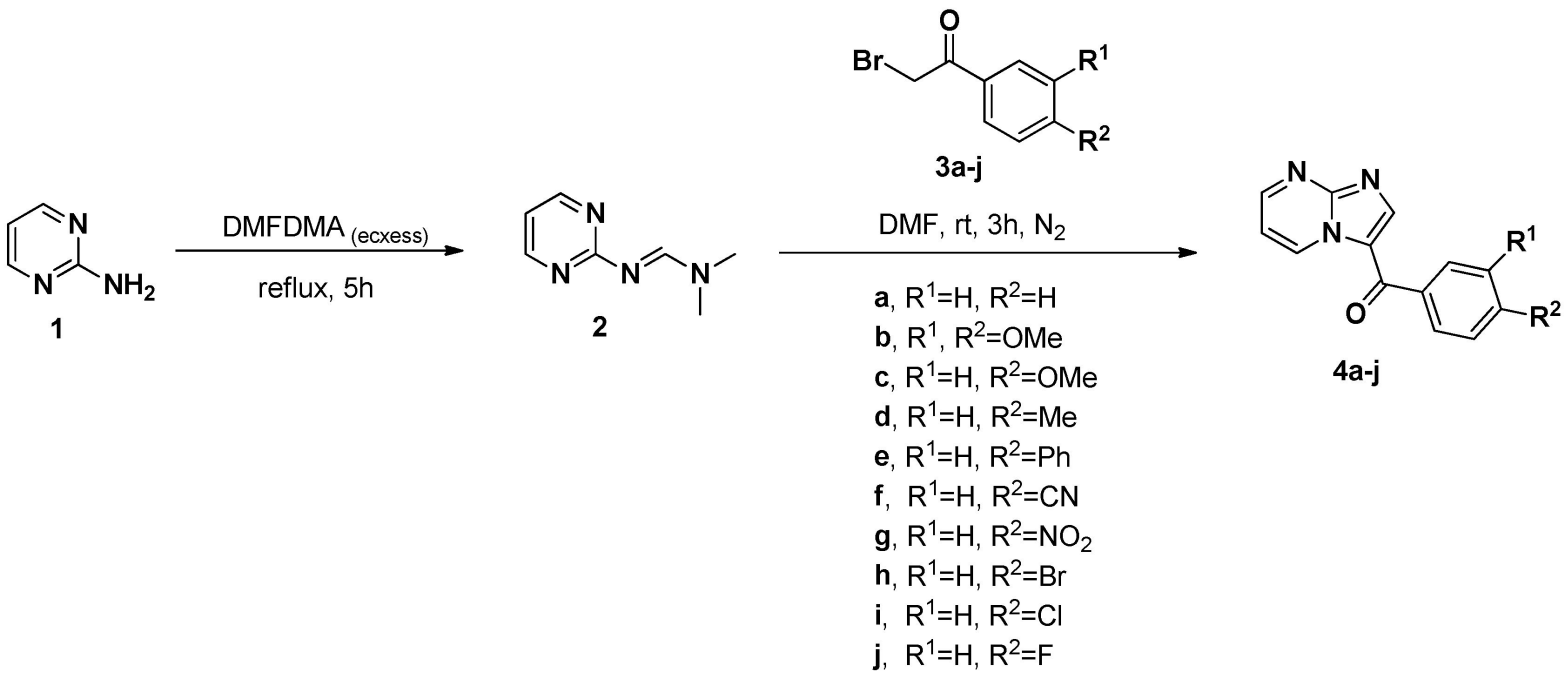
Synthesis of 3-benzoylimidazo[1,2-*a*]pyrimidines **4a**–**j** without conventional heating.

Imidazo[1,2-*a*]pyrimidines were presently synthesized from *N*,*N*-dimethyl-*N*-pyrimidilformamidine, which is obtained in quantitative yield by condensation of 2-amino pyrimidine **1** and an excess of *N*,*N*-dimethylformamide dimethyl acetal under reflux conditions. The synthetic route herein employed (Scheme 1) is a variation on our previous work [29]. The treatment of amidine **2** with different phenacyl bromides under inert atmosphere at room temperature for three hours gave the 3-benzoyl derivatives in synthetically useful yields (62–98%).

Thus, imidazo[1,2-*a*]pyrimidine derivatives with a benzoyl group having electron-donating groups (**4b**–**e**) or electron-withdrawing groups (**4f**–**j**) were prepared and fully characterized (Table 1).

The ^1^H NMR spectroscopic data are summarized in Table 2. Some compounds were analyzed in CDCl_3_ and others in deuterated trifluoroacetic acid.

### 2.2. Modeling CYP51 from Candida spp.

**Figure 1:**
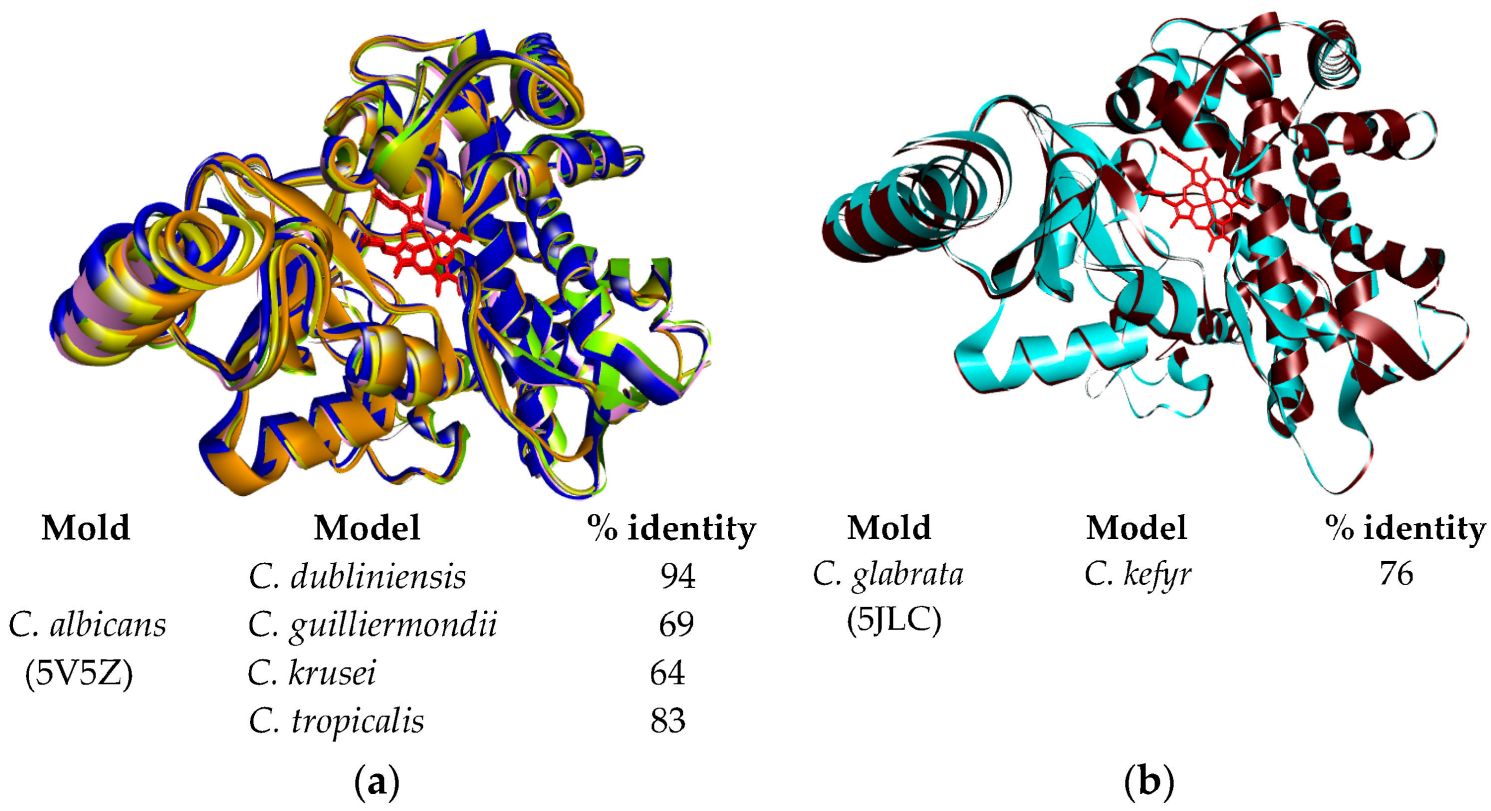
Flat ribbon representation of the CYP51 of *Candida* spp. (a) Overlapping of CYP51_Cd_ (purple), CYP51_Cgui_ (yellow), CYP51_Ck_ (orange), and CYP51_Ct_ (blue) with CYP51_Ca_ (PDB: 5V5Z) (green); (b) Overlapping of CYP51_Cke_ (brown) with CYP51_Cg_ (PDB: 5JLC) (cyan blue). The heme as the prosthetic group is shown in red (stick representation). For each of the models, moreover, the percentage of identity with their respective template is listed.

The 3D structures of the different CYP51 of *Candida* spp. (CYP51_Ca_) were initially generated with the homology modeling technique [30], widely used for the generation of comparative models. Fifteen models were presently generated by each query sequence. The DOPE method was utilized to evaluate the quality of the models obtained [31], selecting the best model based on the lowest score. The structures generated show overlapping with the template, indicating a high percentage of identity (>60%) with the latter (Figure 1). Ramachandran plots were constructed for the CYP51 sequences from *Candida* spp., revealing that 91.3% of the residues were located in favorable regions for the CYP51 sequence of *C. dubliniensis* (CYP51_Cd_), 91.5% for *C. guilliermondii* (CYP51_Cgui_), 92.4% for *C. kefyr* (CYP51_Cke_), 89.4% for *C. krusei* (CYP51_Ck_), and 91.4% for *C. tropicalis* (CYP51_Ct_) (Supplementary Materials Figure S57).

### 2.3. Molecular Docking of 3-Benzoyl Imidazo[1,2-*a*]pyrimidines in the Active Site of CYP51 of Candida spp.

**Figure 2:**
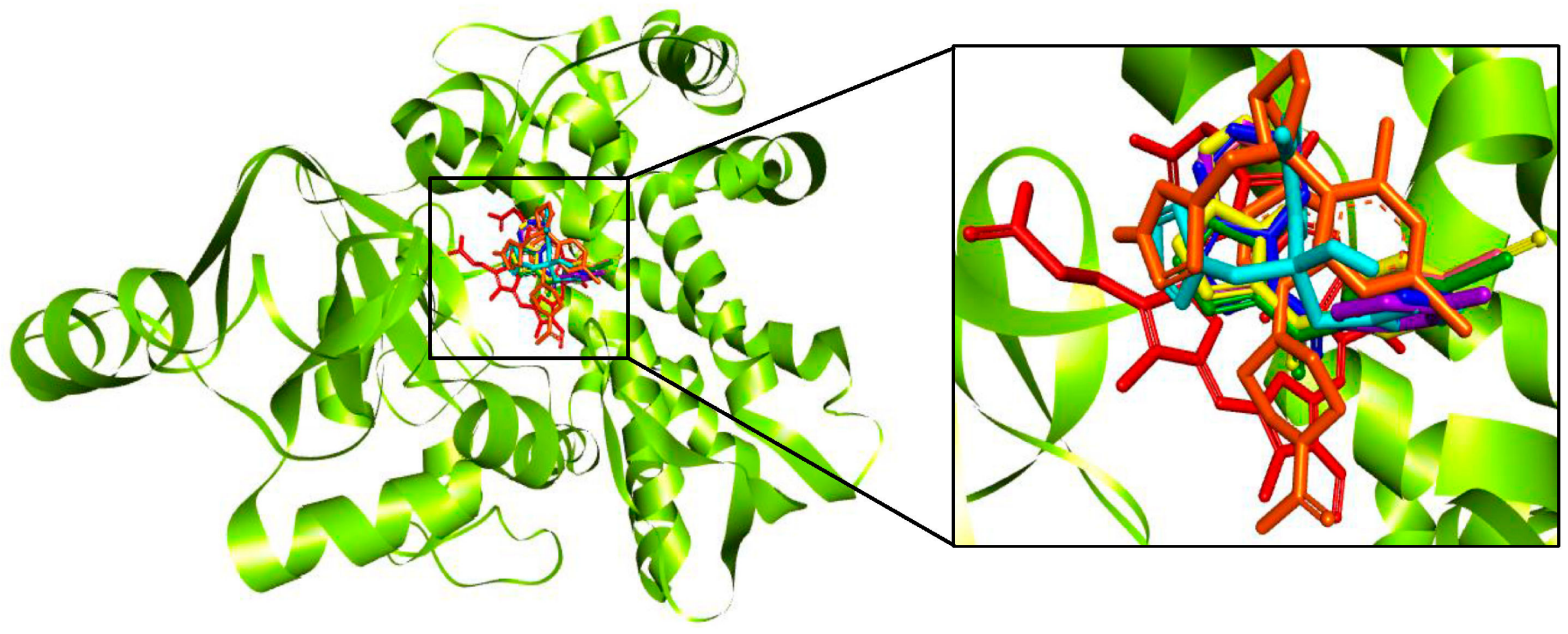
Binding mode on CYP51_Ca_ of the 3-benzoyl imidazo[1,2-*a*]pyrimidines and reference compounds. The five selected compounds and the reference compounds were overlaid on this receptor. CYP51_Ca_ is shown as a flat ribbon, while the compounds are illustrated with a stick model. Accordingly, the heme group is depicted in red, fluconazole in cyan blue, the ketoconazole in orange, **4a** in dark blue, **4d** in pink, **4f** in yellow, **4i** in green, and **4j** in purple.

A docking study was carried out between the 3-benzoyl imidazo[1,2-*a*]pyrimidine derivatives and the CYP51 from *Candida* spp. to explore the binding mode of these compounds and compare them to the two reference drugs, fluconazole and ketoconazole. It was observed that the synthesized and reference compounds bind in a similar manner inside the active site of CYP51 from *Candida* spp. A representative sample portrays the binding mode of the test compounds to CYP51_Ca_ (Figure 2).

Once the binding mode of imidazo[1,2-*a*]pyrimidine 3-benzoyl derivatives to the CYP51 of *Candida* spp. had been established, the affinity of each of these compounds to the distinct CYP51 models was analyzed. For this purpose, 100 different conformations were obtained for each compound, selecting the one with the lowest binding energy. Each of the 3-benzoyl imidazo[1,2-*a*]pyrimidine derivatives proved to have a better docking energy (−6.11 to −9.43 kcal/mol) in all CYP51 of *Candida* spp. than that found for fluconazole (−3.16 to −5.68 kcal/mol) or ketoconazole (48.93 to −6.16 kcal/mol) (Table 3). The current results are comparable to those found in other studies for fluconazole [32,33]. Of the five imidazo[1,2-*a*]pyrimidine derivatives with the best binding energies in CYP51_Ck_, **4f** had the lowest value (−9.43 kcal/mol). 

The interactions of ligands **4a**, **4d**, **4f**, **4i** and **4j** with each of the CYP51 from *Candida* spp. (Figures S61–S103 of Supplementary Materials) were analyzed to determine the residues involved. Within the active site of each of the CYP51 enzymes, functional groups or rings in imidazo[1,2-*a*]pyrimidine derivatives and reference compounds (fluconazole and ketoconazole) exhibited binding with similar polar and non-polar amino acid residues, such as Tyr108, Gly283, Leu352, Thr287, Thr98, Met493, Tyr94 and Phe102.

**Figure 3:**
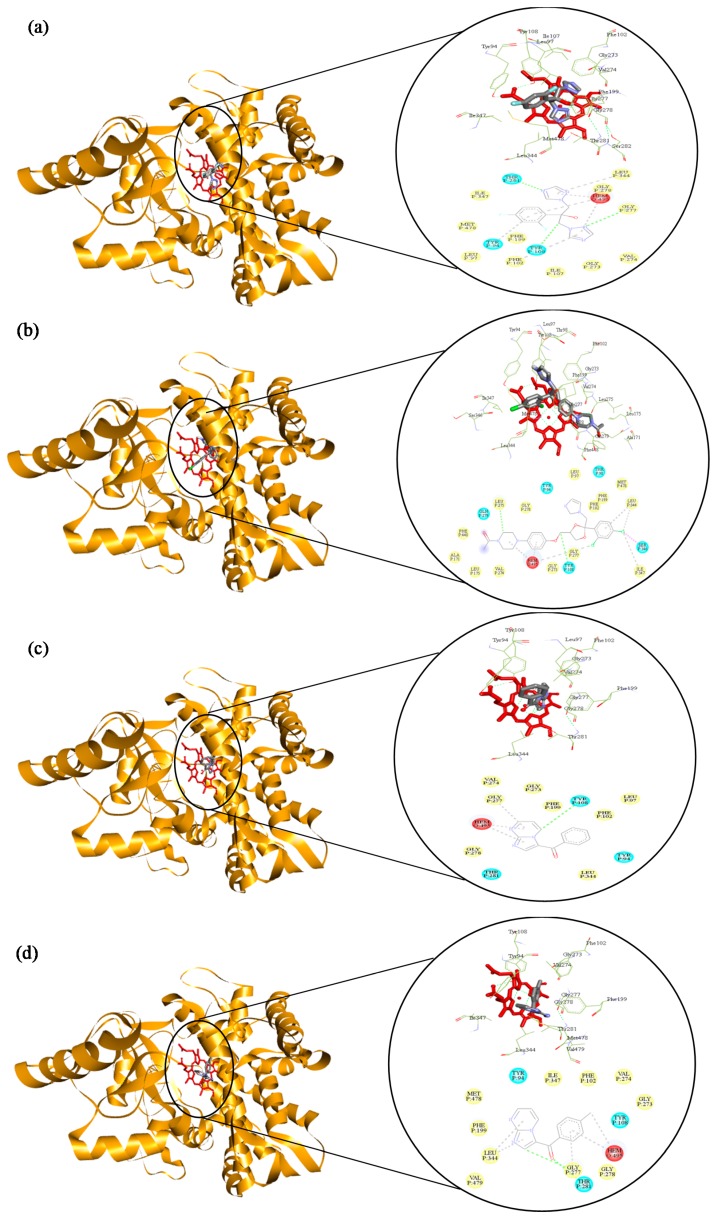

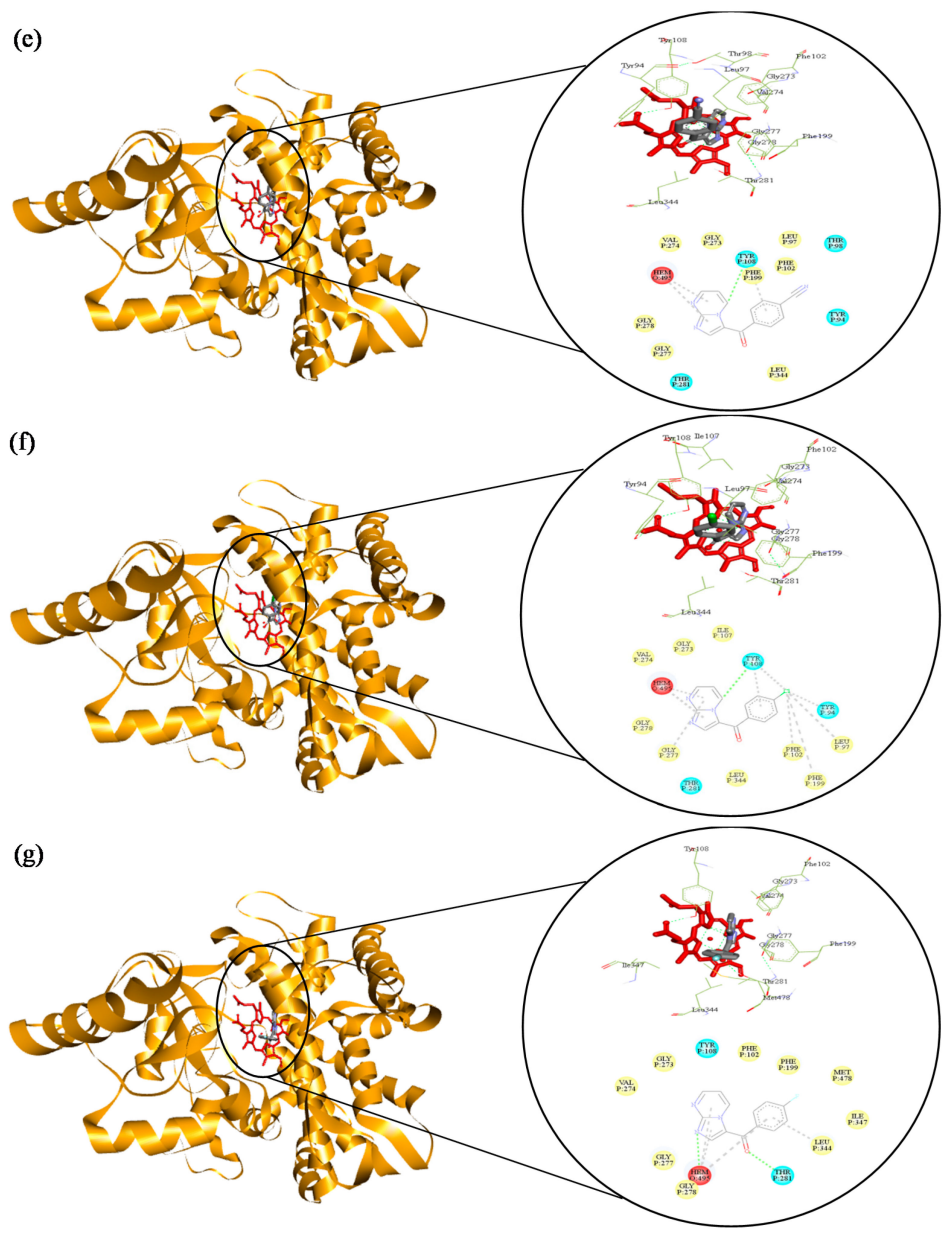
Schematic representation of the interactions of the five 3-benzoyl imidazo[1,2-*a*]pyrimidines and the two reference compounds with CYP51_Ck_. The flat ribbon denotes CYP51_Ck_, and the stick model portrays the heme group in red and the compounds in gray ((**a**), fluconazole; (**b**), ketoconazole; (**c**), **4a**; (**d**), **4d**; (**e**), **4f**; (**f**), **4i**; and (**g**), **4j**) within the active site of the enzyme. The coupling of the compounds with amino acid residues from the active site of CYP51_Ck_ are illustrated in a 3D model. The 2D model shows hydrophilic interactions (green dotted lines), hydrophobic interactions (gray dotted lines) and electrostatic interactions (without lines). Polar amino acids (cyan blue) and non-polar ones (green) are displayed in the circle.

Once the interactions between the compounds and each of the CYP51 of the *Candida* species had been examined, it was found that the CYP51 of *Candida krusei* displayed the best binding energies. Hence, CYP51_Kru_ was utilized as the receptor model to illustrate the interactions of the five docked compounds (Figure 3a–g).

**Figure 4:**
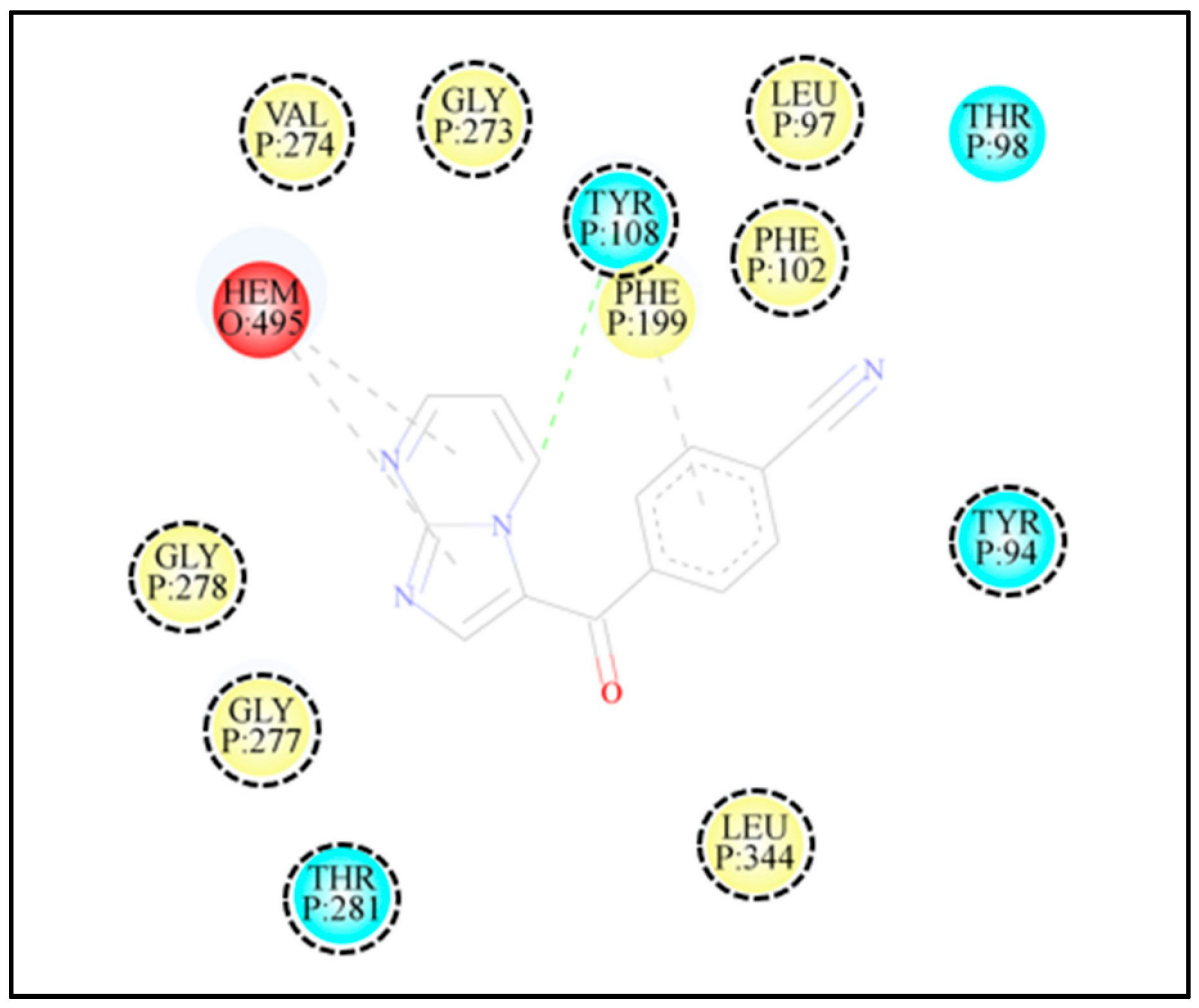
Schematic representation of the interactions between compound **4f** and CYP51_Ck_. The dashed lines indicate the amino acids in the five 3-benzoyl imidazo[1,2-*a*]pyrimidines and the two reference compounds.

An extension the 2D model is provided to depict the interactions between the imidazo[1,2-*a*]pyrimidine derivative **4f** and CYP51_Ck_ (Figure 4). Interestingly, polar and non-polar amino acid residues are shared between the reference compounds and imidazo[1,2-*a*]pyrimidine derivatives. The most frequently shared residues were non-polar, including Tyr94, Leu97, Thr98, Phe102, Tyr108, Phe199, Gly273, Val274, Gly277, Gly278, and Leu344. This suggests an important role of the amino acid residues of a hydrophobic character in the binding mechanism of the compounds to CYP51_Ck_ [34].

### 2.4. Susceptibility of Candida spp. to 3-Benzoylimidazo[1,2-*a*]pyrimidines

We evaluated the susceptibility of the *Candida* strains to the imidazo[1,2-*a*]pyrimidine derivatives by the microdilution method [35]. The MIC50 was calculated for each of the compounds according to materials and methods (Table 4), finding that the five test compounds have a value much lower than that determined for either fluconazole or ketoconazole. The imidazo[1,2-*a*]pyrimidine derivative **4f** showed the lowest MIC50 values for *C. dubliniensis*, *C. glabrata* and *C. krusei*, while **4j** had the lowest values for *C. albicans* and *C. guilliermondii*. For *C. kefyr*, only compound **4d** presented a MIC50 slightly higher than the remaining 3-benzoyl imidazo[1,2-*a*]pyrimidine derivatives.

## 3. Discussion

The mechanistic pathway for the synthesis of imidazo[1,2-*a*]pyrimidines is herein reported. It involves the formation of the pyridinium salt followed by the intramolecular cyclization of the carbanion (adjacent to the quaternary nitrogen) on the amidine carbon via a *5-exo-trig* process. Finally, aromatization took place with the loss of dimethylamine. IR and NMR (^1^H and ^13^C) spectroscopy as well as mass spectrometry analysis confirmed the structures of the synthesized compounds. With the ^1^H NMR spectrum of compounds **4a**–**j**, an abnormal chemical shift to downfield of proton 5 (~9.88–10.10 ppm) was found. This effect, also observed in 3-acyl imidazo[1,2-*a*]pyridines [36], is due to the interaction of C-H····O with the oxygen of the carbonyl group.

The identification of compound **4b** was unequivocally established. Its molecular weight was found to be *m*/*z* = 283 (as determined by HREIMS). In the ^13^C NMR spectrum, the conjugated carbonyl group is evidenced by the signal at 183.7 ppm. The HMBC experiment showed two correlations between C-8a and H-5, as well as another interaction of C-8a with H-2. Moreover, an important correlation existed between C-3 and H-2, an interaction that confirms the presence of a quaternary carbon. On the other hand, the carbonyl group exhibited two correlations, with H-12 and H-16. The proton resonances of **4e**–**i** were shifted due to the solvent used for the dilution (Table 2). In all cases, the compounds examined in CF_3_COOD were downfield compared to those analyzed in CDCl_3_, due to the greater polarity of CF_3_COOD versus CDCl_3_. The most significant changes (over 1 ppm) were detected for the equivalent protons (H-13 and H-15) in **4c** with respect to **4g**, where displacement strongly depended both on the solvent [37] and the electron-withdrawing and electron-donor nature of the *nitro* and *methoxy* substituents.

To evaluate whether the compounds synthesized herein are linked to the CYP51 enzyme of yeasts, models of CYP51 of *Candida* spp., were generated. The CYP51 of various species of the *Candida* genus proved to have a high structural similarity, which indicates that this structure is conserved among species [38], and emphasizes the importance of CYP51 as a therapeutic target of new drugs with antifungal activity [34].

Ramachandran diagrams were constructed to allow for visualization of the energetically favorable regions that are established by the dihedral angles of a protein [39]. For each CYP51 analyzed, 90% of the residues fall within the favorable regions. Hence, the models obtained are of good quality and comparable to those employed elsewhere [40]. The docking studies revealed that the 3-benzoyl imidazo[1,2-*a*]pyrimidine derivatives act on the same target as the azoles [40]. Consequently, these derivatives likely exhibit a broad spectrum of antifungal action on various species of the *Candida* genus, similar to that found previously for other azoles [32].

According to the docking results (Table 3), **4f** and **4d** have the best binding energies in relation to CYP51_Ca_ and CYP51_Cd_, **4i** with respect to CYP51_Cg_ (and to a lesser extent in CYP51_Cgui_ and CYP51_Ck_), as well as **4a** and **4j** in relation to CYP51_Cke_. Thus, 3-benzoyl imidazo[1,2-*a*]pyrimidines bound with distinct affinity in each of the CYP51 presently analyzed. Moreover, the docking studies on the different CYP51 enzymes demonstrated that similar amino acid residues interact with groups in the structures of the five 3-benzoyl imidazo[1,2-*a*]pyrimidine derivatives and the reference compounds. These findings indicate that the test compounds likely have a mechanism similar to that reported for azoles [33,41]. Additionally, hydrophobic, hydrophilic, and electrostatic interactions were evidenced between different substituents in the test ligands, such as methyl and halogen (Cl or F). Therefore, these connections are favored in the *para* position of the benzene ring.

According to the results, the best binding energies with CYP51_Ck_ were shown by the five imidazo[1,2-*a*] pyrimidine derivatives selected in this study. Compound **4f** displayed the greatest affinity for the active site of the enzyme, which is due to the C–H ·····O interactions of the imidazo[1,2-*a*]pyrimidine ring with the polar side chain of Tyr108 and Gly273 C–H. The current affinity data imply that imidazo[1,2-*a*]pyrimidine derivatives inhibit CYP51_Ck_ more effectively than other CYP51. We propose that the cyano substituent in the structure of **4f** may play an important role in the binding mode of this compound to the CYP51_Ck_.

During the docking study, the heme group of the protein exhibited hydrophobic interactions with the heterocyclic rings (imidazole and pyrimidine) of the 3-benzoyl imidazo[1,2-*a*]pyrimidine compounds, as well as with the triazole and imidazole rings of fluconazole and ketoconazole, respectively. For imidazo[1,2-*a*]pyrimidine derivatives **4a** and **4i**, hydrogen bonds were detected between amino acid Tyr108 of CYP51_Ck_ and H-5 of the pyrimidine ring. Moreover, halogen bonds (Cl) were observed between compound **4i** and the Tyr94, Leu97, Phe199, and Phe102 residues. Also found was another hydrogen bond between the oxygen of the carbonyl group in compound **4j** and the amino acid residue Tyr281. Hydrophobic interactions could be appreciated between Gly277 and the heterocyclic moiety in compound **4a** and **4i**, and between this residue and the benzene ring of compound **4d**. For fluconazole, hydrogen bonds were identified between Gly277 and the triazole ring, and hydrophobic interactions between Tyr94 and the other triazole moiety. For ketoconazole, hydrogen bonds were formed with the side chain of Tyr108, and type π-alkyl hydrophobic interactions occurred between Leu344 and the benzene ring, as well as between Leu344 and the Cl substituent in the benzene ring. Another important interaction was between the chlorine in the benzene ring and Ser346 [17,34,42].

Regarding the susceptibility of the *Candida* species to inhibition, the test compounds had lower MIC50 values than fluconazole and ketoconazole. However, if these results are compared with those reported in the literature for another class of azoles, such as voriconazole, we observe that the MIC50 for this antifungal in *C. krusei* is 0.25 [35], similar to that found in compound **4f**, for *C. glabrata* and *C. albicans* MIC50 of 0.125 [43] and 1 [44] were found, respectively, observing that in *C. glabrata*, four compounds showed an MIC50 similar or better than voriconazole, while in *C. albicans*, the five derivatives showed better results. Regarding *C. kefyr*, four of the compounds showed MIC50 equivalent to that reported in voriconazole (0.125) [45,46], in *C. guilliermondii* [45,46], three of the compounds exhibited similar MIC50 (0.0625), while derivative **4j** obtained a better value than the reference compound, and finally, for *C. tropicalis* [45,46], compound **4i** showed a similar value (0.0312). The comparison of the MIC50 of the imidazo[1,2-*a*]pyrimidine derivatives indicate that different classes of azoles exhibit different inhibitory effects on the seven species of *Candida*, although it is observed that some are better in some species than in others, and it is necessary to search for new drugs or to improve those already proposed, that can be used in combinatorial therapy with already known compounds with the aim of improving the MIC50 reported. Given the above, these observations suggest that 3-benzoyl imidazo[1,2-*a*]pyrimidines may offer an alternative to azoles [33,41,47,48] in the treatment of infections caused by *Candida* spp.

## 4. Materials and Methods

### 4.1. Chemicals and Instruments

All glassware was thoroughly oven-dried. Chemicals and solvents were purchased from commercial suppliers. Melting points were determined on a Melt Temp II apparatus and are reported without correction. By using chloroform-*d* and CF_3_COOD, the ^1^H and ^13^C NMR spectra were recorded on a Varian NMR system (Palo Alto, CA, USA) at 500 MHz (^1^H NMR) and 125 MHz (^13^C NMR), as well as on a Bruker Advance III (Bruker Biospin, Ettlingen, Germany) at 300 MHz (^1^H NMR) and 75 MHz (^13^C NMR). Chemical shifts are given in parts per million with reference to internal TMS (Sigma-Aldrich, San Luis, MO, USA). EI-MS spectra were recorded on a JEOL JMS-AX505 (Akishima, Tokyo, Japan) and a JEOL GCmate spectrometer (Akishima). IR spectra were obtained with a Perkin Elmer FT-IR SPECTRUM 2000 spectrophotometer (Waltham, MA, USA).

### 4.2. Synthesis of Imidazo[1,2-a]pyrimidin-3-yl(phenyl)methanones (***3a**–**j***)

To a solution of *N*,*N*-dimethyl-*N*′-(pyrimidin-2-il)formamidine (**2**, 1 mmol) in anhydrous *N*,*N*-dimethylformamide (7 mL), at room temperature and under N_2_ atmosphere, was added the appropriate phenacyl bromide (**3a**–**j**, 1 mmol) in anhydrous *N,N*-dimethylformamide (7 mL). The reaction was stirred for three hours at room temperature. Then the mixture was added to a flask containing H_2_O (30 mL) and extracted with EtOAc (3 × 20 mL). The organic extracts were combined and dried (anhydrous Na_2_SO_4_), and the solvent was removed under reduced pressure to give the title compounds **4a**–**d** and **4j**, which were further purified by column chromatography on silica gel with a mixture of hexane and EtOAc as eluent. In the case of the phenacyl bromides **3e**–**i**, once the reaction was completed the mixture was added to a flask containing ice (30 g), then the product was precipitated, filtered under vacuum, and recrystallized from ethanol–water to deliver the title compounds **4e**–**i**.

*Imidazo[1,2-*a*]pyrimidin-3-yl(phenyl)methanone* (**4a**). Synthesis of **4a** furnished a yellow solid (0.17 g, 77.5% yield); m.p. 234–235.5 °C (lit. m.p. 234 °C) [25]; IR (KBr) υ_max_ 3134 (Csp^2^-H), 3053 (Csp^3^-H), 1611 (C=O), 1513 (C=C), 1475 (C=C) cm^−1^; ^1^H NMR (CDCl_3_, 300 MHz) δ 10.0 (1H, dd, *J* = 7 Hz, *J* = 2.0 Hz, H-5), 8.84 (1H, d, *J* = 4.5 Hz, *J* = 2.0 Hz, H-7), 8.41 (1H, s, H-2), 7.9 (2H, dd, *J* = 7.5 Hz, *J* = 1.5 Hz, H-12, H-16), 7.65 (1H, tt, *J* = 7.5 Hz, *J* = 1.5 Hz, H-14), 7.57 (2H, t, *J* = 7.5 Hz, H-13, H-15), 7.23 (1H, dd, *J* = 7 Hz, *J* = 4.5 Hz, H-6); ^13^C NMR (CDCl_3_, 75 MHz) δ 185.1, 153.7, 151.4, 146.3, 138.2, 136.6, 132.6, 128.8, 128.7, 121.7, 111.3; DART-MS *m*/*z* 224 [M + H]^+^ (100); HREIMS *m*/*z* 223.0746 (calcd. for C_13_H_9_N_3_O, 223.0746).

*(3,4-dimethoxyphenyl)(imidazo[1,2-*a*]pyrimidin-3-yl)methanone* (**4b**). Synthesis of **4b** afforded a white solid (0.23 g, 81.1% yield); m.p. 251.5–252.5 °C; IR (CH_2_Cl_2_) υ_max_ 2959.9 (Csp^3^-H), 1615.5 (C=O), 1578.4 (C=C), 1510.3 (C=C), 1470.9 (C=C), 1280.9 (C–O), 1264.7 (C–O) cm^−1^; ^1^H NMR (CDCl_3_, 300 MHz) δ 9.88 (1H, dd, *J* = 6.9 Hz, *J* = 2.1 Hz, H-5), 8.78 (1H, dd, *J* = 4.2 Hz, *J* = 2.1 Hz, H-7), 8.39 (1H, s, H-2), 7.54 (2H, dd, *J* = 8.4 Hz, *J* = 2.1 Hz, H-16), 7.43 (1H, d, *J* = 1.8 Hz, H-12), 7.18 (1H, dd, *J* = 6.9 Hz, *J* = 4.2 Hz, H-6), 6.96 (1H, d, *J* = 8.4 Hz, H-15), 3.96 (3H, s, OMe), 3.94 (3H, s, OMe); ^13^C NMR (CDCl_3_, 75 MHz) δ 183.7, 153.3, 153.0, 151.2, 149.2, 145.4, 136.5, 130.8, 123.4, 121.7, 111.1, 111.0, 110.3, 56.1, 55.9; DART-MS *m*/*z* 284 [M + H]^+^ (100); HREIMS *m*/*z* 284.1031 (calcd. for C_15_H_13_N_3_O_3_, 284.1035).

*Imidazo[1,2-*a*]pyrimidin-3-yl(4-methoxyphenyl)methanone* (**4c**). Synthesis of **4c** gave a light yellow solid (0.17 g, 67.1% yield); m.p. 224–227 °C; IR (CH_2_Cl_2_) υ_max_ 2915.8 (Csp^3^-H), 1608.3 (C=O), 1575.1 (C=C), 1515.9 (C=C), 1478.6 (C=C), 1256.7 (C–O) cm^−1^; ^1^H NMR (CDCl_3_, 300 MHz) δ 9.90 (1H, dd, *J* = 6.9 Hz, *J* = 1.8 Hz, H-5), 8.78 (1H, dd, *J* = 4.2 Hz, *J* = 1.8 Hz, H-7), 8.37 (1H, s, H-2), 7.88 (2H, d, *J* = 8.7 Hz, H-12, H-16), 7.17 (1H, dd, *J* = 6.9 Hz, *J* = 4.2 Hz, H-6), 7.00 (2H, d, *J* = 8.7 Hz, H-13, H-15), 3.9 (3H, s, OMe); ^13^C NMR (CDCl_3_, 75 MHz) δ 183.8, 163.3, 153.4, 151.1, 145.3, 136.6, 131.1, 130.7. 121.7, 114.0, 111.0, 55.5; DART-MS *m*/*z* 254 [M + H]^+^ (100); HREIMS *m*/*z* 254.0921 (calcd. for C_14_H_11_N_3_O_2_, 254.0929).

*Imidazo[1,2-*a*]pyrimidin-3-yl(p-tolyl)methanone* (**4d**). Synthesis of **4d** delivered a white solid (0.21 g, 90.3% yield); m.p. 208–209 °C; IR (KBr) υ_max_ 3134.7 (Csp^2^-H), 2923.4 (Csp^3^-H), 1611.8 (C=O), 1513.8 (C=C), 1507(C=C),, 1477 (C=C) cm^−1^; ^1^H NMR (CDCl_3_, 300 MHz) δ 9.94 (1H, dd, *J* = 6.9 Hz, *J* = 1.8 Hz, H-5), 8.79 (1H, dd, *J* = 4.2 Hz, *J* = 1.8 Hz, H-7), 8.37 (1H, s, H-2), 7.77 (2H, d, *J* = 7.8 Hz, H-12, H-16), 7.32 (2H, d, *J* = 7.8 Hz, H-13, H-15), 7.19 (1H, d, *J* = 6.9 Hz, *J* = 4.2 Hz, H-6), 2.48 (3H, s, Me); ^13^C NMR (CDCl_3_, 75 MHz) δ 184.8, 153.4, 151.3, 145.9, 143.3, 136.6, 135.5, 129.4, 128.9, 121.7, 111.1, 21.6; EI-MS *m*/*z* 237 [M]^+^ (100); HREIMS *m*/*z* 237.0900 (calcd. for C_14_H_11_FN_3_O, 237.0902).

*[1,1'-biphenyl]-4-yl(imidazo[1,2-*a*]pyrimidin-3-yl)methanone* (**4e**). Synthesis of **4e** provided a light yellow solid (0.21 g, 70.2% yield); m.p. 244.5–247 °C; IR (KBr) υ_max_ 3133.3 (Csp^2^-H), 1607.4 (C=O), 1510.1 (C=C), 1478.2 (C=C) cm^−1^; ^1^H NMR (CF_3_COOD, 300 MHz) δ 10.08 (1H, d, *J* = 6.6 Hz, H-5), 9.08 (1H, d, *J* = 2.7 Hz, H-7), 8.53 (1H, s, H-2), 7.87 (2H, d, *J* = 7.8 Hz, H-12, H-16), 7.74 (3H, d, *J* = 7.8 Hz, H-6, H-13, H-15), 7.52 (2H, d, *J* = 7.8 Hz, H-18, H-22), 7.29-7.37 (3H, m, H-19, H-20, H-21); ^13^C NMR (CF_3_COOD, 75 MHz) δ 183.9, 161.6, 151.5, 144.9, 141.1, 140.0, 132.5, 130.7, 130.4, 129.6, 128.7, 127.7, 125.1, 122.1, 117.4; DART-MS *m*/*z* 300 [M + H]^+^ (100); HREIMS *m*/*z* 300.1130 (calcd. for C_19_H_13_N_3_O, 300.1136).

*4-(imidazo[1,2-*a*]pyrimidine-3-carbonyl)benzonitrile* (**4f**). Synthesis of **4f** generated a white solid (0.21 g, 84.6% yield); m.p. 277–281 °C; IR (KBr) υ_max_ 3097.9, 3074 (Csp^2^-H), 2230.3 (C≡N), 1629.3 (C=O), 1522.3 (C=C), 1476.0 (C=C) cm^−1^; ^1^H NMR (CF_3_COOD, 300 MHz) δ 10.10 (1H, d, *J* = 6.9 Hz, H-5), 9.13 (1H, d, *J* = 4.5 Hz, H-7), 8.51 (s, 1H, H-2), 7.77 (1H, dd, *J* = 6.9 Hz, *J* = 4.5 Hz, H-6), 7.62 (4H, d, *J* = 7.1 Hz, H-12, H-13, H-15, H-16); ^13^C NMR (CF_3_COOD, 75 MHz) δ 185.9, 161.4, 145.0, 140.2, 134.3, 133.8, 133.7, 132.3, 132.1, 130.9, 122.4, 117.2; DART-MS *m*/*z* 249 [M + H]^+^ (100); HREIMS *m*/*z* 249.0775 (calcd. for C_14_H_8_N_4_O, 249.0776).

*Imidazo[1,2-*a*]pyrimidin-3-yl(4-nitrophenyl)methanone* (**4g**). Synthesis of **4g** produced a light yellow solid (0.20 g, 74.5% yield); m.p. 233.2–233.7 °C; IR (KBr) υ_max_ 3113.7 (Csp^2^-H), 1630 (C=O), 1598.2 (C=C), 1521 (C=C), 1480.7 (C=C), 1349.7 (NO_2_) cm^−1^; ^1^H NMR (CF_3_COOD, 300 MHz) δ 10.08 (1H, d, *J* = 6.9 Hz, H-5), 9.10 (1H, d, *J* = 4.5 Hz, H-7), 8.52 (1H, s, H-2), 8.29 (2H, d, *J* = 8.4 Hz, H-13, H-15), 7.96 (2H, d, *J* = 8.4 Hz, H-12, H-16), 7.76 (1H, dd, *J* = 6.9 Hz, *J* = 4.5 Hz, H-6); ^13^C NMR (CF_3_COOD, 75 MHz) δ 183.9, 161.5, 151.4, 144.9, 141.0, 140.0, 132.5, 130.6, 125.1, 121.9, 117.3; DART-MS *m*/*z* 269 [M + H]^+^ (100); HREIMS *m*/*z* 269.0669 (calcd. for C_13_H_8_N_4_O_3_, 269.0674).

*(4-bromophenyl)(imidazo[1,2-*a*]pyrimidin-3-yl)methanone* (**4h**). Synthesis of **4h** resulted in a yellow solid (0.29 g, 98% yield); m.p. 248–249 °C; IR (KBr) υ_max_ 3113.6 (Csp^2^-H), 3043.6 (Csp^3^-H), 1630 (C=O), 1520 (C=C), 1480.7 (C=C) cm^−1^. ^1^H NMR (CF_3_COOD, 300 MHz) δ 10.00 (1H, d, *J* = 6.9 Hz, H-5), 9.04 (1H, d, *J* = 3.6 Hz, H-7), 8.43 (1H, s, H-2), 7.67 (3H, d, *J* = 7.5 Hz, H-6, H-12, H-16), 7.4 (2H, d, *J* = 7.5 Hz, H-13, H-15); ^13^C NMR (CDCl_3_, 75 MHz) δ 180.8, 161.1, 144.7, 143, 140, 133.9, 131.9, 130.8, 130.4, 122.2, 117.0; EI-MS *m*/*z* 302 [M + 2]^+^ (97), 300 [M]^+^ (100); HREIMS *m*/*z* 300.9851 (calcd. for C_13_H_8_BrN_3_O, 300.9851).

*(4-chlorophenyl)(imidazo[1,2-*a*]pyrimidin-3-yl)methanone* (**4i**). Synthesis of **4i** furnished a light yellow solid (0.18 g, 72.1% yield); m.p. 240–241 °C; IR (KBr) υ_max_ 3135 (Csp^2^-H), 1613 (C=O), 1513 (C=C), 1513, 1476 (C=C) cm^−1^. ^1^H NMR (CF_3_COOD, 300 MHz) δ 10.00 (1H, d, *J* = 6.6 Hz, H-5), 9.03 (1H, s, H-7), 8.42 (1H, s, H-2), 7.68 (3H, d, *J* = 7.8 Hz, H-6, H-12, H-16), 7.41 (2H, d, *J* = 7.8 Hz, H-13, H-15). ^13^C NMR (CDCl_3_, 75 MHz) δ 185.3, 161.2, 144.7, 143.4, 139.9, 133.7, 132.0, 130.8, 130.5, 122.3, 117.1; EI-MS *m*/*z* 259 [M + 2]^+^ (33), 257 [M]^+^ (100); HREIMS *m*/*z* 257.0348 (calcd. for C_13_H_8_ClN_3_O, 257.0356).

*(4-fluorophenyl)(imidazo[1,2-*a*]pyrimidin-3-yl)methanone* (**4j**). Synthesis of **4j** gave a white solid (0.15 g, 62.2% yield); m.p. 256.5–257.5 °C; IR (KBr) υ_max_ 3133 (Csp^2^-H), 1614 (C=O), 1513 (C=C), 1507, 1477 (C=C) cm^−1^. ^1^H NMR (CDCl_3_, 500 MHz) δ 9.96 (1H, dd, *J* = 7 Hz, *J* = 2 Hz, H-5), 8.84 (1H, dd, *J* = 4.5 Hz, *J* = 2.0 Hz, H-7), 8.39 (1H, s, H-2), 7.92–7.96 (2H, m, H-12, H-16), 7.22–7.27 (3H, m, H-6. H-13, H-15); EI-MS *m*/*z* 241 [M]^+^ (100); HREIMS *m*/*z* 241.0647 (calcd. for C_13_H_8_FN_3_O, 241.0651).

### 4.3. Microorganisms and Compounds Tested

**Figure 5:**
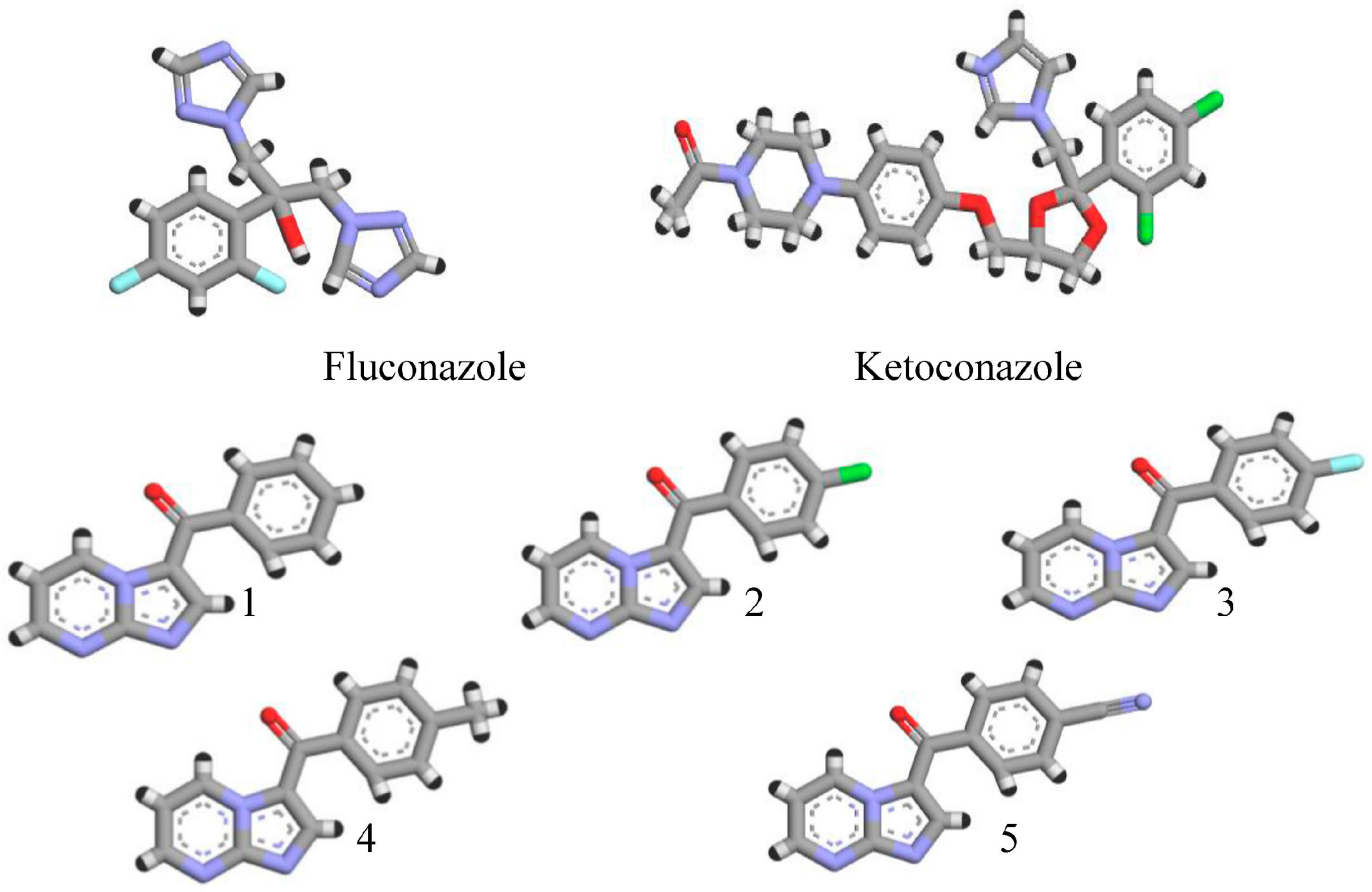
3D representation of the compounds used for biological activity and molecular docking.

The fungal strains included presently were *C. albicans* (ATCC 10231), *C. dubliniensis* (CD36), *C. glabrata* (CBS138), *C. guilliermondii* (ATCC 6260, also called *Meyerozyma guilliermondi*), *C. krusei* (ATCC 6358), *C. tropicalis* (MYA-3404), and *C. kefyr* ENCB-BMBL (also called *Kluyveromyces marxianus*), kindly provided by Lourdes Villa Tanaca of IPN-ENCB. The yeast strains were previously incubated in YPD medium (1% yeast extract, 2% casein peptone, and 2% dextrose) to verify their purity. The five 3-benzoyl imidazo[1,2-*a*]pyrimidines tested herein were selected based on their expected good inhibitory effect. Fluconazole (triazole) and ketoconazole (imidazole) were the reference compounds (Figure 5).

### 4.4. Homology Modeling

The amino acid sequences of lanosterol 14α-demethylase (CYP51) proteins of the *Candida* spp. were downloaded from the NCBI database: (*C. dubliniensis* XP_002420370.1), (*C. guilliermondii* XP_001484034.1), (*C. kefyr* AHL25033.1), (*C. krusei* XP_020542549.1), and (*C. tropicalis* XP_002550985.1). The 3D models were generated with the Modeller 9.10 program [49] (http://www.salilab.org/modeller/), using as a template the crystallized structures of CYP51 of *C. glabrata* (PDB: 5JLC) and *C. albicans* (PDB: 5V5Z), respectively, deposited in the protein data bank (PDB) [50] (http://www.rcsb.org./pdb). The structures were selected based on the highest percentage of identity with the downloaded sequences. An overlapping of the structures was constructed and displayed with Discovery 4.0 Client [51].

The quality of the models was evaluated with the DOPE (discrete optimized protein energy) method [31]. The model having the lowest DOPE score was selected for ligand–protein interaction studies. In addition, Ramachandran plots [39] were calculated by using the PDBsum database [52] for validation of the 3D structure.

### 4.5. Molecular Docking Studies

A ligand–protein interaction study was previously validated with molecular docking software Autodock version 4.0 (The Scripps Research Institute, La Jolla, CA, USA) [53]. The 2D structure of each ligand was sketched in editor chemical MedChem Designer 3.0 (http://www.simulations-plus.com/software/medchem-designer) and converted to 3D, mol2 format in the Open Babel GUI program [54]. Hydrogens were added to the models generated with the MolProbity program [55] and prepared with Visual Molecular Dynamics (VMD 1.9.1) [56]. All ions were added by utilizing the optimization Nanoscale Molecular Dynamics (NAMD) software program (Illinois University, Urbana and Champaign, IL. USA) [57]. The resulting structures were used for docking.

The selected test compounds and the reference compounds (fluconazole and ketoconazole) were docked in the active site of CYP51. For the preparation of docking, the following parameters were estimated in AutoDock Tools (ADT) [53]. The grid dimensions were 48 × 42 × 40 Å^3^ and the points were separated by 0.375 Å. The following grid centers were calculated for CYP51 from *C. albicans* (CYP51_Ca_; X = −47.731, Y = −13.422 and Z = 22.982), *C. dubliniensis* (CYP51_Cd_; X = −43.598, Y = −13.588 and Z = 25.836), *C. glabrata* (CYP51_Cg_; X = −31.107, Y = 68.515 and Z = −21.415), *C. kefyr* (CYP51_Cke_; X = −31.107, Y = 69.159 and Z = −19.206), *C. krusei* (CYP51_Ckru_; X = −43.311, Y = −9.941 and Z = 25.384), and *C. tropicalis* (CYP51_Ct_; X = −45.446, Y = −10.26 and Z = 23.625).

Random starting positions, orientations and torsion angles were established for all ligands. Default values of translation, quaternation, and torsion steps were employed for the simulation. The hybrid Lamarckian Genetic Algorithm (set with default parameters) was applied for minimization. The number of docking runs was 100. The docked model with the lowest binding energy was considered for all further simulations. Docking results were analyzed in AutoDockTools and edited in Discovery 4.0 Client [51].

### 4.6. Antifungal Activity Tests

The minimum inhibitory concentration (MIC50) was determined according to CLSI guidelines in the document M27-A3 for yeasts [35]. The preparation of the dilutions of the reference compounds and five 3-benzoyl imidazo[1,2-*a*]pyrimidines selected was carried out by the method of serial double additive dilutions. For the water-soluble compound (fluconazole), the concentrations tested were 64–0.125 μg/mL, using RPMI 1640 as diluent with glutamine and without sodium bicarbonate, buffered with morpholino propane sulfonic acid (MOPS) at 0.164 M, adjusted to pH 7 ± 0.1, and with 0.2% glucose. For the water insoluble antifungals (ketoconazole and the test compounds), the concentrations ranged from 16 to 0.0312 μg/mL, using DMSO as diluent.

For the preparation of the inoculum of *Candida* spp., the optical density was adjusted in a spectrophotometer (530 nm) to 0.5 McFarland. Subsequently, a 1:1000 dilution was made with RPMI medium (at concentrations of 1 × 10^3^–5 × 10^3^). The antifungal assay was performed with the latter dilution. The 96-well plates were inoculated with 100 µL of yeast suspension. RPMI was utilized as the sterility control and DMSO without antifungal as the growth control. The plates were incubated for 24 h at 37 °C. The optical density was determined in a Multiskan™ GO microplate spectrophotometer by agitation of the plates to obtain a homogeneous suspension, followed by a spectrophotometric reading at 530 nm. The MIC50 is the antifungal concentration whose optical density equals 50% of the growth in the control well. The value reported herein represents the average of three different experiments.

## 5. Conclusions

A series of 3-benzoyl imidazo[1,2-*a*]pyrimidines was synthesized and tested in silico and in vitro. By docking the test compounds in the active site of fungal CYP51, the binding mode and binding energy could be predicted in each case. The MIC50 was determined for each compound as well as for two reference compounds (fluconazole and ketoconazole), in order to evaluate the respective capacity for growth inhibition of distinct *Candida* species. The docking results show that for each species of *Candida* spp., the binding mode of each test compound shares at least three amino acid residues with the reference drugs. We analyzed and described the interactions of the electron-donor and electron-withdrawing substituents in the aromatic ring with key amino acid side chains in the active site of CYP450. Although the antifungal activity of imidazo[1,2-*a*]pyrimidines has been studied, the present findings should certainly be instrumental in the design and development of new antifungal drugs derived from 3-benzoyl imidazo[1,2-*a*]pyrimidines. Further research is needed for this purpose, and to test new derivatives in combination with conventional drugs in animal models, and possibly later, in clinical trials as promising candidates for antifungal therapy.

## Figures and Tables

**Table 1:**
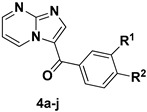
Synthesized 3-benzoyl imidazo[1,2-*a*]pyrimidines **4a**–**j**.

Entry	Compound	R^1^	R^2^	Yield (%)
1	**4a**	H	H	77
2	**4b**	OMe	OMe	81
3	**4c**	H	OMe	67
4	**4d**	H	Me	90
5	**4e**	H	Ph	70
6	**4f**	H	CN	84
7	**4g**	H	NO_2_	74
8	**4h**	H	Br	98
9	**4i**	H	Cl	72
10	**4j**	H	F	62

**Table 2:**
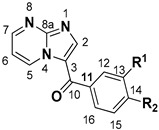
^1^H NMR spectral data for synthesized 3-benzoylimidazo[1,2-*a*]pyrimidines.

Compound	H-2	H-5	H-6	H-7	H-12	H-13	H-14	H-15	H-16
**4a** *	8.41	10.0	7.23	8.84	7.90	7.57	7.65	7.57	7.90
**4b** *	8.39	9.88	7.18	8.78	7.43	3.94	3.96	6.96	7.54
**4c** *	8.37	9.90	7.17	8.78	7.88	7.00	3.90	7.00	7.88
**4d** *	8.37	9.94	7.19	8.79	7.77	7.32	2.48	7.32	7.77
**4e** **	8.53	10.08	7.74	9.08	7.87	7.74	7.29–7.52	7.74	7.87
**4f** **	8.51	10.10	7.77	9.13	7.62	7.62	-	7.62	7.62
**4g** **	8.52	10.08	7.76	9.10	7.96	8.29	-	8.29	7.96
**4h** **	8.43	10.00	7.67	9.04	7.67	7.40	-	7.40	7.67
**4i** **	8.42	10.00	7.68	9.03	7.68	7.41	-	7.41	7.68
**4j** **	8.39	9.96	7.92–7.96	8.84	7.92–7.96	7.22–7.27	-	7.22–7.27	7.92–7.96

* ^1^H NMR determined in CDCl_3_; ** ^1^H NMR ascertained in CF_3_COOD.

**Table 3:** Docking results of 3-benzoyl imidazo[1,2-*a*]pyrimidines in the active site of CYP51 of *Candida* spp.

Compound	Binding Energy (kcal/mol) inCYP51 from *Candida* spp.						
	A	B	C	D	E	F	G
**Fluconazole**	−4.44	−4.56	−4.38	−4.07	−3.16	−5.68	−4.99
**Ketoconazole**	32.55	−1.95	−6.16	−5.5	48.93	1.44	3.92
**4a**	−7.65	−7.53	−7.1	−7.21	−6.85	−8.41	−7.86
**4d**	−8.06	−7.87	−7.64	−7.79	−6.34	−9.09	−8.52
**4f**	−8.43	−7.91	−7.61	−7.89	−6.11	−9.43	−8.39
**4i**	−8.02	−7.74	−7.73	−7.87	−6.21	−9.26	−8.45
**4j**	−7.8	−7.59	−7.13	−7.19	−7.17	−8.5	−8.02

Fluconazole and ketoconazole (reference compounds). **A**, CYP51_Ca_; **B**, CYP51_Cd_; **C**, CYP51_Cg_; **D**, CYP51_Cgui_; **E**, CYP51_Cke_; **F**, CYP51_Ck_; and **G**, CYP51_Ct_.

**Table 4:** Antifungal activity of compounds **4a**, **4d**, **4f**, **4i**, and **4j** against the *Candida* species.

Compound	MIC50 (µg/mL)						
	A	B	C	D	E	F	G
**Fluconazole**	0.5	0.5	4	1	0.5	4	1
**Ketoconazole**	8	2	0.125	0.25	0.25	1	2
**4a**	0.125	0.0625	0.0625	0.0625	0.125	0.5	0.5
**4d**	0.125	0.25	0.0625	0.0625	0.5	0.5	0.25
**4f**	0.25	0.0625	0.0312	0.0625	0.125	0.25	0.0625
**4i**	0.25	0.5	0.0625	1	0.125	0.5	0.0312
**4j**	0.0625	0.5	0.25	0.0312	0.125	0.5	0.25

Fluconazole and ketoconazole (reference compounds). **A**, *Candida albicans* (ATCC 10231); **B**, *Candida dubliniensis* (CD36); **C**, *Candida glabrata* (CBS138); **D**, *Candida guilliermondii* (ATCC 6260); **E**, *Candida kefyr*; **F**, *Candida krusei* (ATCC 6358); and **G**, *C. tropicalis* (MYA-3404).

## Supplementary Materials

The supplementary materials are available online. Figures S1–S103.
