# Supplementary material for: Synthesis, Molecular Docking, and Antimycotic Evaluation of Some 3-Acyl Imidazo[1,2-a]pyrimidines
Source: Molecules. 2018 Mar 7;23(3):599. doi: 10.3390/molecules23030599 (PMC6017402; doi:10.3390/molecules23030599)
Supplement: Supplementary file 1 [file molecules-23-00599-s001.pdf]

Synthesis, molecular docking and antimycotic evaluation of some 3-acylimidazo[1,2-*a*]pyrimidines

Omar Gómez-García[1,*], Dulce Andrade-Pavón[2,*,] Elena Campos-Aldrete[1]

Ricardo Ballinas-Indilí[3], Alfonso Méndez-Tenorio[4], Lourdes Villa-Tanaca[2], Cecilio Álvarez-Toledano[3,*]

1 Departamento de Química Orgánica-Laboratorio de Investigación 6 de Síntesis de Heterociclos, Escuela Nacional de Ciencias Biológicas-IPN, Prolongación de Carpio y Plan de Ayala S/N, Colonia Santo Tomás, 11340, Ciudad de México, México. Email: jogomezga@ipn.mx

2 Departamento de Microbiología-Laboratorio de Biología Molecular de Bacterias y Levaduras, Escuela Nacional de Ciencias Biológicas-IPN, Prolongación de Carpio y Plan de Ayala S/N, Colonia Santo Tomás, 11340, Ciudad de México, México. Email: andrade_eclud88@hotmail.com

3 Instituto de Química-UNAM, Circuito Exterior, Ciudad Universitaria, Coyoacán, C.P. 04510, Ciudad de México, México.

4 Departamento de Bioquímica-Laboratorio de Biotecnología y Bioinformática Genómica, Escuela Nacional de Ciencias Biológicas-IPN, Prolongación de Carpio y Plan de Ayala S/N, Colonia Santo Tomás, 11340, Ciudad de México, México.

Supporting Information

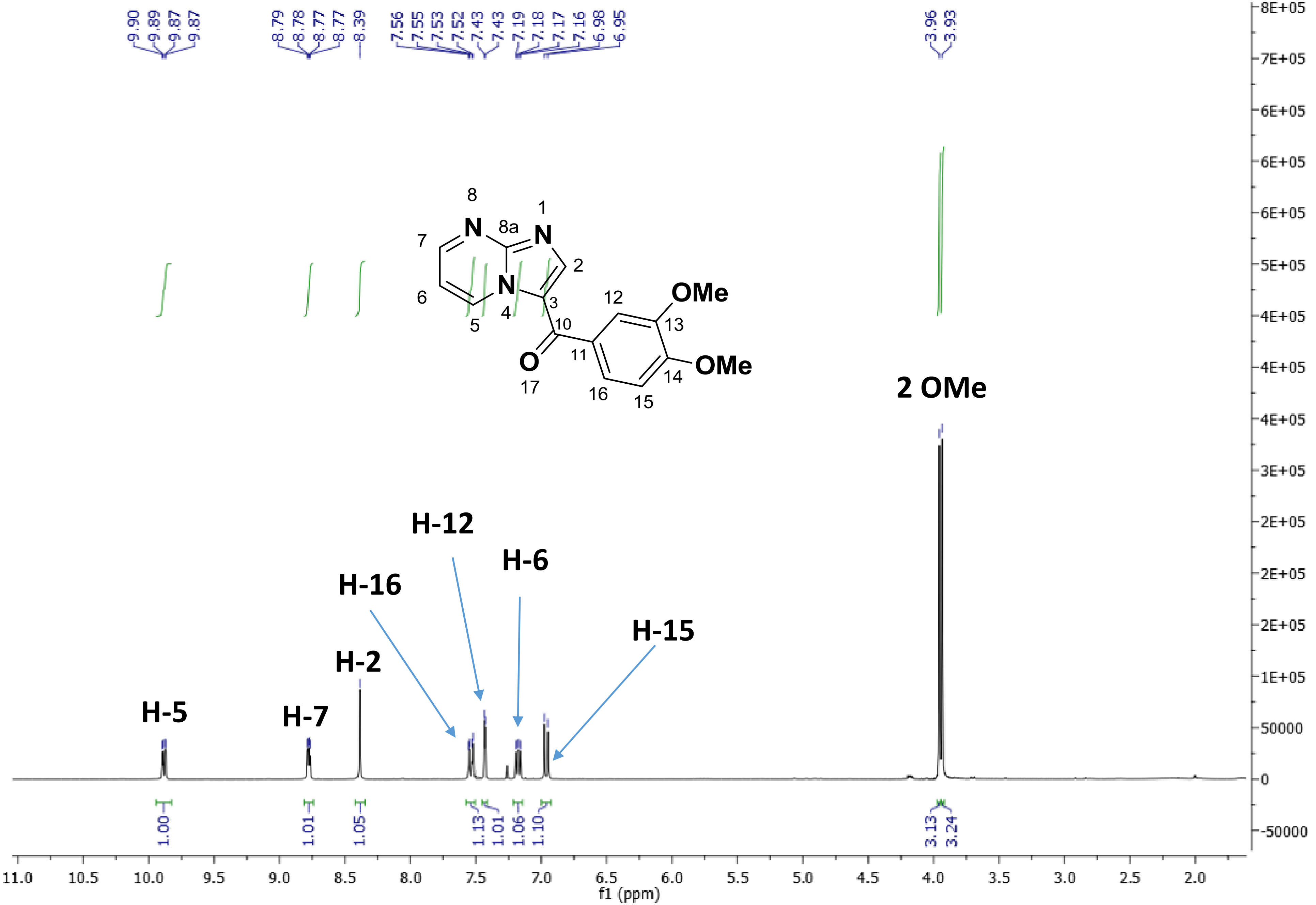

Figure S1: ^{1}H NMR (300 MHz, $CDCl_3$) of (3,4-dimethoxyphenyl)(imidazo[1,2-*a*]pyrimidin-3-yl)methanone (**4b**).

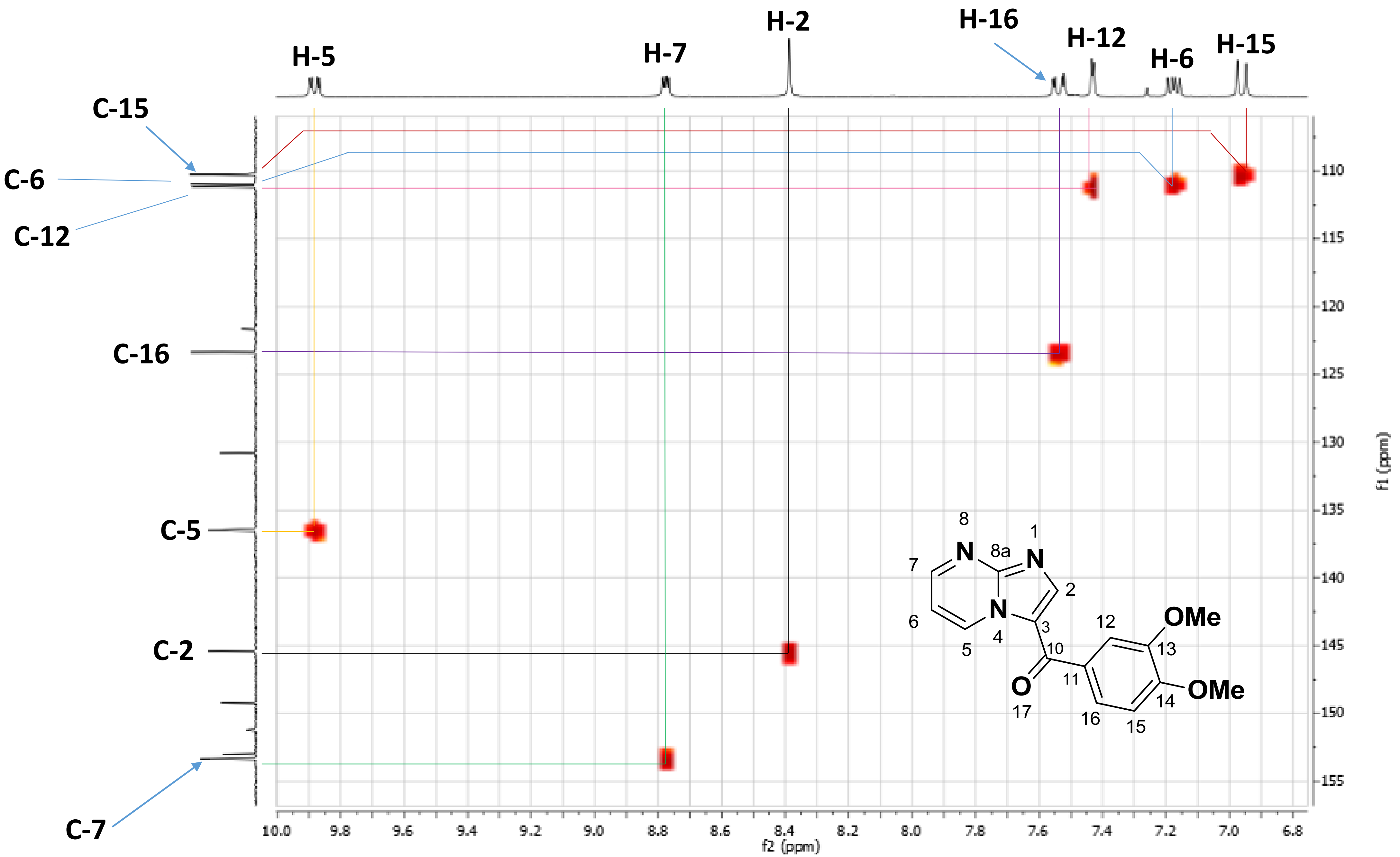

Figure S2: HSQC experiment of (3,4-dimethoxyphenyl)(imidazo[1,2-*a*]pyrimidin-3-yl)methanone (**4b**).

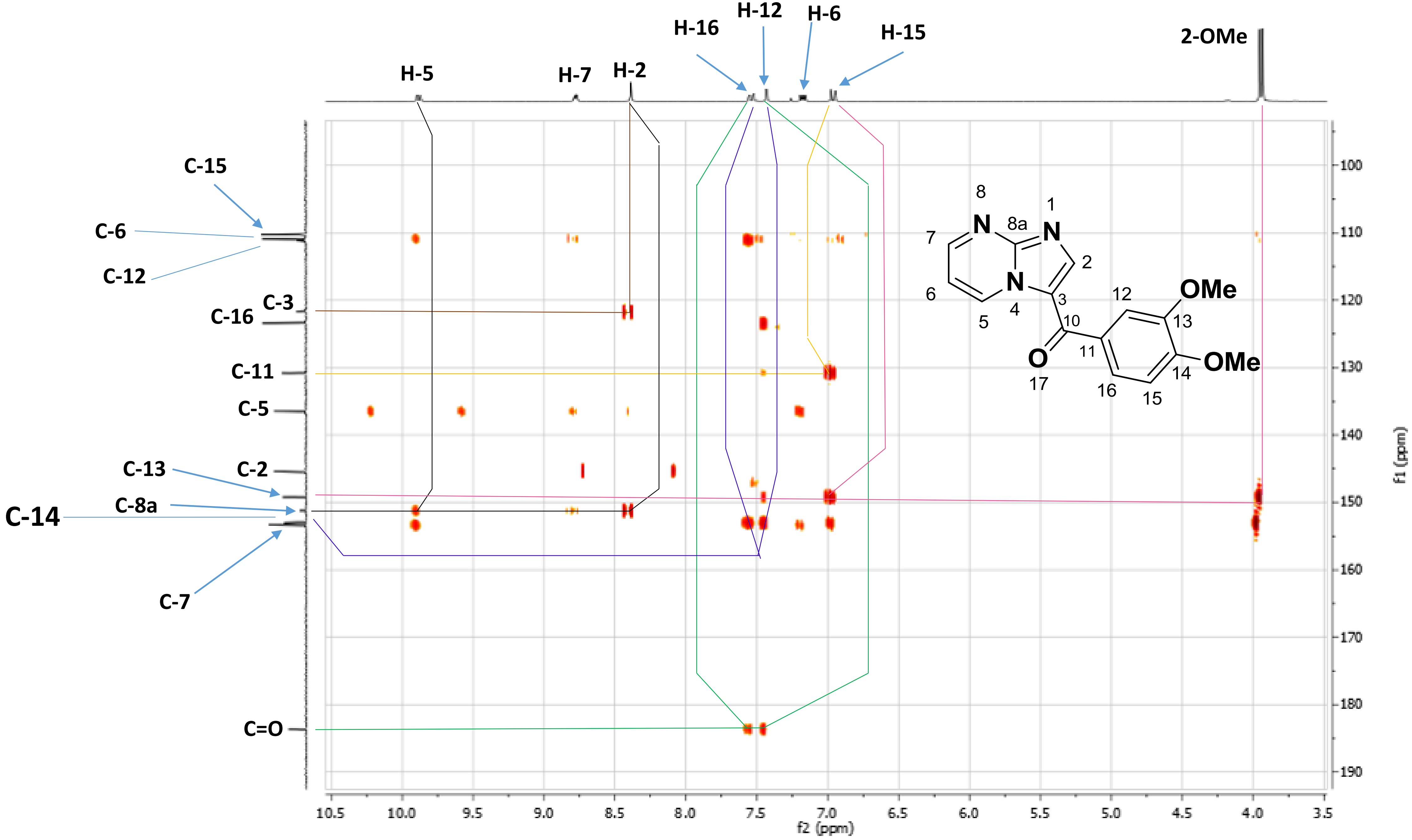

Figure S3: HMBC experiment of (3,4-dimethoxyphenyl)(imidazo[1,2-*a*]pyrimidin-3-yl)methanone (**4b**).

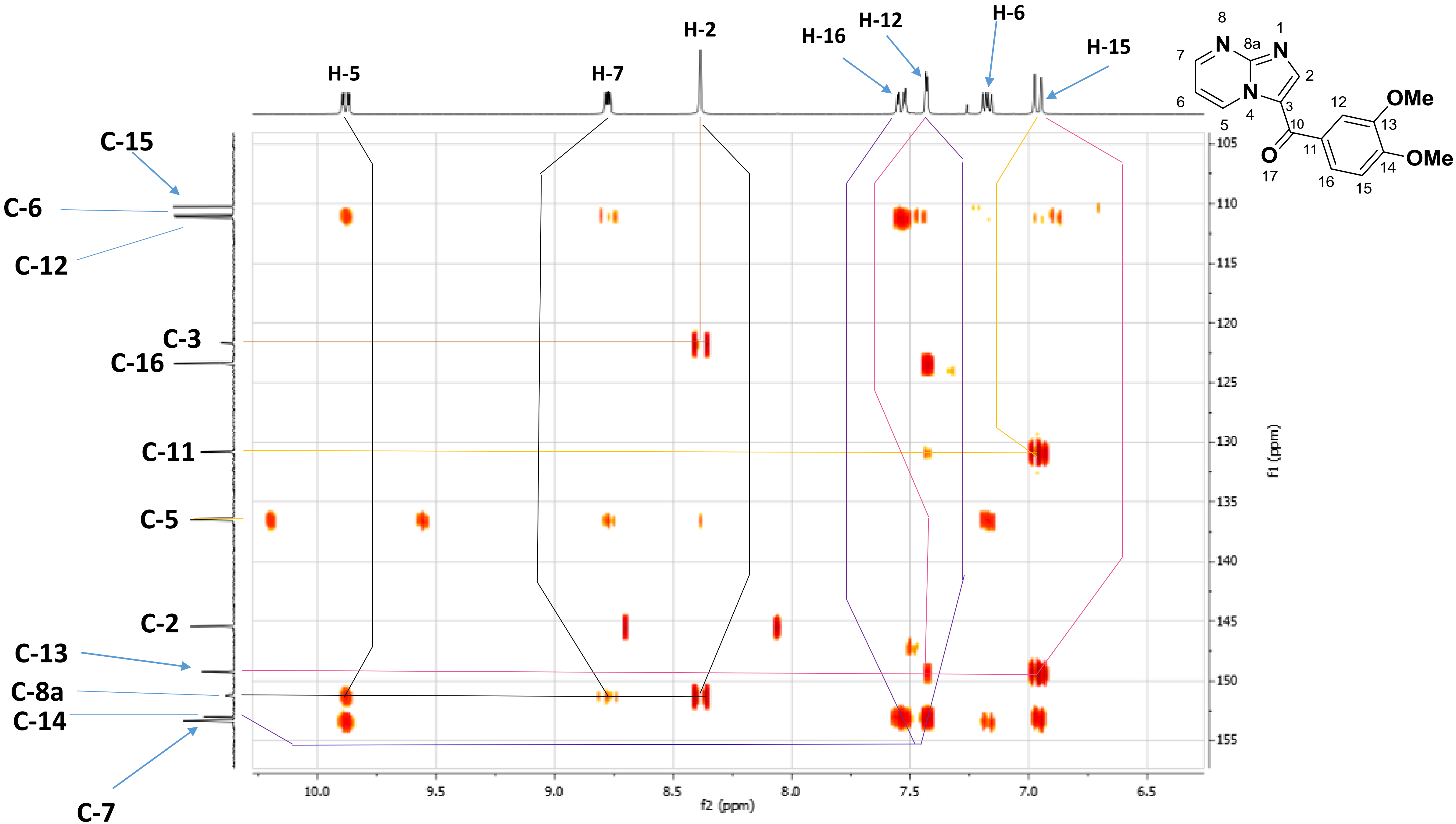

Figure S4: HMBC experiment of (3,4-dimethoxyphenyl)(imidazo[1,2-*a*]pyrimidin-3-yl)methanone (**4b**).

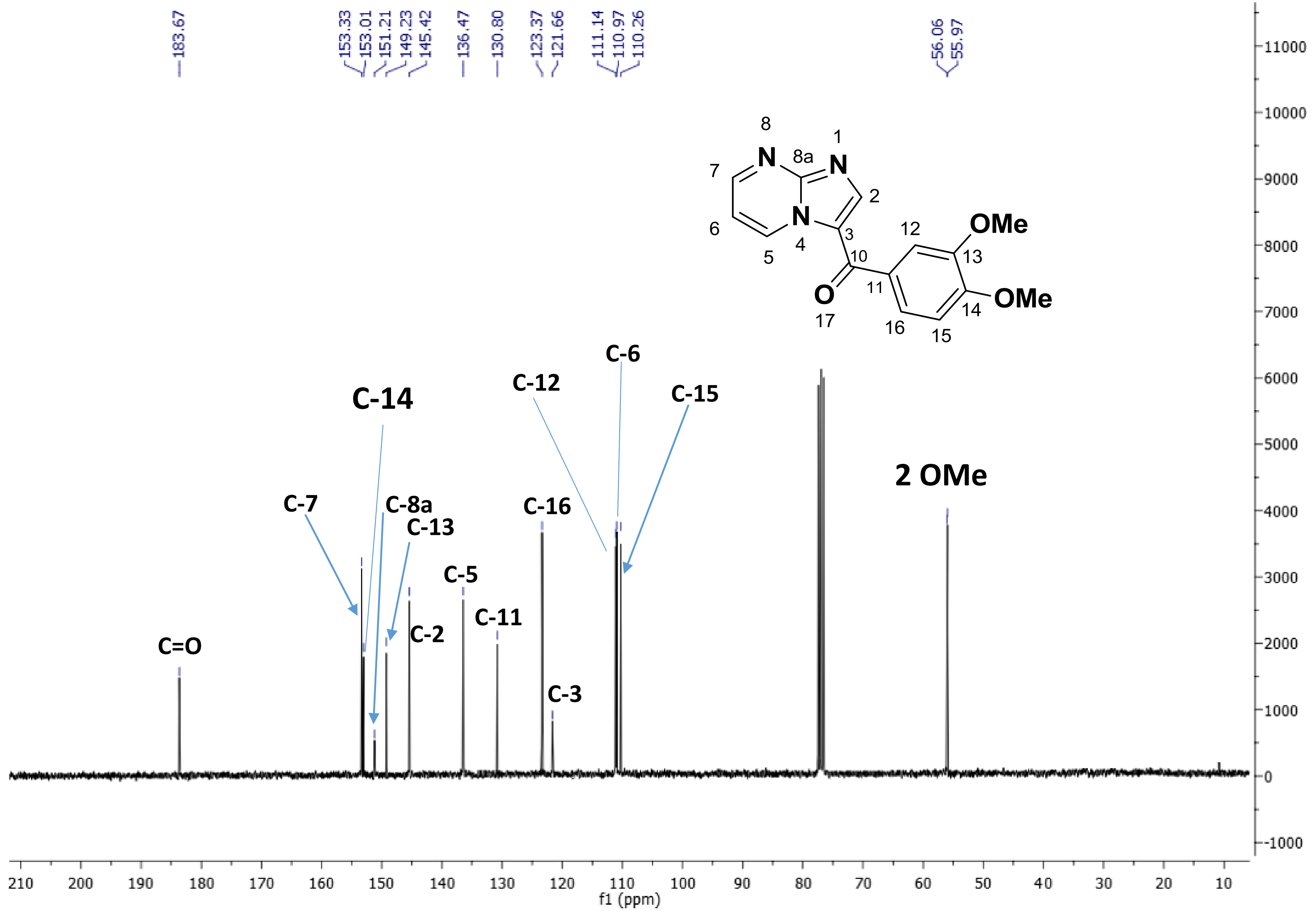

Figure S5: ^{13}C NMR (75 MHz, $CDCl_3$) of (3,4-dimethoxyphenyl)(imidazo[1,2-*a*]pyrimidin-3-yl)methanone (**4b**).

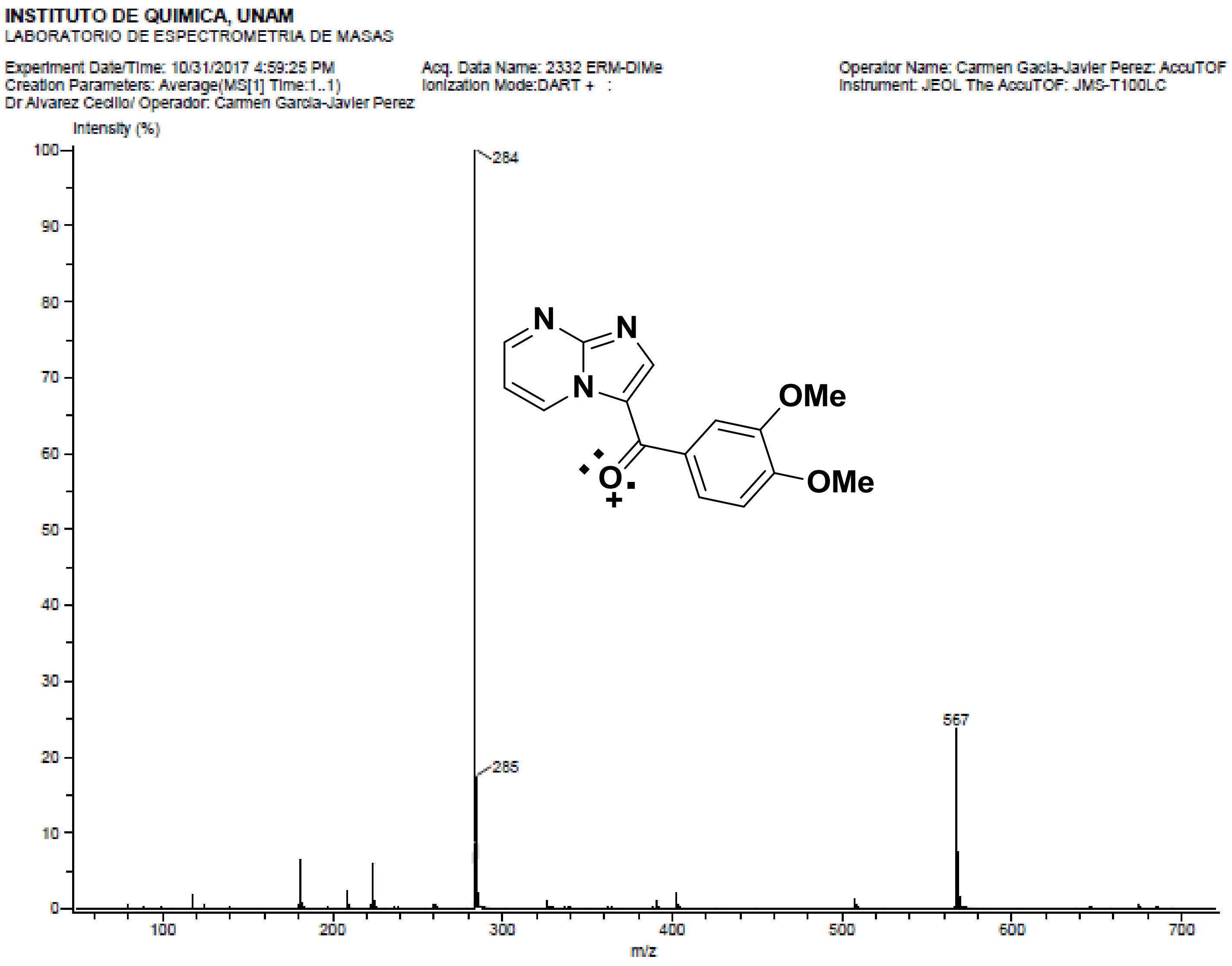

Figure S6: Mass spectrum of (3,4-dimethoxyphenyl)(imidazo[1,2-*a*]pyrimidin-3-yl)methanone (**4b**).

Data:2332 ERM-DIMe
Sample Name:Dr Alvarez Cecilio/ Operador: Carmen Garcia-Javier Perez
Description:
Ionization Mode:ESI+
History:Determine m/z[Peak Detect[Centroid,30,Area];Correct Base[10.0%]];Correct Base[5.0%];Average(MS[1] 1..1)

Acquired:10/31/2017 4:59:25 PM
Operator:AccuTOF
Mass Calibration data:Cal Peg 600
Created:11/15/2017 12:52:55 PM
Created by:

Charge number:1
Tolerance:5.00(mmu)
Unsaturation Number:0.0 .. 50.0 (Fraction:Both)
Element:^{12}C:0 .. 15, ^{1}H:0 .. 40, ^{14}N:0 .. 4, ^{16}O:0 .. 3

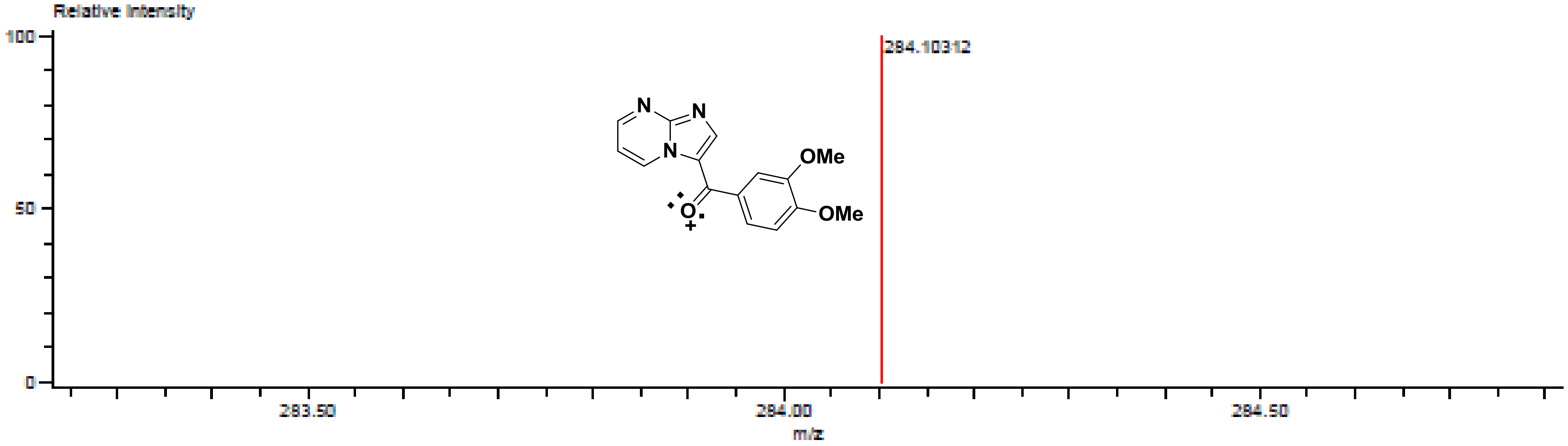

Mass	Intensity	Calc. Mass	Mass Difference (mmu)	Mass Difference (ppm)	Possible Formula	Unsaturation Number
284.10312	328233.63	284.10352	-0.40	-1.40	$^{12}C_{15}{}^{1}H_{14}{}^{14}N_{3}{}^{16}O_{3}$	10.5

Figure S7: HRMS of (3,4-dimethoxyphenyl)(imidazo[1,2-*a*]pyrimidin-3-yl)methanone (**4b**).

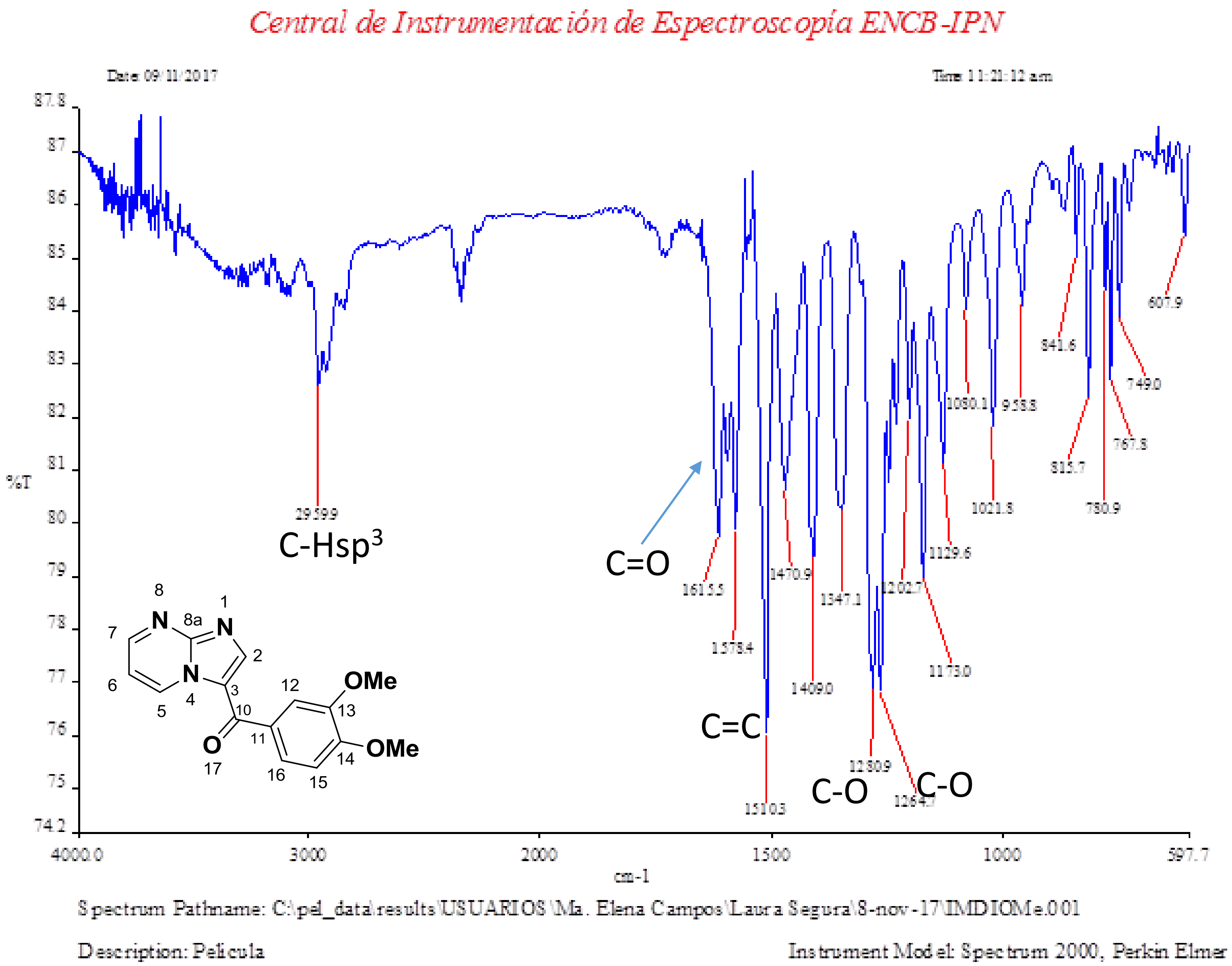

Figure S8: IR spectrum of (3,4-dimethoxyphenyl)(imidazo[1,2-*a*]pyrimidin-3-yl)methanone (**4b**).

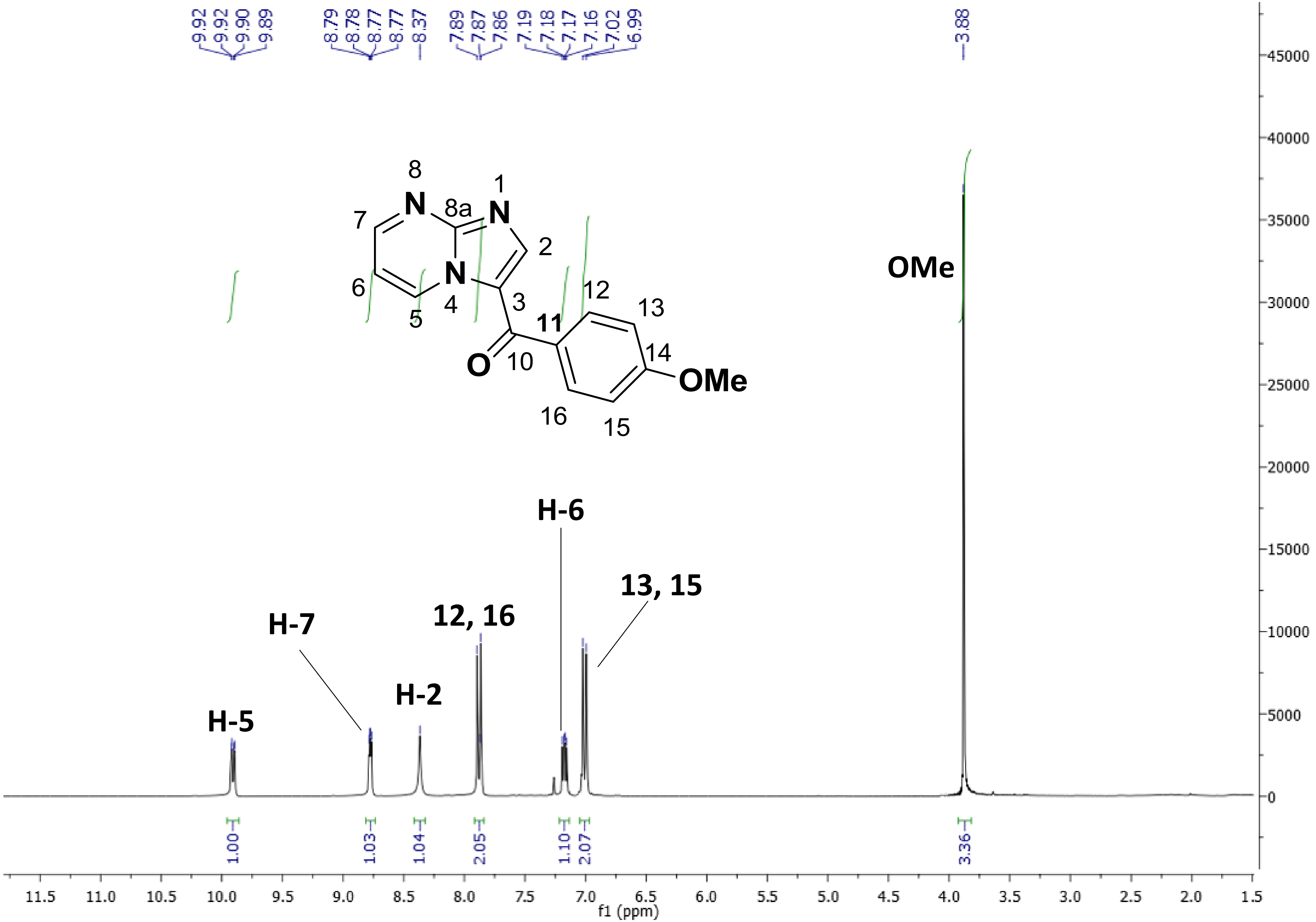

Figure S9: ^{1}H NMR (300 MHz, $CDCl_3$) of imidazo[1,2-*a*]pyrimidin-3-yl(4-methoxyphenyl)methanone (**4c**).

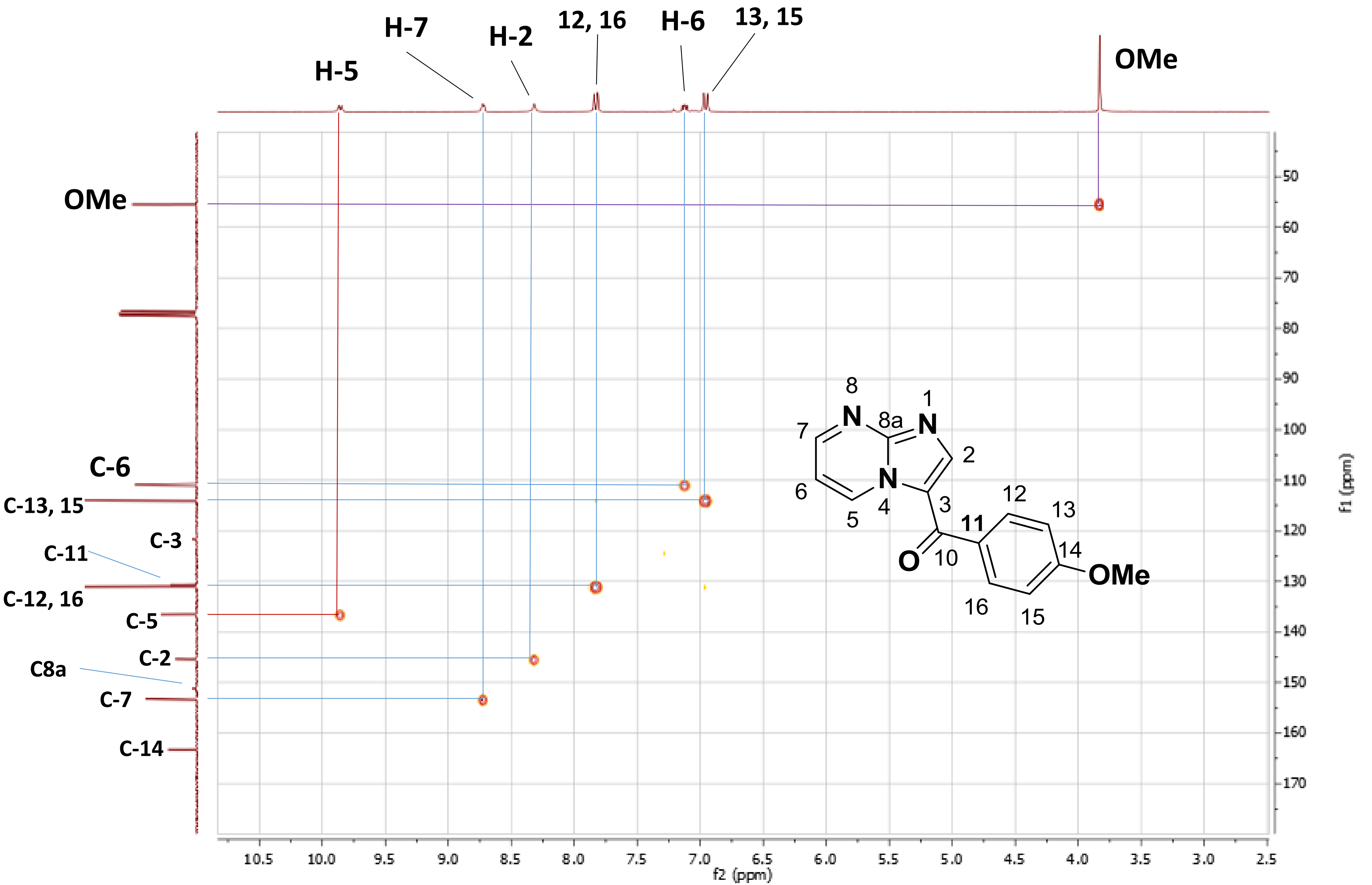

Figure S10: HSQC experiment of imidazo[1,2-*a*]pyrimidin-3-yl(4-methoxyphenyl)methanone (**4c**).

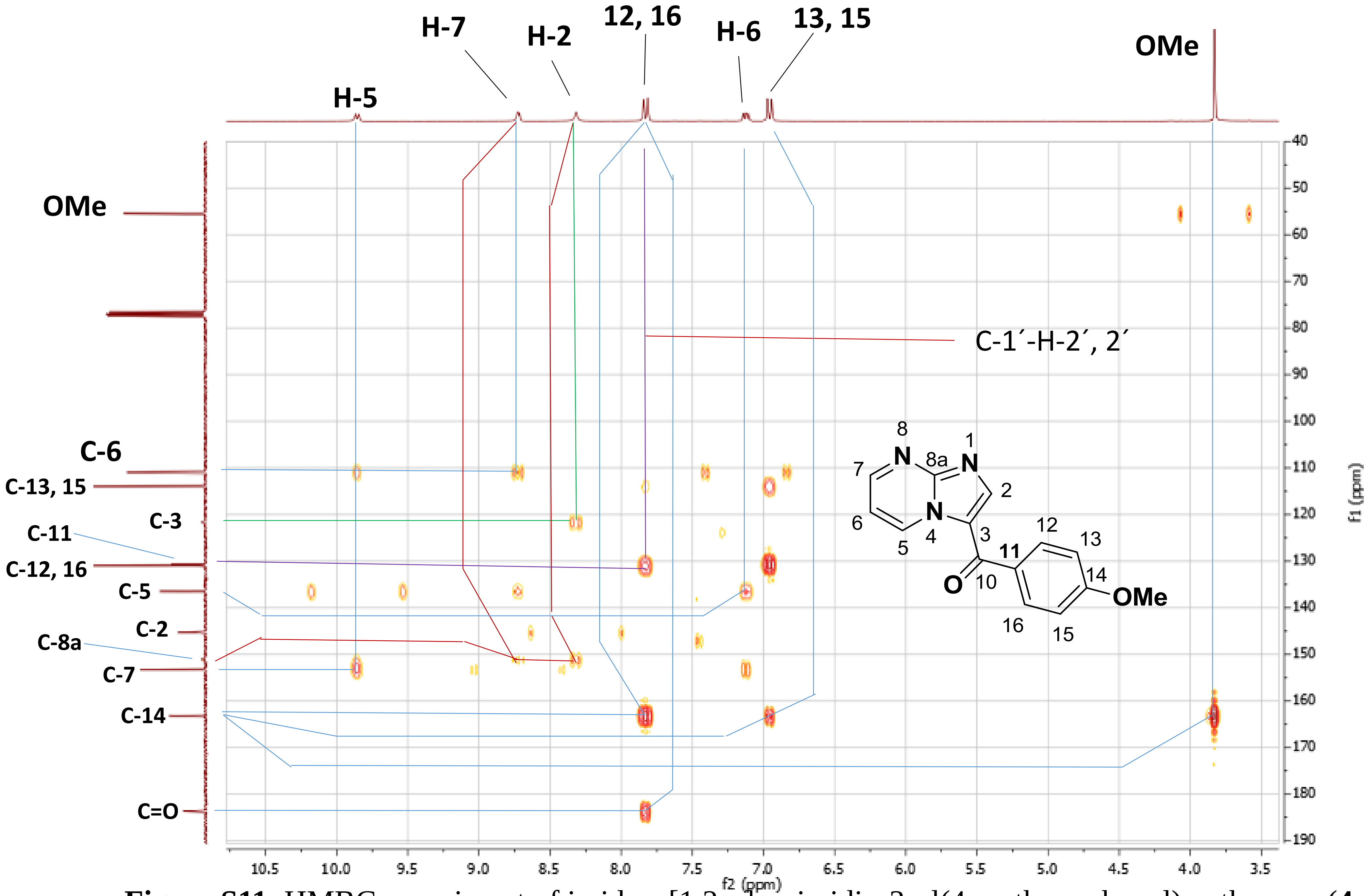

Figure S11: HMBC experiment of imidazo[1,2-*a*]pyrimidin-3-yl(4-methoxyphenyl)methanone (**4c**).

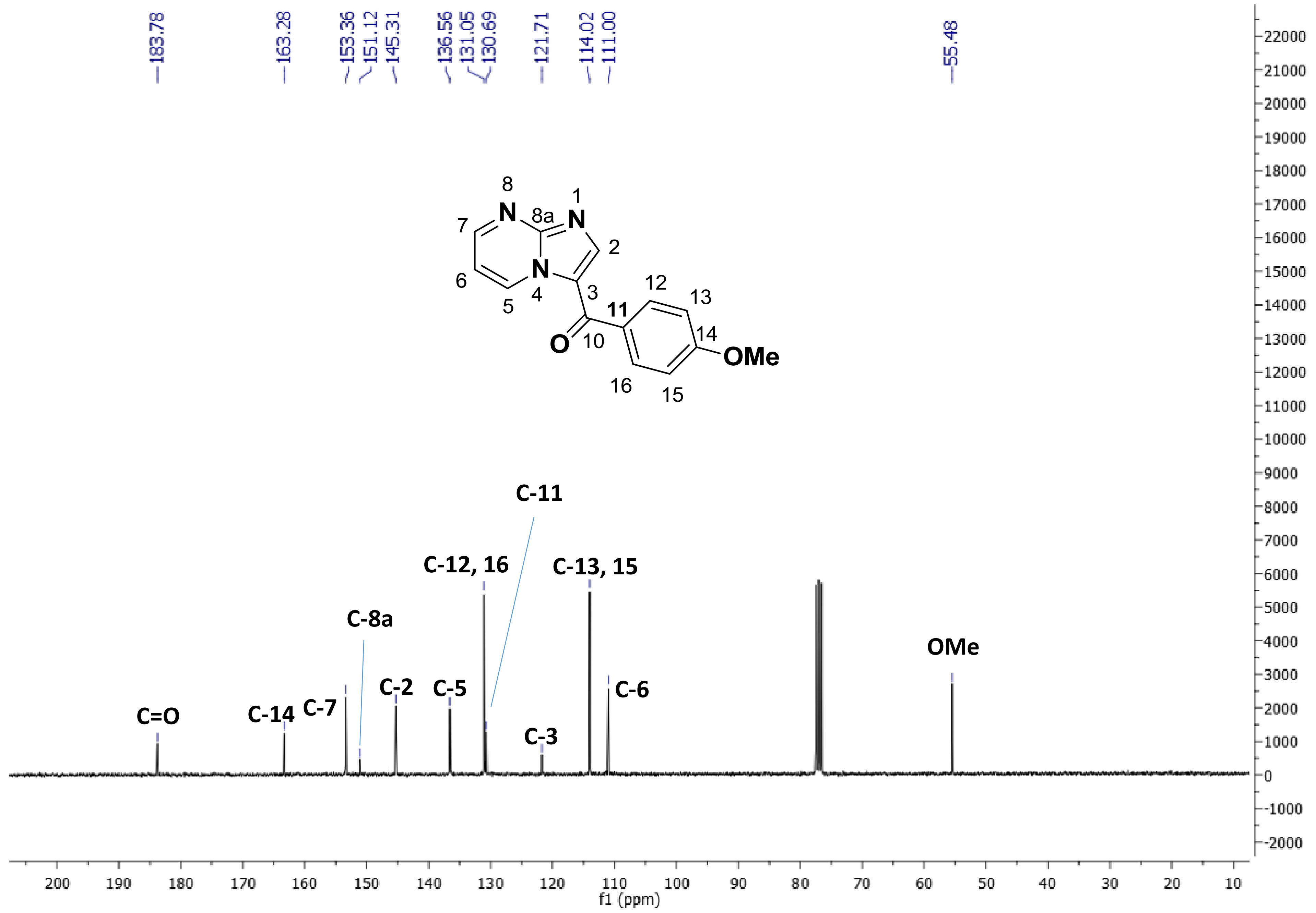

Figure S12: ^{13}C NMR (75 MHz, $CDCl_3$) of imidazo[1,2-*a*]pyrimidin-3-yl(4-methoxyphenyl)methanone (**4c**).

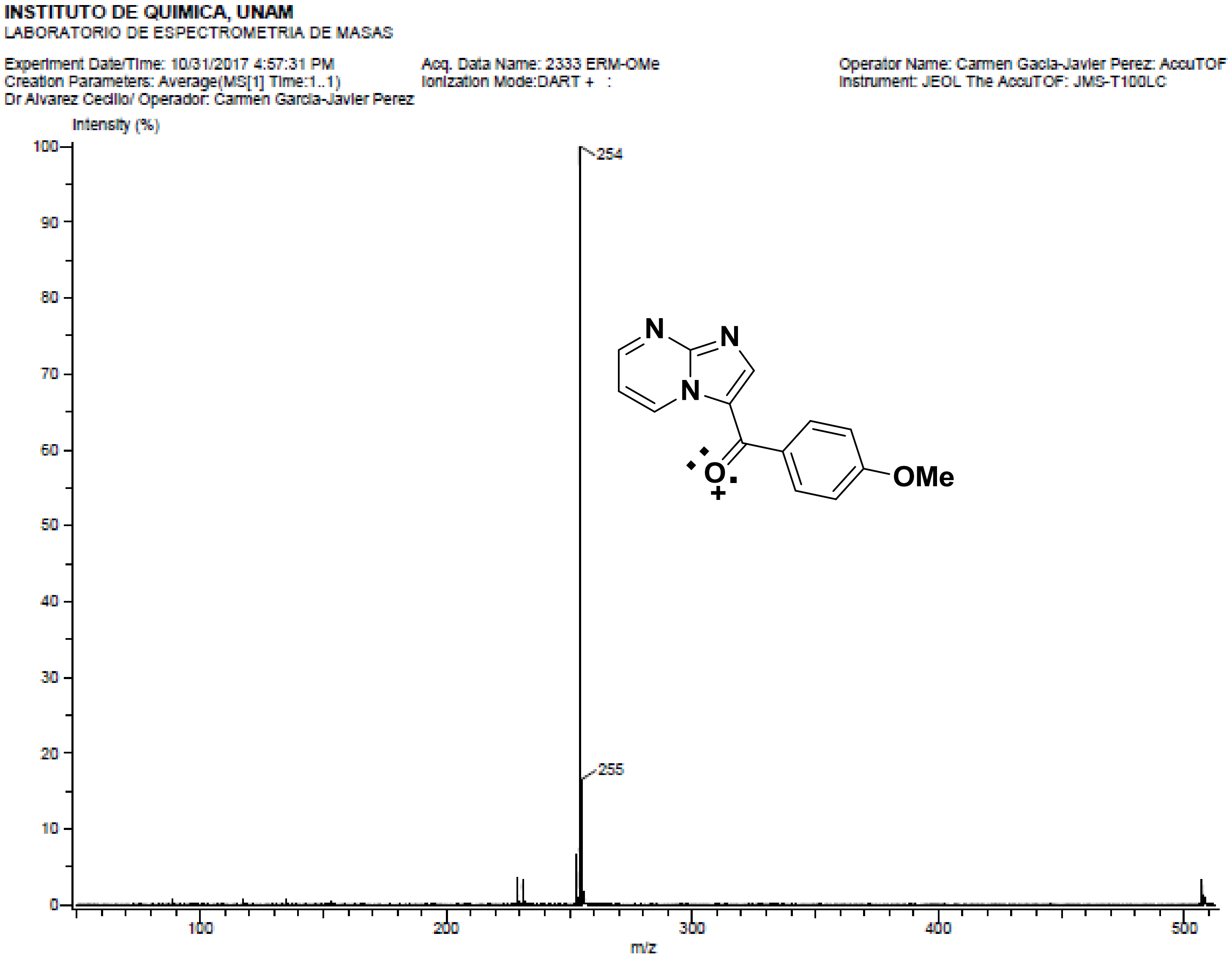

Figure S13: Mass spectrum of imidazo[1,2-*a*]pyrimidin-3-yl(4-methoxyphenyl)methanone (**4c**).

Data:2333 ERM-OMe
Sample Name:Dr Alvarez Cecilio/ Operador: Carmen Garcia-Javier Perez
Description:
Ionization Mode:ESI+
History:Determine m/z[Peak Detect[Centroid,30,Area];Correct Base[10.0%]];Correct Base[5.0%];Average(MS[1] 0..0)

Acquired:10/31/2017 4:57:31 PM
Operator:AccuTOF
Mass Calibration data:Cal Peg 600
Created:11/15/2017 12:38:06 PM
Created by:

Charge number:1
Tolerance:5.00(mmu)
Unsaturation Number:0.0 .. 50.0 (Fraction:Both)
Element:^{12}C:0 .. 15, ^{1}H:0 .. 40, ^{14}N:0 .. 4, ^{16}O:0 .. 2

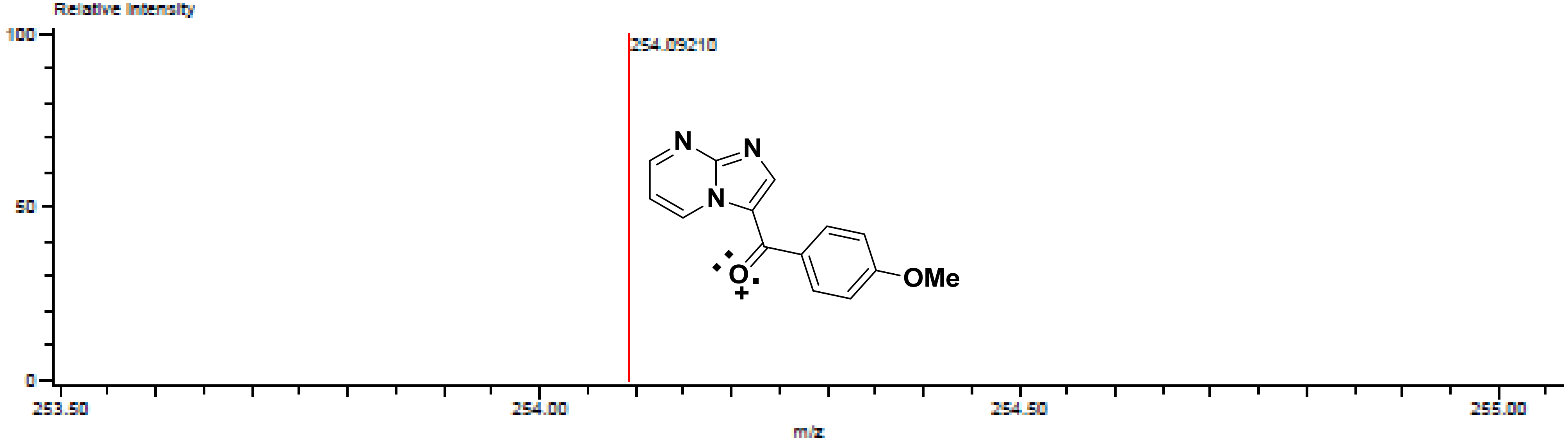

Mass	Intensity	Calc. Mass	Mass Difference (mmu)	Mass Difference (ppm)	Possible Formula	Unsaturation Number
254.09210	2038612.25	254.09295	-0.85	-3.35	$^{12}C_{14}{}^{1}H_{12}{}^{14}N_{3}{}^{16}O_{2}$	10.5

Figure S14: HRMS of imidazo[1,2-*a*]pyrimidin-3-yl(4-methoxyphenyl)methanone (**4c**).

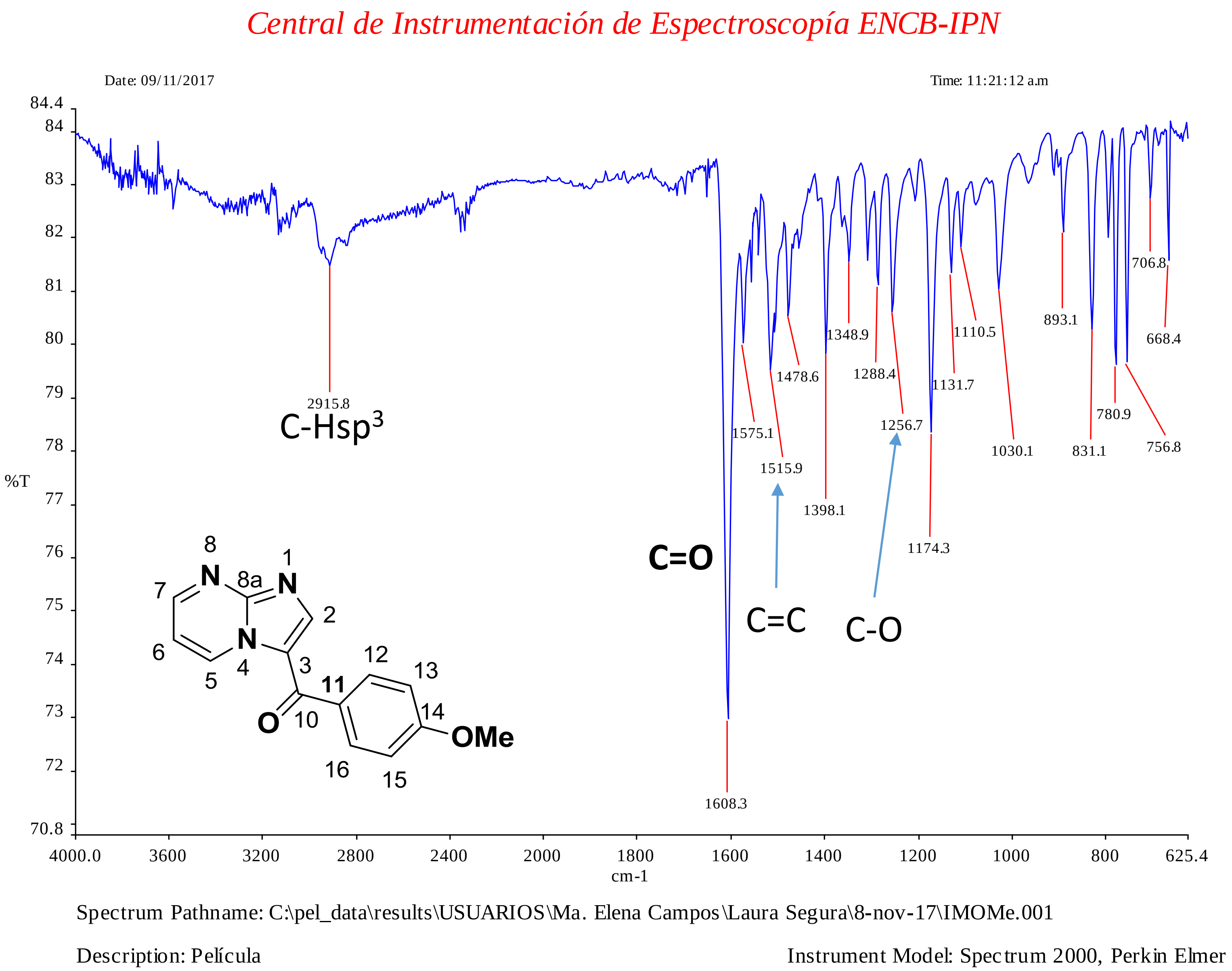

Figure S15: IR spectrum of imidazo[1,2-*a*]pyrimidin-3-yl(4-methoxyphenyl)methanone (**4c**).

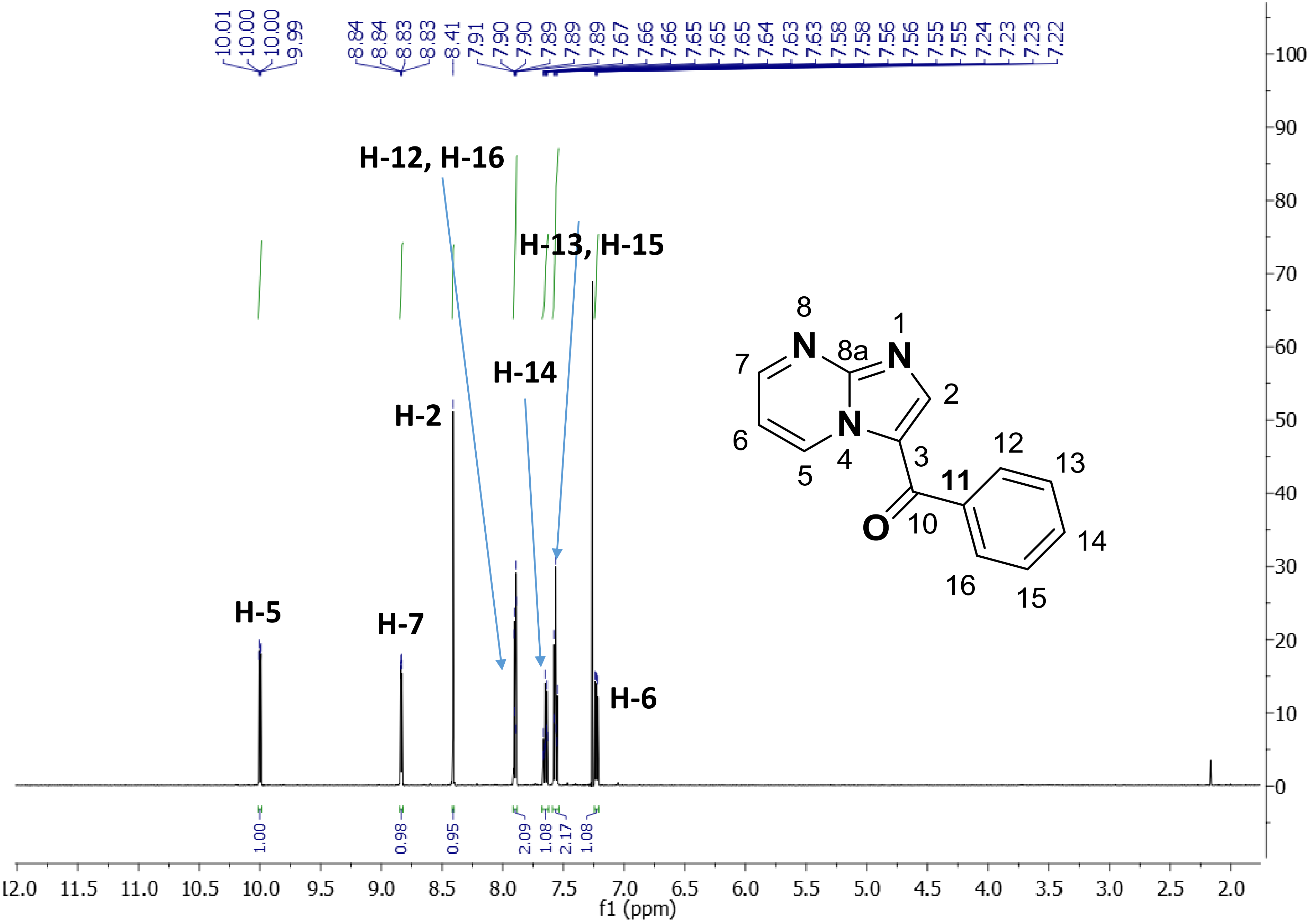

Figure S16: ^{1}H NMR (500 MHz, $CDCl_3$) of imidazo[1,2-*a*]pyrimidin-3-yl(phenyl)methanone (**4a**).

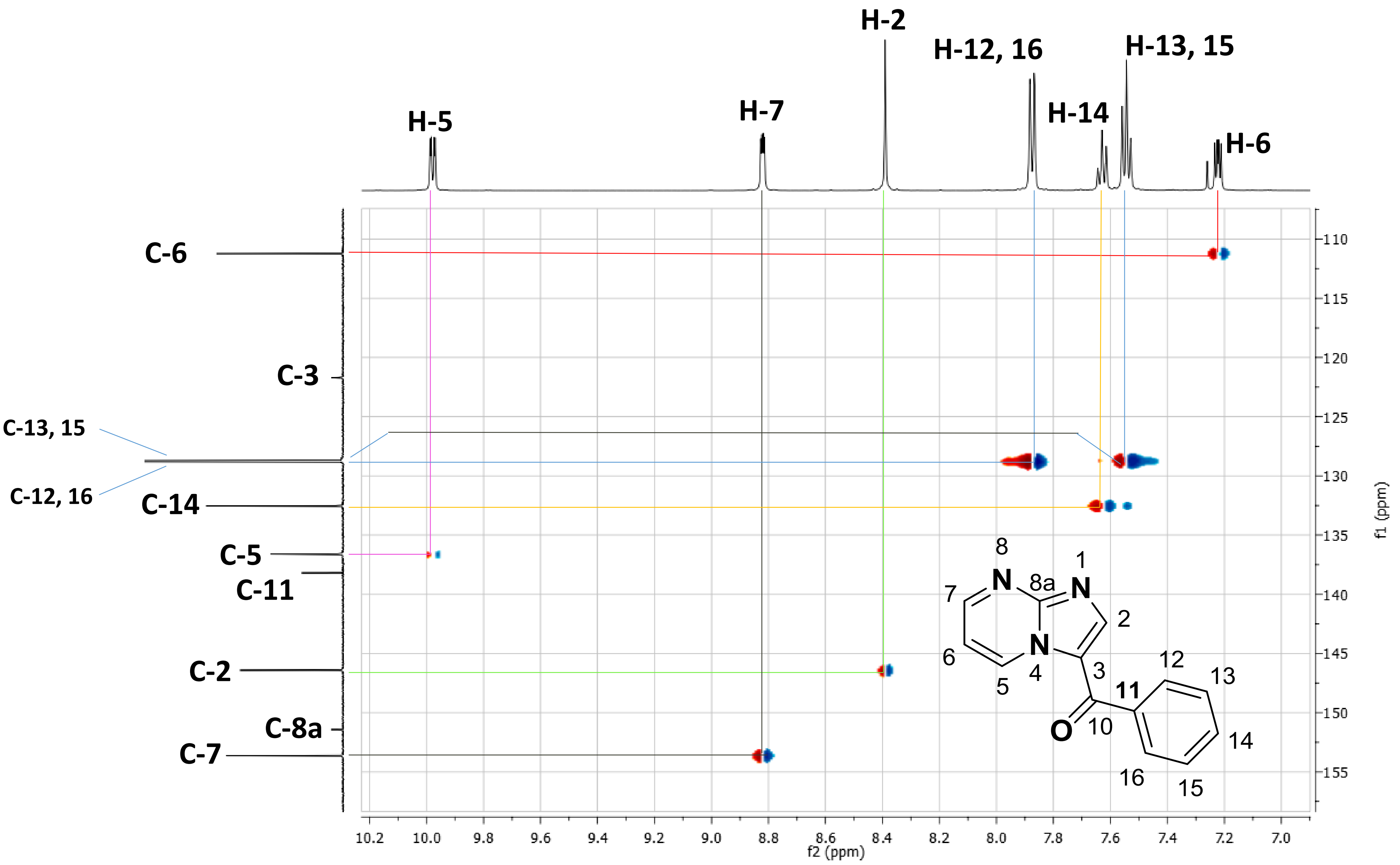

Figure S17: HSQC experiment of imidazo[1,2-*a*]pyrimidin-3-yl(phenyl)methanone (**4a**).

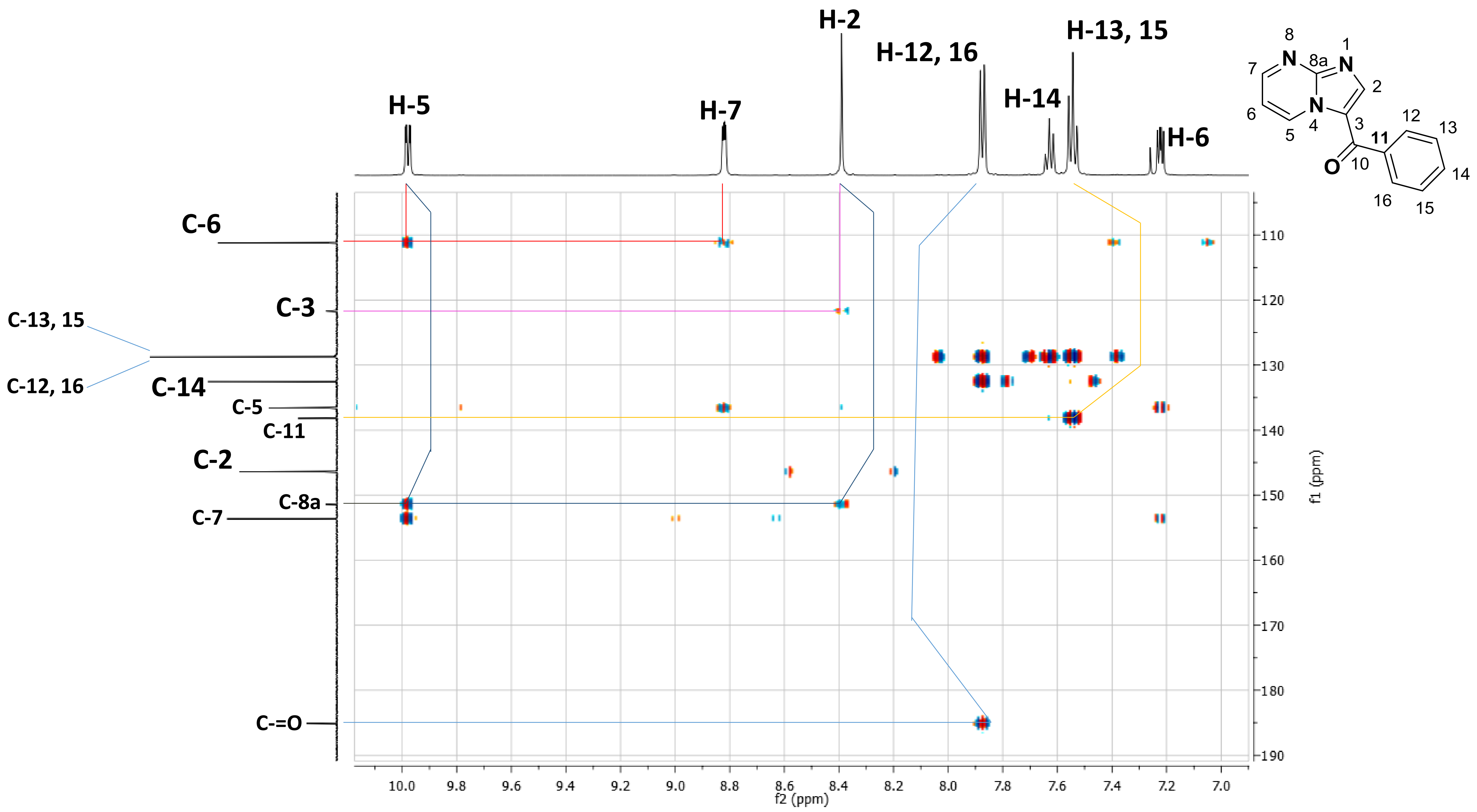

Figure S18: HMBC experiment of imidazo[1,2-*a*]pyrimidin-3-yl(phenyl)methanone (**4a**).

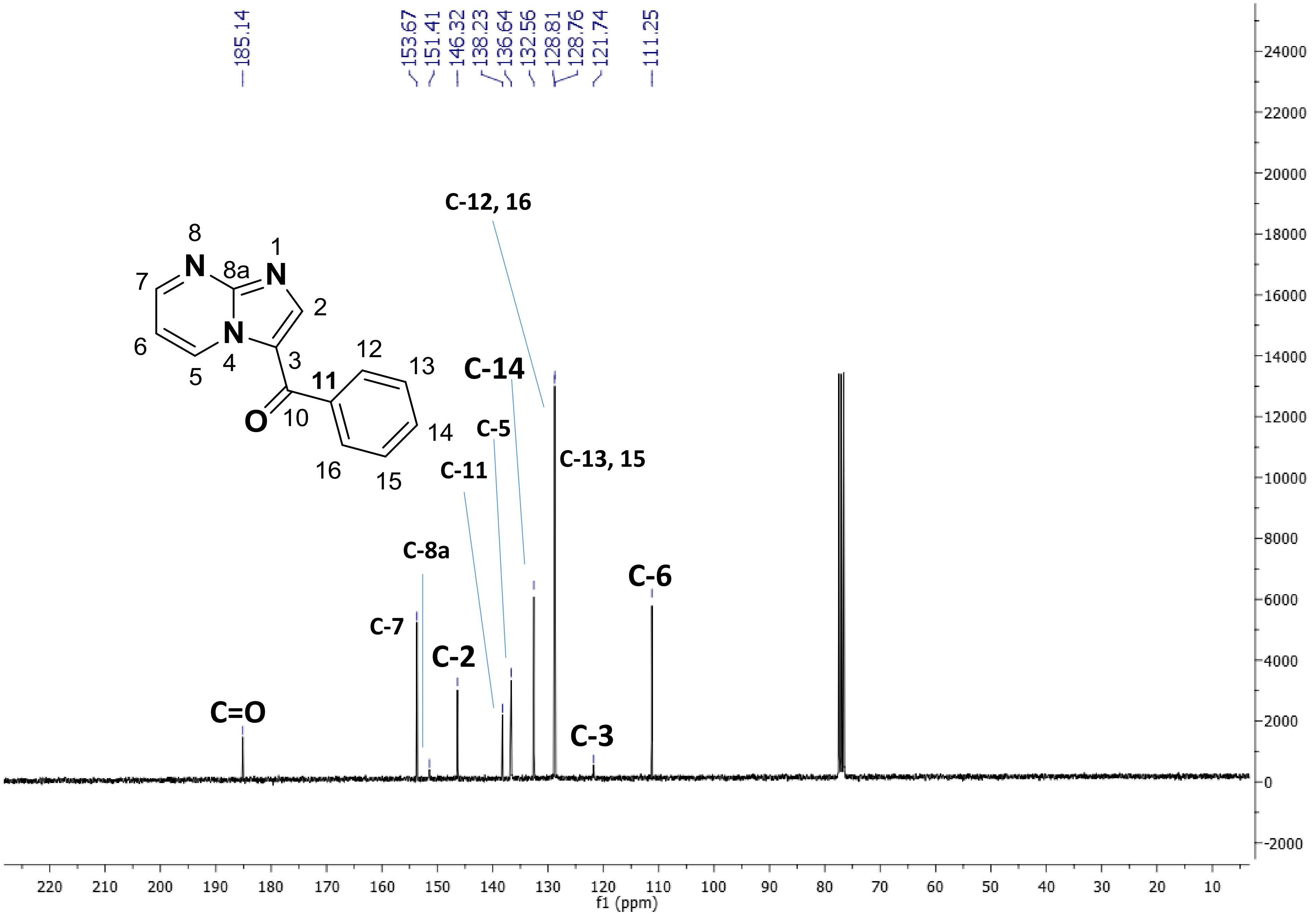

Figure S19: ^{13}C NMR (125 MHz, $CDCl_3$) of imidazo[1,2-*a*]pyrimidin-3-yl(phenyl)methanone (**4a**).

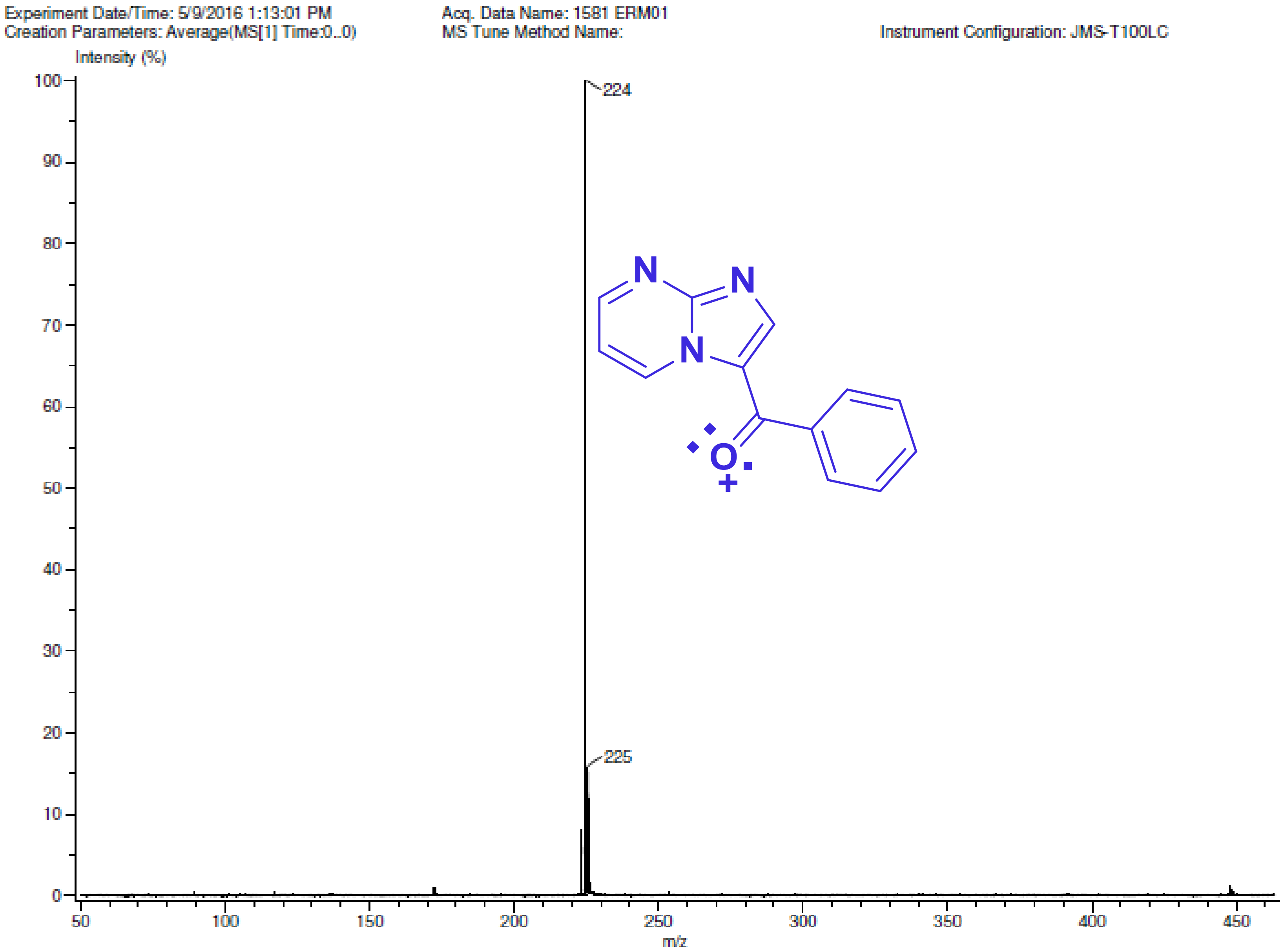

Figure S20: Mass spectrum of imidazo[1,2-*a*]pyrimidin-3-yl(phenyl)methanone (4**a**).

Instrument: JEOL GCmate
Inlet: Direct Probe Ionization mode: EI+

Scan: 222 R.T.: 2.96
Base: m/z 223; 1.6%FS TIC: 251696 #Ions: 332

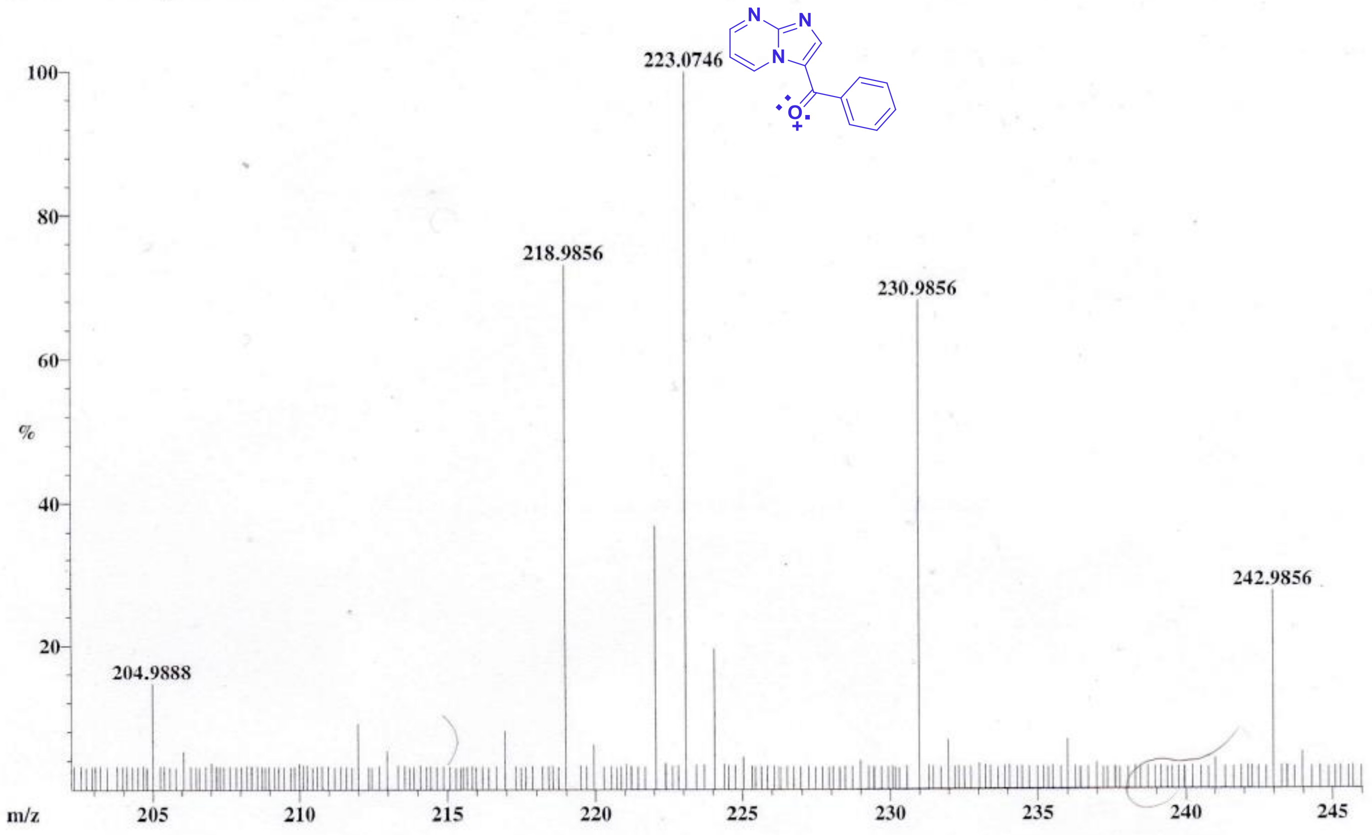

Selected Isotopes : $H_{0-9}C_{0-13}N_{0-3}O_{0-1}$ Error Limit : 5 ppm

Measured Mass	% Base	Formula	Calculated Mass	Error
223.0746	100.0%	$C_{13}H_9N_3O$	223.0746	0.2

Figure S21: HREIMS of imidazo[1,2-*a*]pyrimidin-3-yl(phenyl)methanone (**4a**).

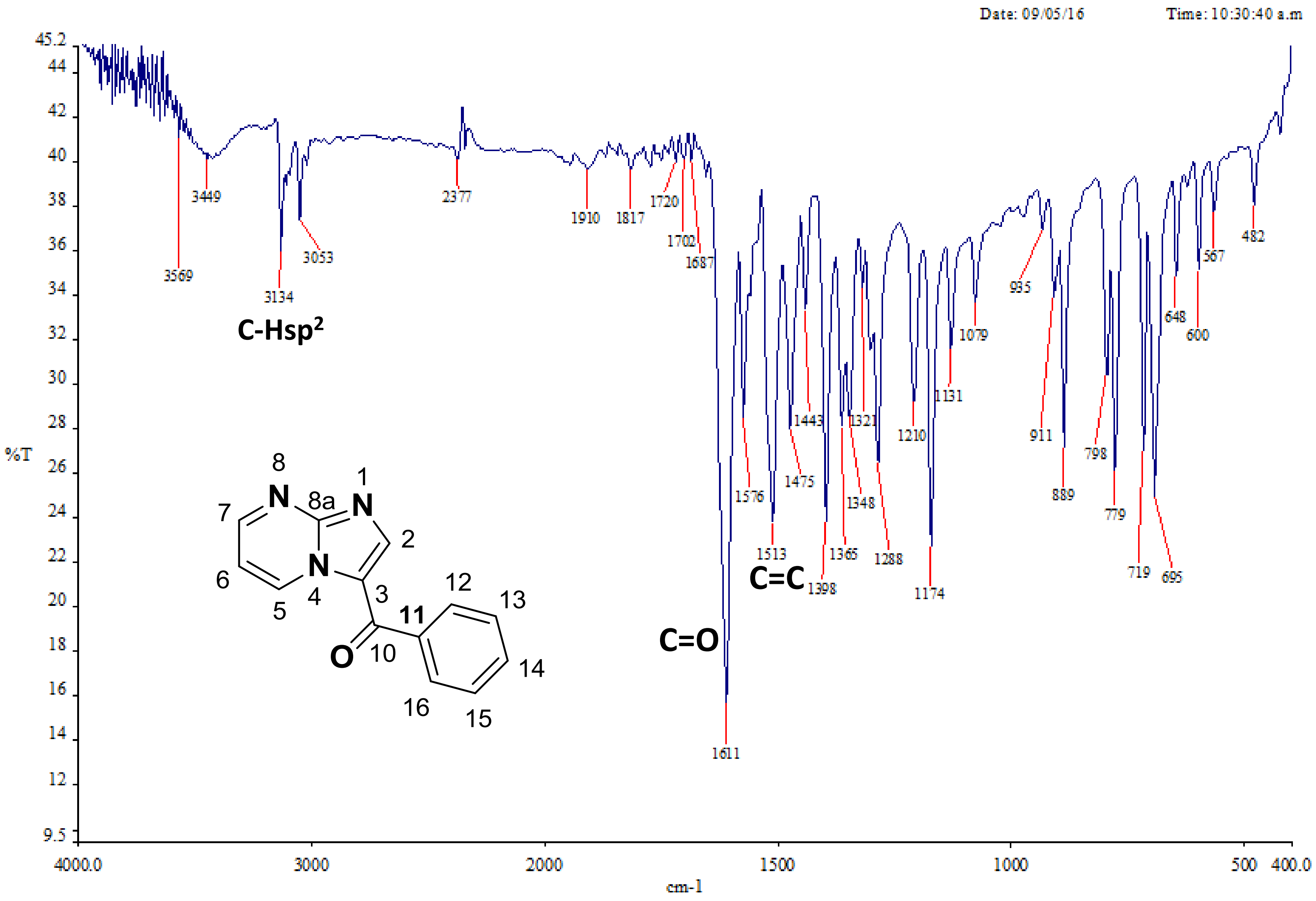

Figure S22: IR spectrum of imidazo[1,2-*a*]pyrimidin-3-yl(phenyl)methanone (**4a**).

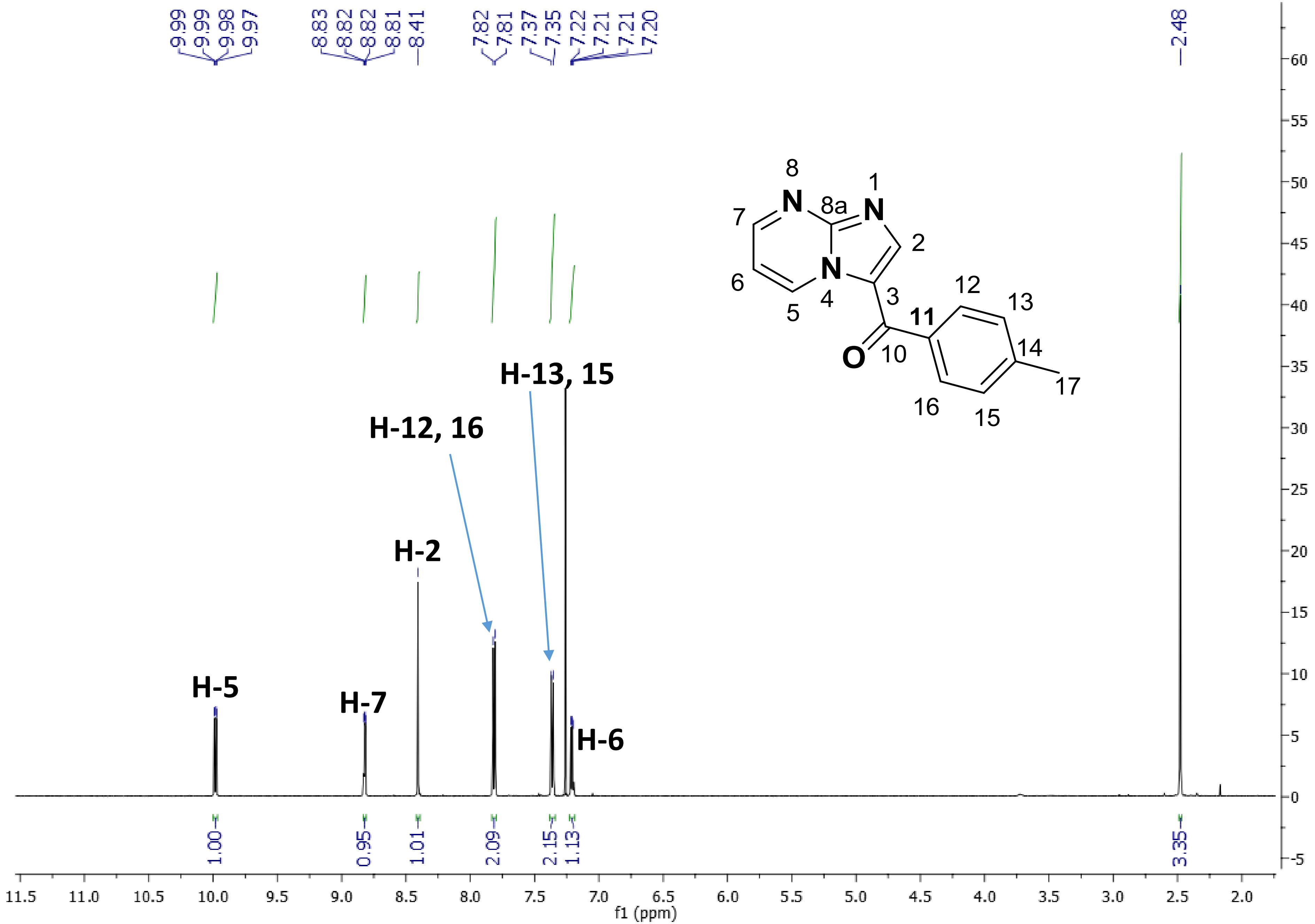

Figure S23: ^{1}H NMR (300 MHz, $CDCl_3$) of imidazo[1,2-*a*]pyrimidin-3-yl(p-tolyl)methanone (**4e**).

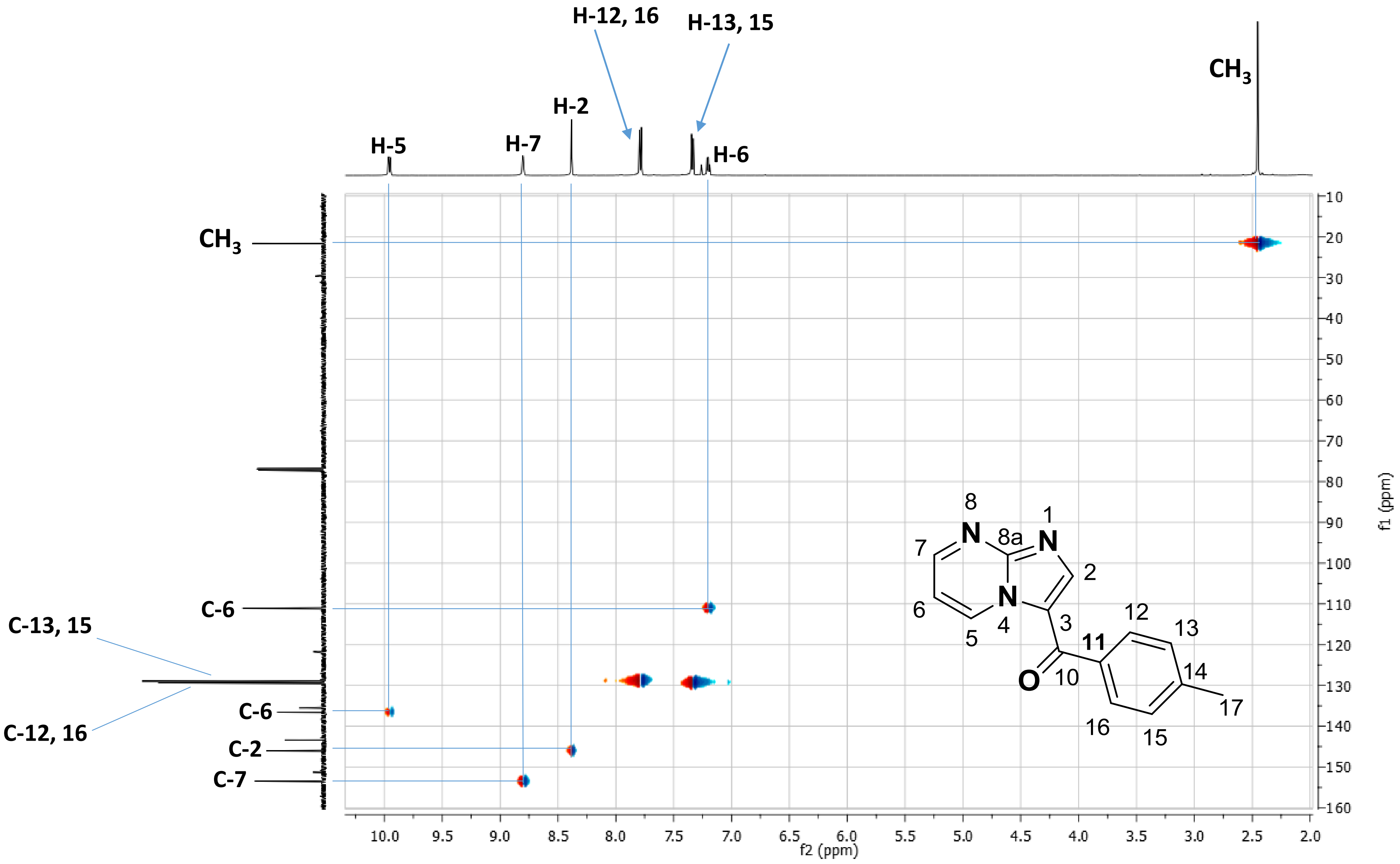

Figure S24: HSQC experiment of imidazo[1,2-*a*]pyrimidin-3-yl(p-tolyl)methanone (**4e**).

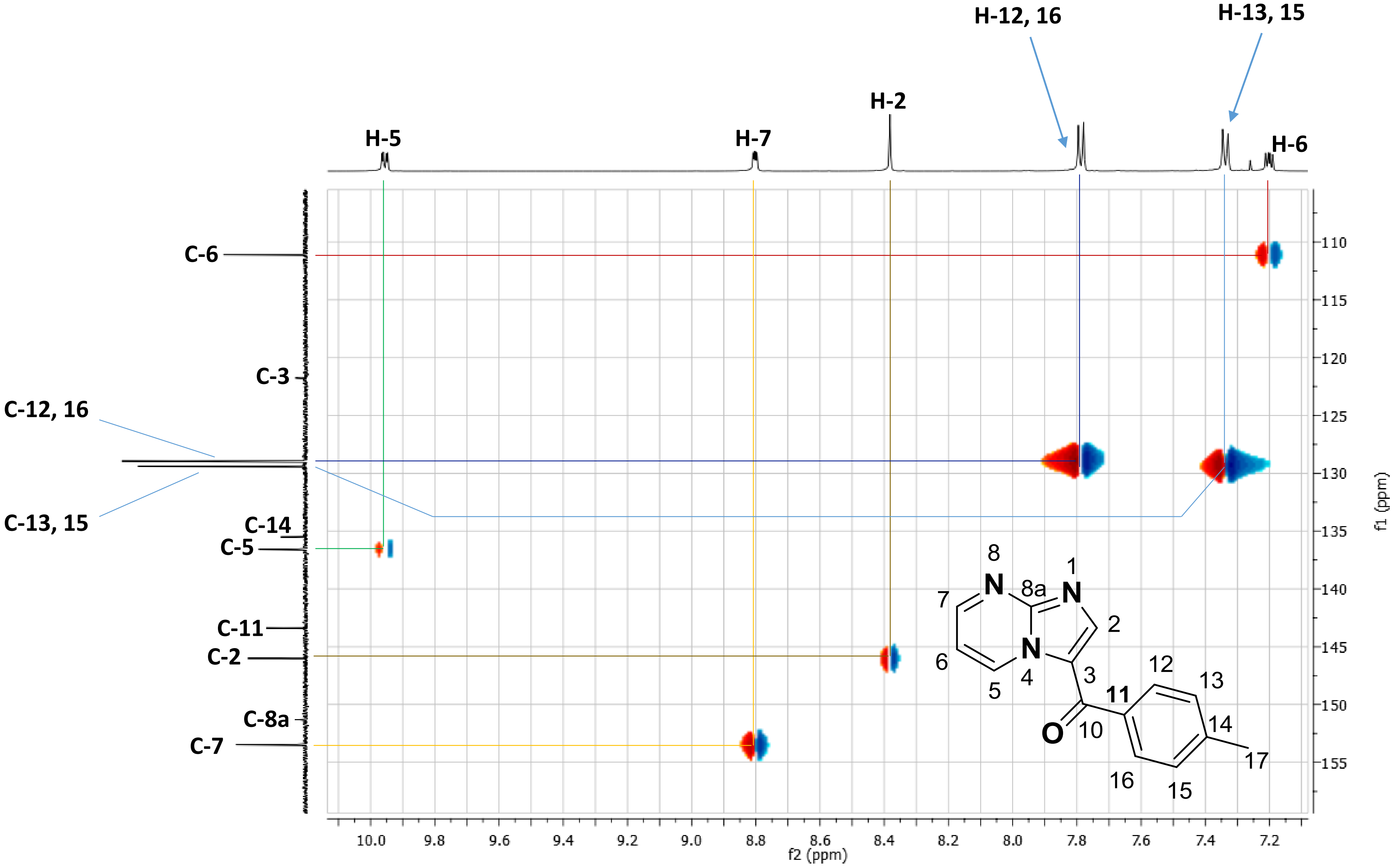

Figure S25: HSQC experiment of imidazo[1,2-*a*]pyrimidin-3-yl(p-tolyl)methanone (**4e**).

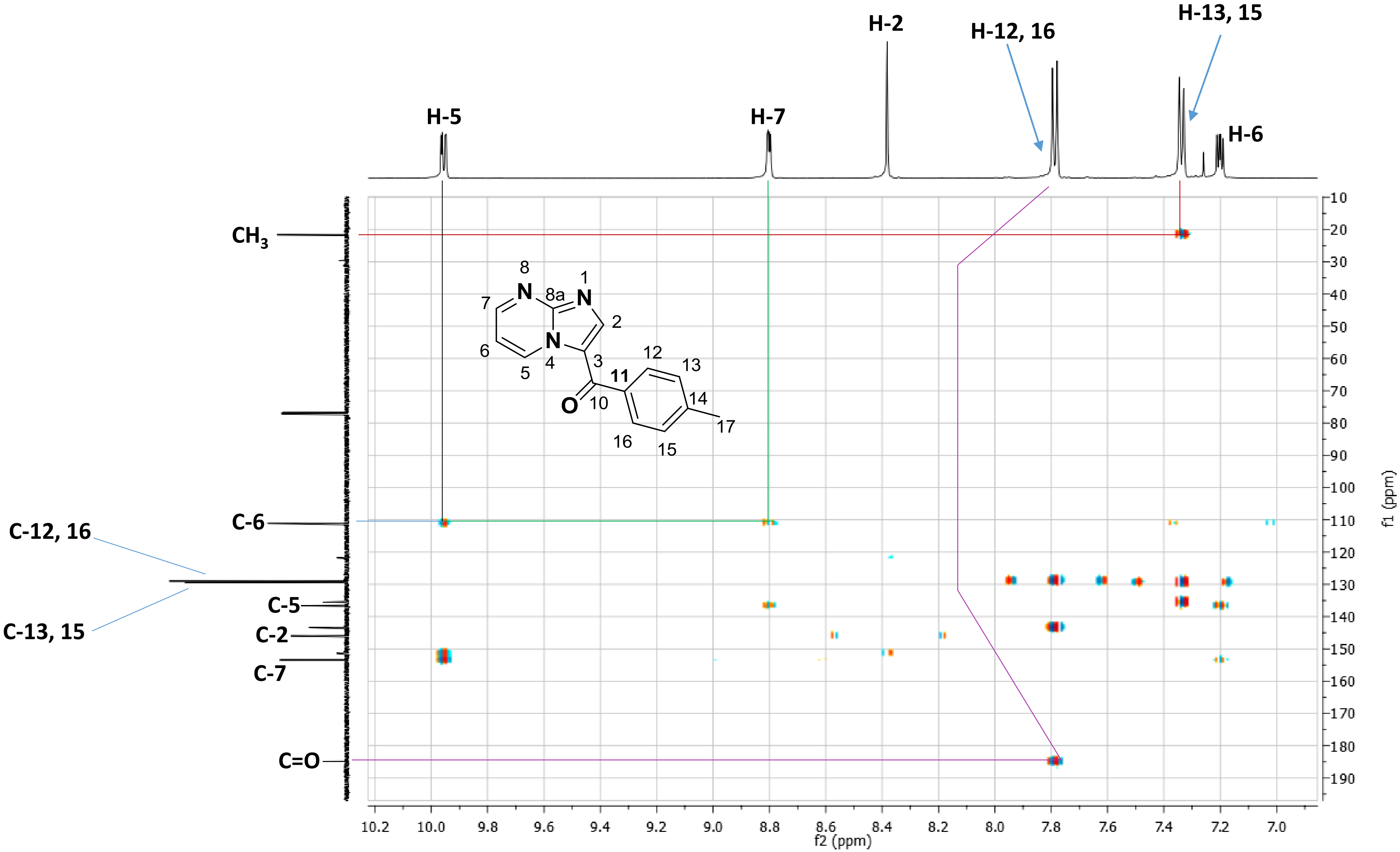

Figure S26: HMBC experiment of imidazo[1,2-*a*]pyrimidin-3-yl(p-tolyl)methanone (**4e**).

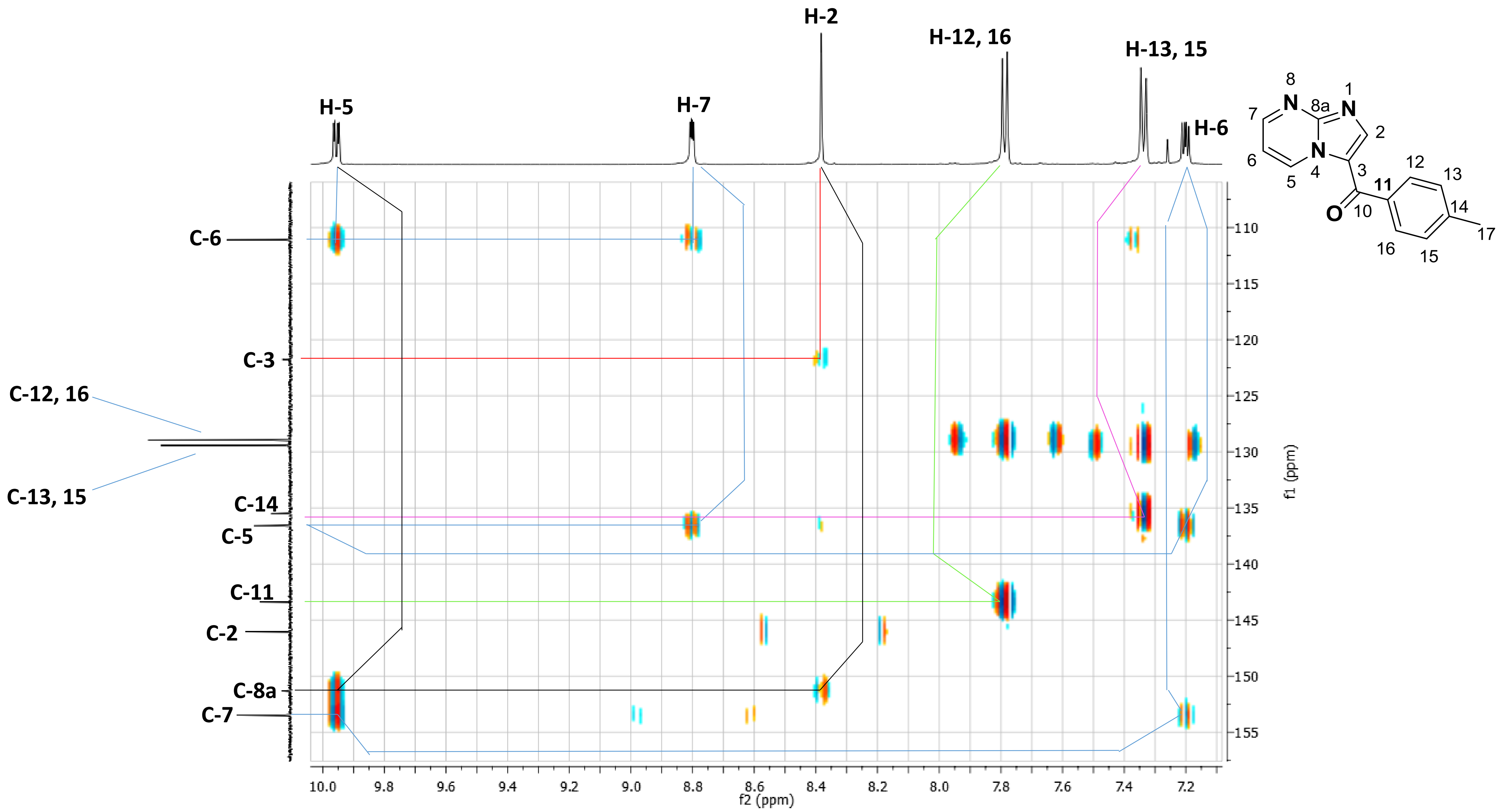

Figure S27: HMBC experiment of imidazo[1,2-*a*]pyrimidin-3-yl(p-tolyl)methanone (**4e**).

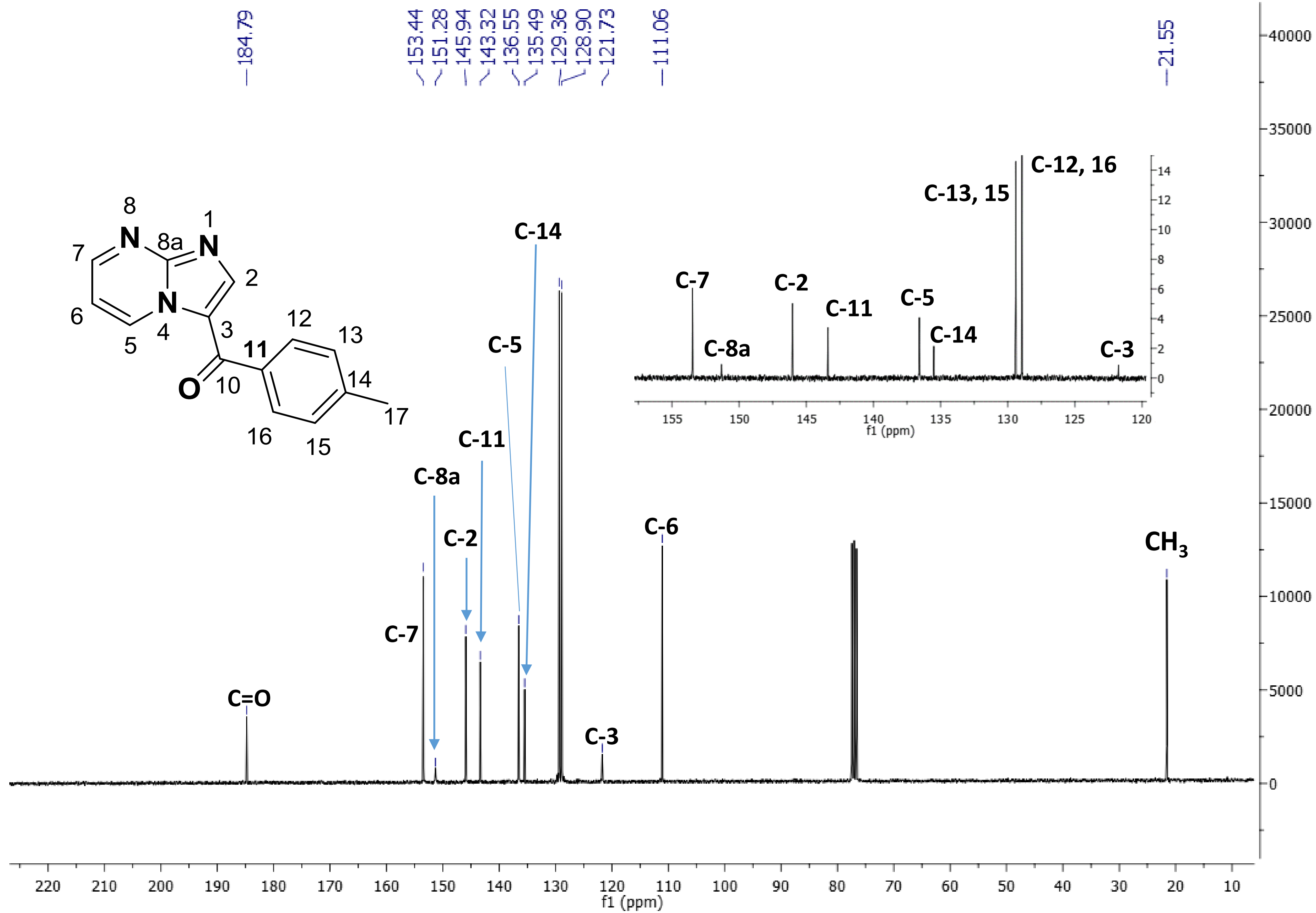

Figure S28: ^{13}C NMR (75 MHz, $CDCl_3$) of imidazo[1,2-*a*]pyrimidin-3-yl(p-tolyl)methanone (**4e**).

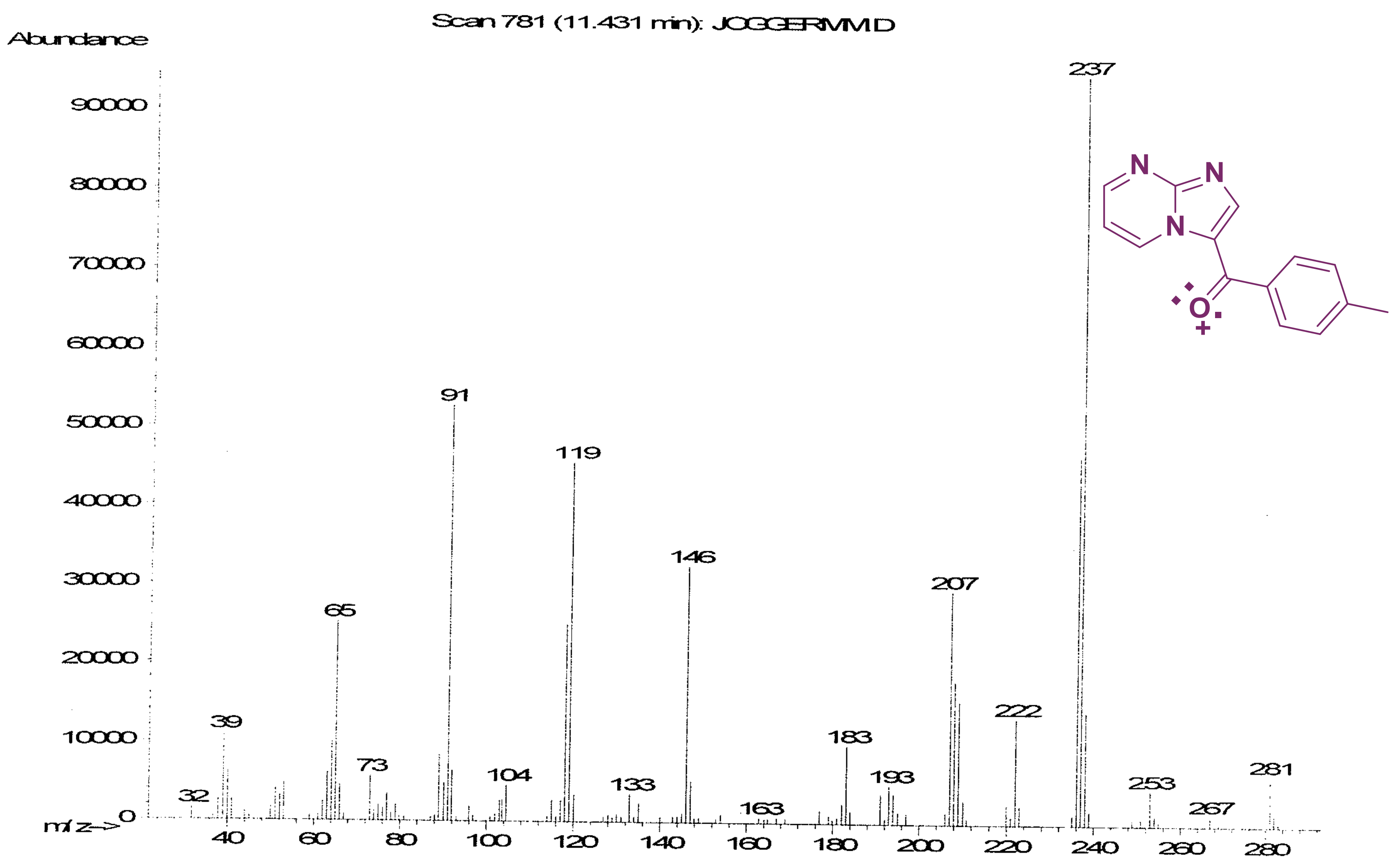

Figure S29: Mass spectrum of imidazo[1,2-*a*]pyrimidin-3-yl(p-tolyl)methanone (**4e**).

Instrument: JEOL GCmate
Inlet: Direct Probe

Ionization mode: EI+

Scan: 234
Base: m/z 237; 4.8%FS TIC: 320240

R.T.: 3.13

#Ions: 329

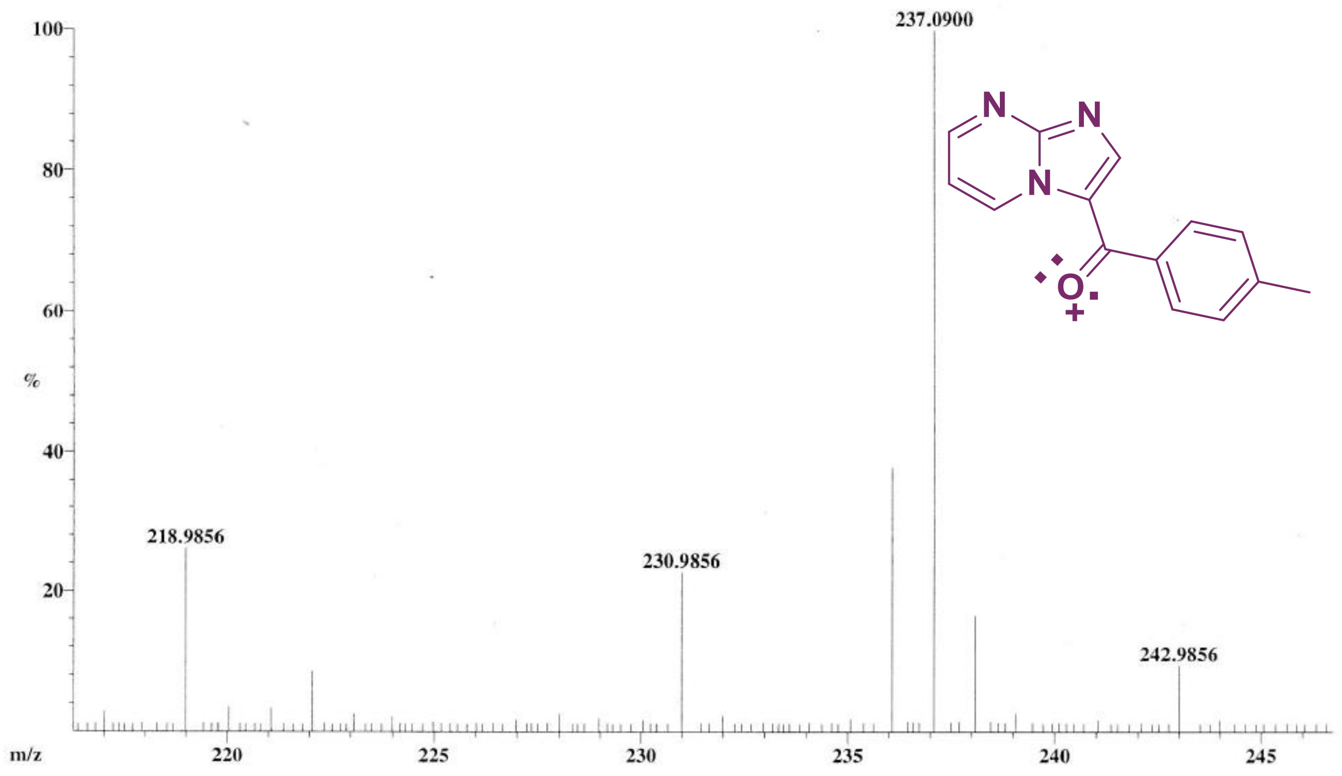

Selected Isotopes : $H_{0-11}C_{0-14}N_{0-3}O_{0-1}$

Error Limit : 5 ppm

Measured Mass	% Base	Formula	Calculated Mass	Error
237.0900	100.0%	$C_{14}H_{11}N_{3}O$	237.0902	-0.9

Figure S30: EREIMS of imidazo[1,2-*a*]pyrimidin-3-yl(p-tolyl)methanone (**4e**).

Central de Instrumentación de Espectroscopía ENCB-IPN

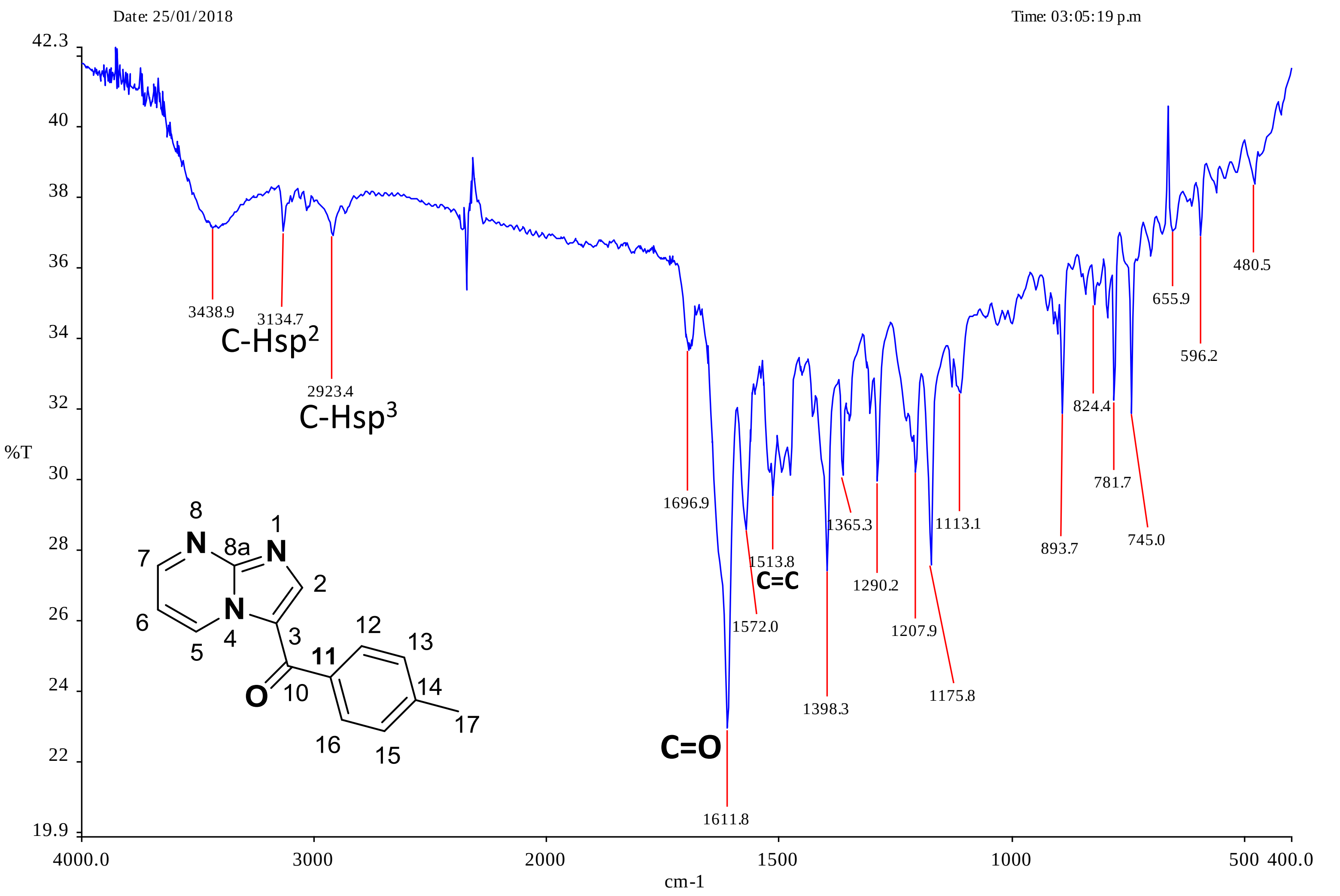

Spectrum Pathname: C:\pel_data\results\USUARIOS\Ma. Elena Campos\Laura Segura\25-enero-18\ERM-Me.001

Description: Pastilla

Instrument Model: Spectrum 2000, Perkin Elmer

Figure S31: IR spectrum of imidazo[1,2-*a*]pyrimidin-3-yl(p-tolyl)methanone (**4e**).

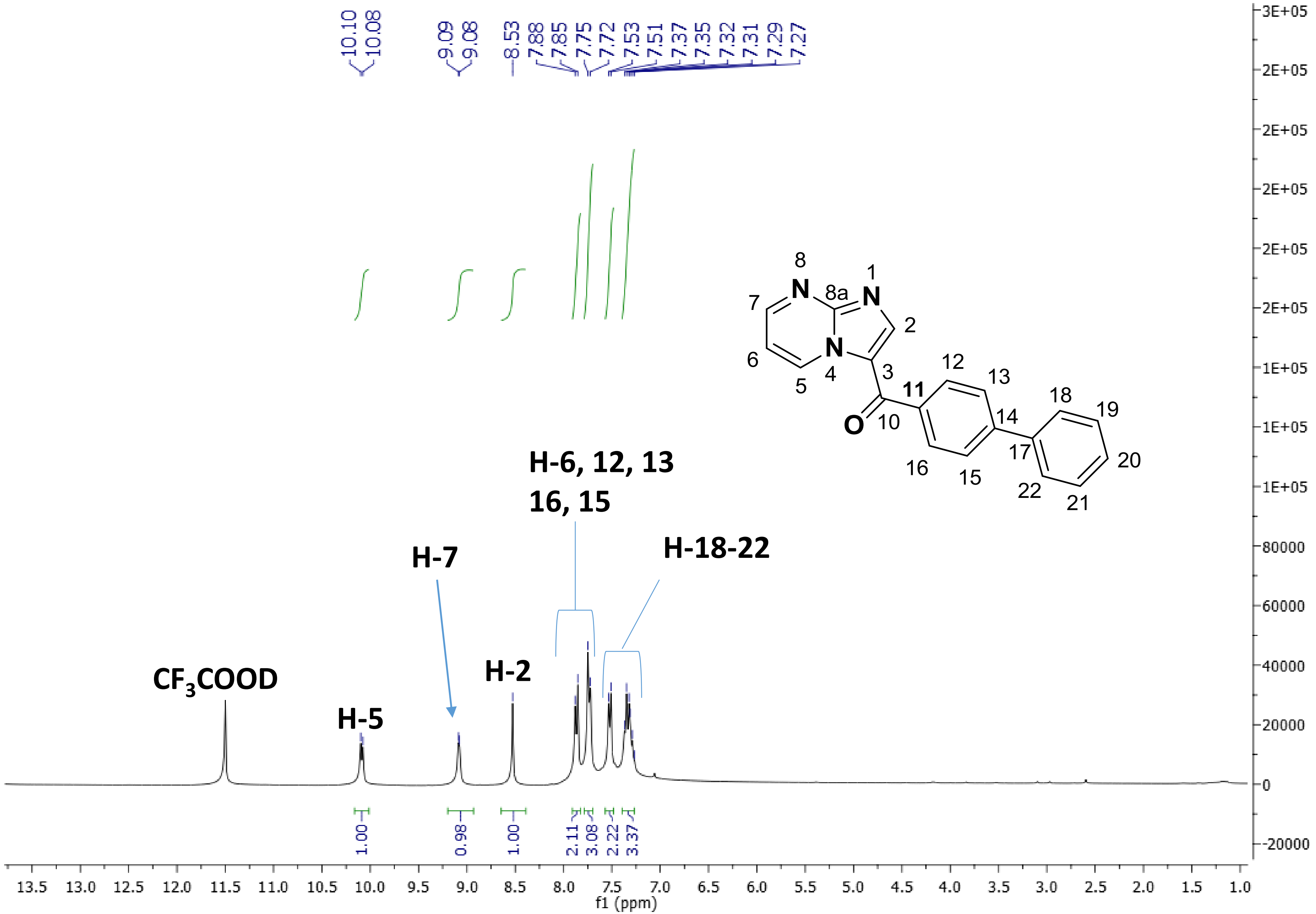

Figure S32: ^{1}H NMR (300 MHz, CF_3COOD) of [1,1'-biphenyl]-4-yl(imidazo[1,2-*a*]pyrimidin-3-yl)methanone (**4d**).

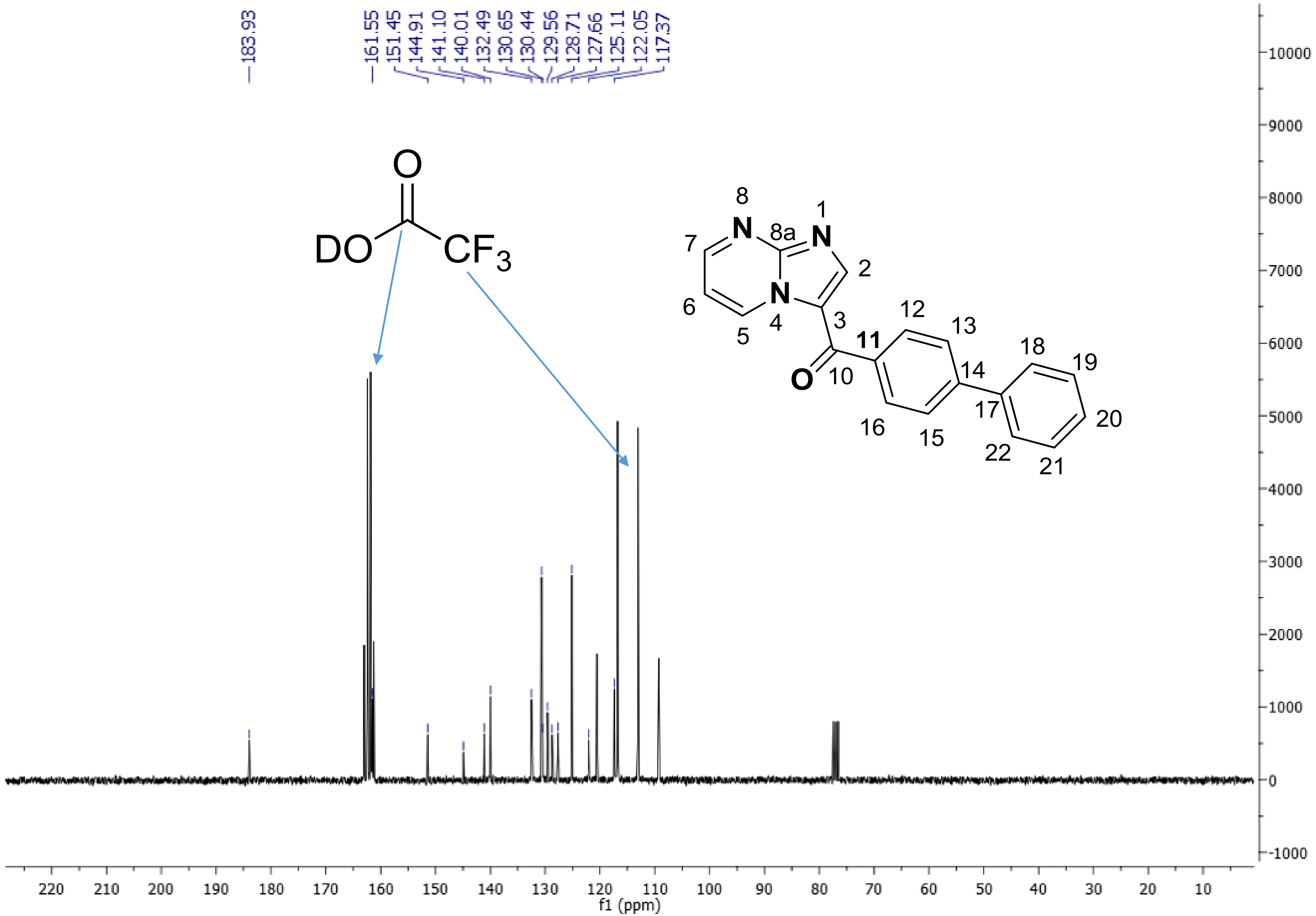

Figure S33: ^{13}C NMR (75 MHz, CF_3COOD) of [1,1'-biphenyl]-4-yl(imidazo[1,2-*a*]pyrimidin-3-yl)methanone (**4d**).

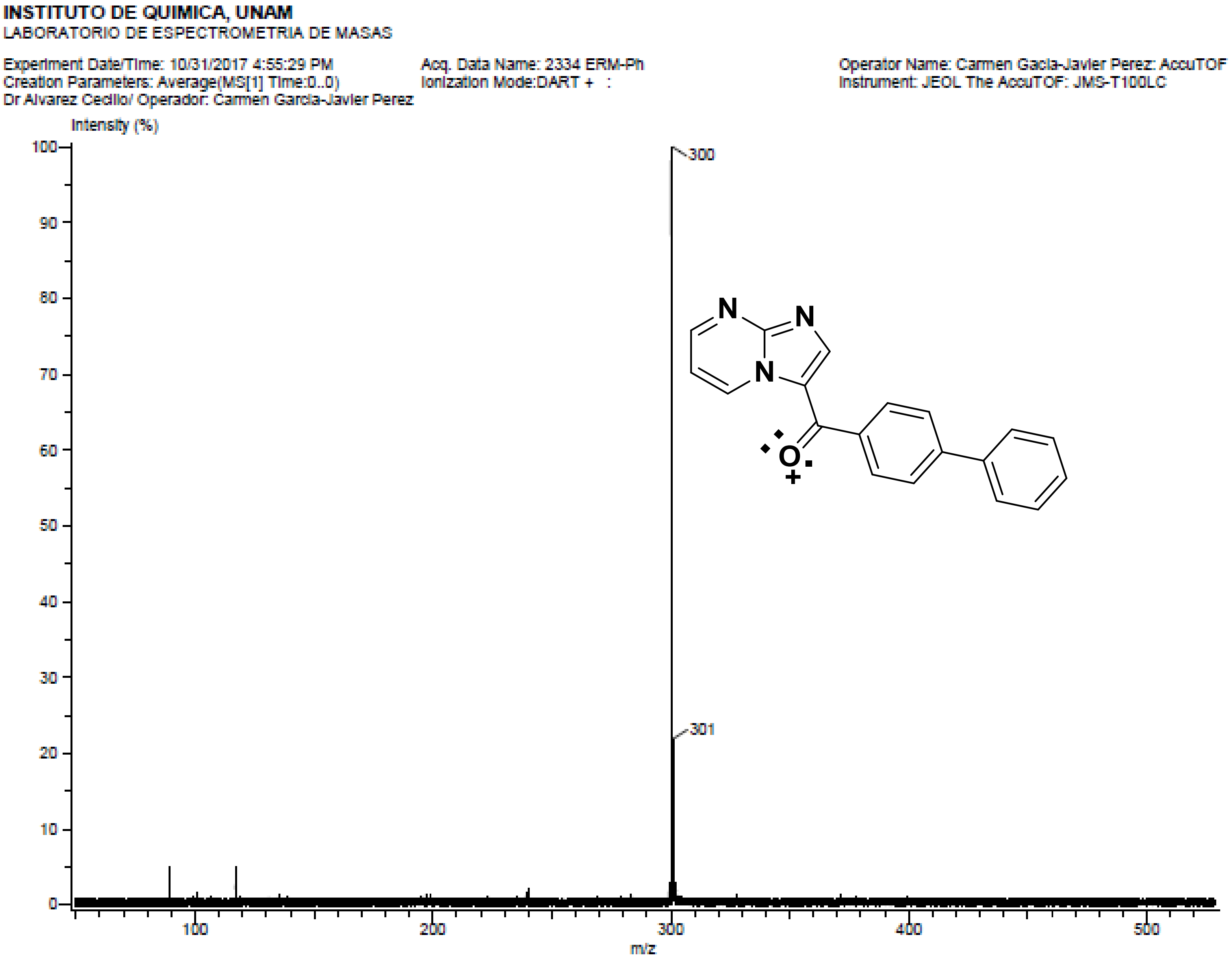

Figure S34: Mass spectrum of [1,1'-biphenyl]-4-yl(imidazo[1,2-*a*]pyrimidin-3-yl)methanone (**4d**).

Data:2334 ERM-Ph
Sample Name:Dr Alvarez Cecilio/ Operador: Carmen Garcia-Javier Perez
Description:
Ionization Mode:ESI+
History:Determine m/z[Peak Detect[Centroid,30,Area];Correct Base[10.0%]];Correct Base[5.0%];Average(MS[1] 0..1)

Acquired:10/31/2017 4:55:29 PM
Operator:AccuTOF
Mass Calibration data:Cal Peg 600
Created:11/15/2017 12:48:43 PM
Created by:

Charge number:1
Element:^{12}C:0 .. 20, ^{1}H:0 .. 40, ^{14}N:0 .. 4, ^{16}O:0 .. 2

Tolerance:5.00(mmu)

Unsaturation Number:0.0 .. 50.0 (Fraction:Both)

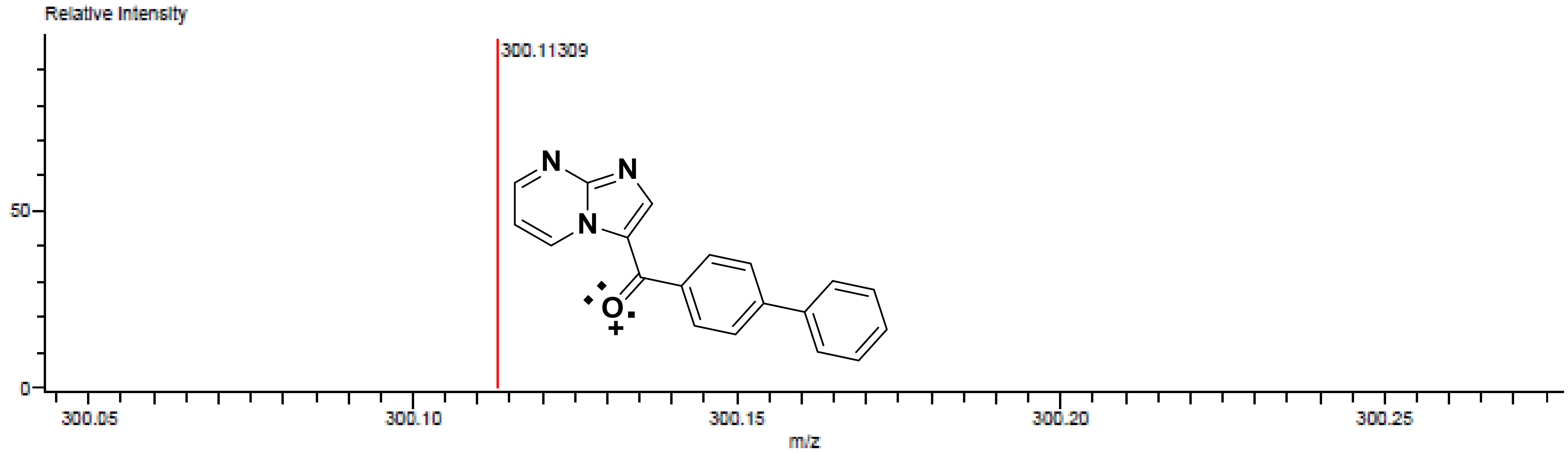

Mass	Intensity	Calc. Mass	Mass Difference (mmu)	Mass Difference (ppm)	Possible Formula	Unsaturation Number
300.11309	21085.85	300.11369	-0.59	-1.98	$^{12}C_{19}{}^{1}H_{14}{}^{14}N_{3}{}^{16}O_{1}$	14.5

Figure S35: HRMS of [1,1'-biphenyl]-4-yl(imidazo[1,2-*a*]pyrimidin-3-yl)methanone (**4d**).

Central de Instrumentación de Espectroscopía ENCB-IPN

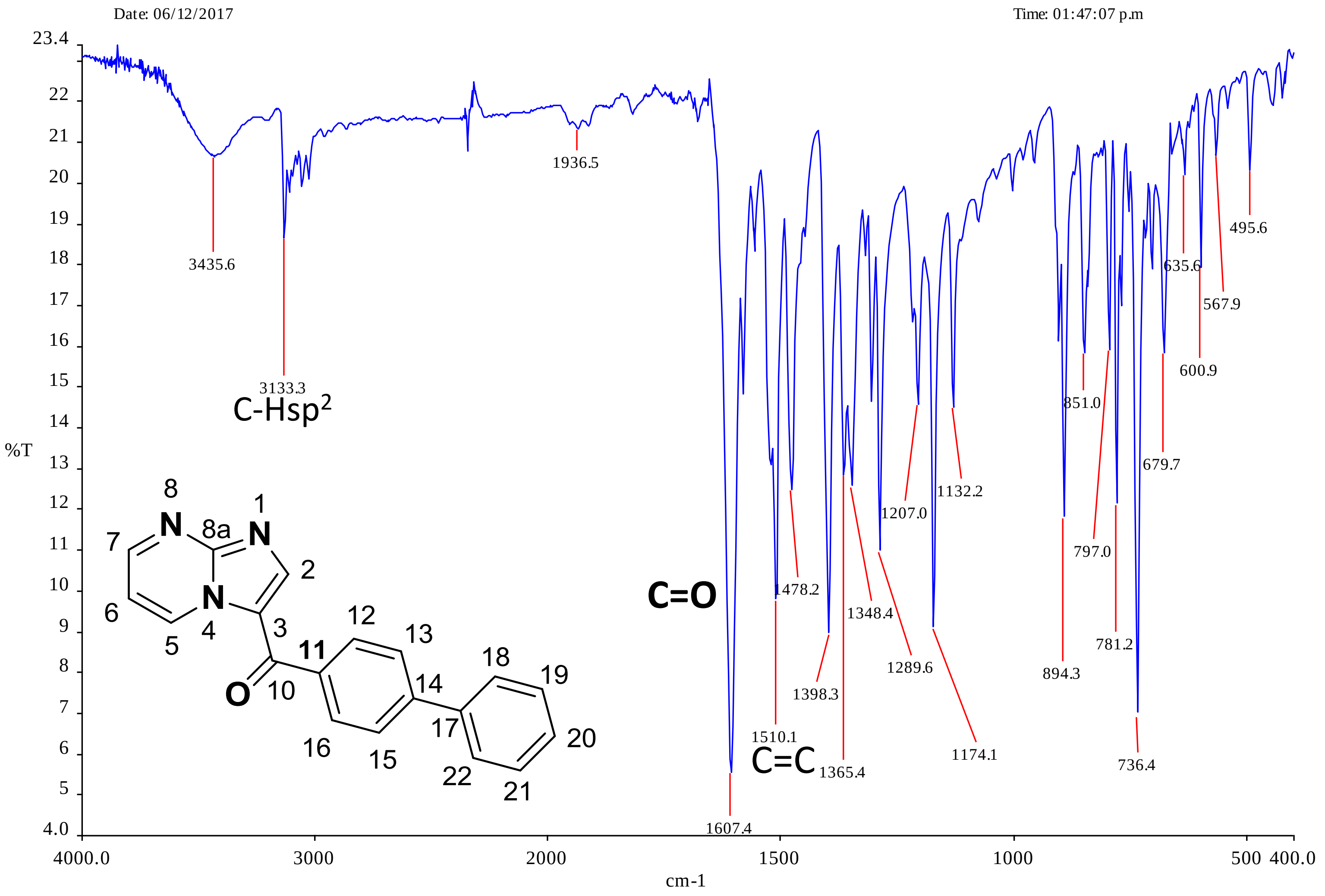

Spectrum Pathname: C:\pel_data\results\USUARIOS\Ma. Elena Campos\Laura Segura\6-dic-17\ERM-PH.001

Description: Pastilla

Instrument Model: Spectrum 2000, Perkin Elmer

Figure S36: IR spectrum of [1,1'-biphenyl]-4-yl(imidazo[1,2-*a*]pyrimidin-3-yl)methanone (**4d**).

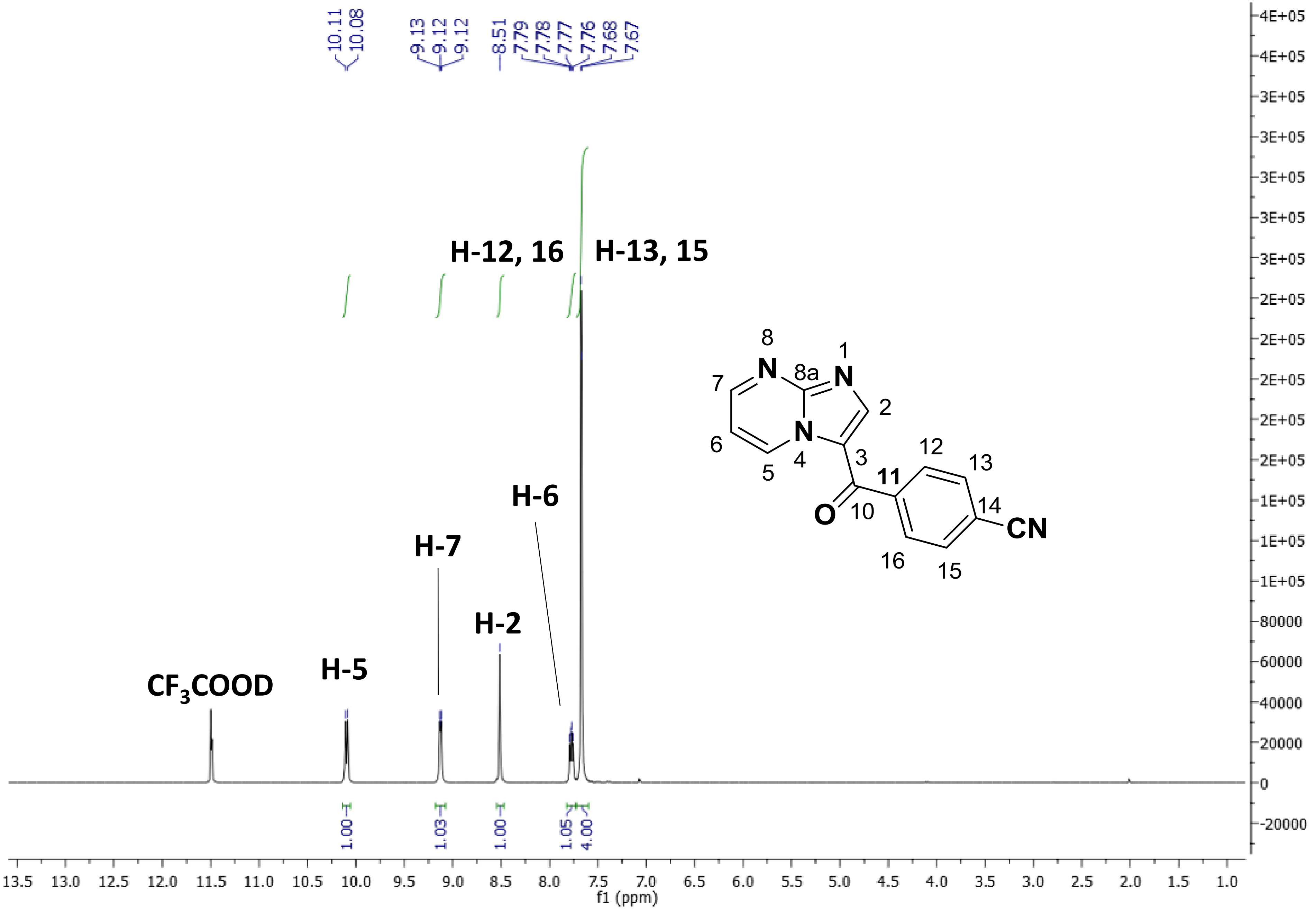

Figure S37: ^{1}H NMR (300 MHz, CF_3COOD) of 4-(imidazo[1,2-*a*]pyrimidine-3-carbonyl)benzonitrile (**4f**).

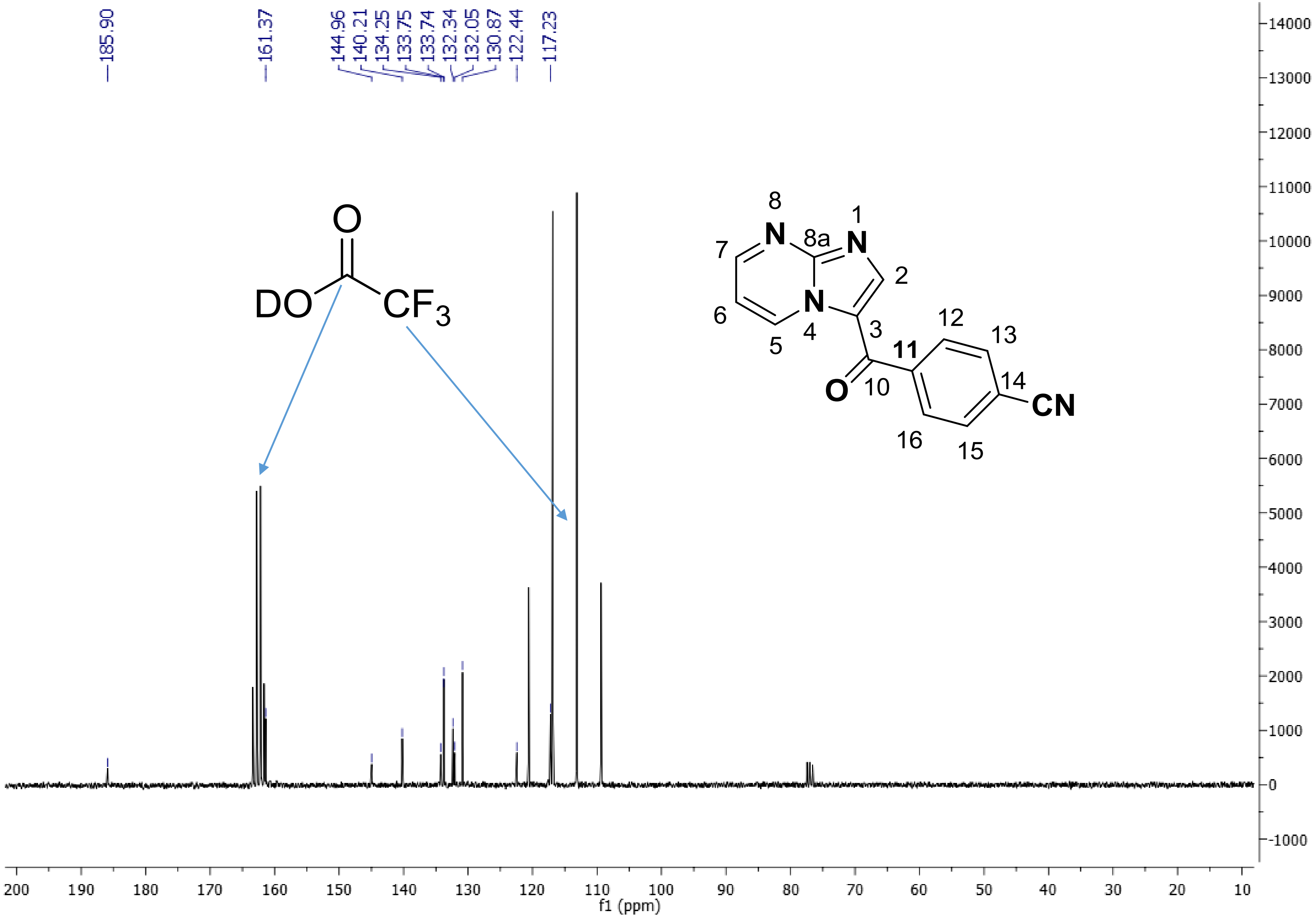

Figure S38: ^{13}C NMR (75 MHz, CF_3COOD) of 4-(imidazo[1,2-*a*]pyrimidine-3-carbonyl)benzonitrile (**4f**).

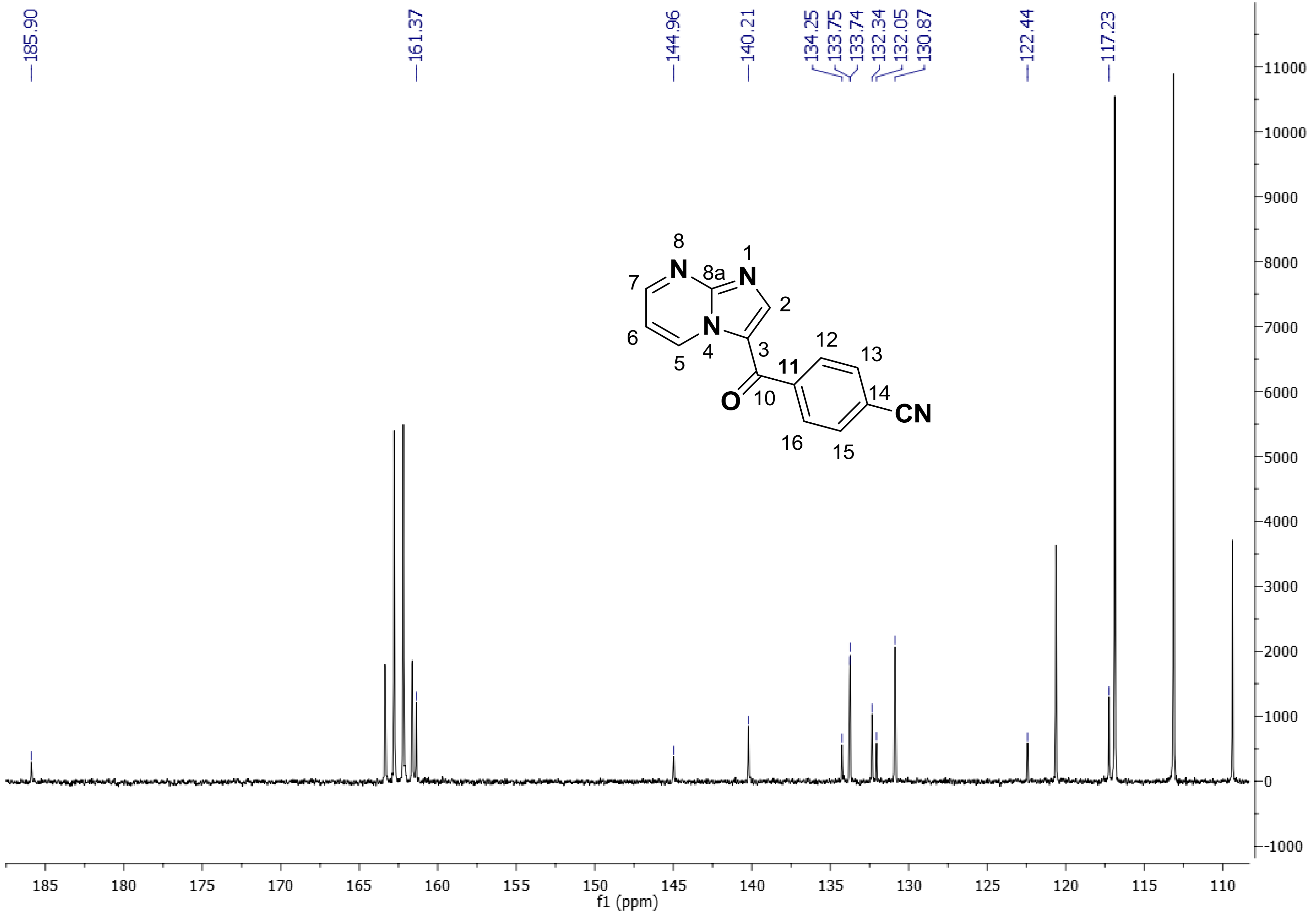

Figure S39: ^{13}C NMR (75 MHz, CF_3COOD) of 4-(imidazo[1,2-*a*]pyrimidine-3-carbonyl)benzonitrile (**4f**).

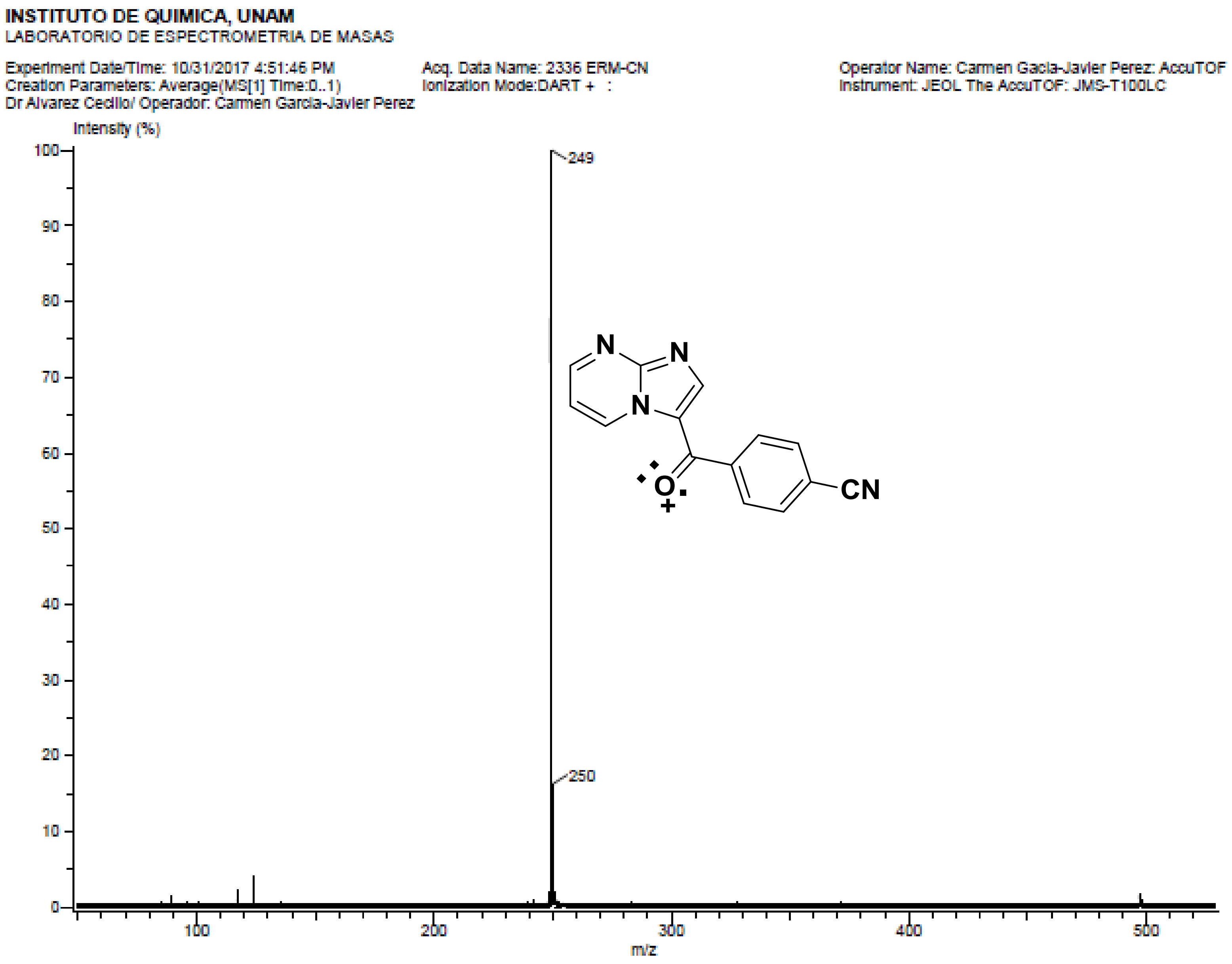

Figure S40: Mass spectrum of 4-(imidazo[1,2-*a*]pyrimidine-3-carbonyl)benzonitrile (**4f**).

Data:2336 ERM-CN
Sample Name:Dr Alvarez Cecilio/ Operador: Carmen Garcia-Javier Perez
Description:
Ionization Mode:ESI+
History:Determine m/z[Peak Detect[Centroid,30,Area];Correct Base[10.0%]];Correct Base[5.0%];Average(MS[1] 0..0)

Acquired:10/31/2017 4:51:46 PM
Operator:AccuTOF
Mass Calibration data:Cal Peg 600
Created:11/15/2017 12:55:17 PM
Created by:

Charge number:1
Tolerance:5.00(mmu)
Unsaturation Number:0.0 .. 50.0 (Fraction:Both)
Element:^{12}C:0 .. 15, ^{1}H:0 .. 40, ^{14}N:0 .. 4, ^{16}O:0 .. 3

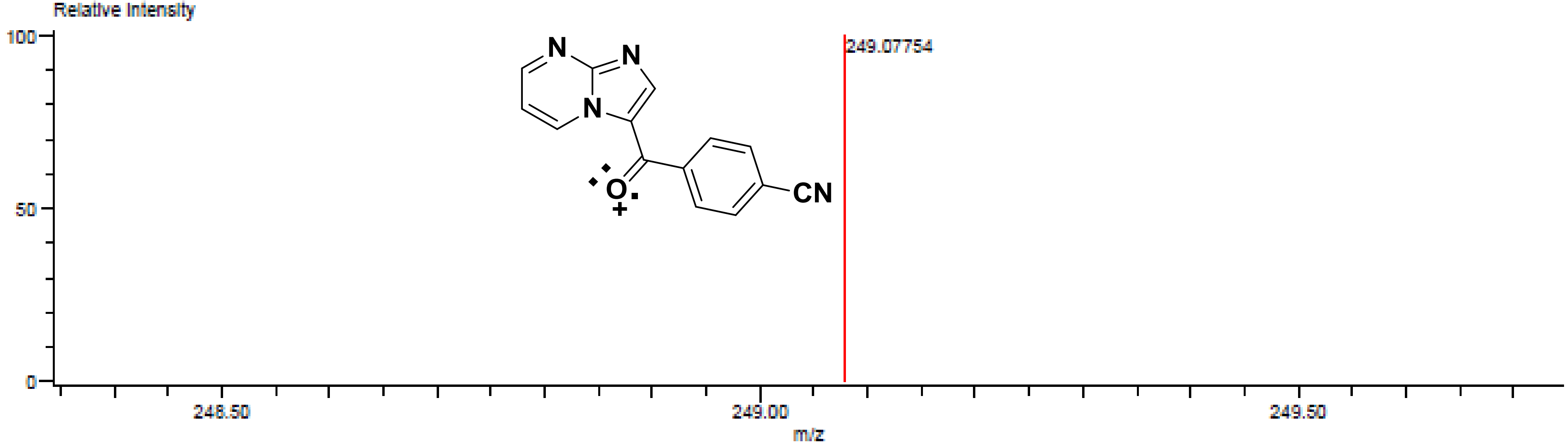

Mass	Intensity	Calc. Mass	Mass Difference (mmu)	Mass Difference (ppm)	Possible Formula	Unsaturation Number
249.07754	428846.42	249.07764	-0.10	-0.38	$^{12}C_{14}{}^{1}H_{9}{}^{14}N_{4}{}^{16}O_{1}$	12.5

Figure S41: HRMS of 4-(imidazo[1,2-*a*]pyrimidine-3-carbonyl)benzonitrile (**4f**).

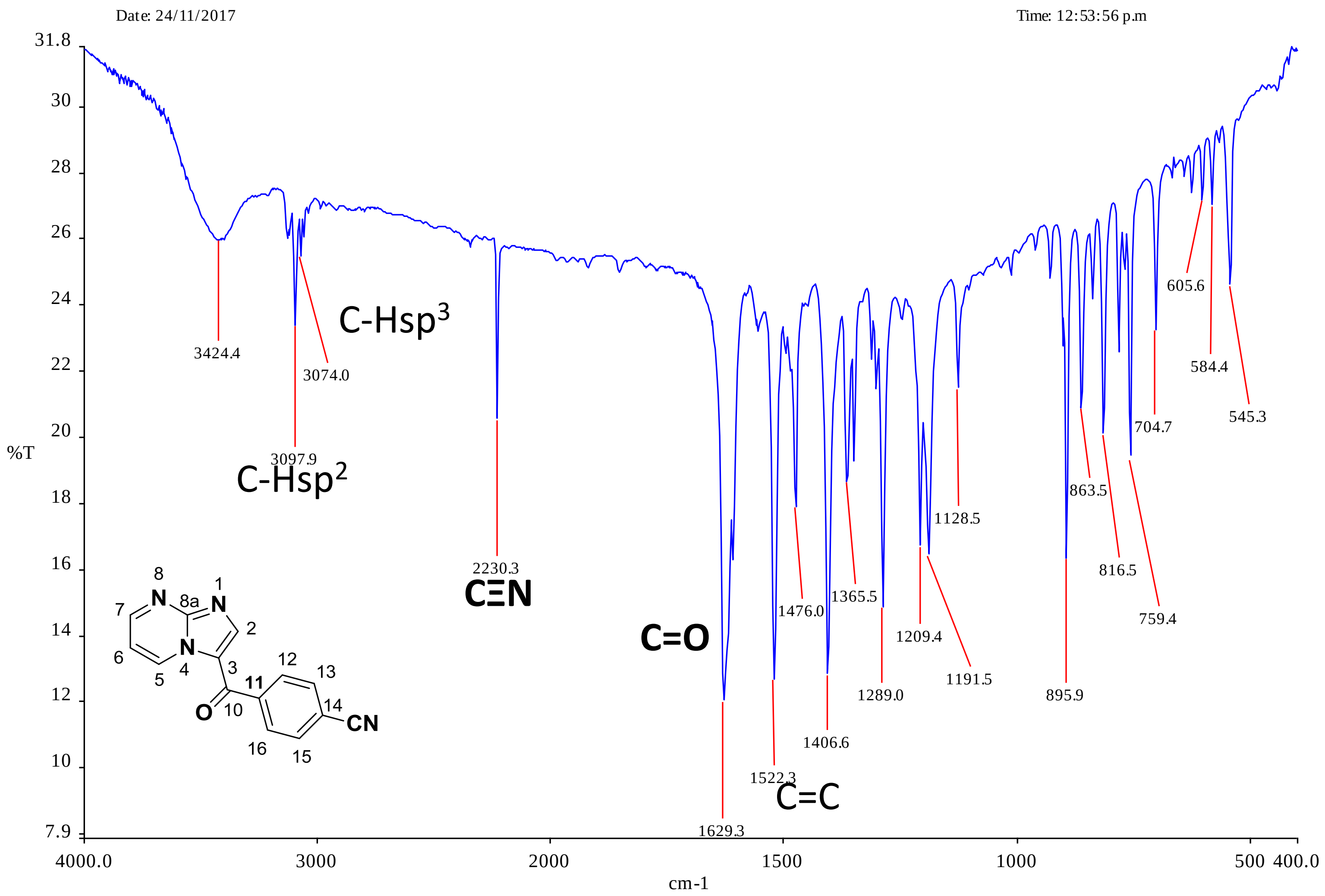

Spectrum Pathname: C:\pel_data\results\USUARIOS\Ma. Elena Campos\Laura Segura\24-nov-17\D6-CN.001

Description: Pastilla

Instrument Model: Spectrum 2000, Perkin Elmer

Figure S42: Mass spectrum of 4-(imidazo[1,2-*a*]pyrimidine-3-carbonyl)benzonitrile (**4f**).

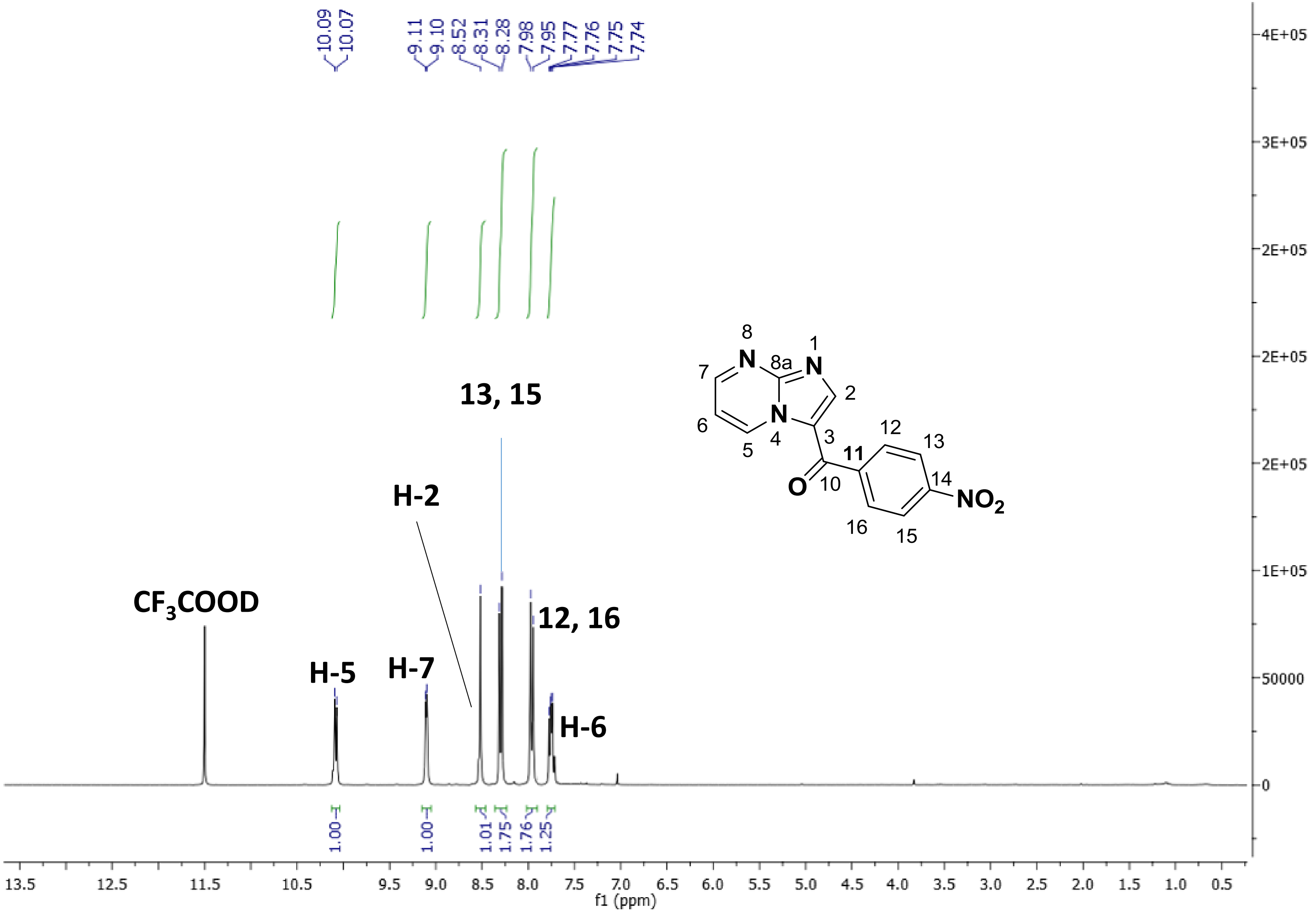

Figure S43: ^{1}H NMR (300 MHz, CF_3COOD) of imidazo[1,2-*a*]pyrimidin-3-yl(4-nitrophenyl)methanone (**4g**).

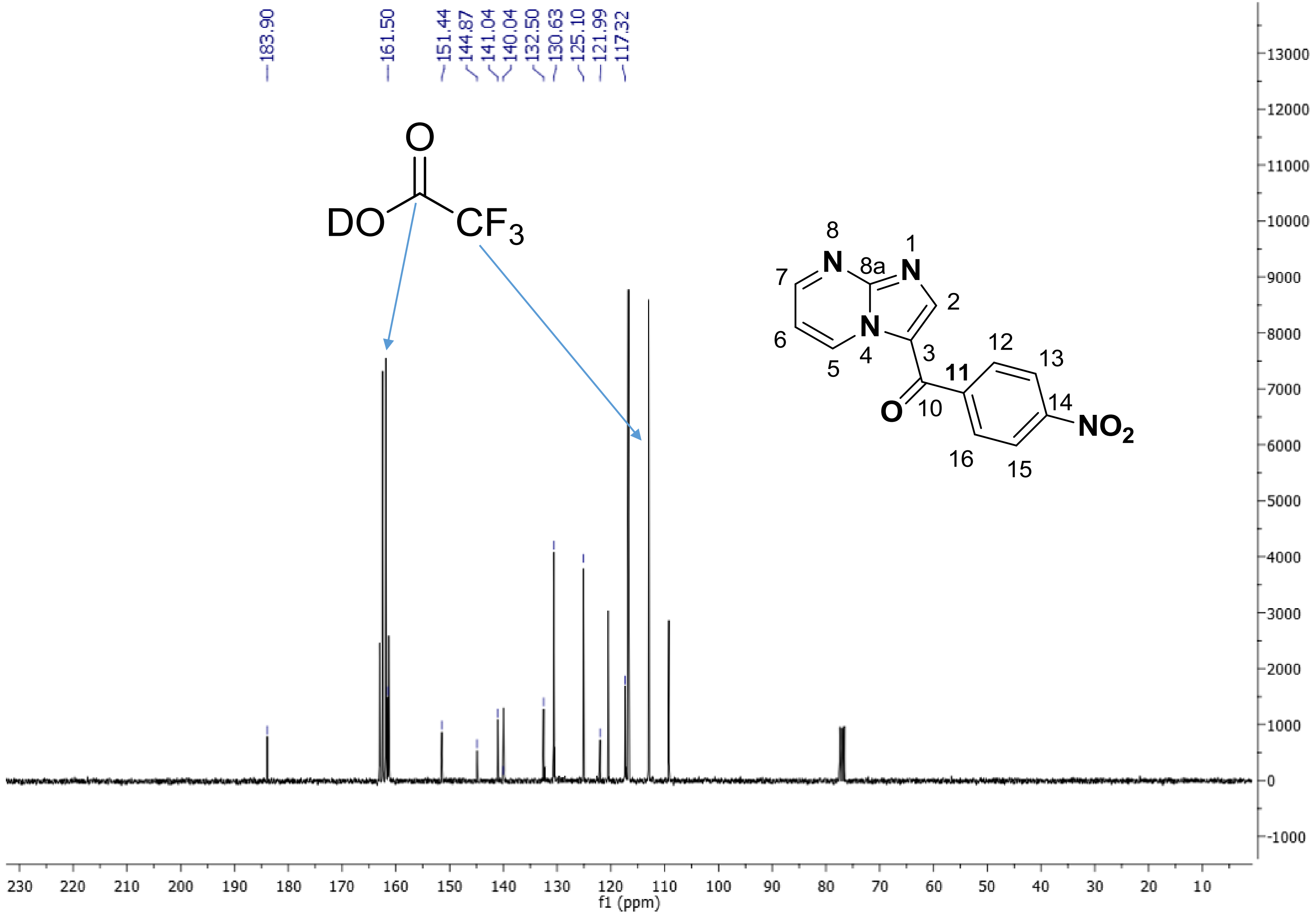

Figure S44: ^{13}C NMR (75 MHz, CF_3COOD) of imidazo[1,2-*a*]pyrimidin-3-yl(4-nitrophenyl)methanone (**4g**).

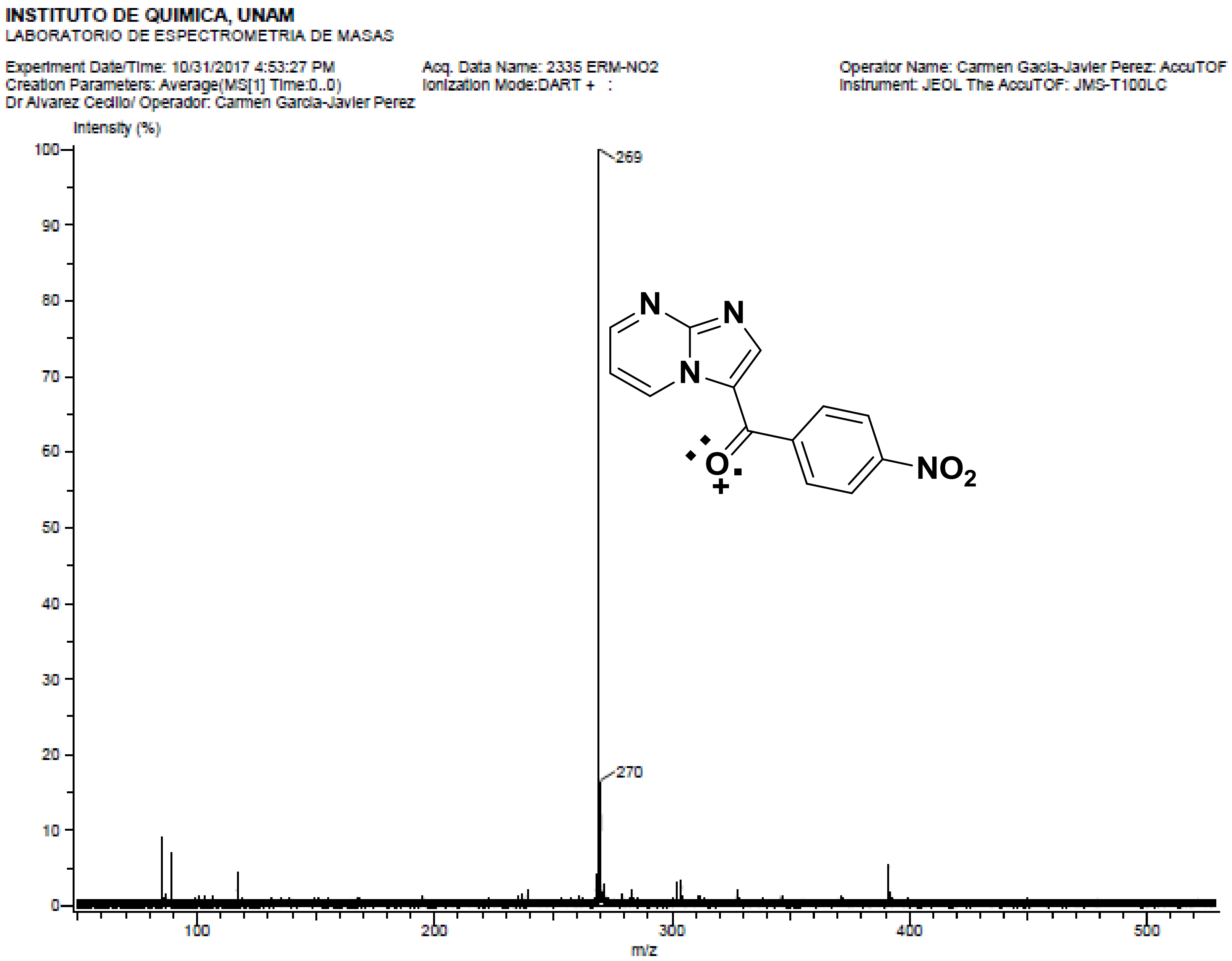

Figure S45 Mas spectrum of imidazo[1,2-*a*]pyrimidin-3-yl(4-nitrophenyl)methanone (**4g**).

Data:2335 ERM-NO2
Sample Name:Dr Alvarez Cecilio/ Operador: Carmen Garcia-Javier Perez
Description:
Ionization Mode:ESI+
History:Determine m/z[Peak Detect[Centroid,30,Area];Correct Base[10.0%]];Correct Base[5.0%];Average(MS[1] 0..1)

Acquired:10/31/2017 4:53:27 PM
Operator:AccuTOF
Mass Calibration data:Cal Peg 600
Created:11/15/2017 12:58:26 PM
Created by:

Charge number:1
Tolerance:5.00(mmu)
Unsaturation Number:0.0 .. 50.0 (Fraction:Both)
Element:^{12}C:0 .. 15, ^{1}H:0 .. 40, ^{14}N:0 .. 4, ^{16}O:0 .. 3

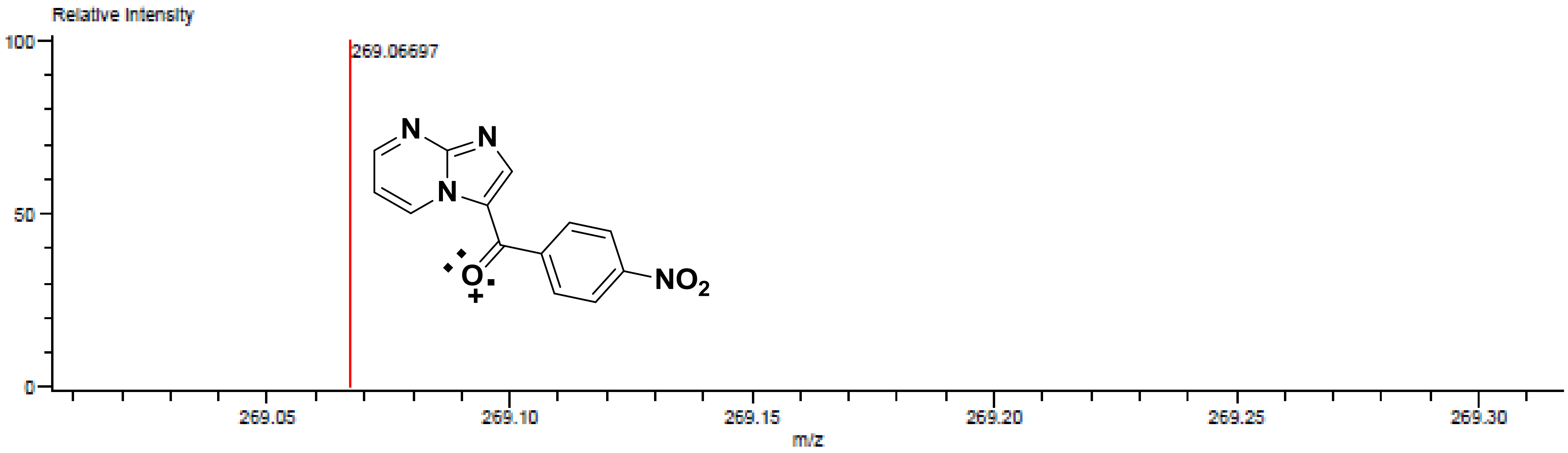

Mass	Intensity	Calc. Mass	Mass Difference (mmu)	Mass Difference (ppm)	Possible Formula	Unsaturation Number
269.06697	25870.50	269.06746	-0.49	-1.83	$^{12}C_{13}{}^{1}H_{9}{}^{14}N_{4}{}^{16}O_{3}$	11.5

Figure S46: HRMS of imidazo[1,2-*a*]pyrimidin-3-yl(4-nitrophenyl)methanone (**4g**).

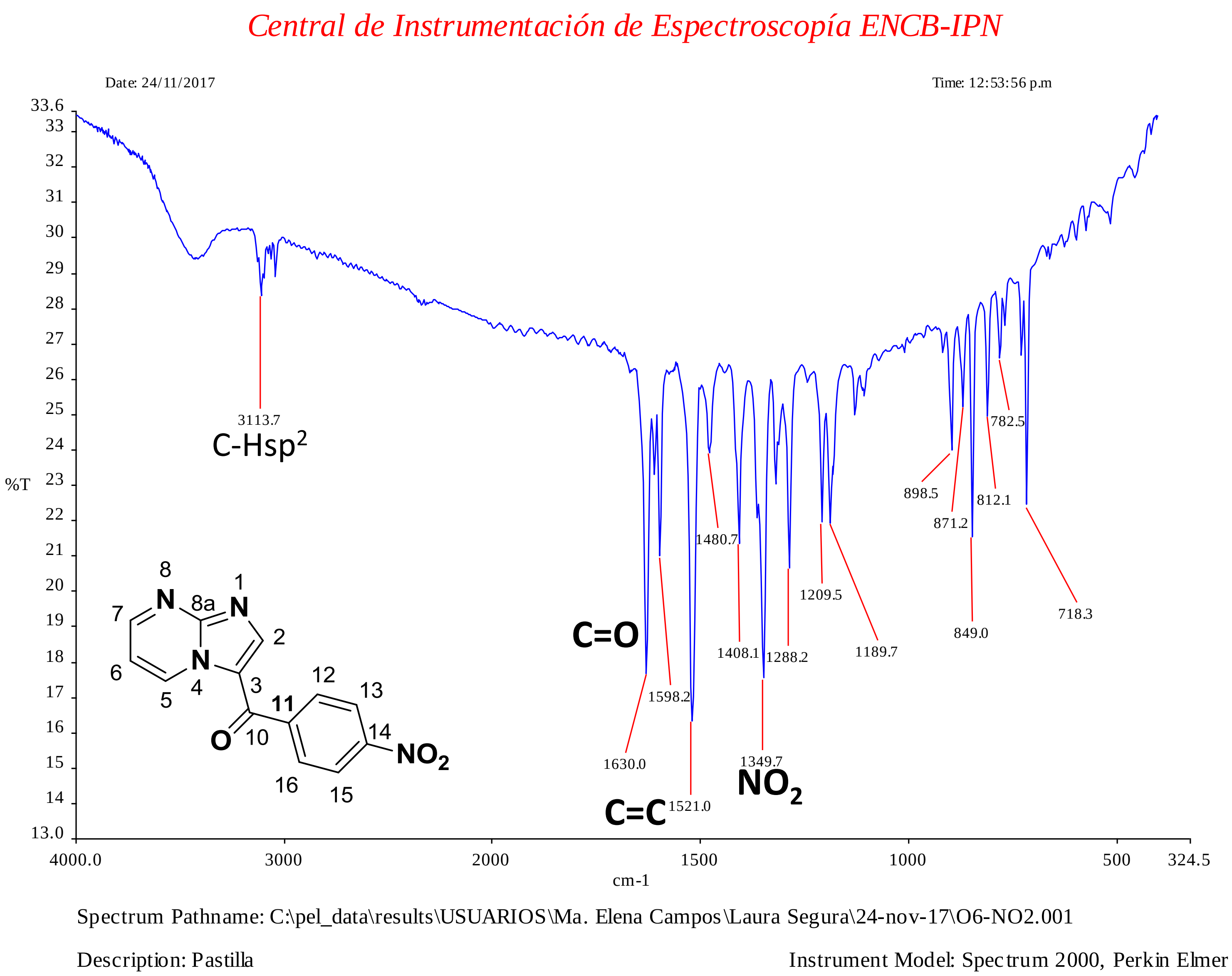

Figure S47: IR spectrum of imidazo[1,2-*a*]pyrimidin-3-yl(4-nitrophenyl)methanone (**4g**).

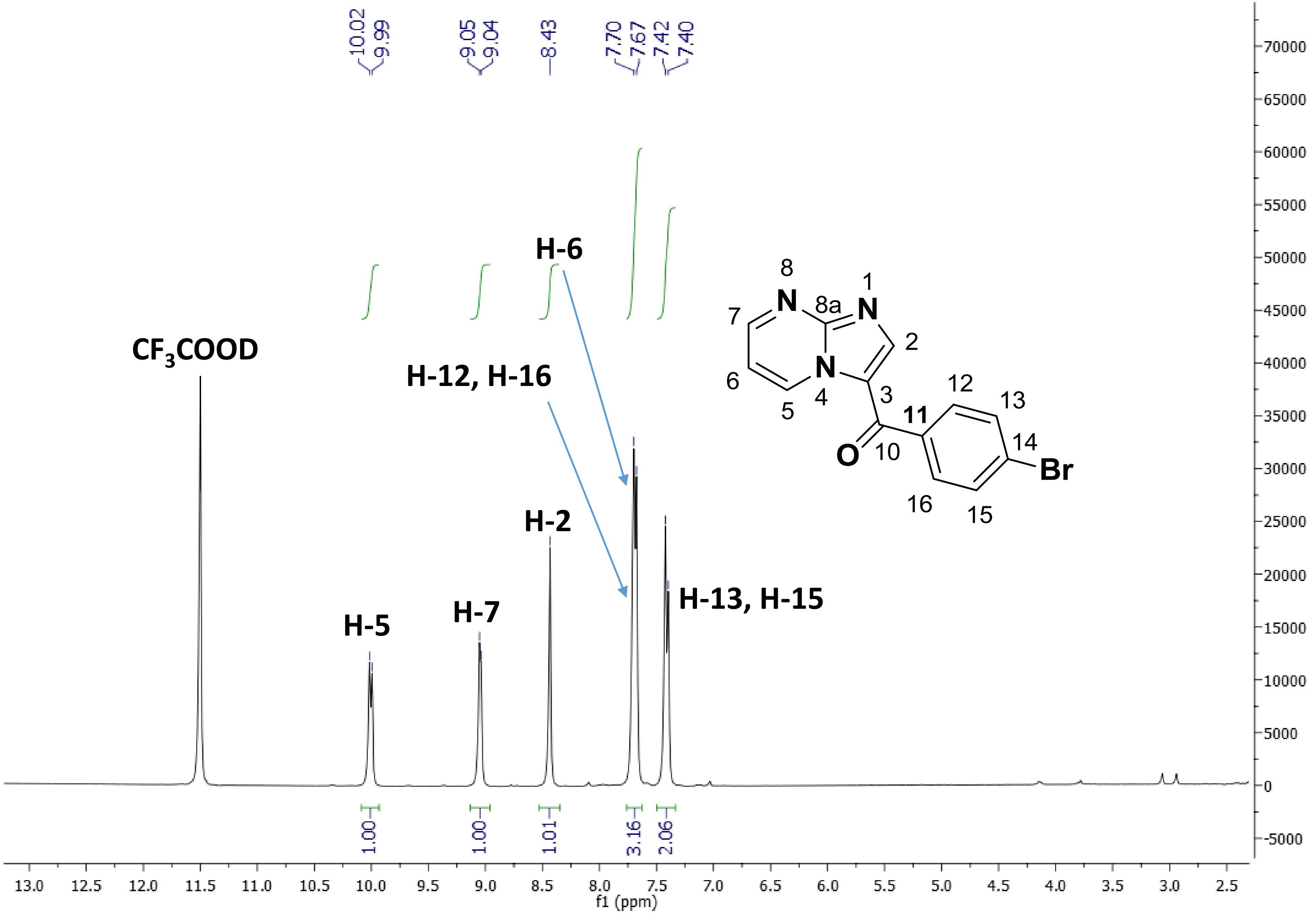

Figure S48: ^{1}H NMR (300 MHz, $CDCl_3$) of (4-bromophenyl)(imidazo[1,2-*a*]pyrimidin-3-yl)methanone (**4j**).

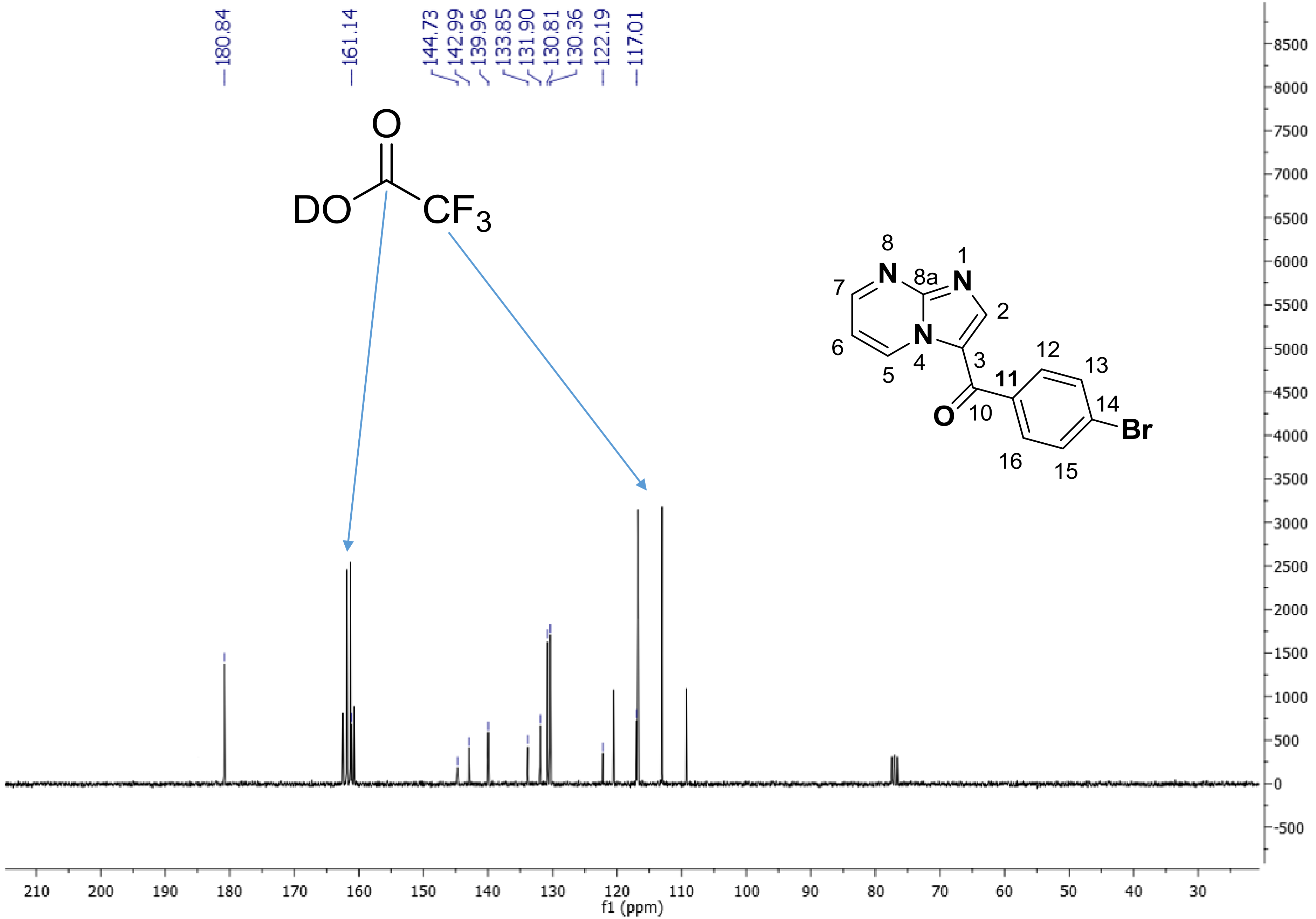

Figure S49: ^{13}C NMR (75 MHz, $CDCl_3$) of (4-bromophenyl)(imidazo[1,2-*a*]pyrimidin-3-yl)methanone (**4j**).

Instrument: JEOL GCmate
Inlet: Direct Probe

Ionization mode: EI+

Scan: 344
Base: m/z 281; 1.2%FS TIC: 178048

R.T.: 4.6

#Ions: 229

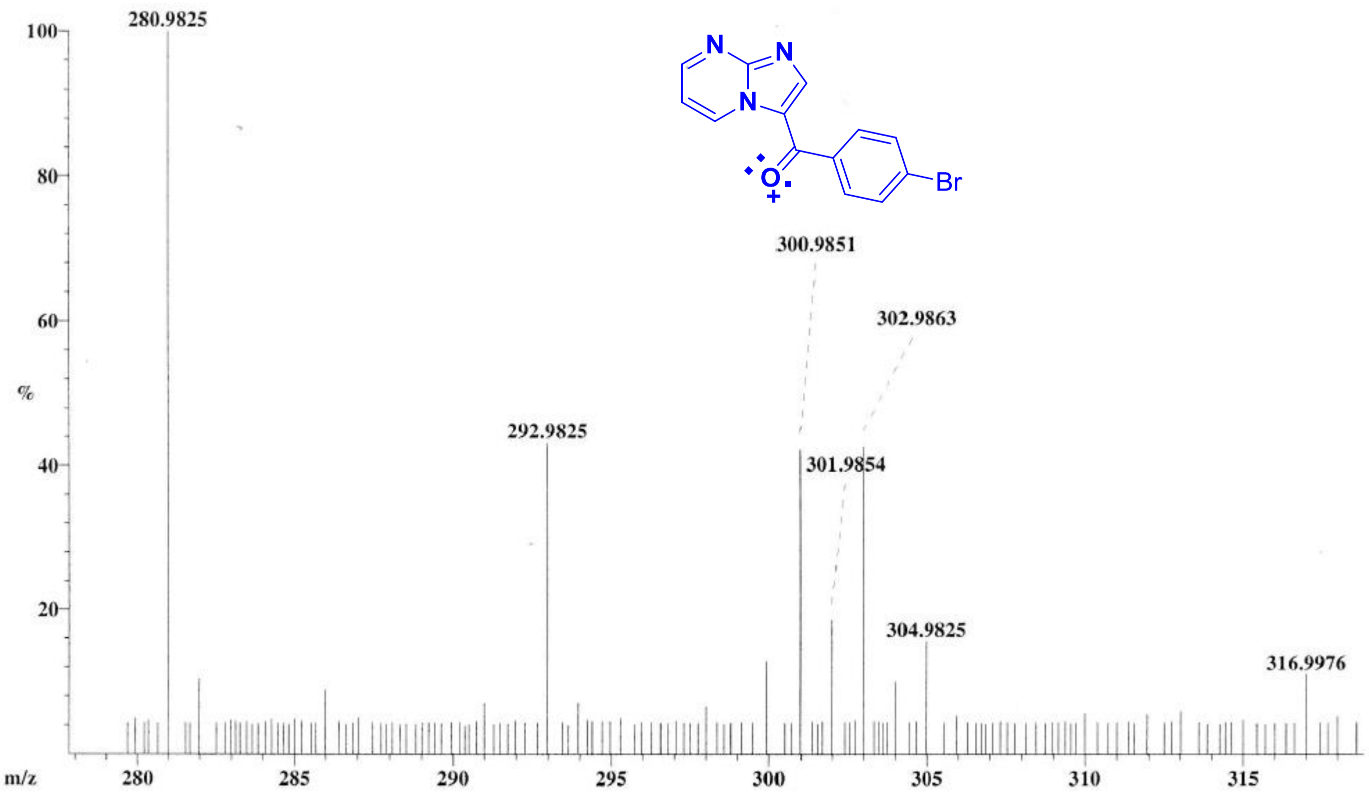

Selected Isotopes : $H_{0-8}C_{0-13}N_{0-3}O_{0-1}Br_{0-1}$

Error Limit : 5 ppm

Measured Mass	*% Base*	*Formula*	*Calculated Mass*	*Error*
300.9851	42.3%	$C_{13}H_8N_3O$ Br	300.9851	0.1

Figure S50: EREIMS of (4-bromophenyl)(imidazo[1,2-*a*]pyrimidin-3-yl)methanone (**4j**).

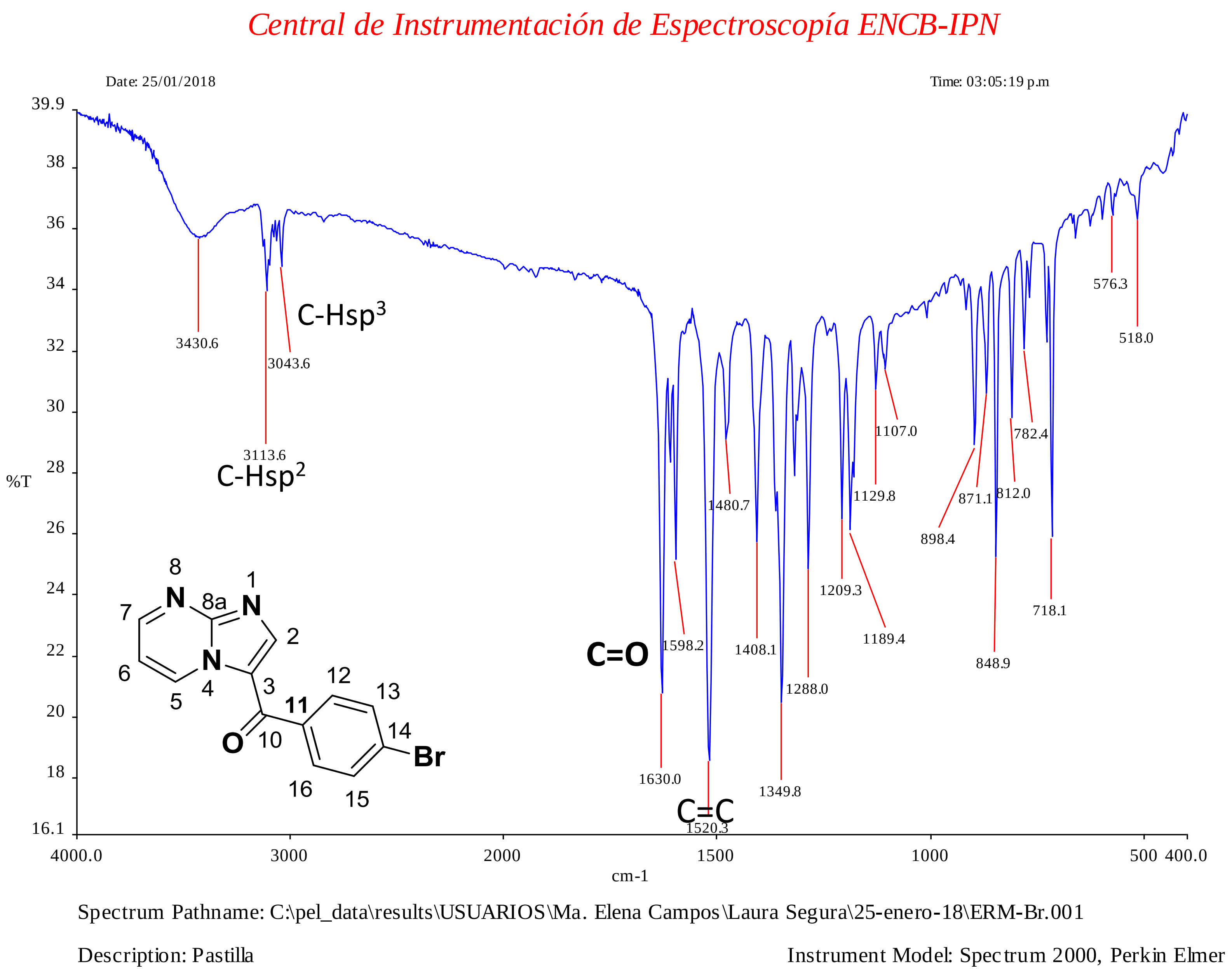

Figure S51: IR spectrum of (4-bromophenyl)(imidazo[1,2-*a*]pyrimidin-3-yl)methanone (**4j**).

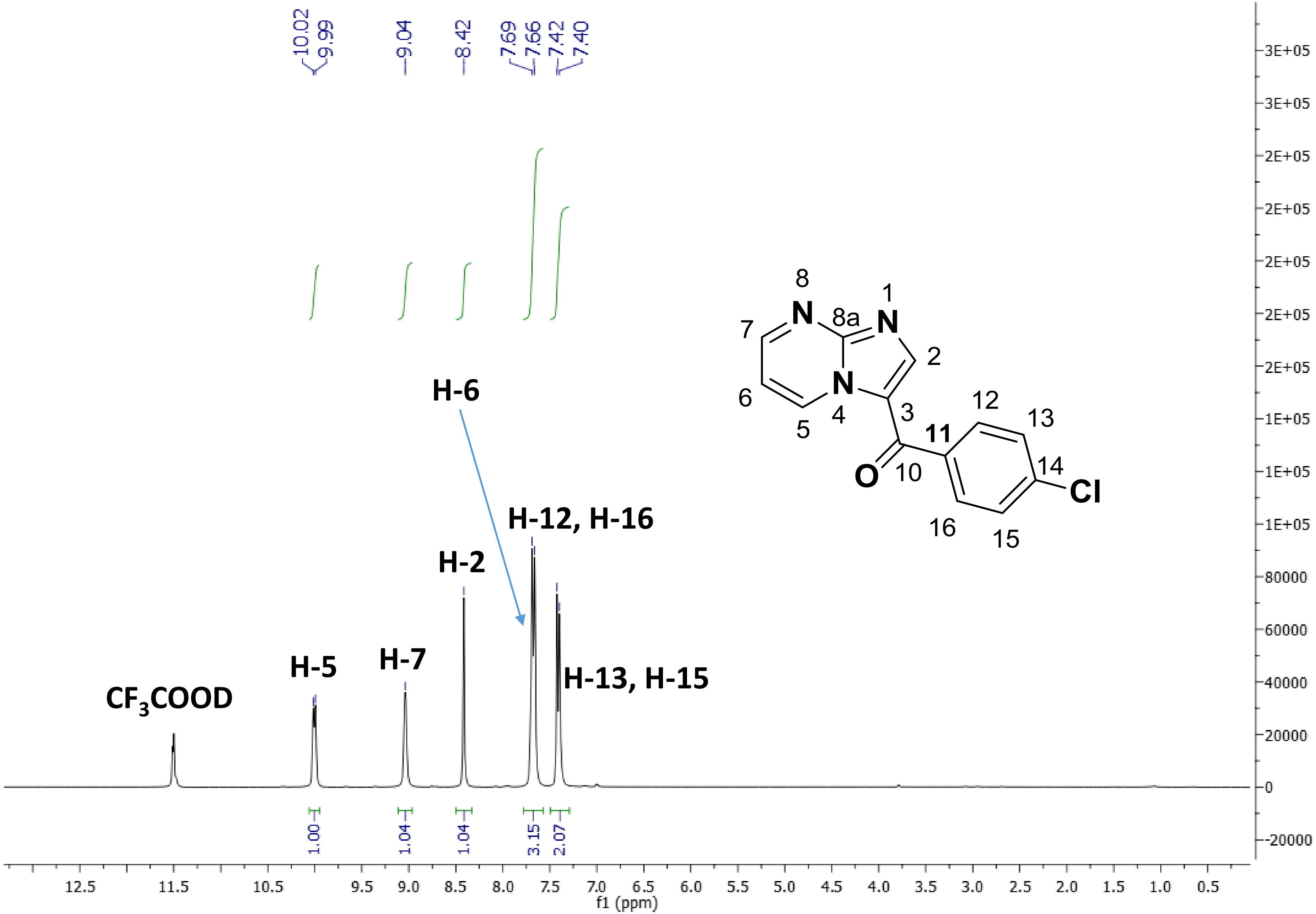

Figure S52: ^{1}H NMR (300 MHz, $CDCl_3$) of (4-chlorophenyl)(imidazo[1,2-*a*]pyrimidin-3-yl)methanone (**4h**).

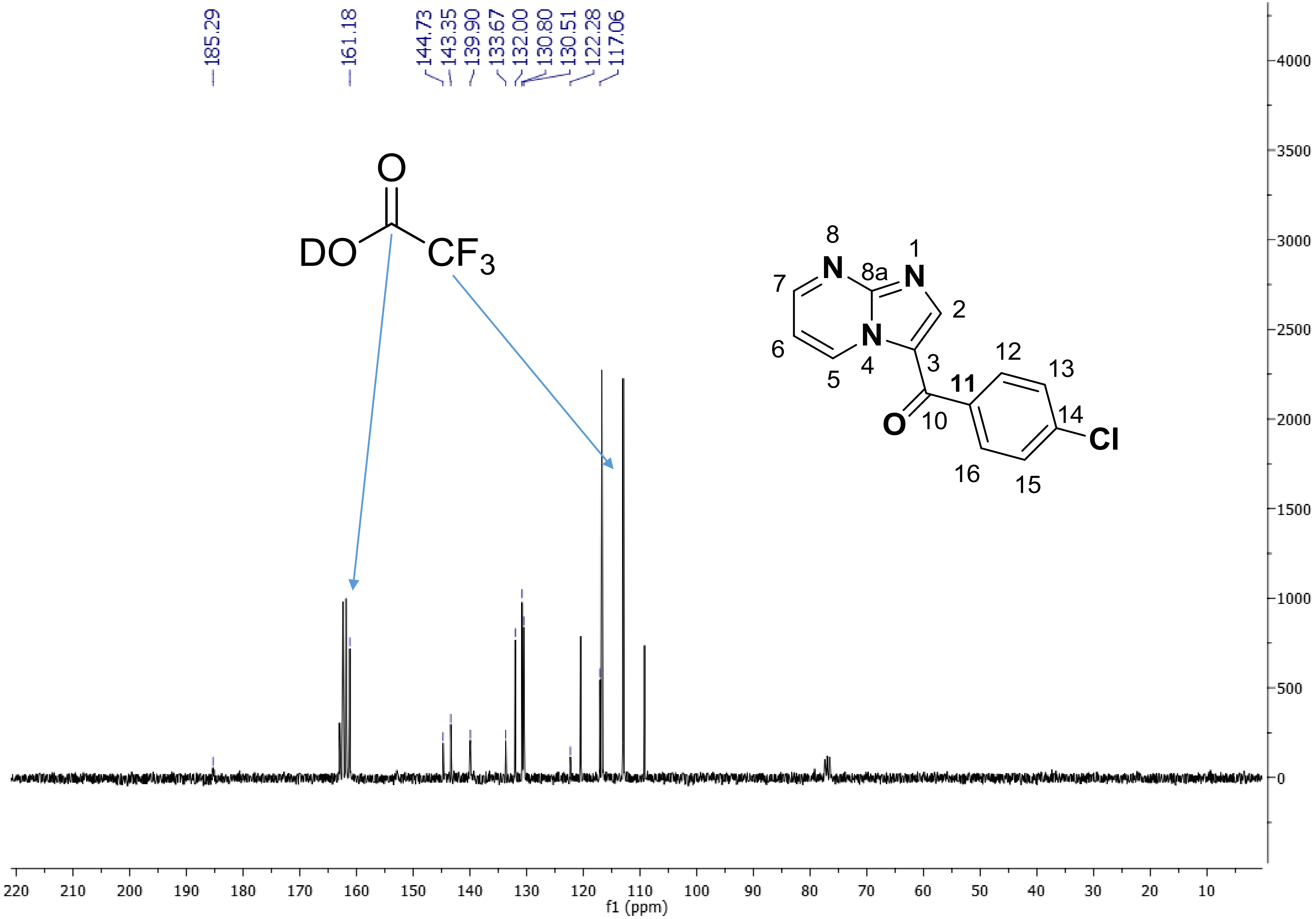

Figure S53: ^{13}C NMR (75 MHz, $CDCl_3$) of (4-chlorophenyl)(imidazo[1,2-*a*]pyrimidin-3-yl)methanone (**4h**).

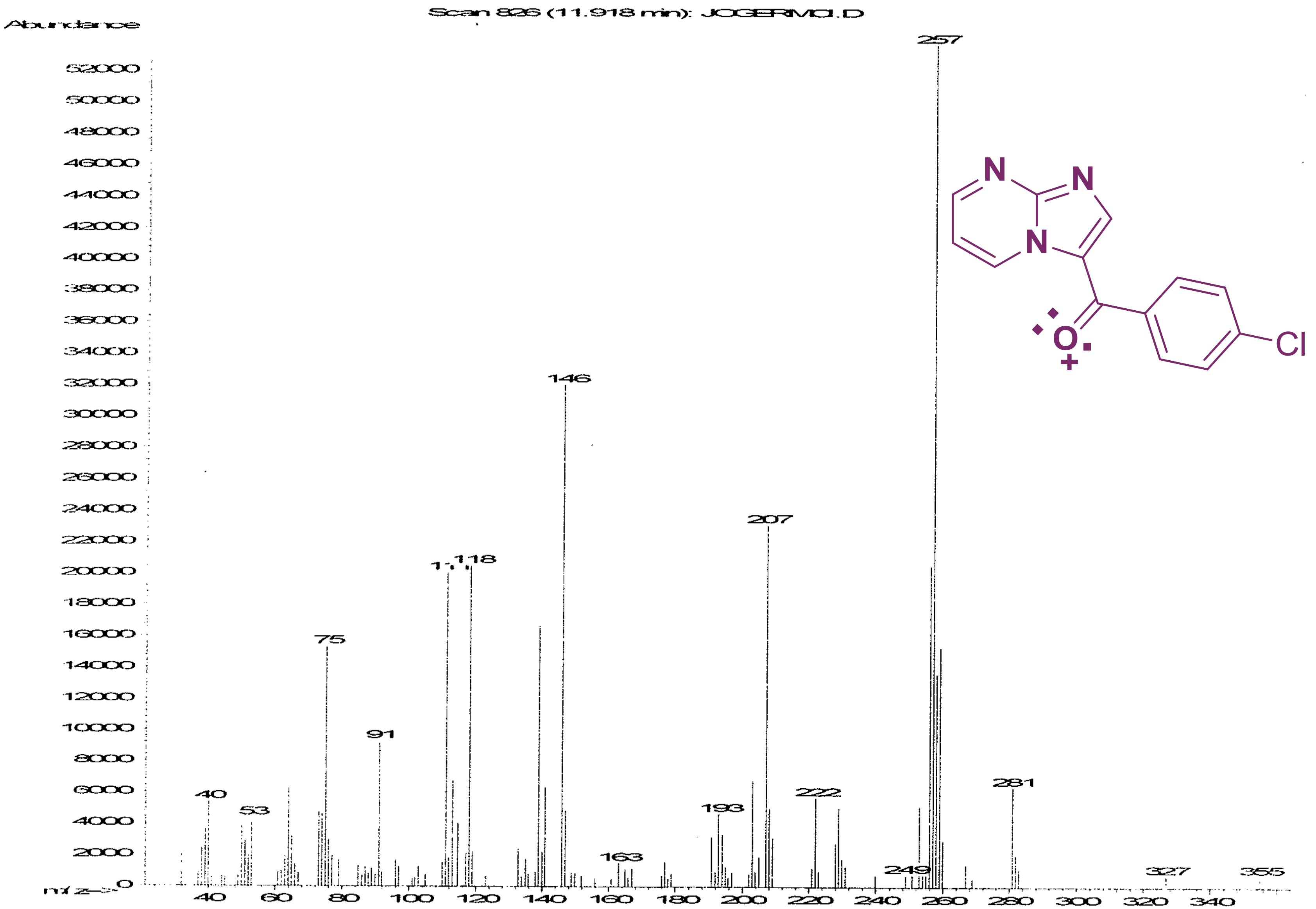

Figure S54: Mass spectrum of (4-chlorophenyl)(imidazo[1,2-*a*]pyrimidin-3-yl)methanone (**4h**).

Instrument: JEOL GCmate
Inlet: Direct Probe
Ionization mode: EI+

Scan: 197
R.T.: 2.61
Base: m/z 219; 3.6%FS TIC: 296512
#Ions: 207

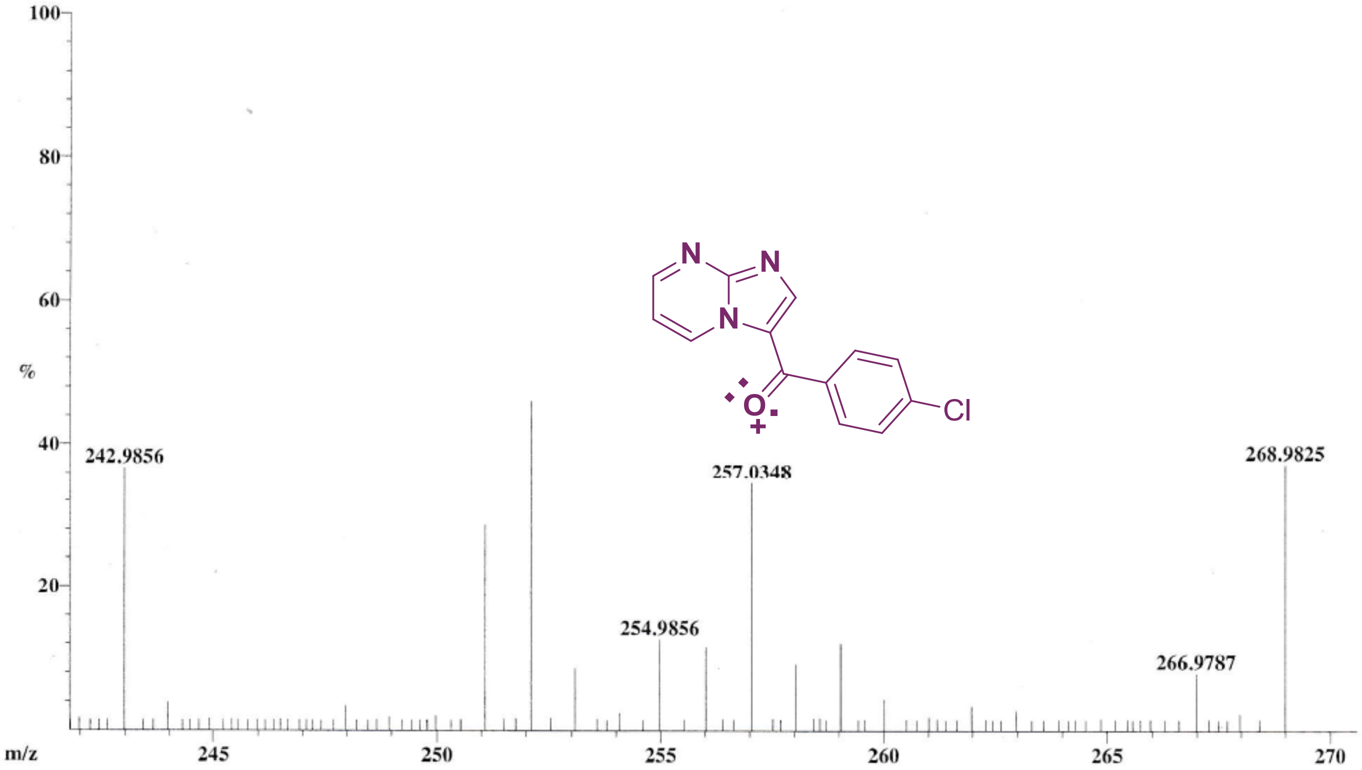

Selected Isotopes : $H_{0\text{-}8}C_{0\text{-}13}N_{0\text{-}3}O_{0\text{-}1}Cl_{0\text{-}1}$

Error Limit : 5 ppm

Measured Mass	*% Base*	*Formula*	*Calculated Mass*	*Error*
257.0348	34.6%	$C_{13}H_8N_3O$ Cl	257.0356	-3.1

Figure S55: HREIMS of (4-chlorophenyl)(imidazo[1,2-*a*]pyrimidin-3-yl)methanone (**4h**).

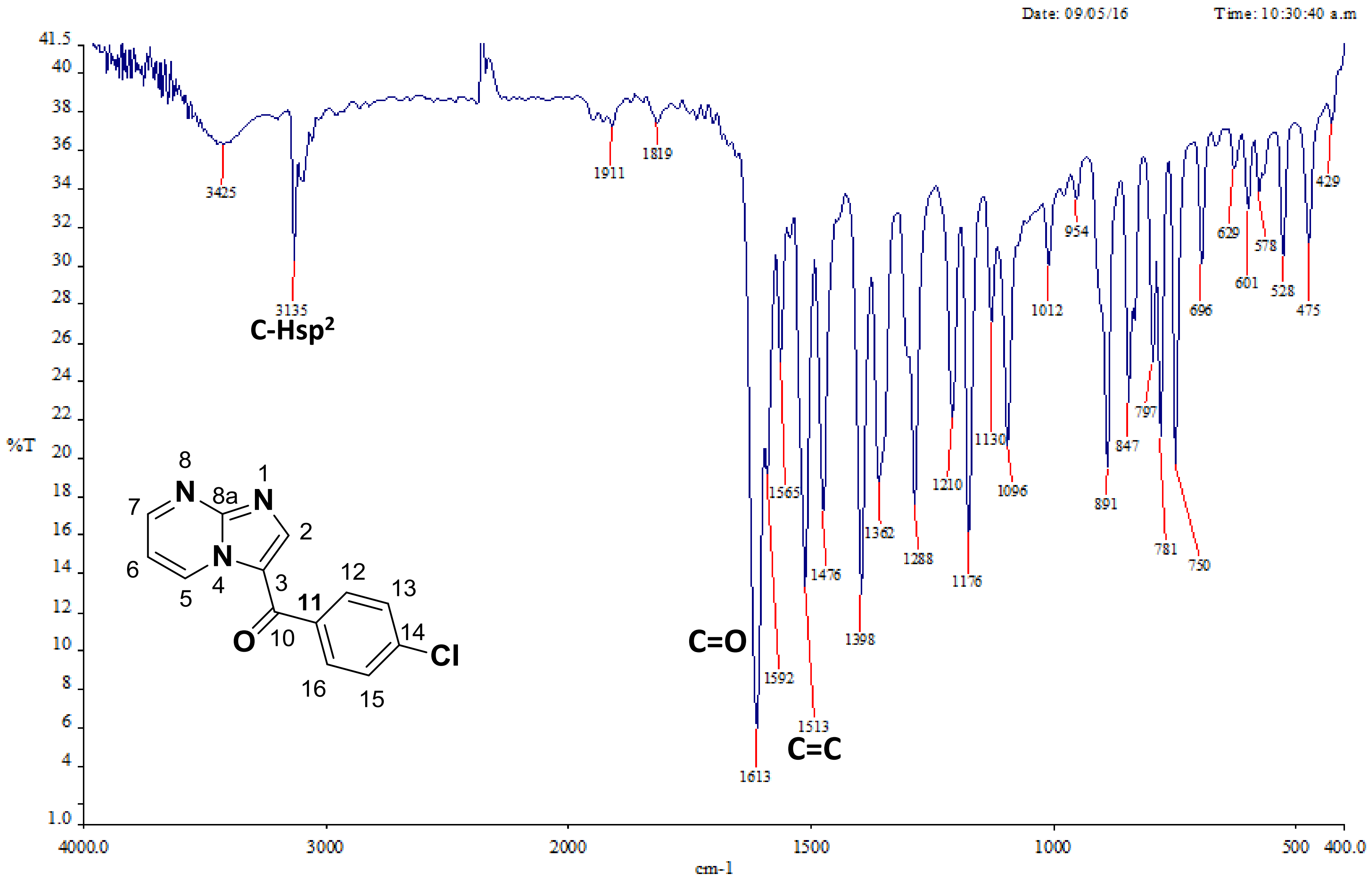

Figure S56: Mass spectrum of (4-chlorophenyl)(imidazo[1,2-*a*]pyrimidin-3-yl)methanone (**4h**).

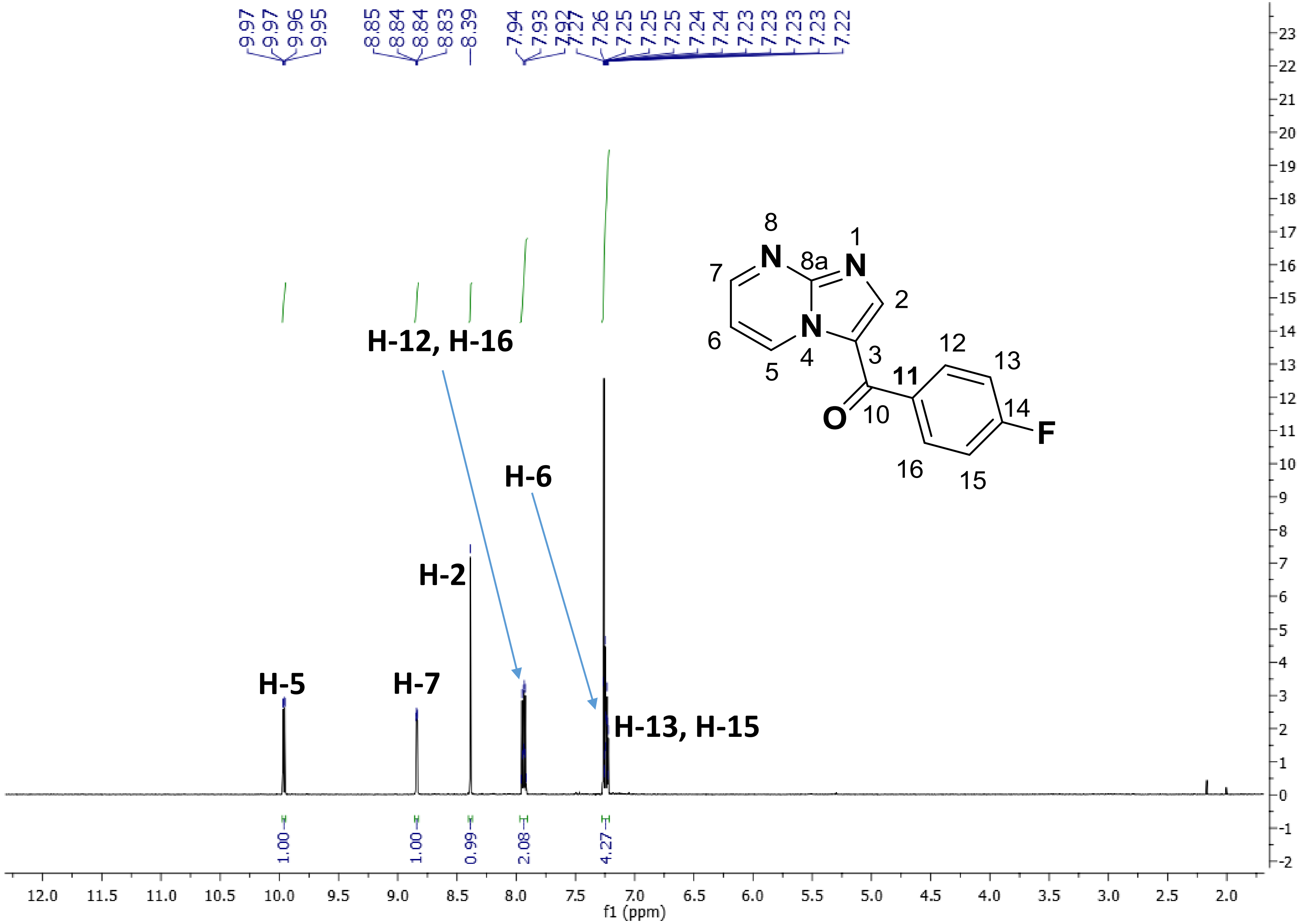

Figure S57: ^{1}H NMR (500 MHz, $CDCl_3$) of (4-fluorophenyl)(imidazo[1,2-*a*]pyrimidin-3-yl)methanone (**4i**).

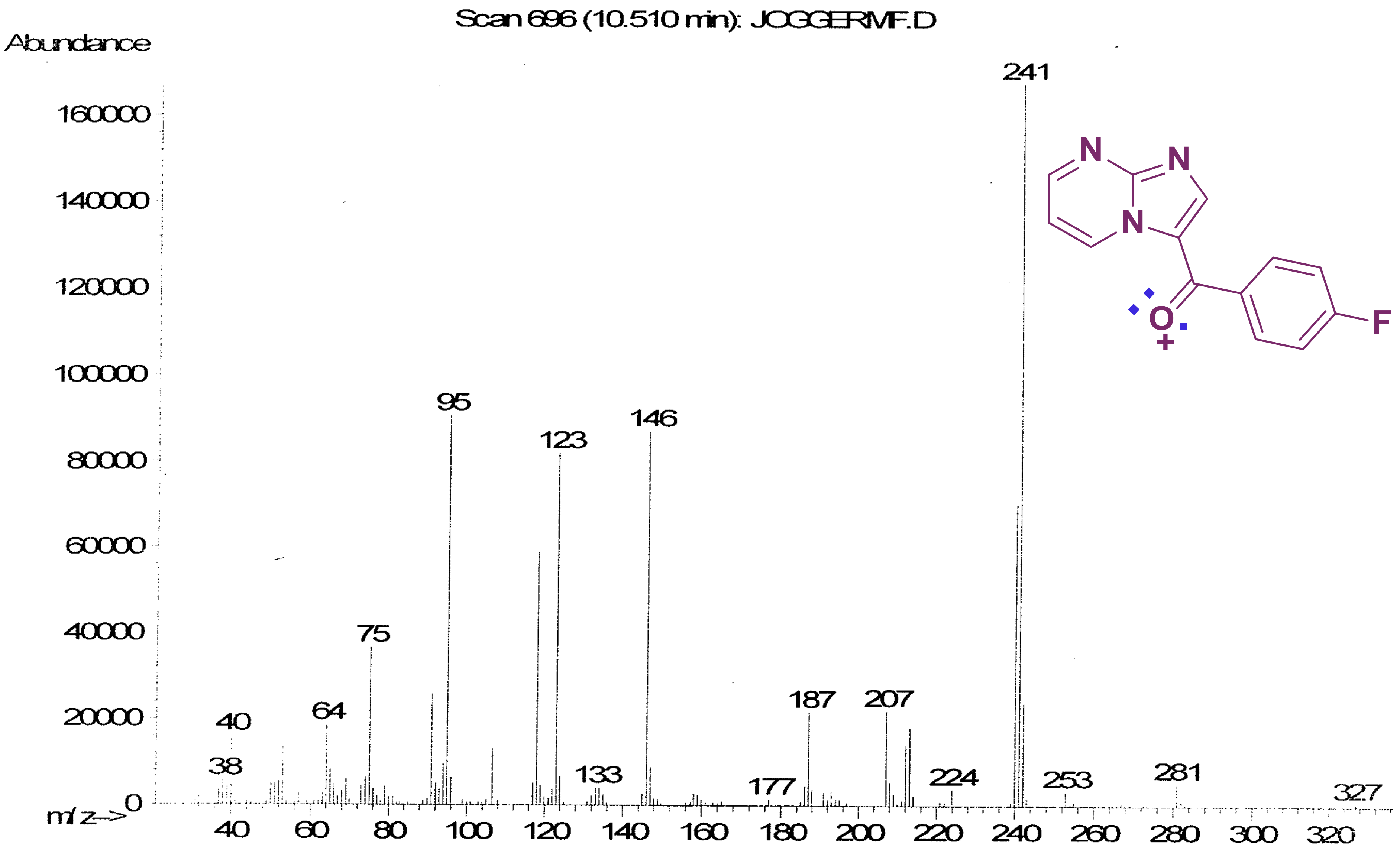

Figure S58: Mass spectrum of (4-fluorophenyl)(imidazo[1,2-*a*]pyrimidin-3-yl)methanone (**4i**).

Instrument: JEOL GCmate
Inlet: Direct Probe Ionization mode: EI+

Scan: 304 R.T.: 4.03
Base: m/z 241; 1.8%FS TIC: 204272 #Ions: 226

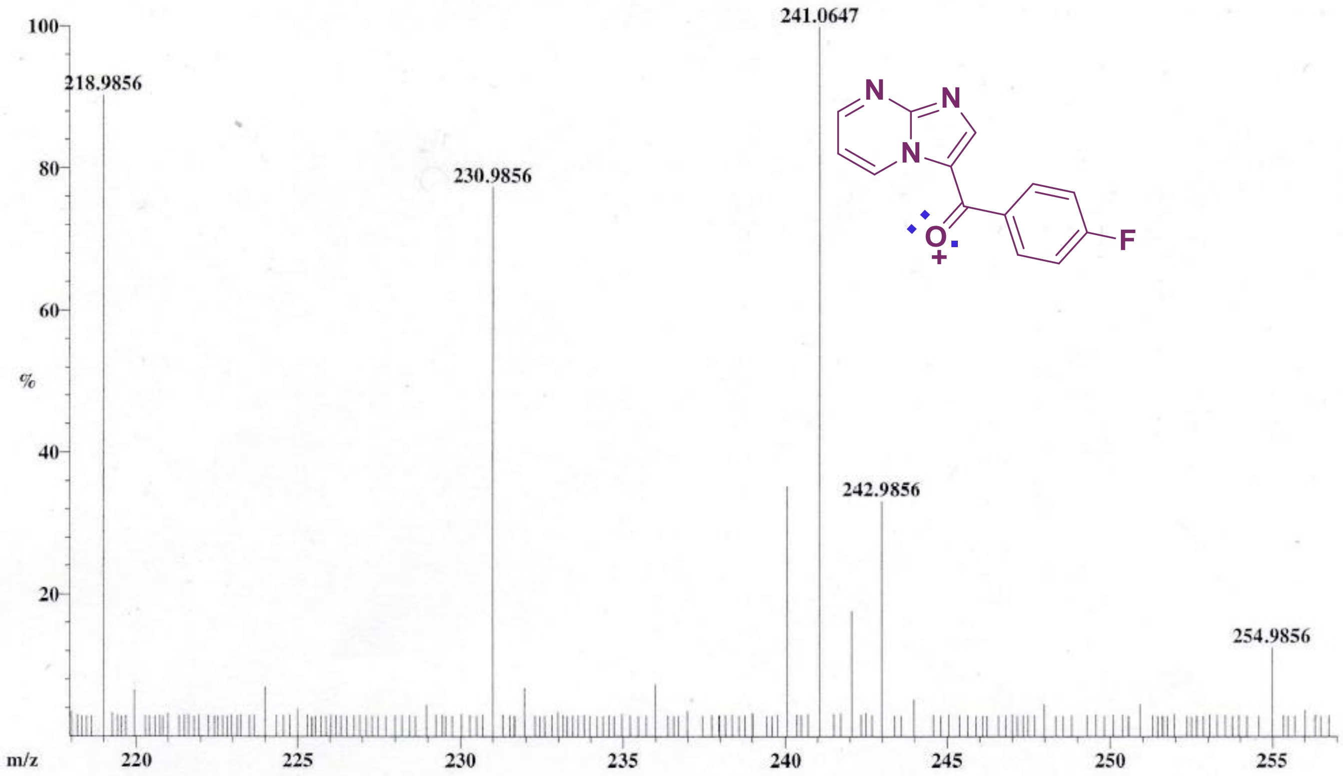

Selected Isotopes : $H_{0-8}C_{0-13}N_{0-3}O_{0-1}F_{0-1}$ Error Limit : 5 ppm

Measured Mass	*% Base*	*Formula*	*Calculated Mass*	*Error*
241.0647	100.0%	$C_{13}H_8N_3O F$	241.0651	-1.8

Figure S59: HREIMS of (4-fluorophenyl)(imidazo[1,2-*a*]pyrimidin-3-yl)methanone (**4i**).

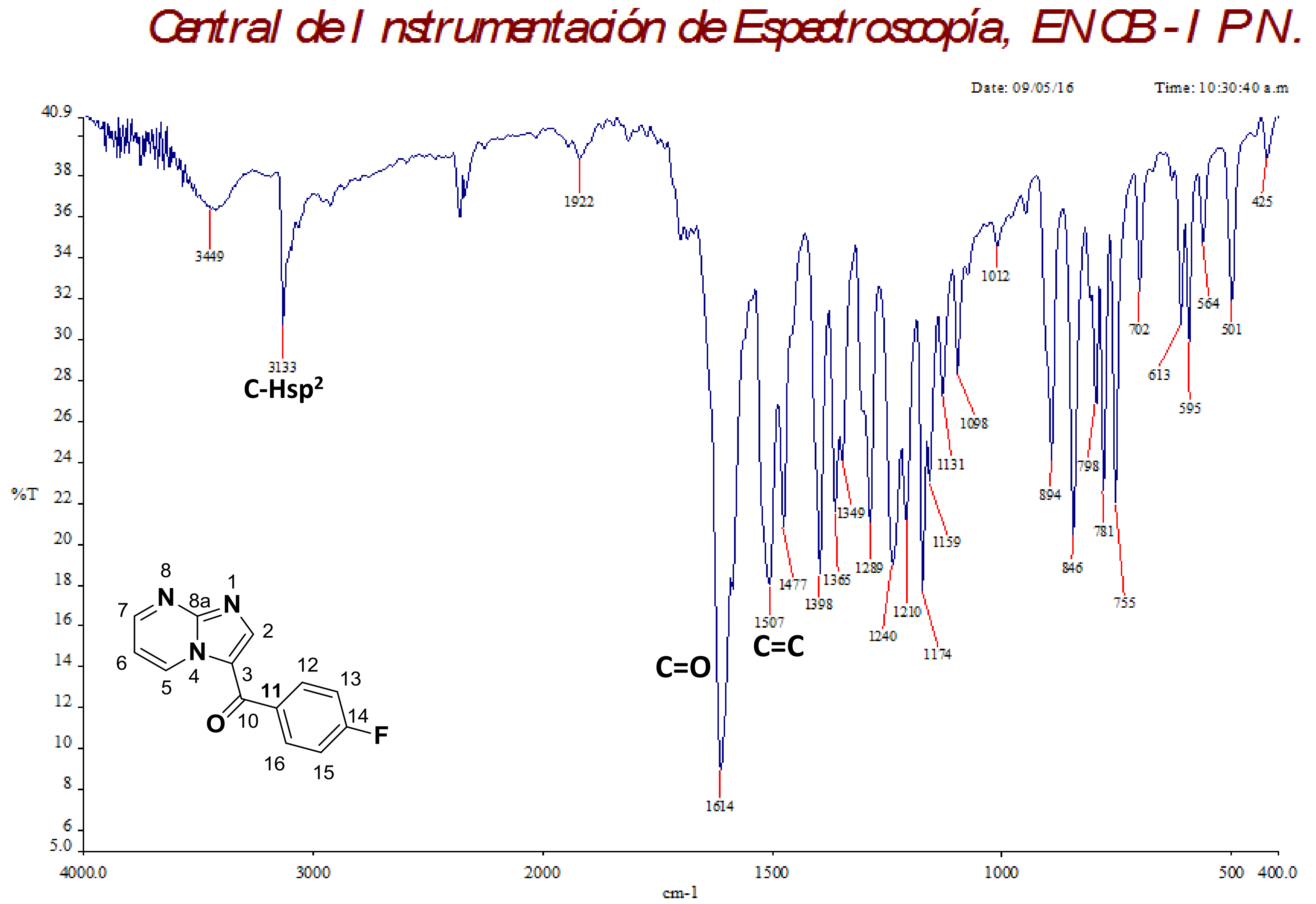

Figure S60: IR spectrum of (4-fluorophenyl)(imidazo[1,2-*a*]pyrimidin-3-yl)methanone (**4i**).

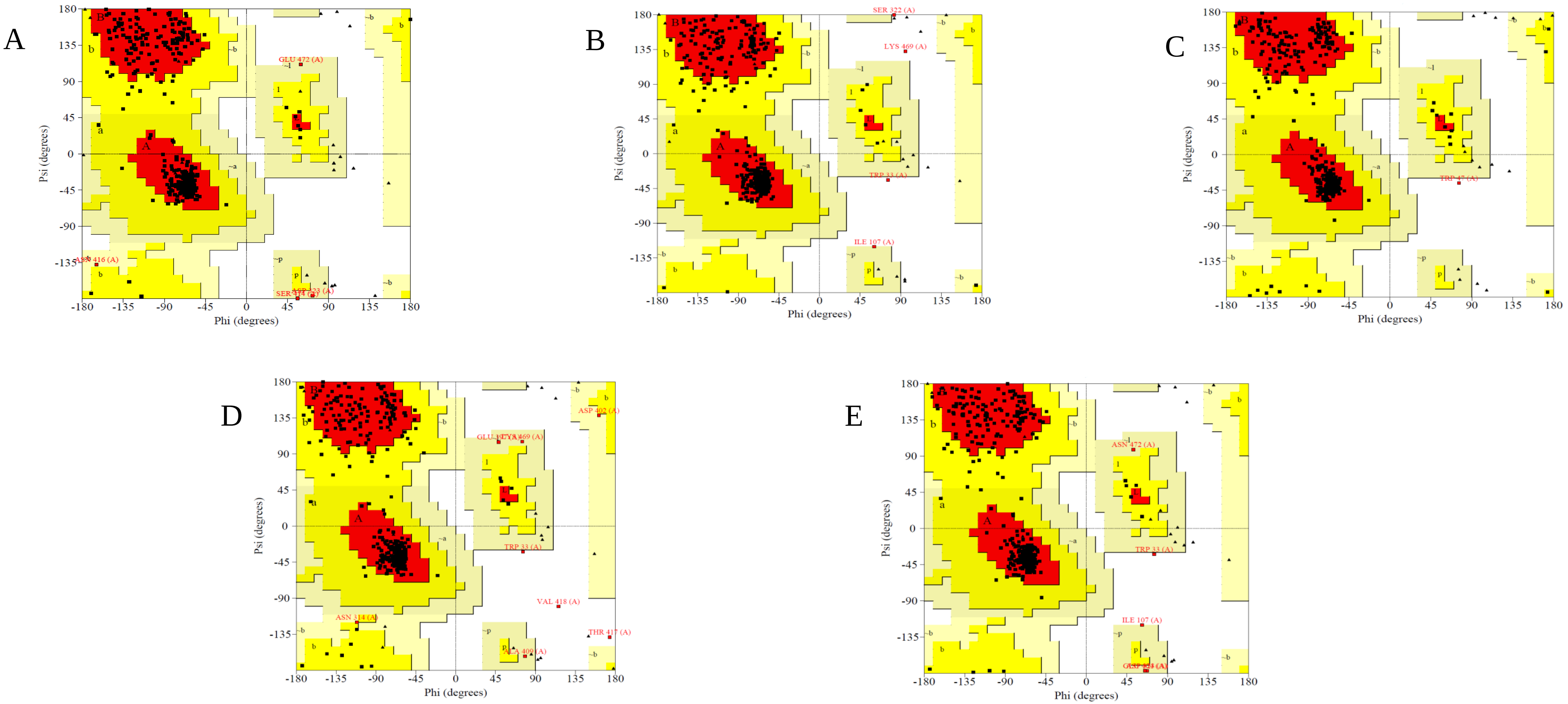

Figure S61: Distribution diagrams of Ramachandran psi-phi plots of the CYP51 models. A, $CYP51_{Cd}$. B, $CYP51_{Cgui}$. C, $CYP51_{Ck}$. D, $CYP51_{Cke}$. E, $CYP51_{Ct}$. The favorable residues [A,B,L] are in the red zone, additional allowed residues [a,b,l,p] in the yellow zones, generously permitted residues [~a,~b,~l,~p] in the beige zone, and not allowed regions in the white zone.

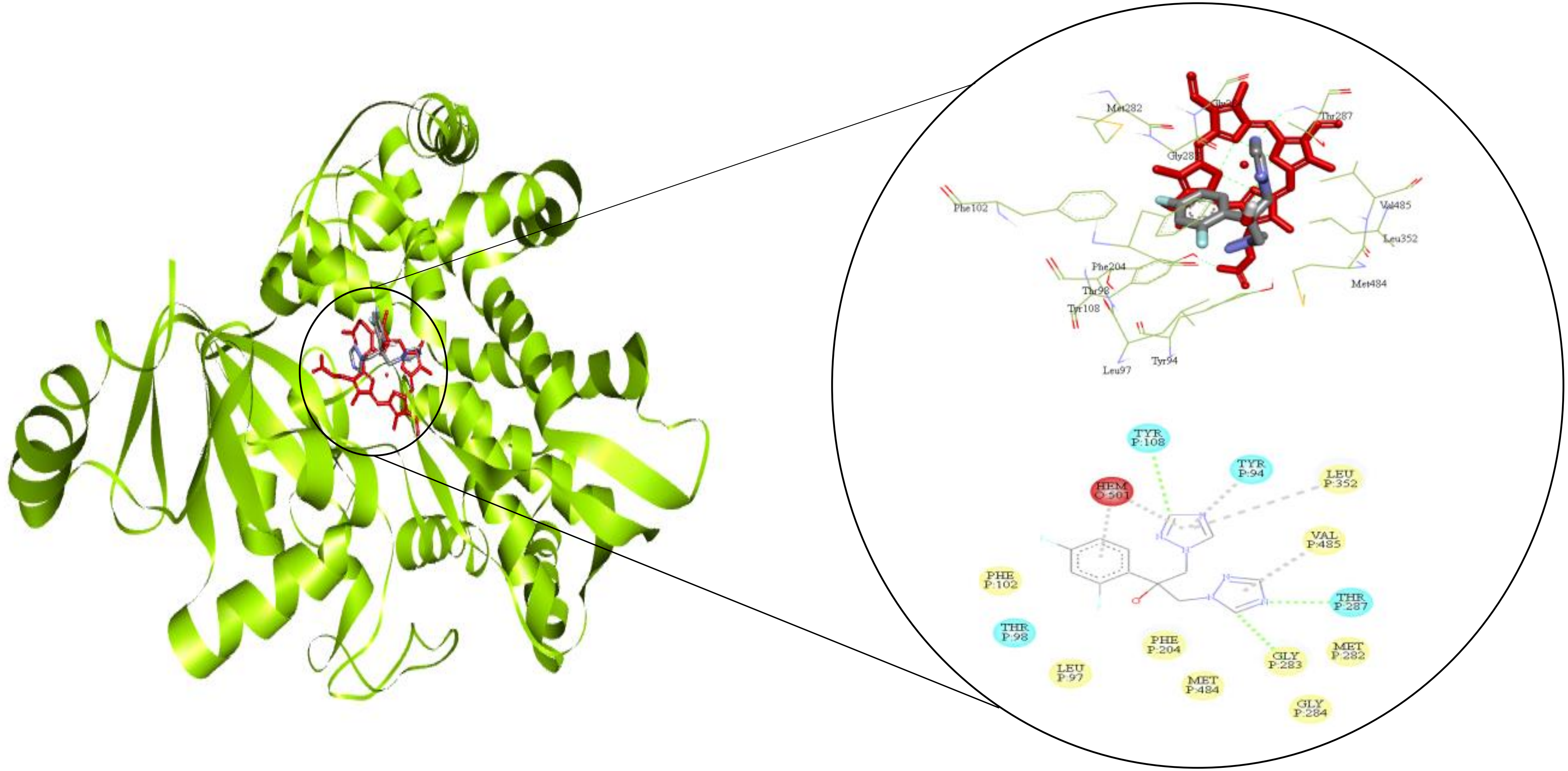

Figure S62: Schematic representation of the interactions of fluconazole with $CYP51_{Ca.}$

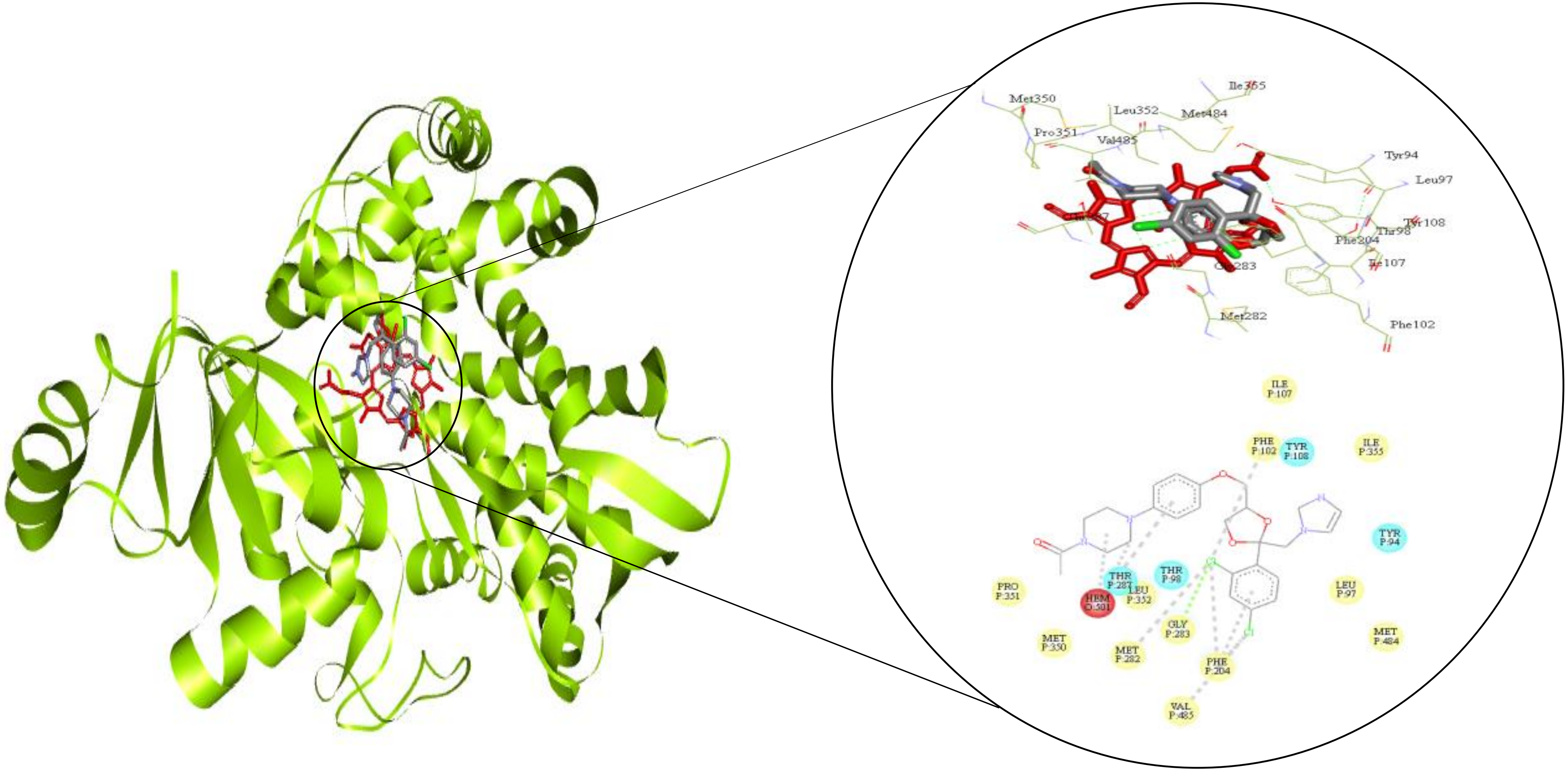

Figure S63: Schematic representation of the interactions of ketoconazole with $CYP51_{Ca.}$

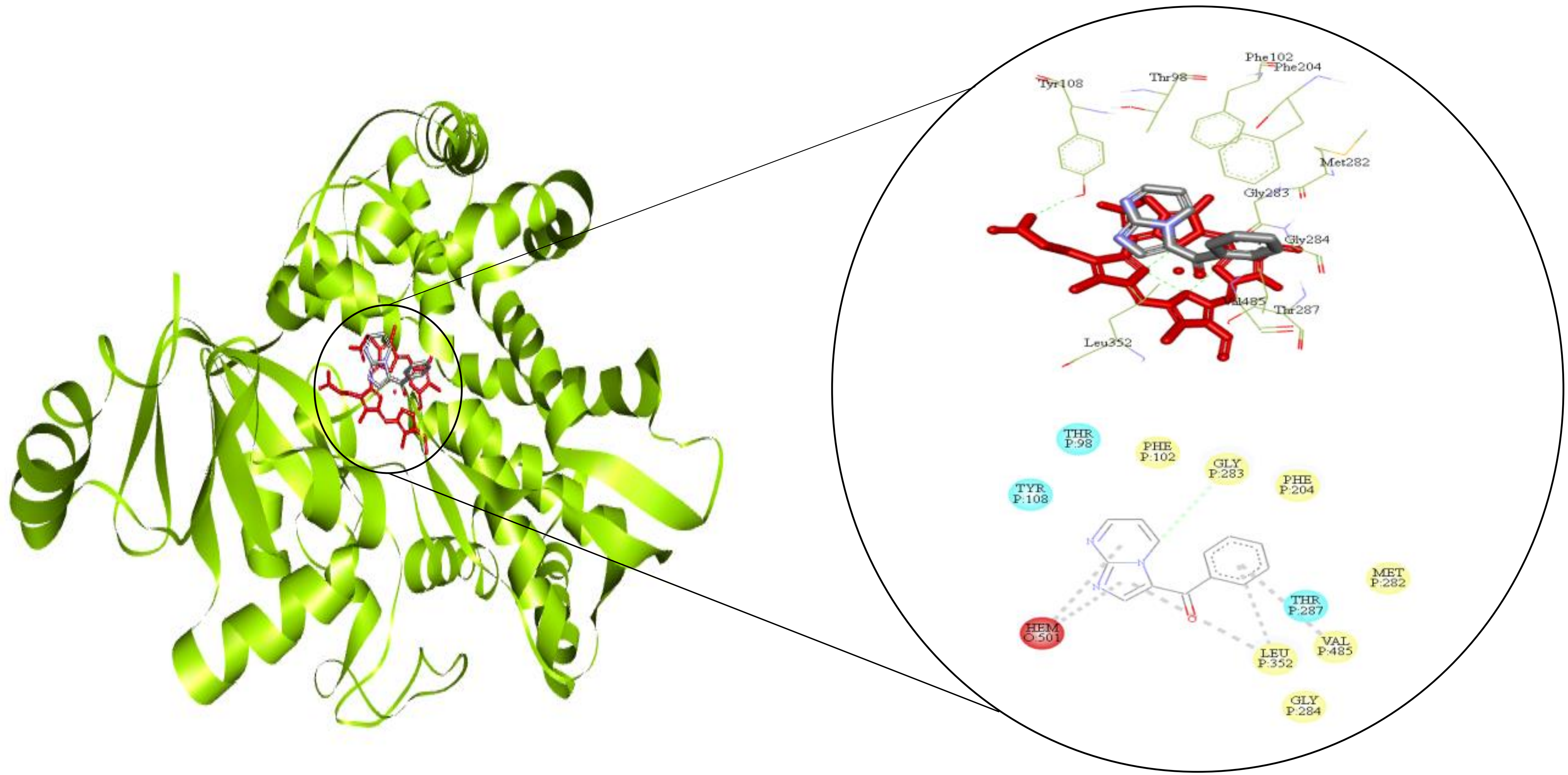

Figure S64: Schematic representation of the interactions of 4a with $CYP51_{Ca.}$

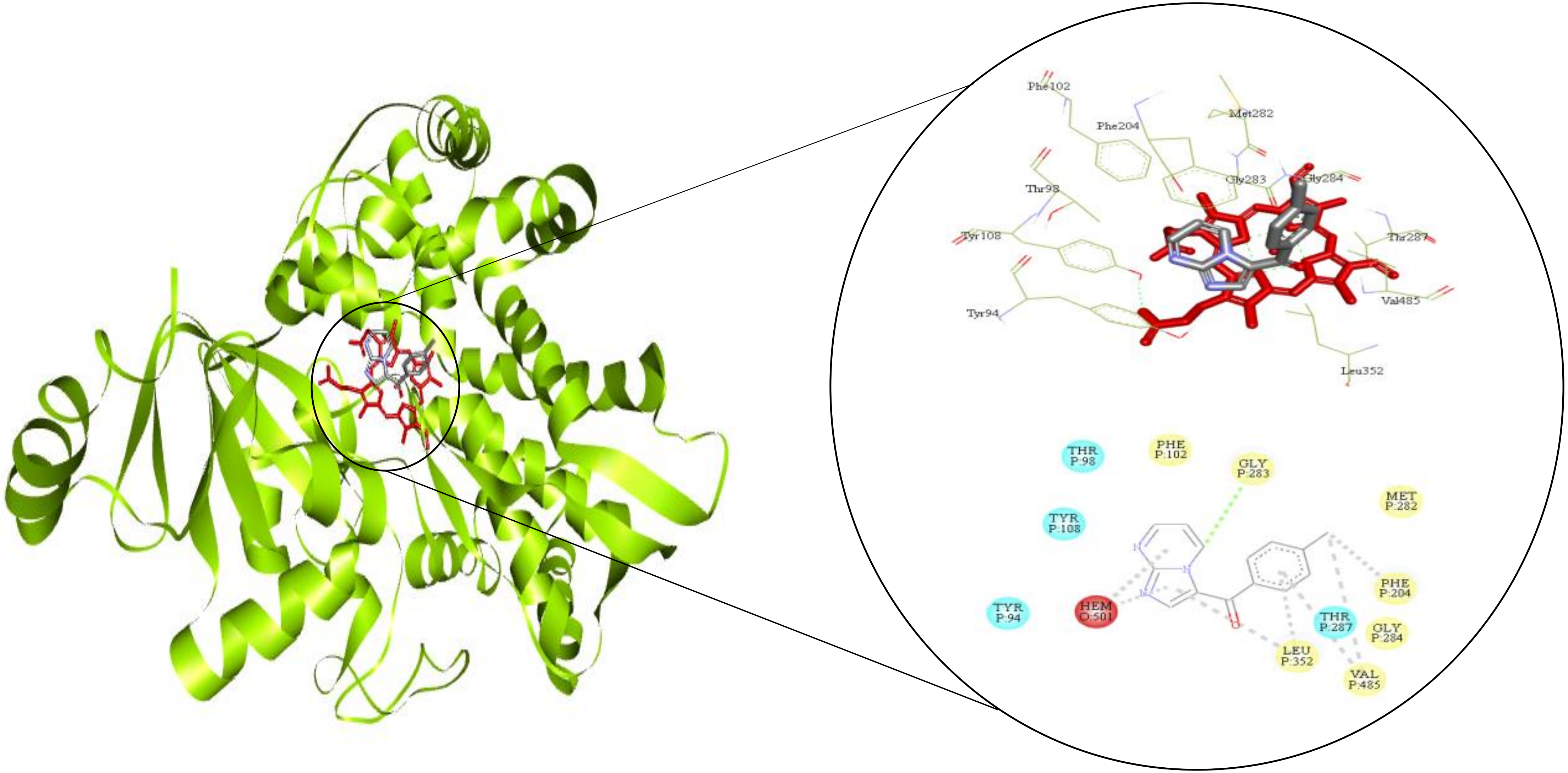

Figure S65: Schematic representation of the interactions of 4d with $CYP51_{Ca.}$

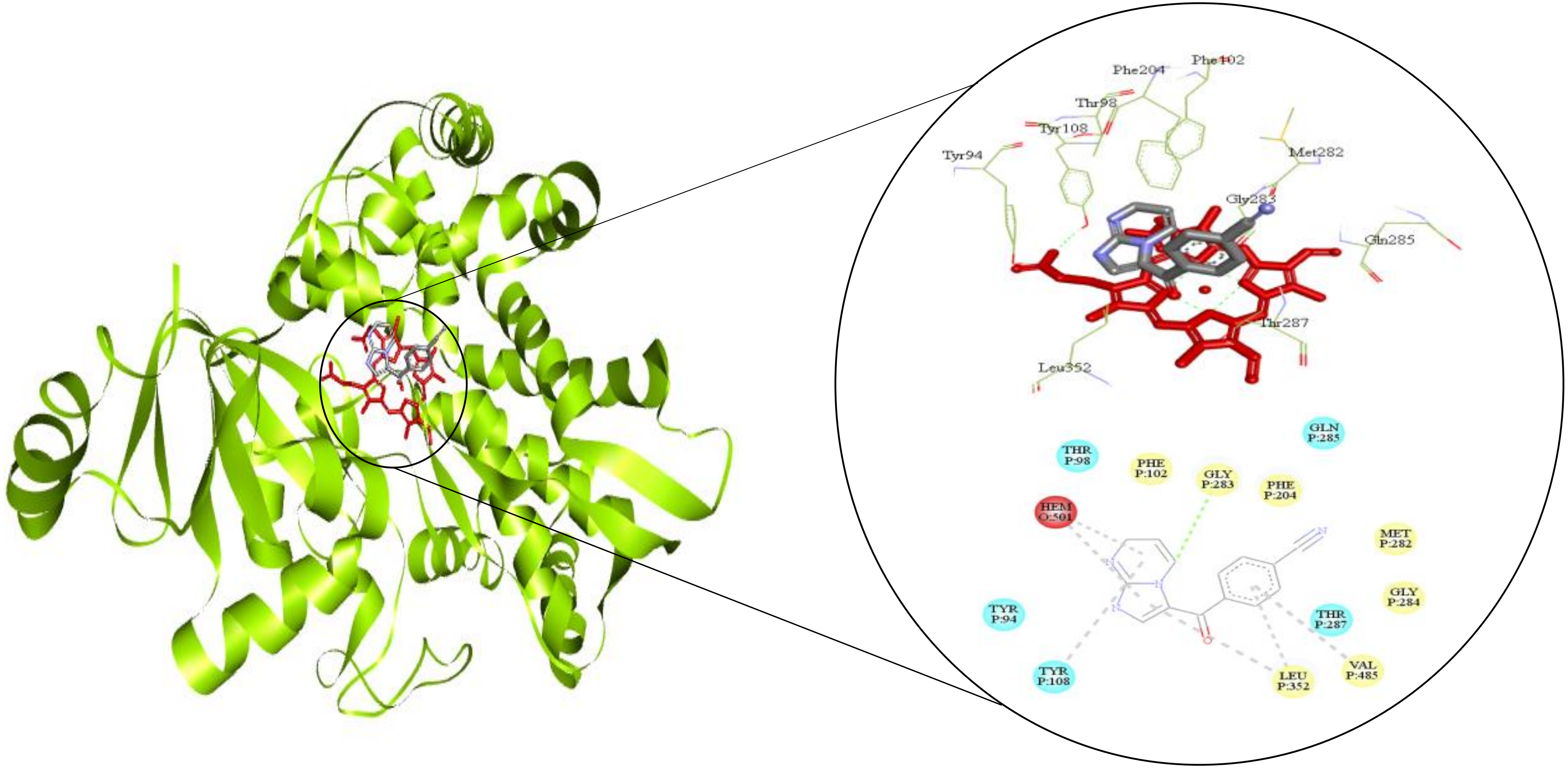

Figure S66: Schematic representation of the interactions of 4f with $CYP51_{Ca.}$

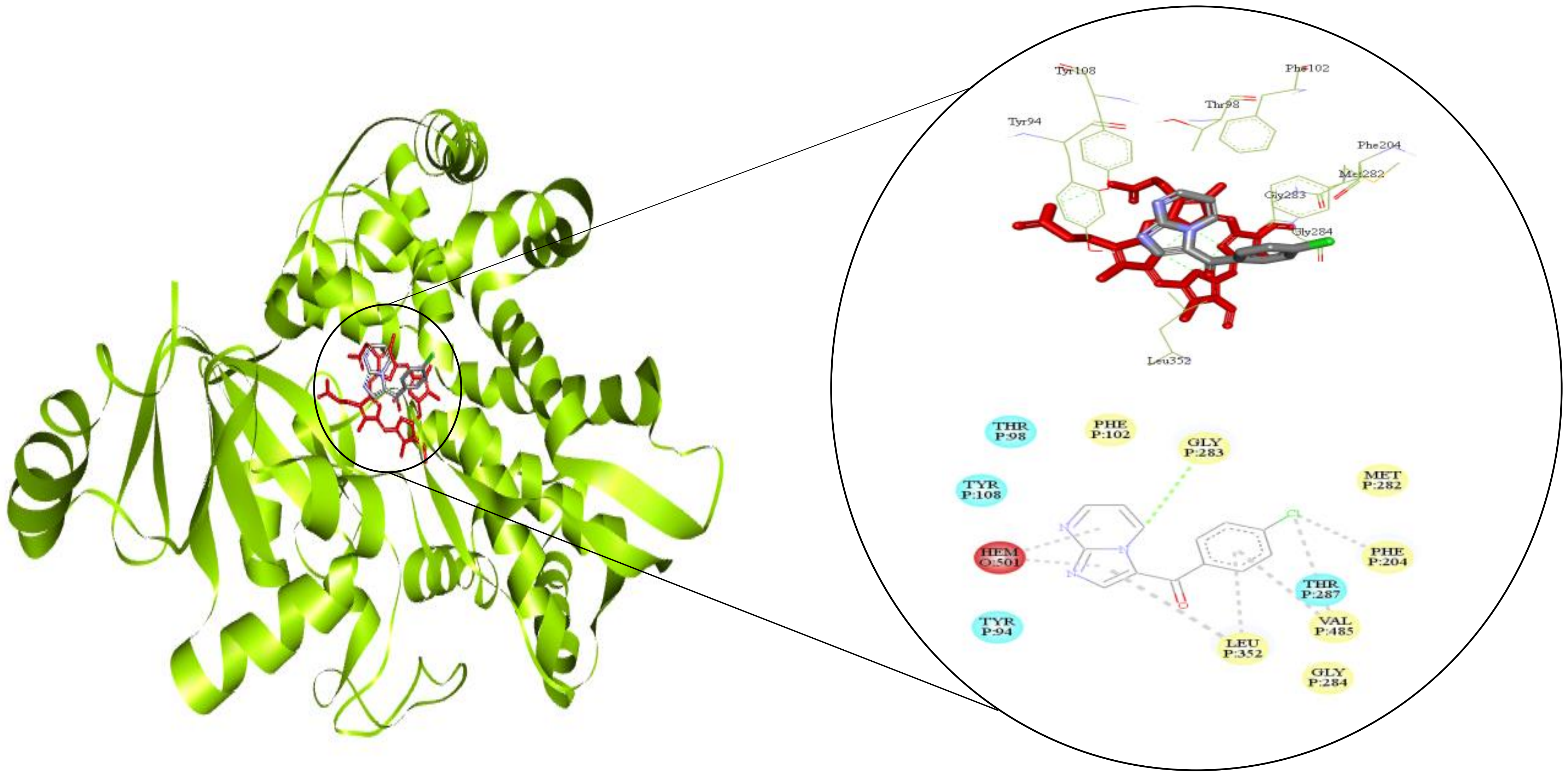

Figure S67: Schematic representation of the interactions of 4i with $CYP51_{Ca.}$

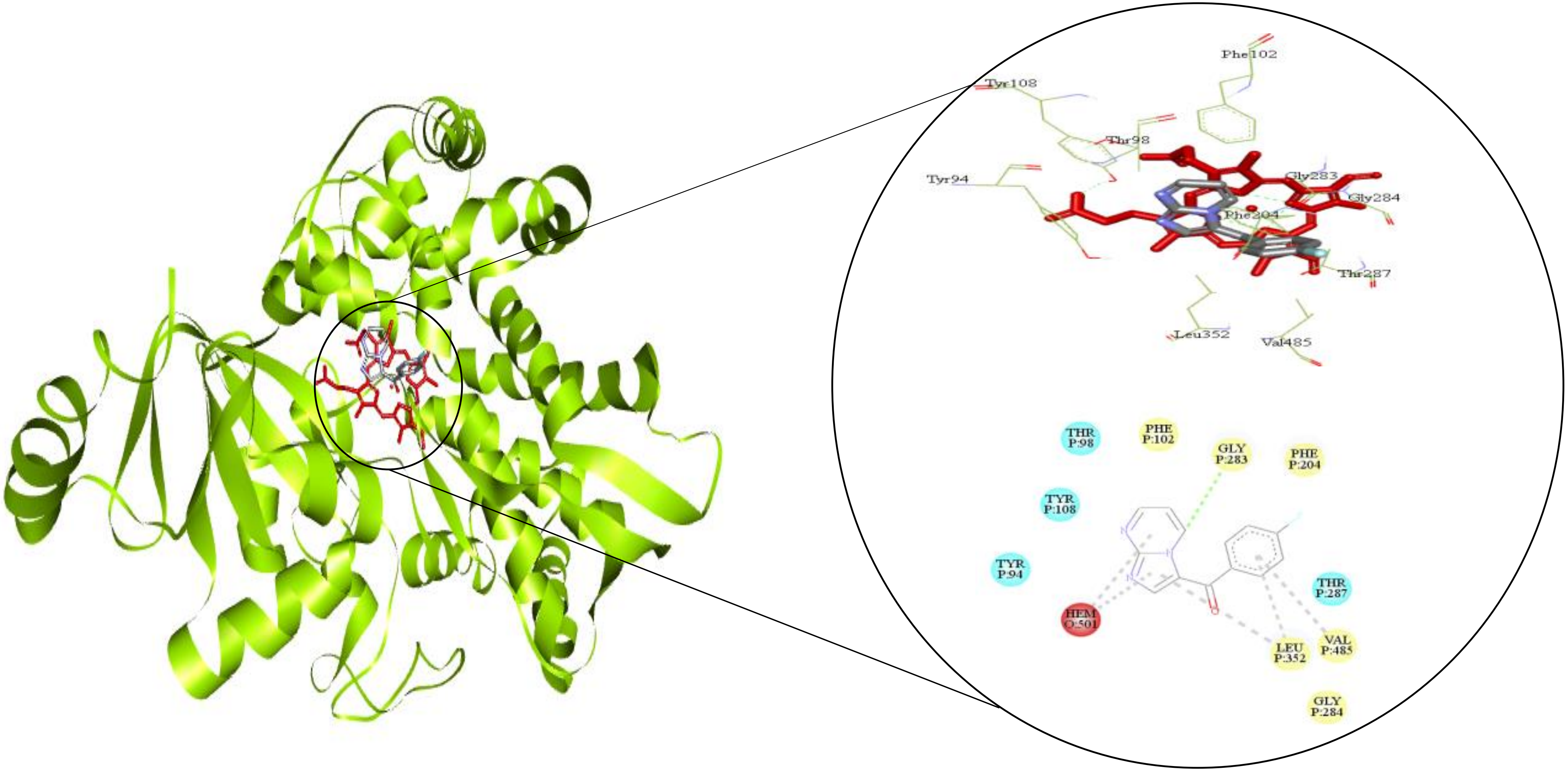

Figure S68: Schematic representation of the interactions of 4j with $CYP51_{Ca.}$

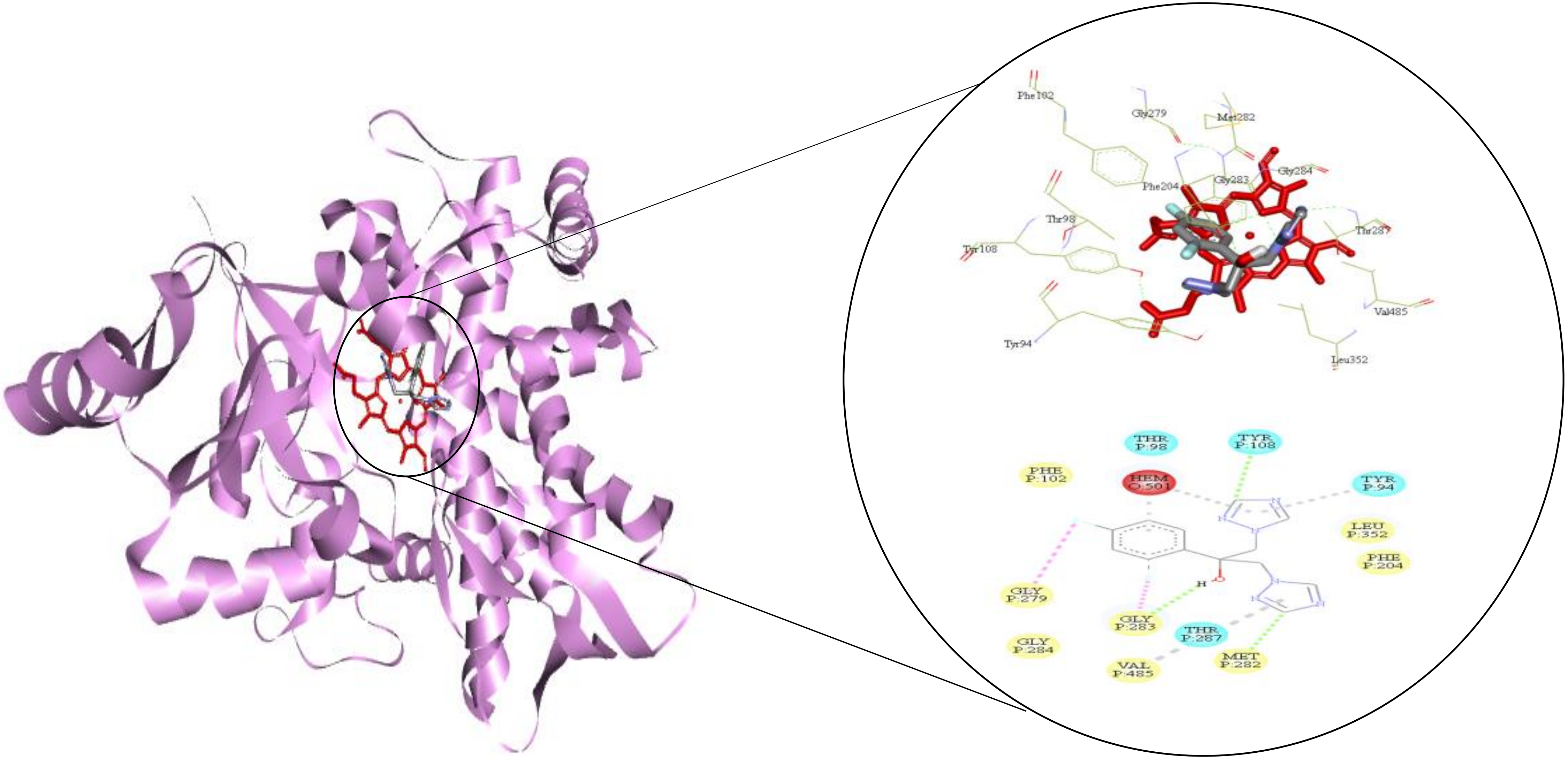

Figure S69: Schematic representation of the interactions of fluconazole with $CYP51_{Cd.}$

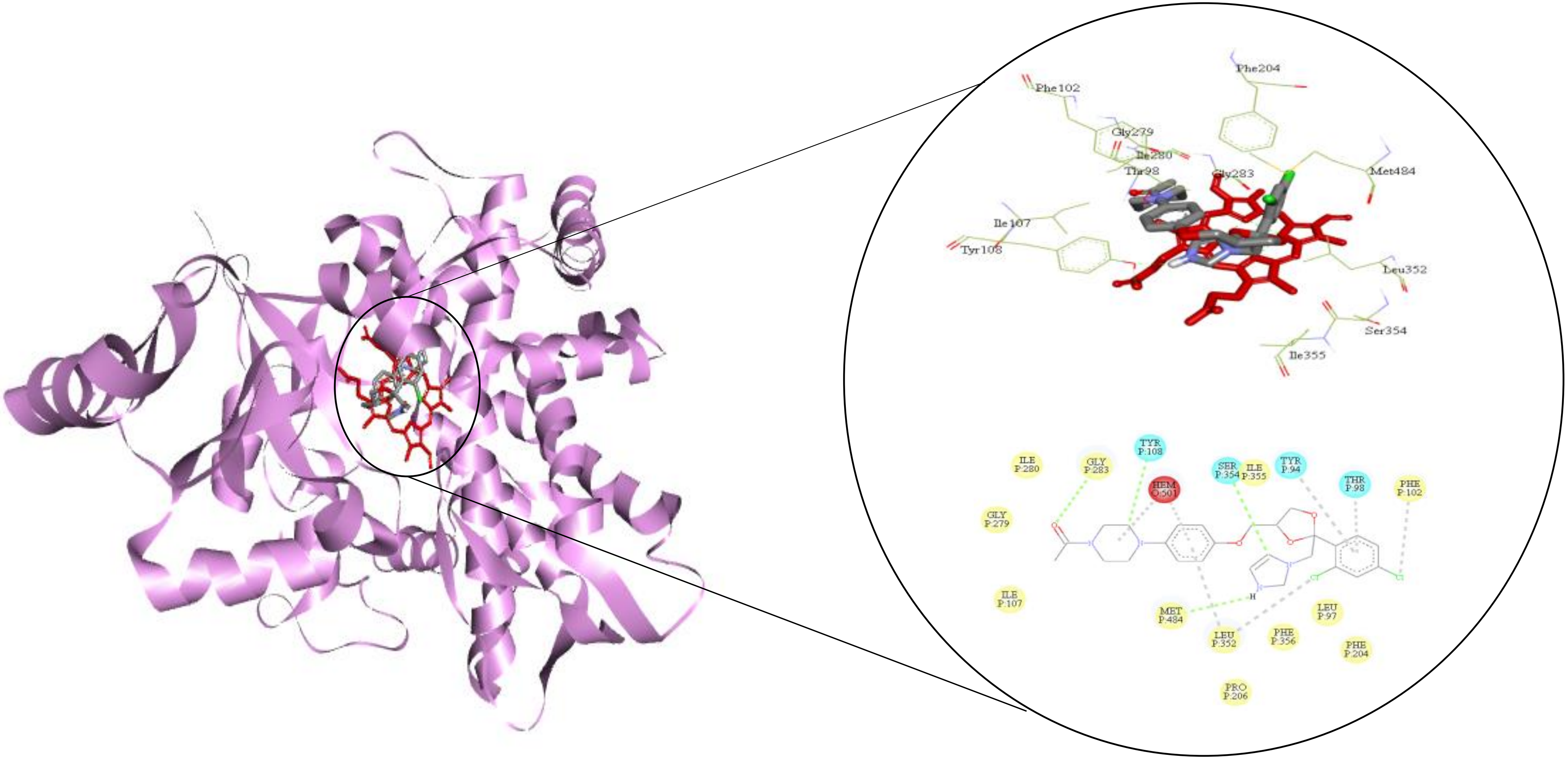

Figure S70: Schematic representation of the interactions of ketoconazole with $CYP51_{Cd.}$

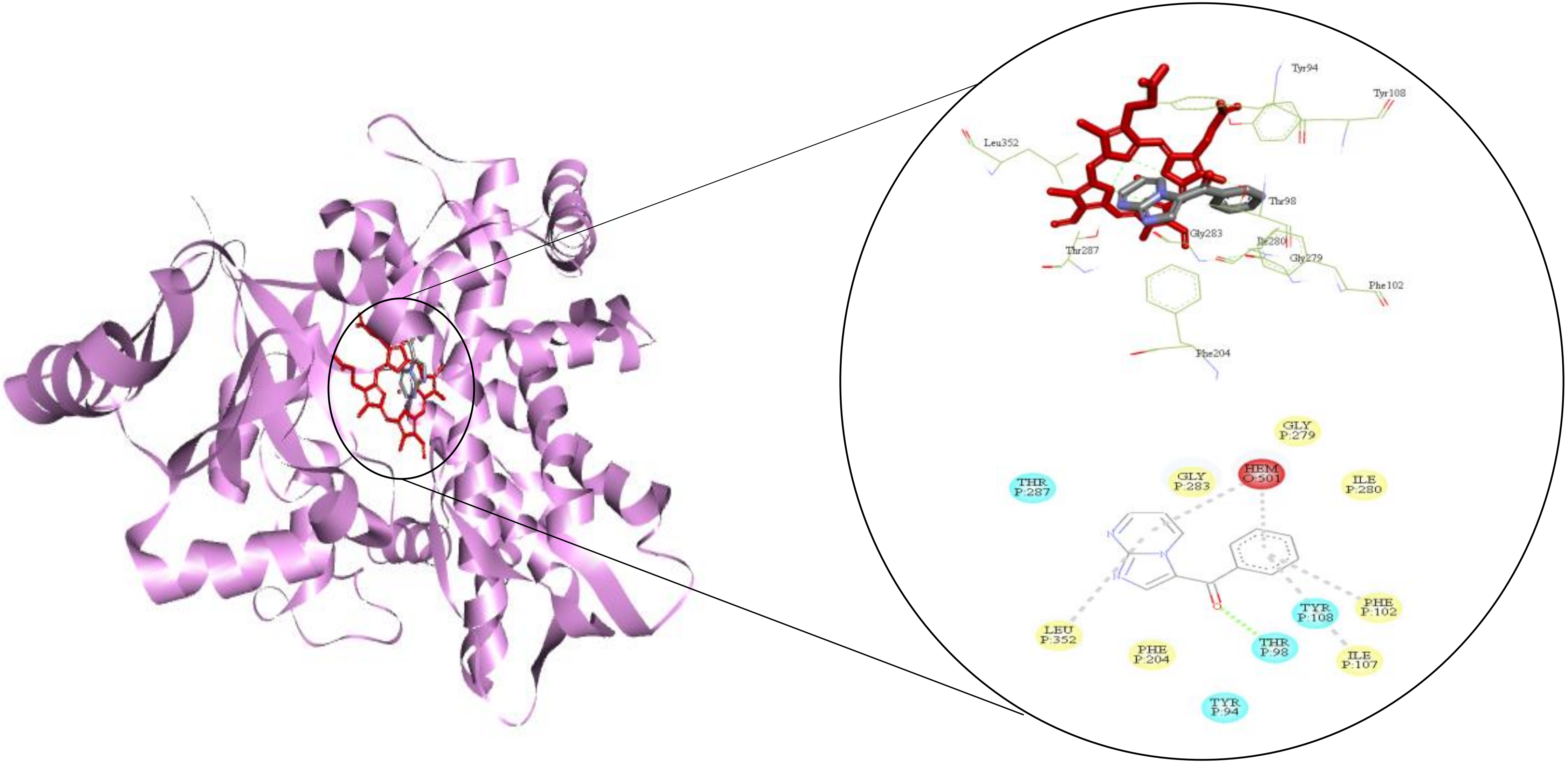

Figure S71: Schematic representation of the interactions of 4a with $CYP51_{Cd.}$

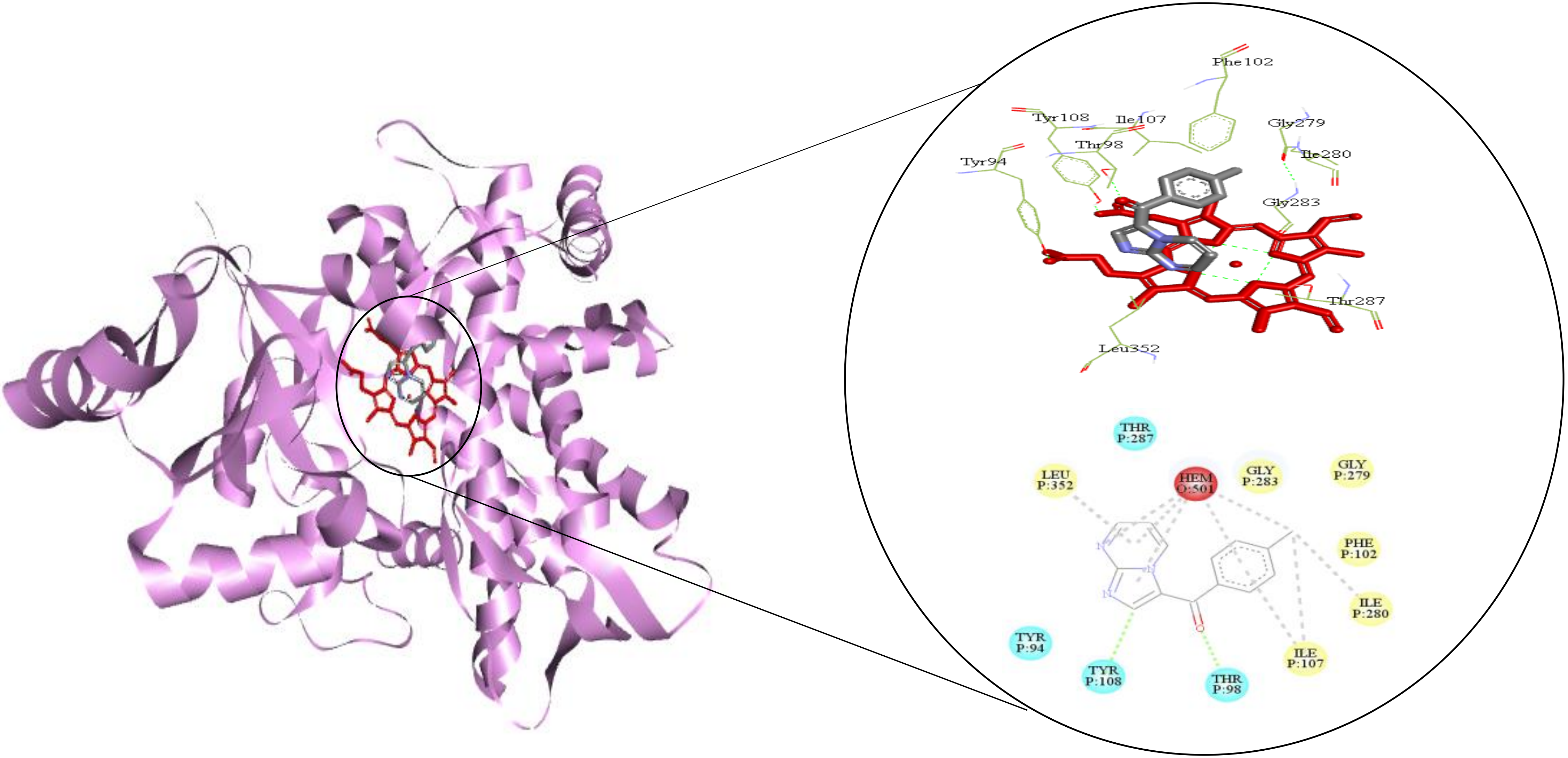

Figure S72: Schematic representation of the interactions of 4d with $CYP51_{Cd.}$

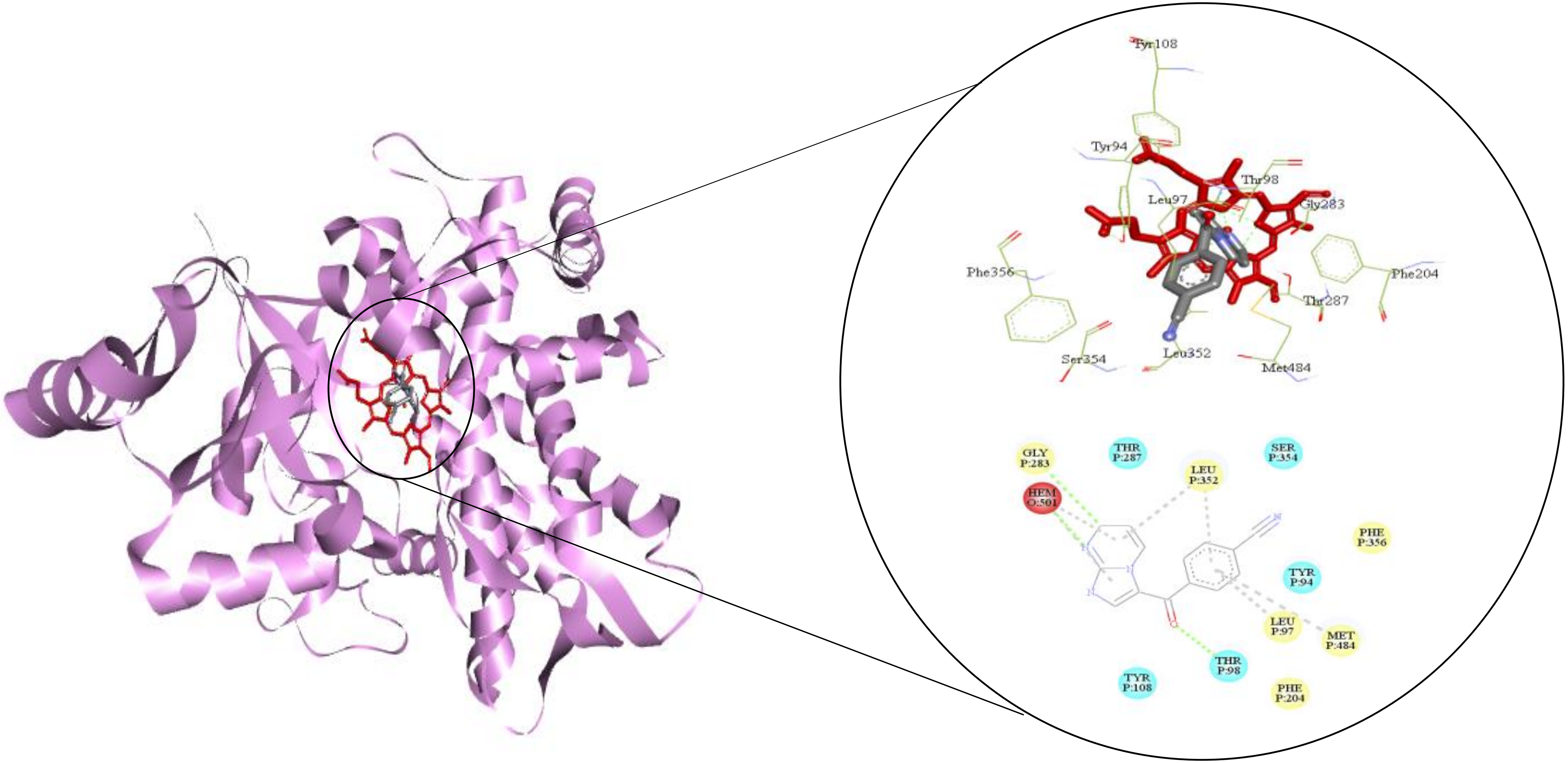

Figure S73: Schematic representation of the interactions of 4f with $CYP51_{Cd.}$

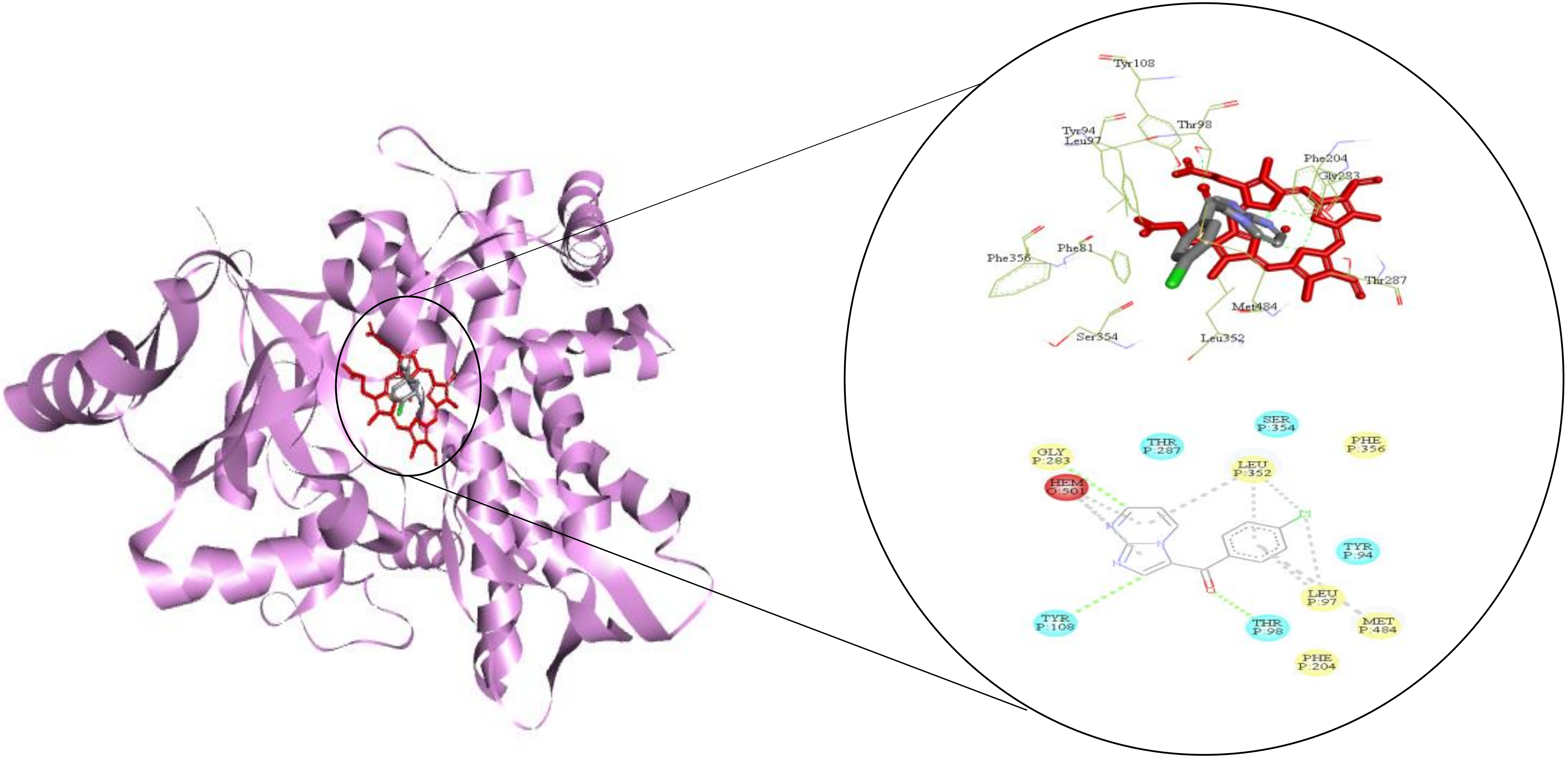

Figure S74: Schematic representation of the interactions of 4i with $CYP51_{Cd.}$

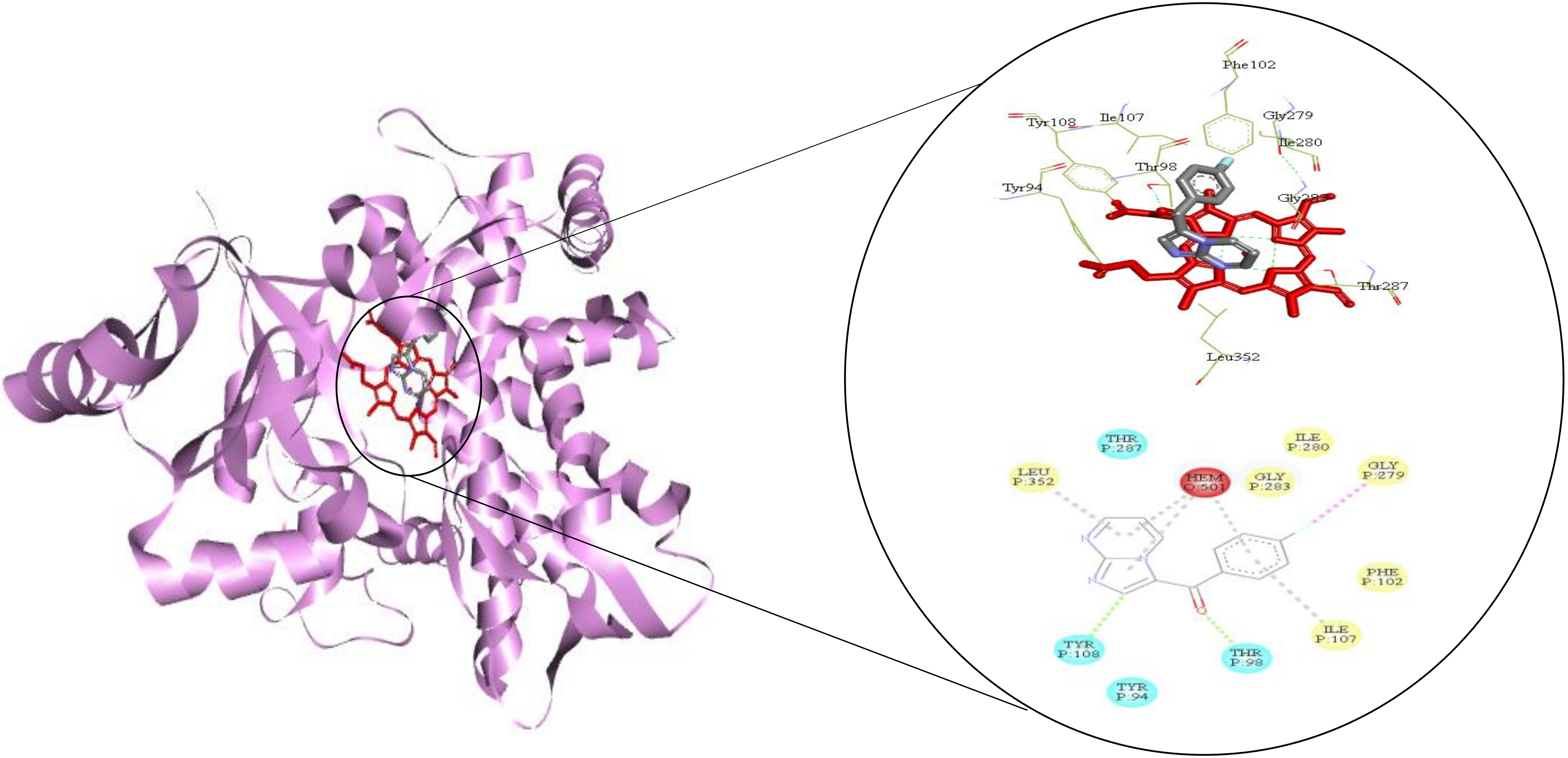

Figure S75: Schematic representation of the interactions of 4j with $CYP51_{Cd.}$

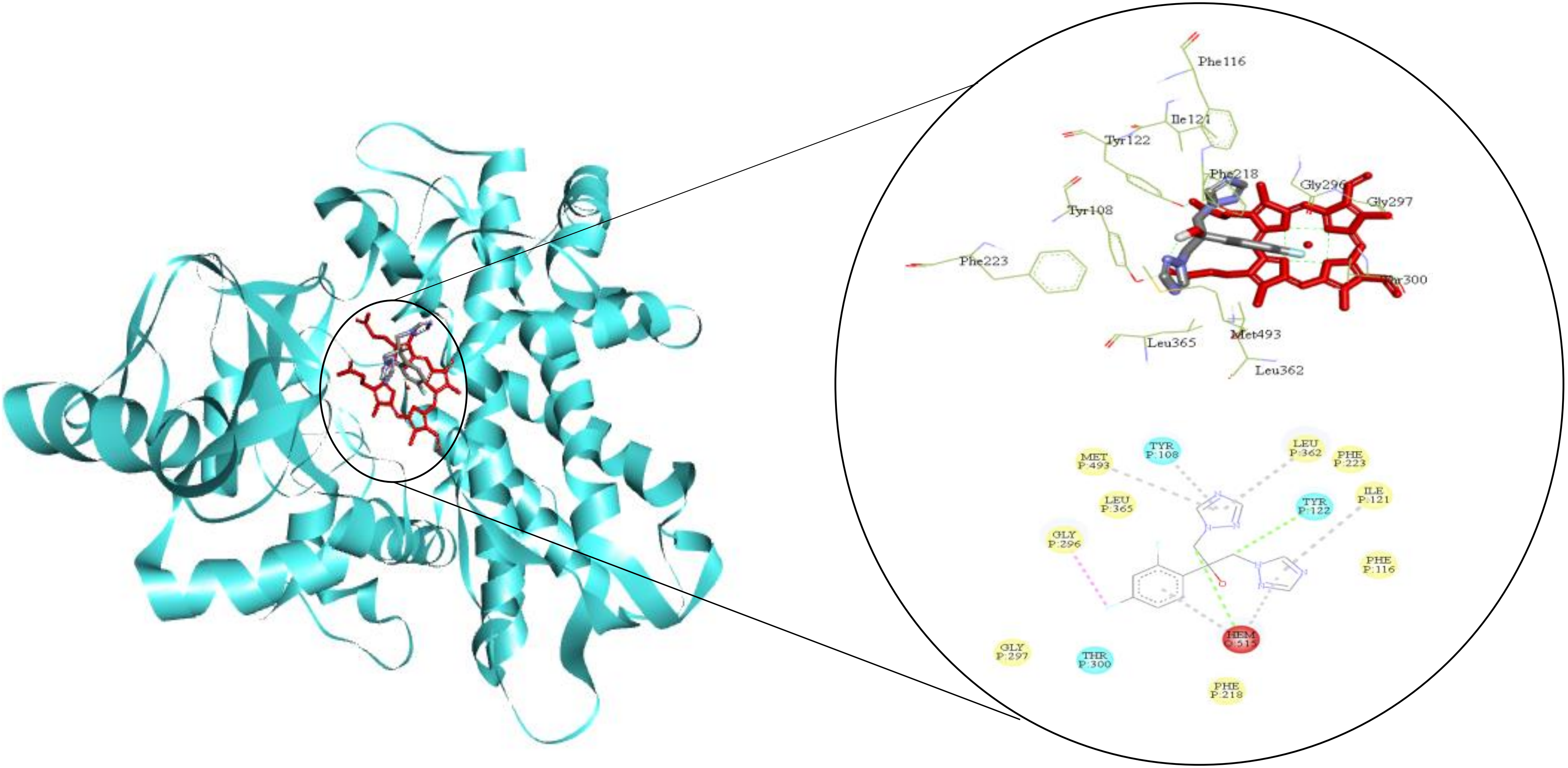

Figure S76: Schematic representation of the interactions of fluconazole with $CYP51_{Cg.}$

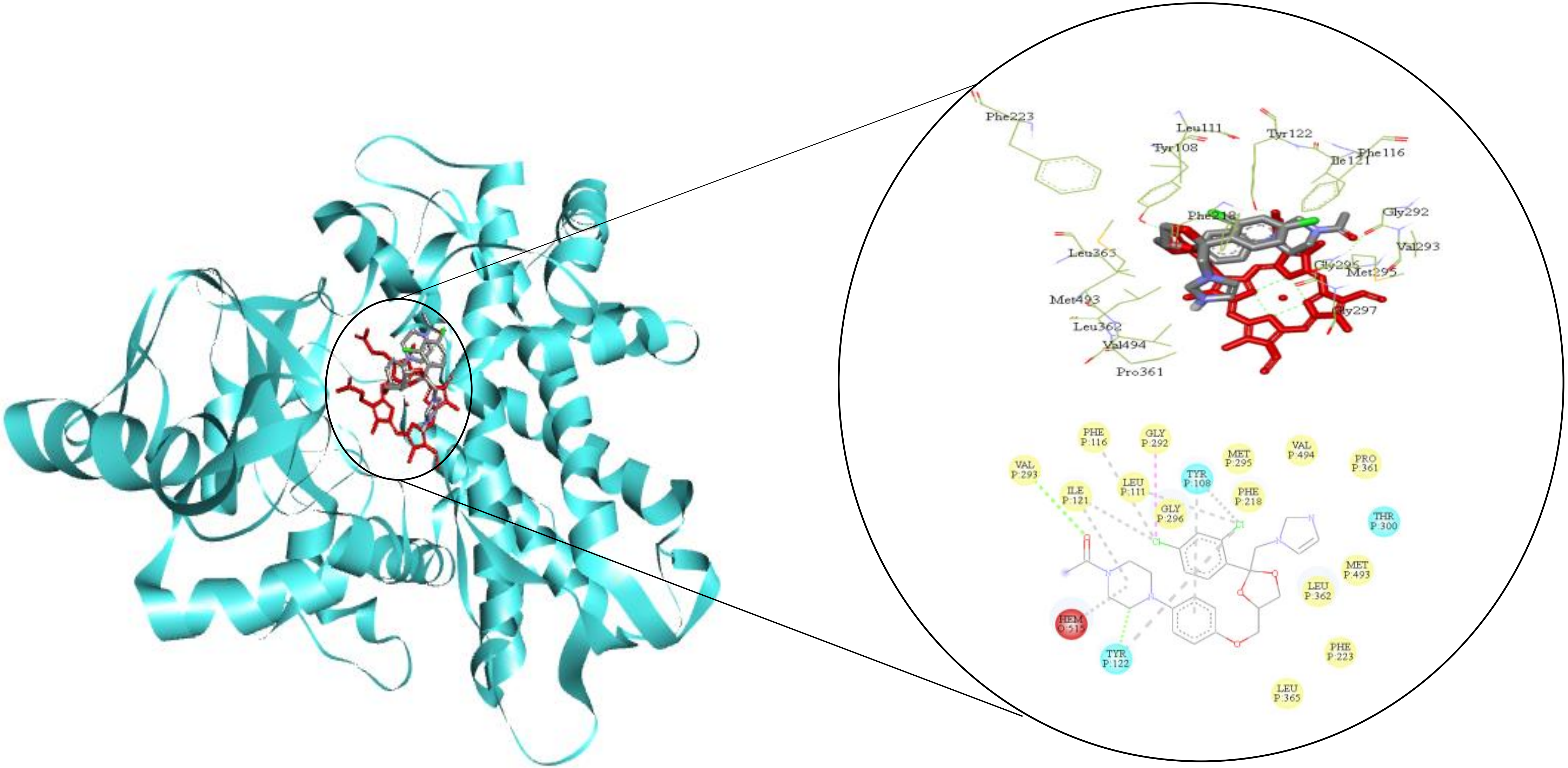

Figure S77: Schematic representation of the interactions of ketoconazole with $CYP51_{Cg.}$

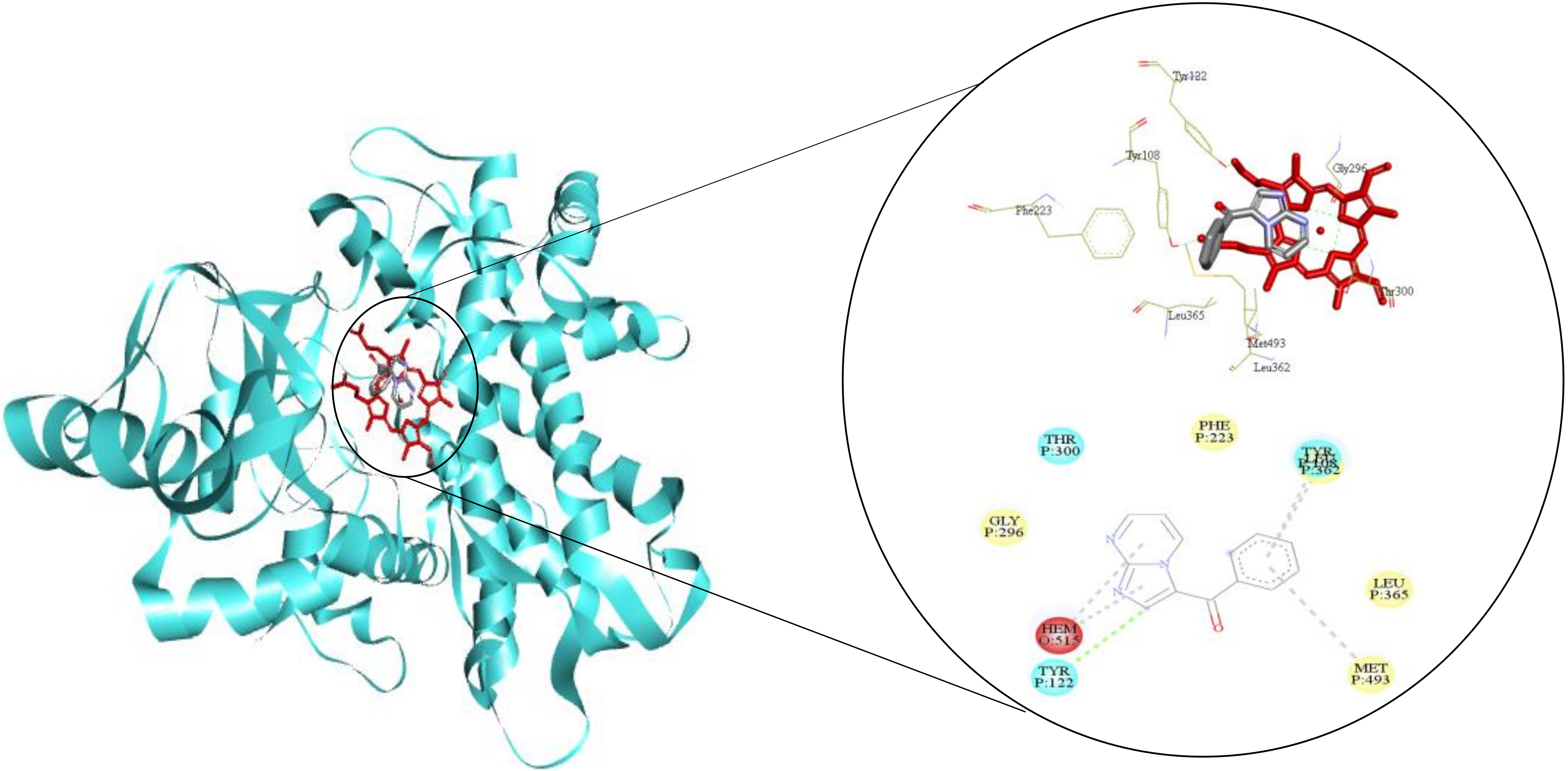

Figure S78: Schematic representation of the interactions of 4a with $CYP51_{Cg.}$

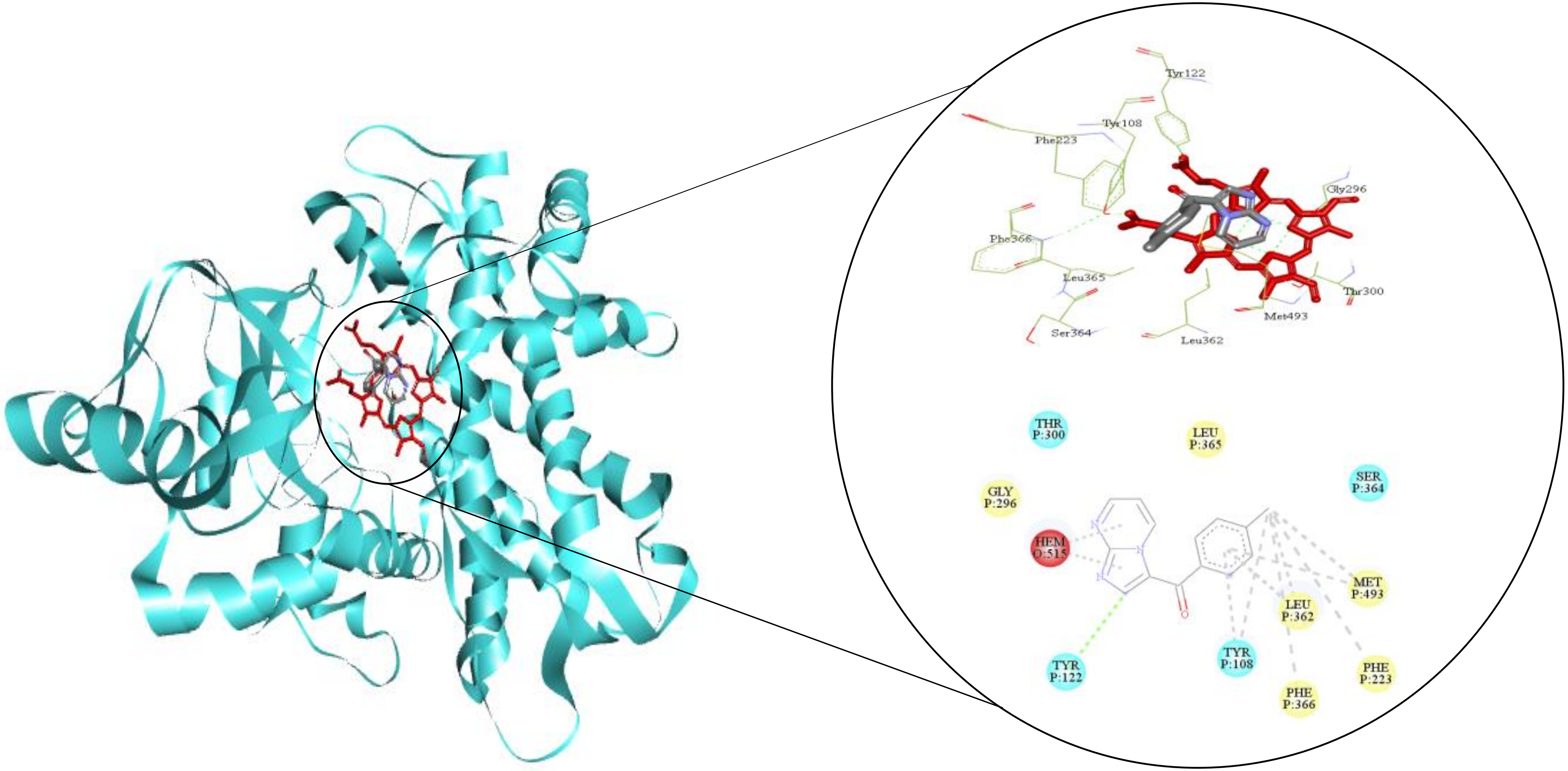

Figure S79: Schematic representation of the interactions of 4d with $CYP51_{Cg.}$

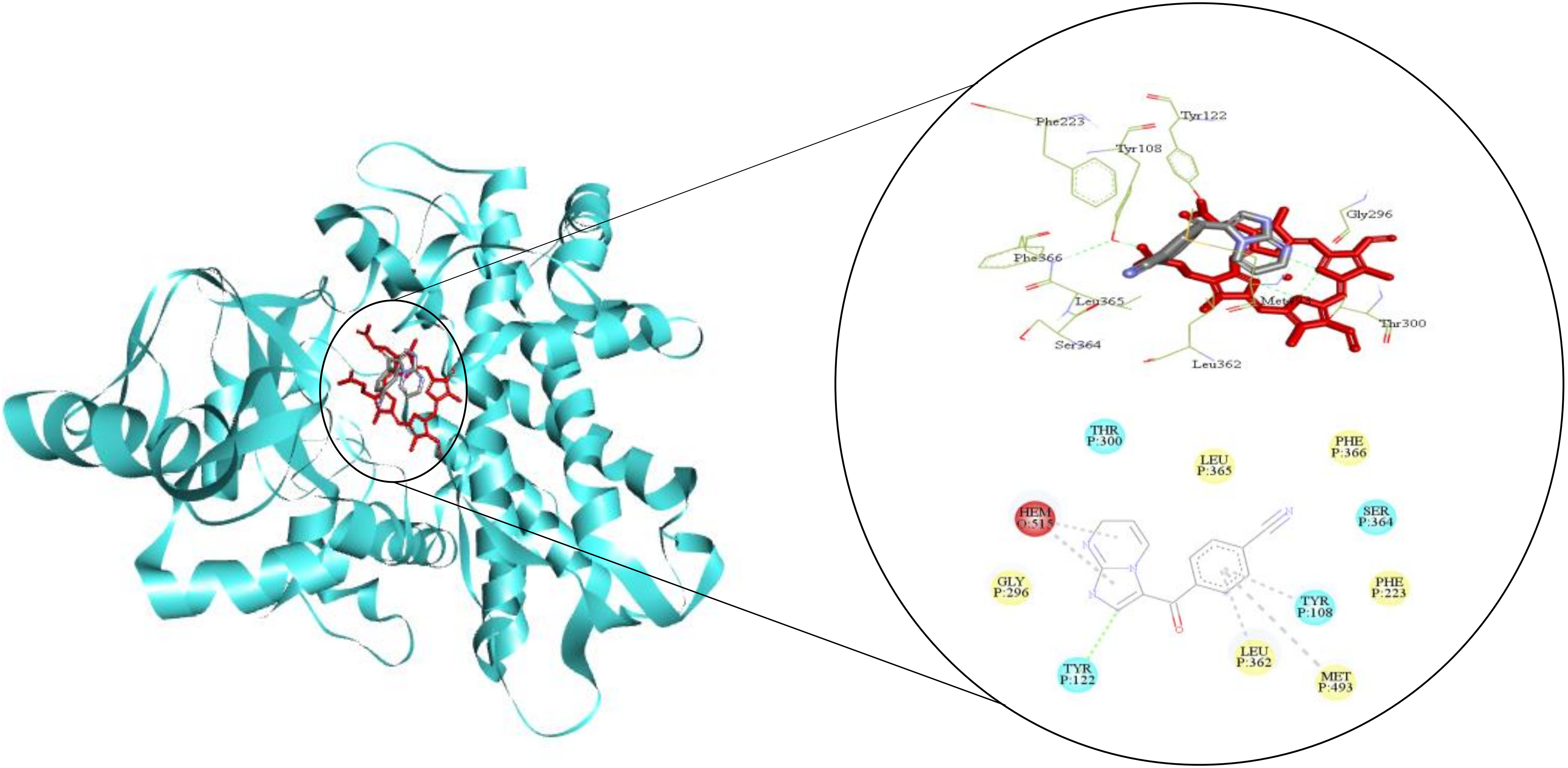

Figure S80: Schematic representation of the interactions of 4f with $CYP51_{Cg.}$

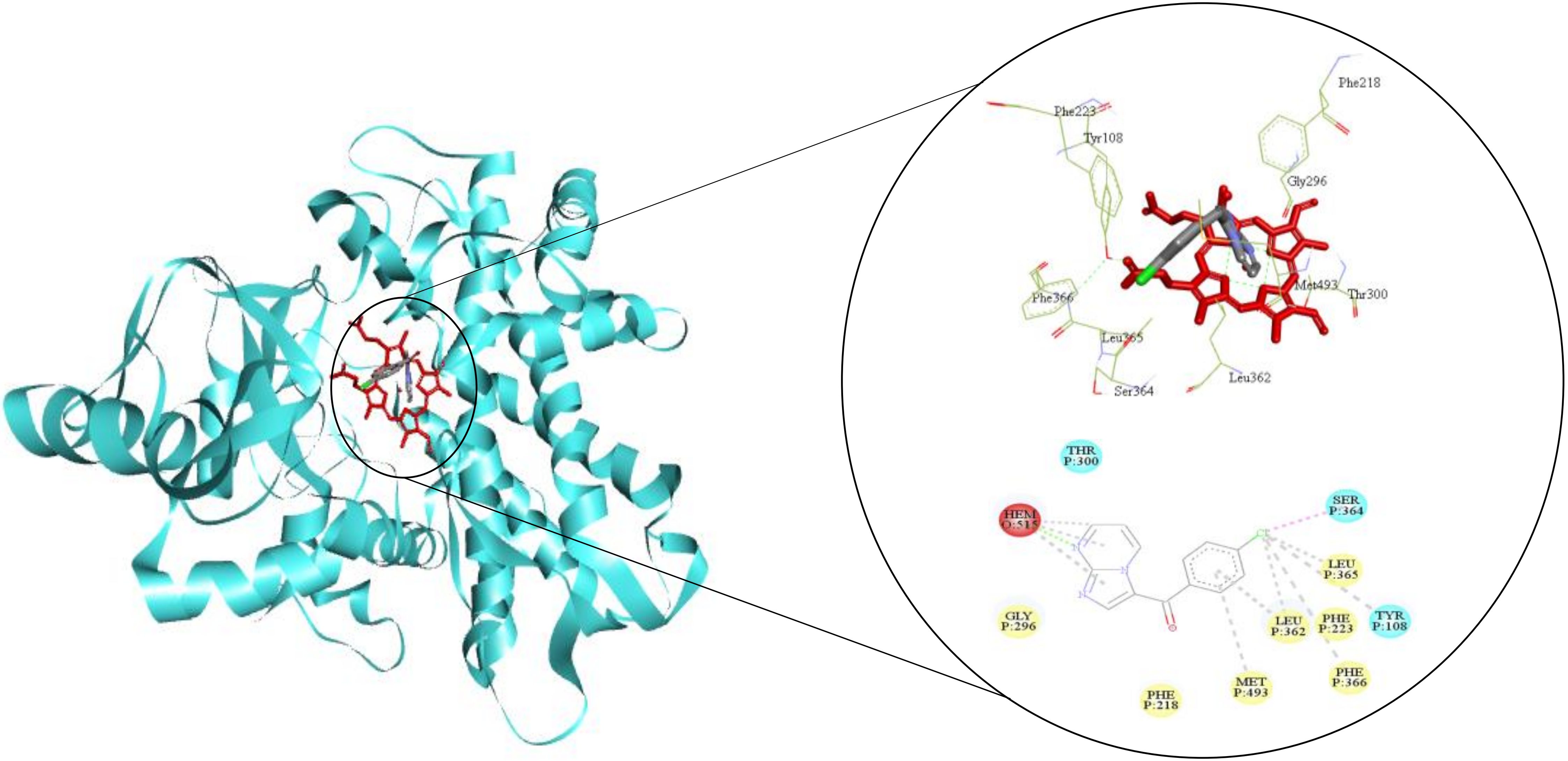

Figure S81: Schematic representation of the interactions of 4i with $CYP51_{Cg.}$

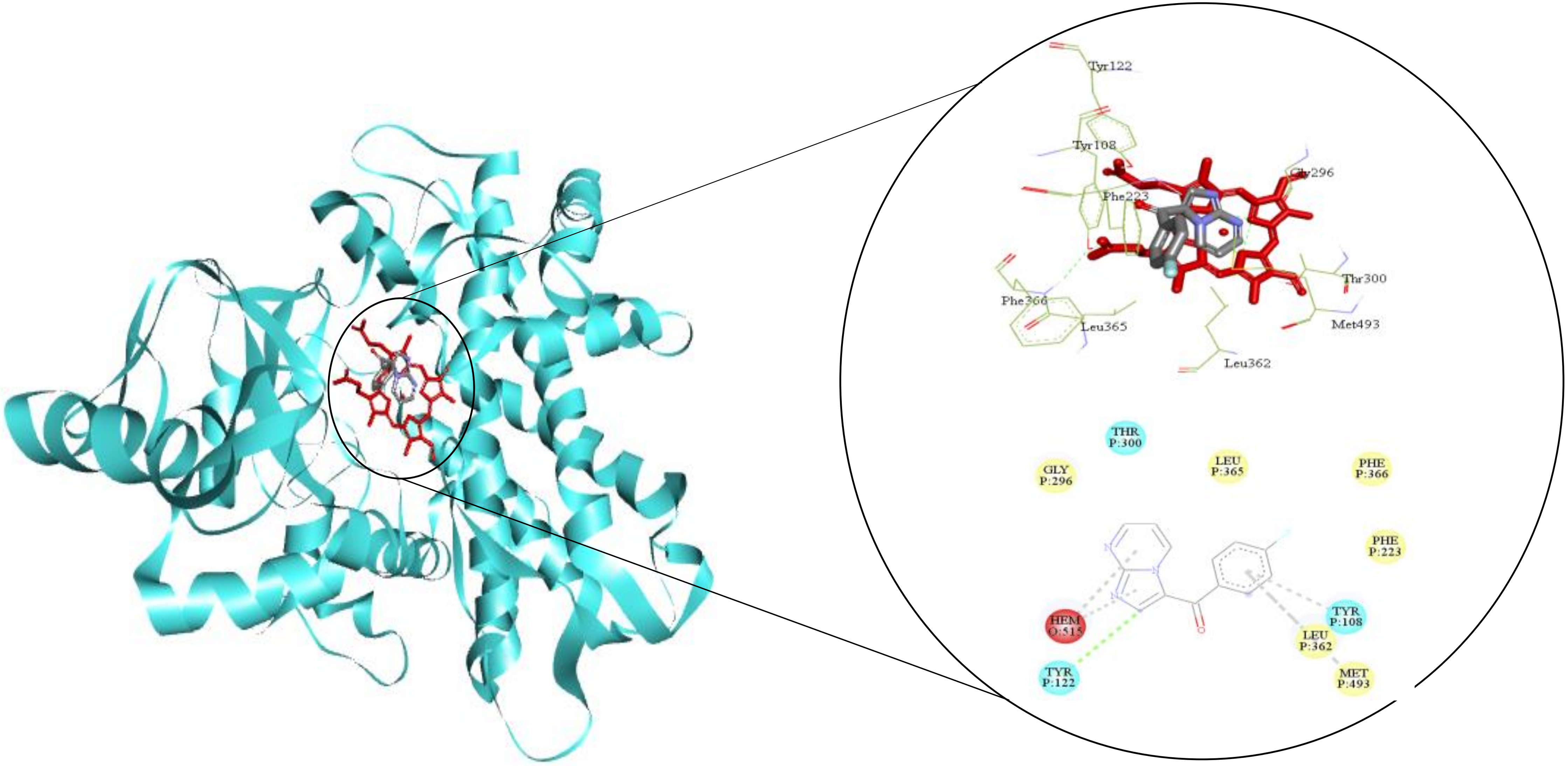

Figure S82: Schematic representation of the interactions of 4j with $CYP51_{Cg.}$

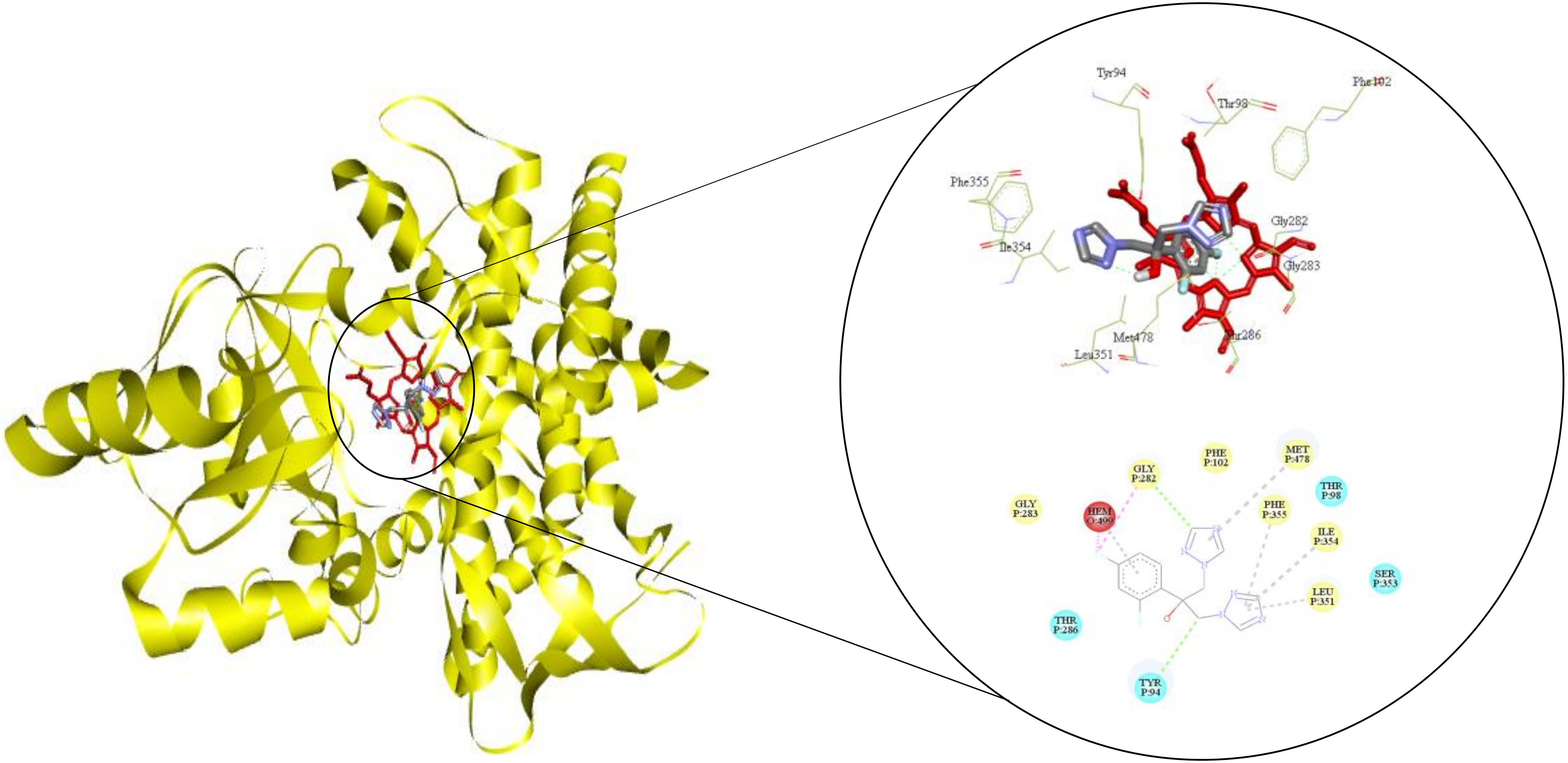

Figure S83: Schematic representation of the interactions of fluconazole with $CYP51_{Cgui.}$

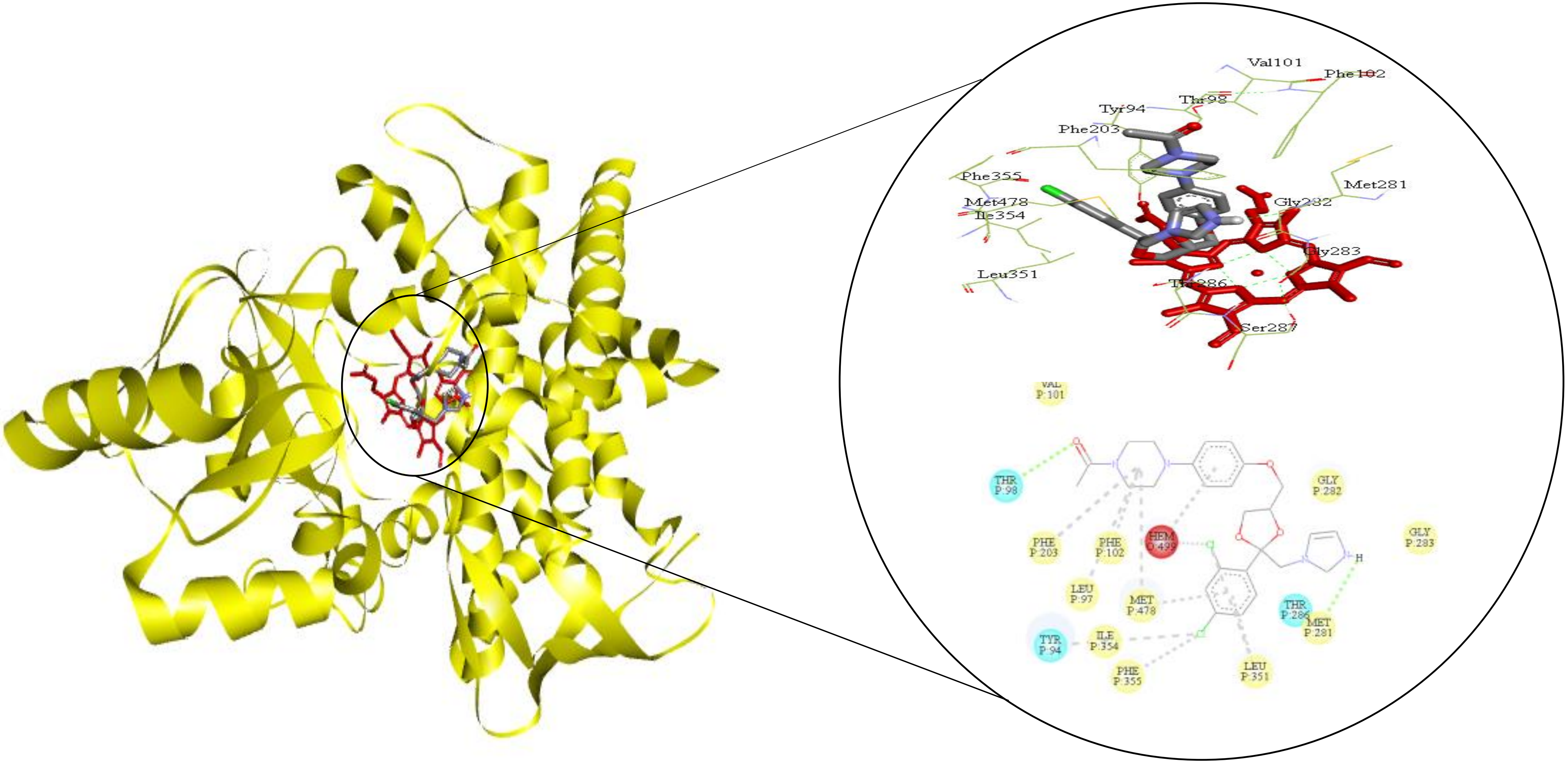

Figure S84: Schematic representation of the interactions of ketoconazole with $CYP51_{Cgui.}$

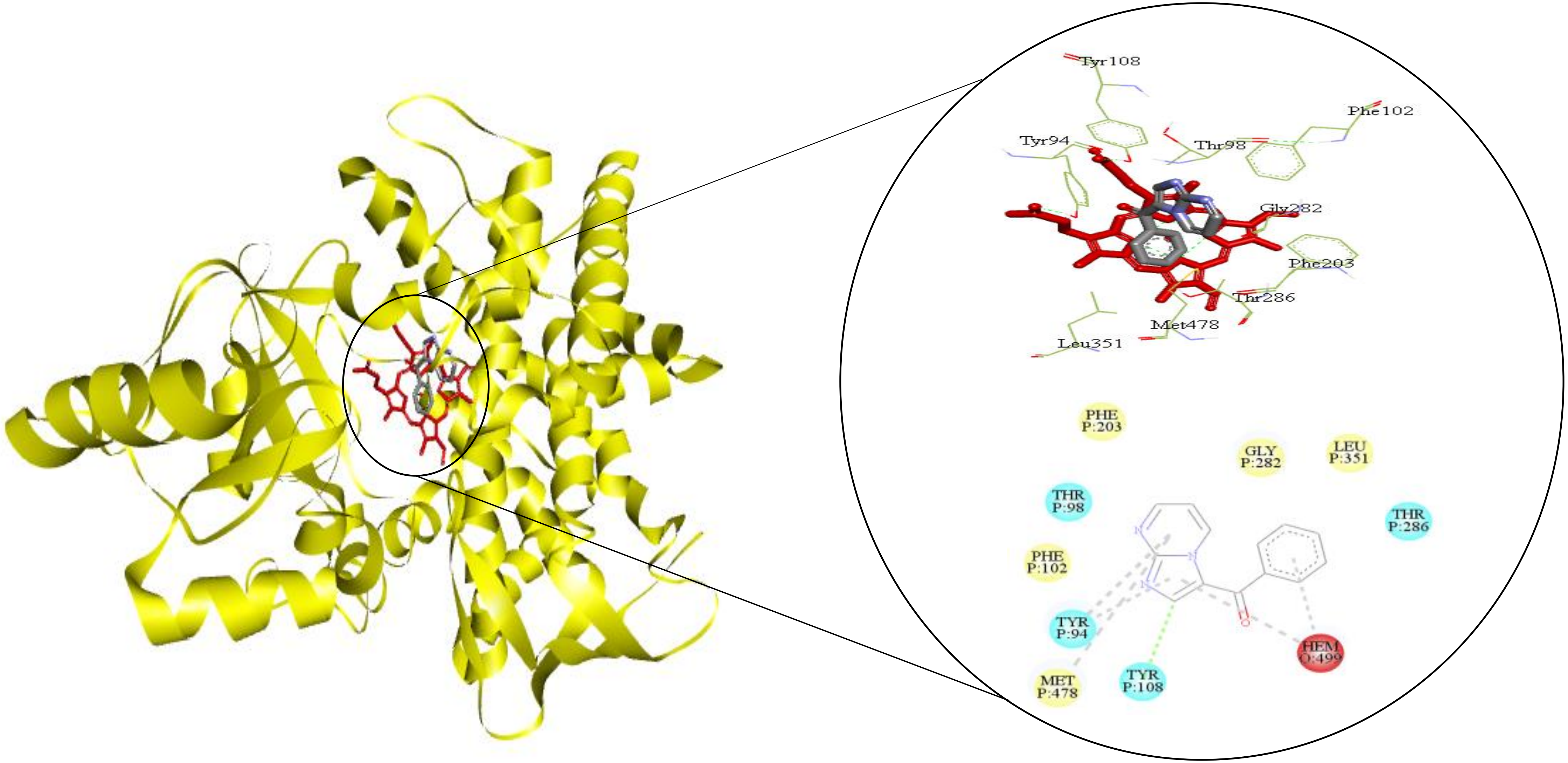

Figure S85: Schematic representation of the interactions of 4a with $CYP51_{Cgui.}$

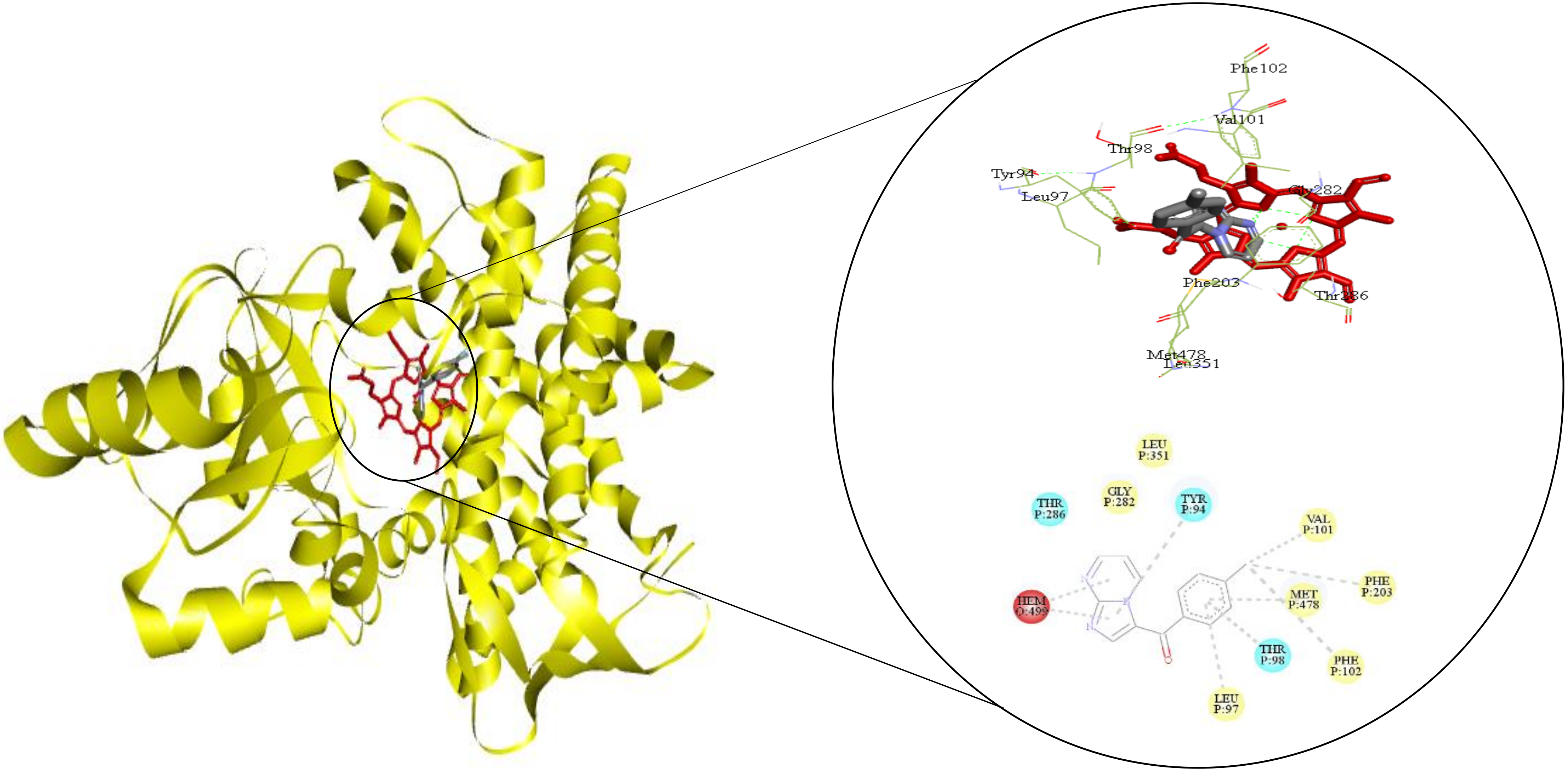

Figure S86: Schematic representation of the interactions of 4d with $CYP51_{Cgui.}$

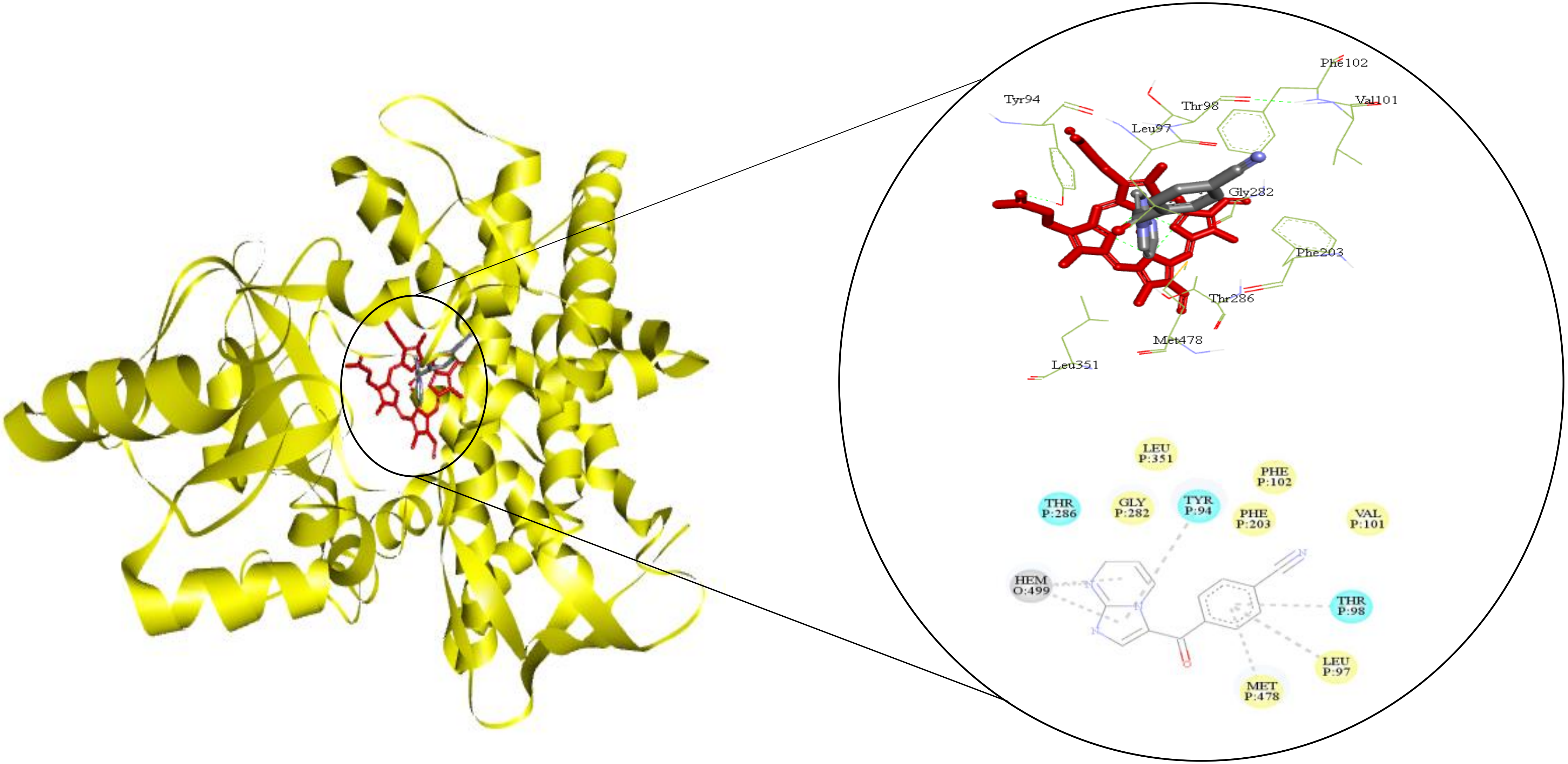

Figure S87: Schematic representation of the interactions of 4f with $CYP51_{Cgui.}$

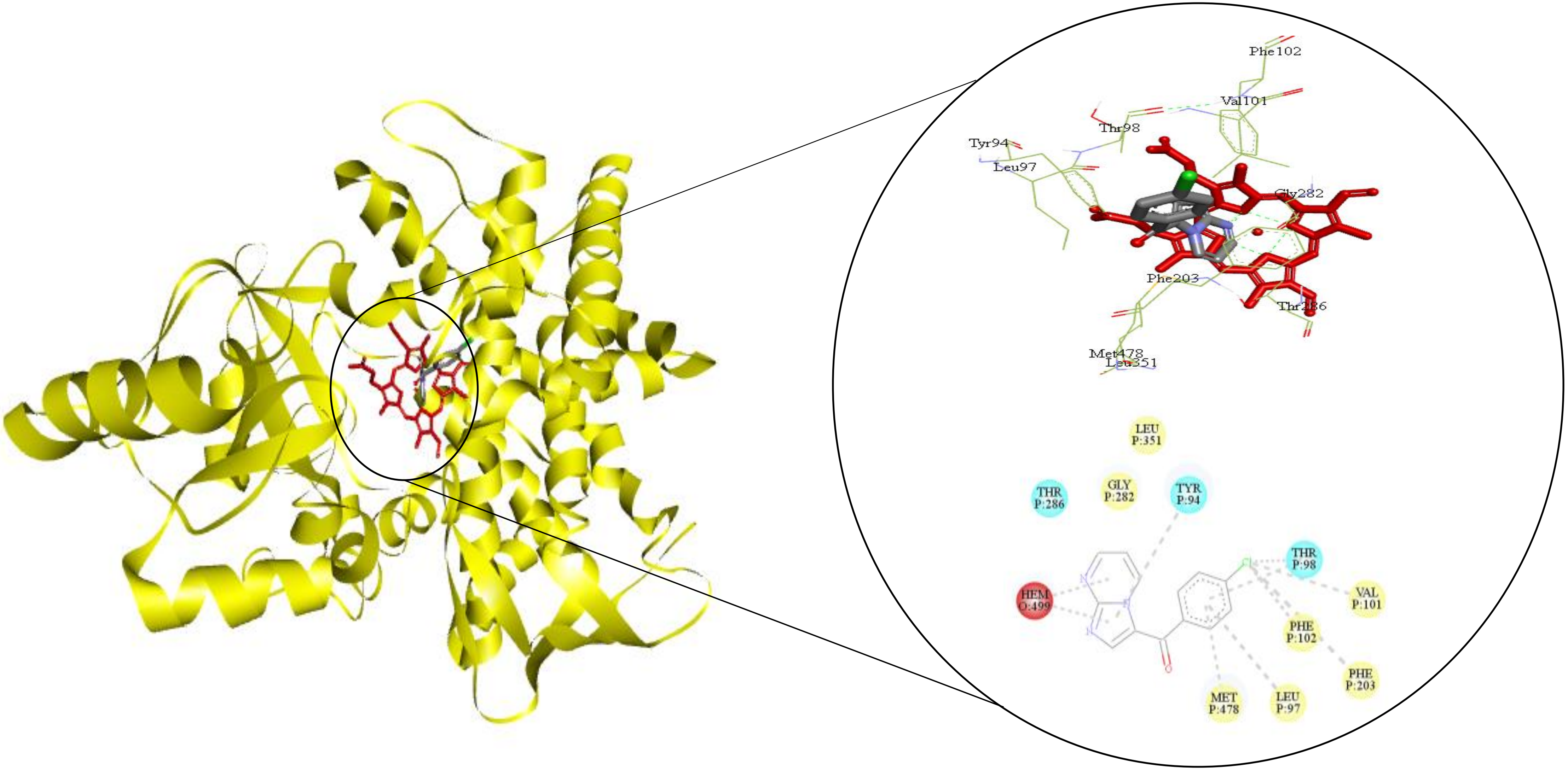

Figure S88: Schematic representation of the interactions of 4i with $CYP51_{Cgui.}$

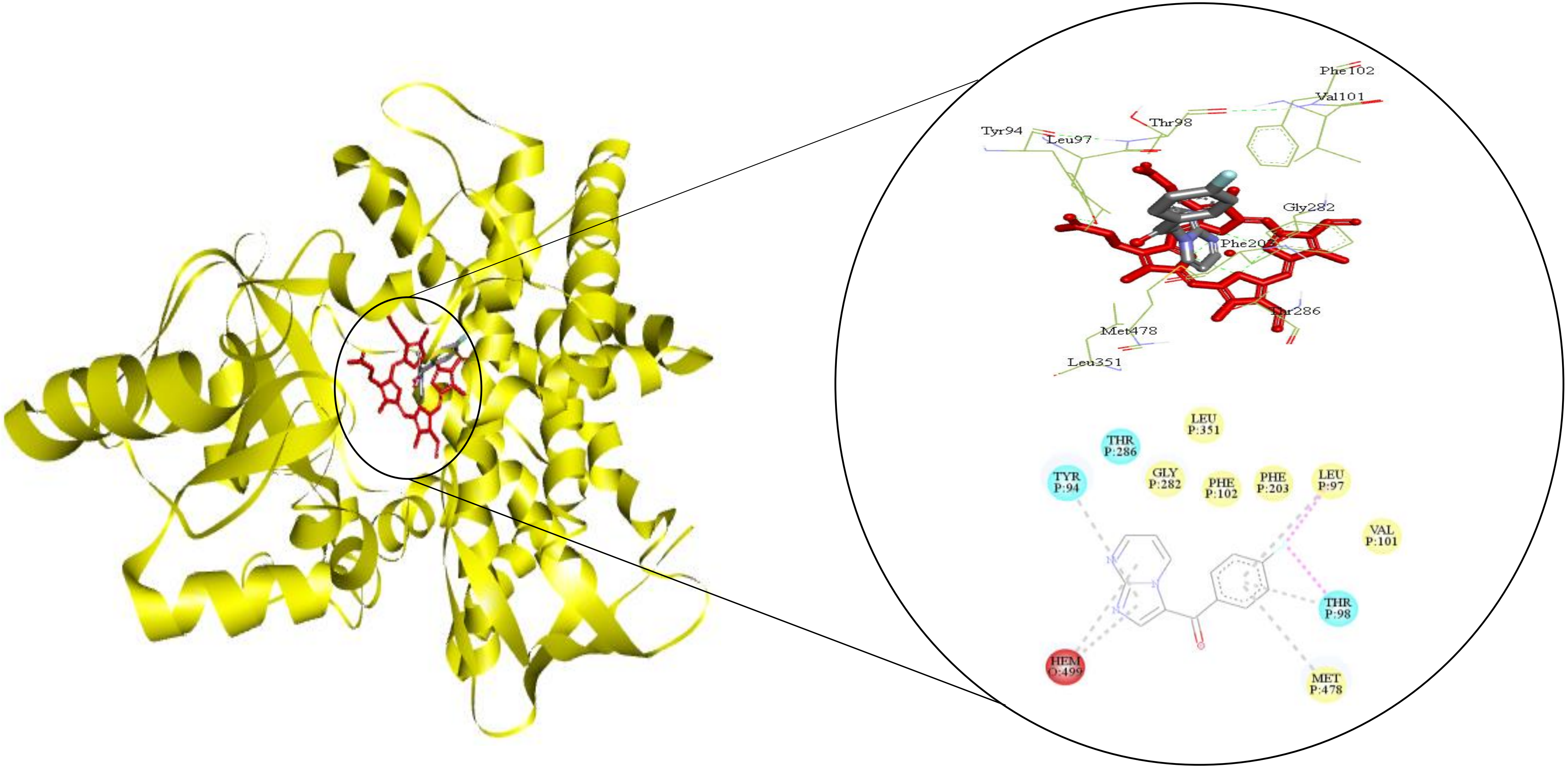

Figure S89: Schematic representation of the interactions of 4j with $CYP51_{Cgui.}$

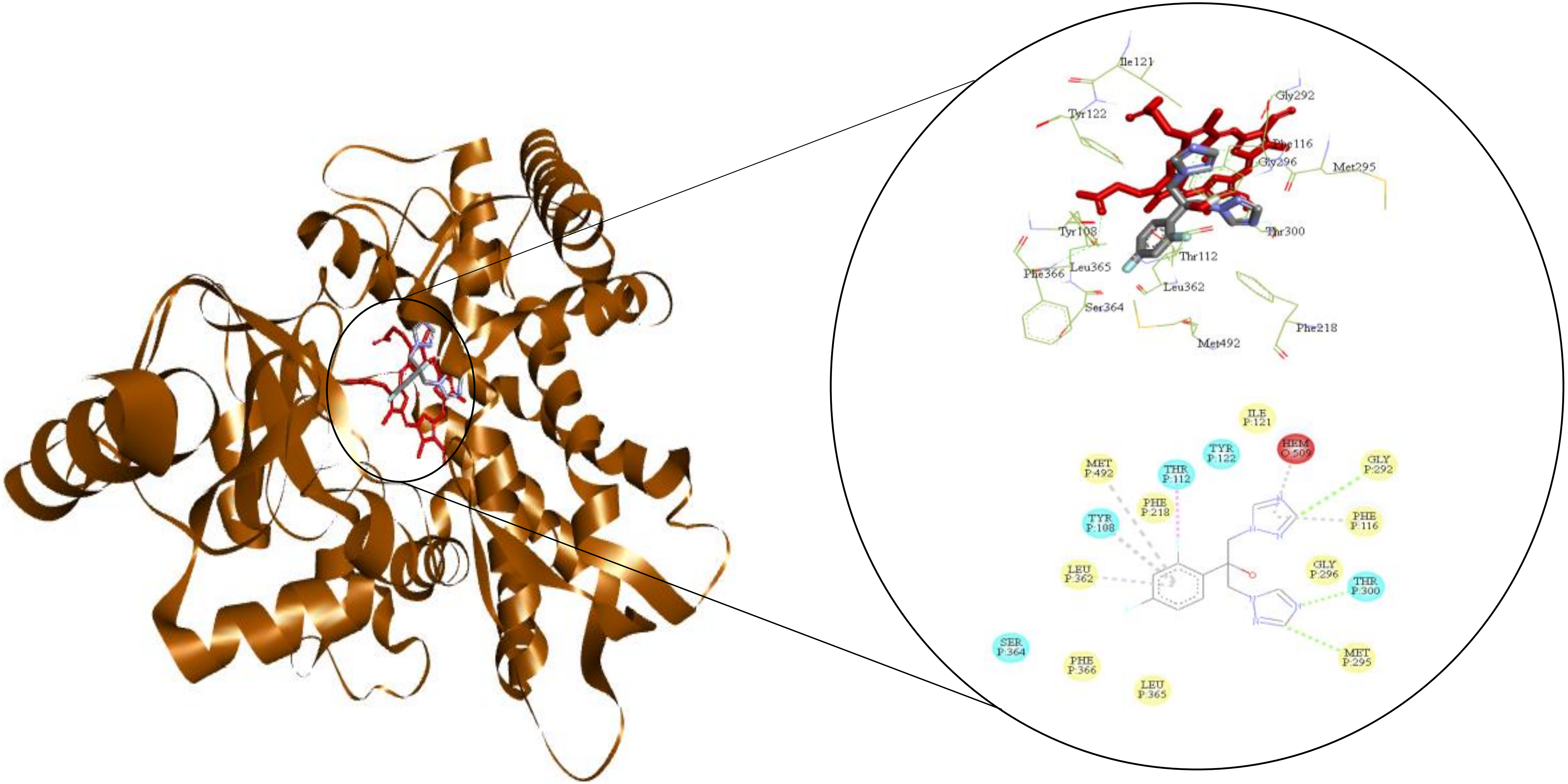

Figure S90: Schematic representation of the interactions of fluconazole with $CYP51_{Cke.}$

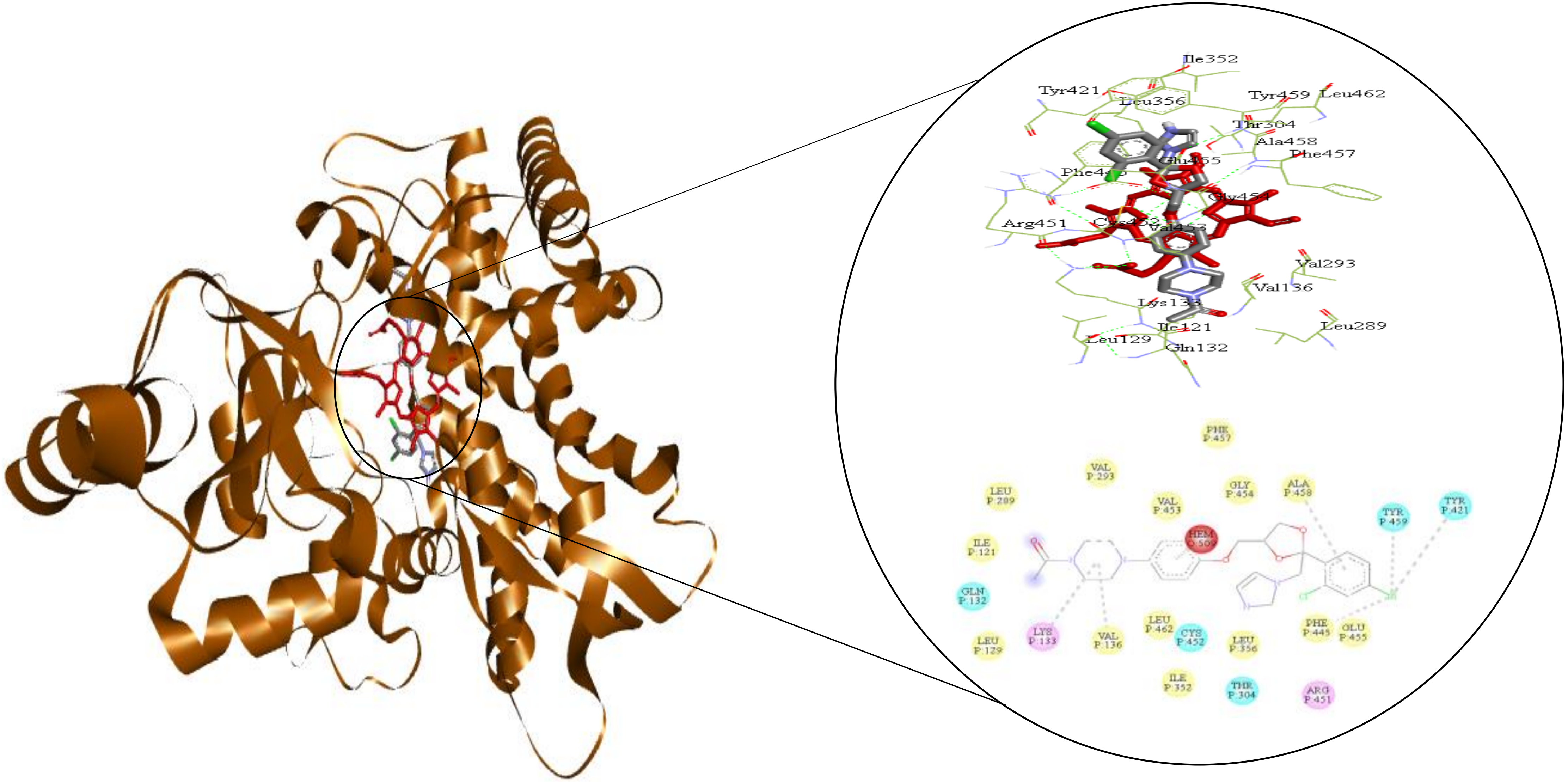

Figure S91: Schematic representation of the interactions of ketoconazole with $CYP51_{Cke}$.

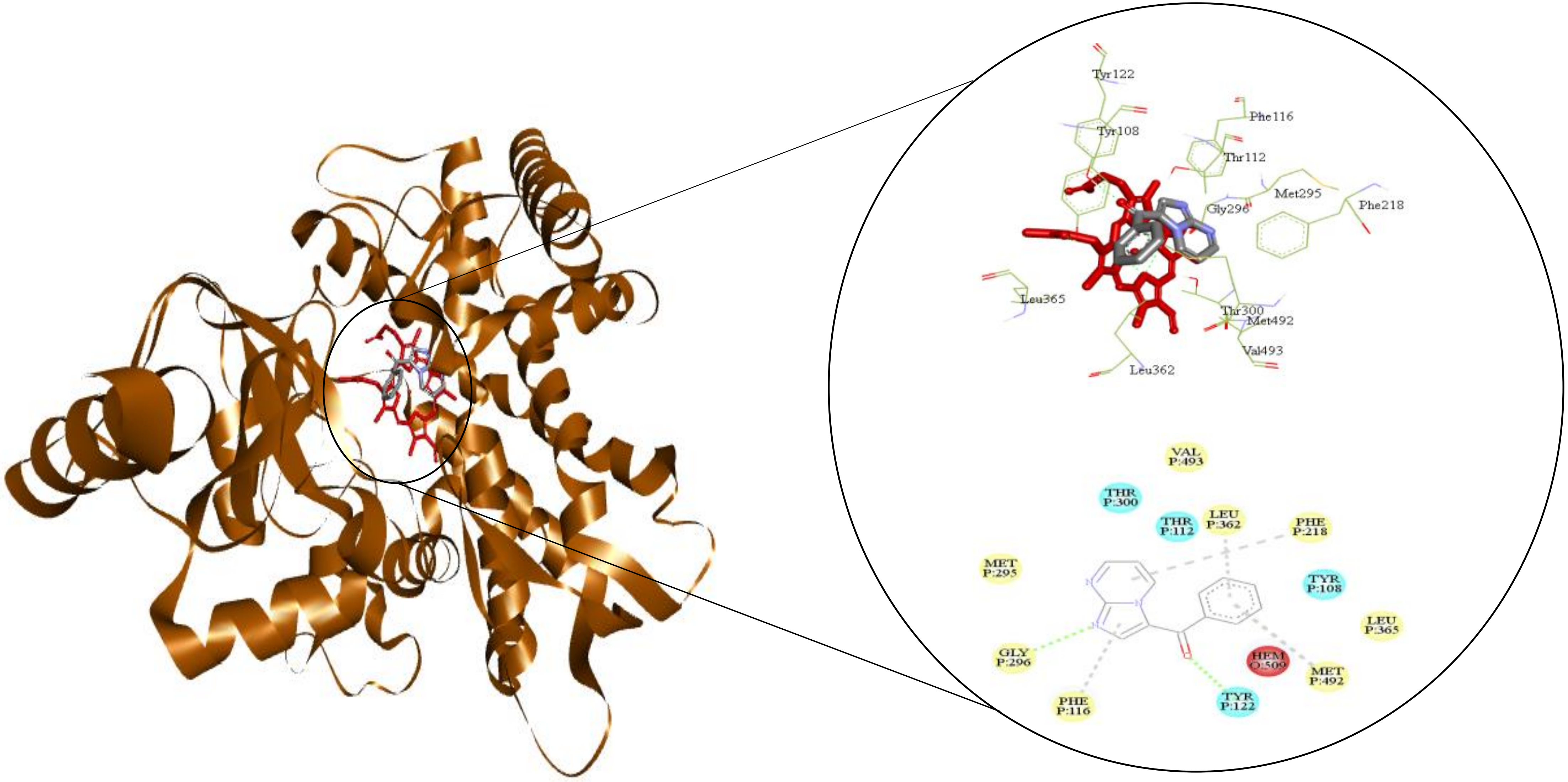

Figure S92: Schematic representation of the interactions of 4a with $CYP51_{Cke.}$

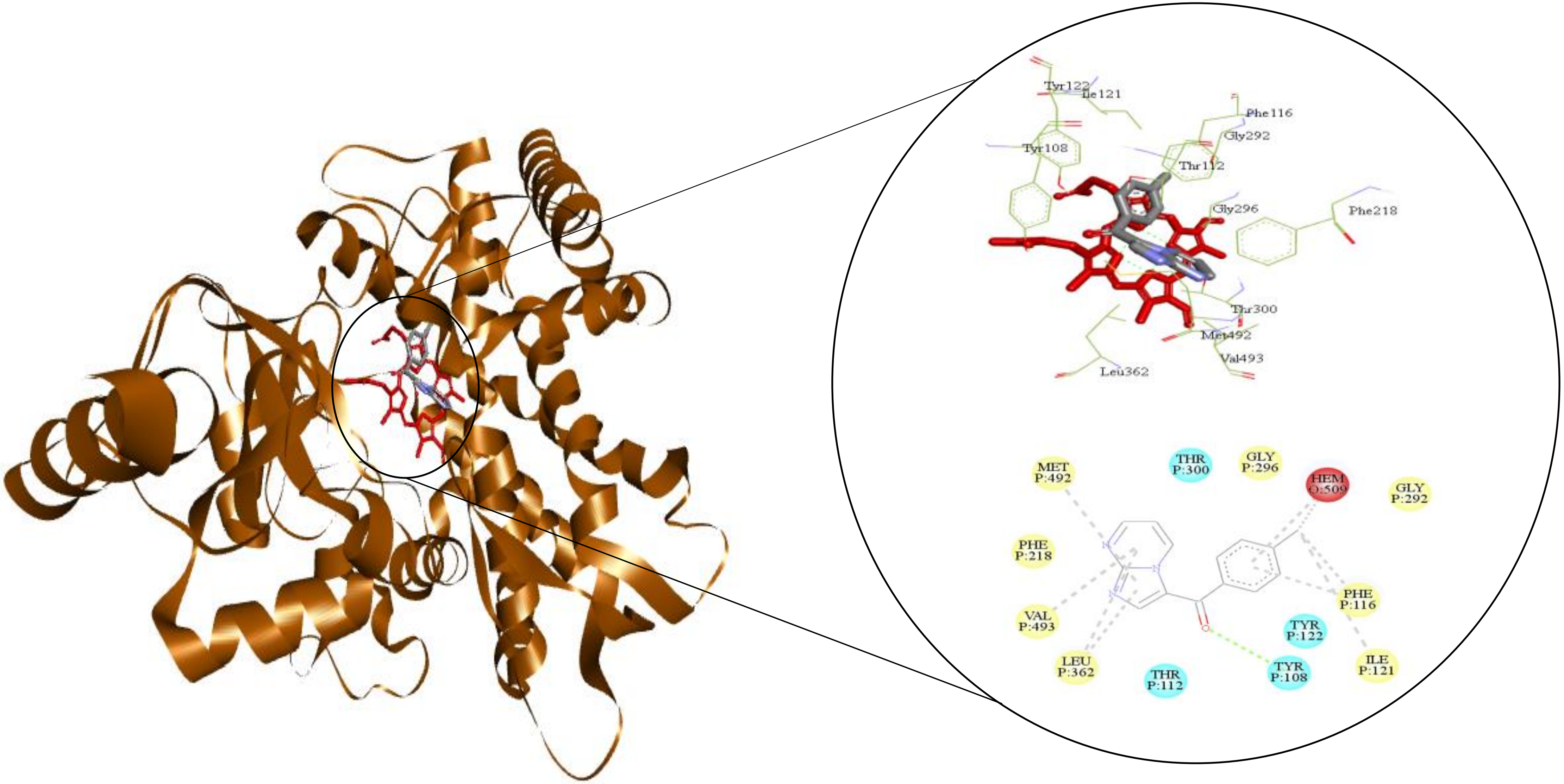

Figure S93: Schematic representation of the interactions of 4d with $CYP51_{Cke.}$

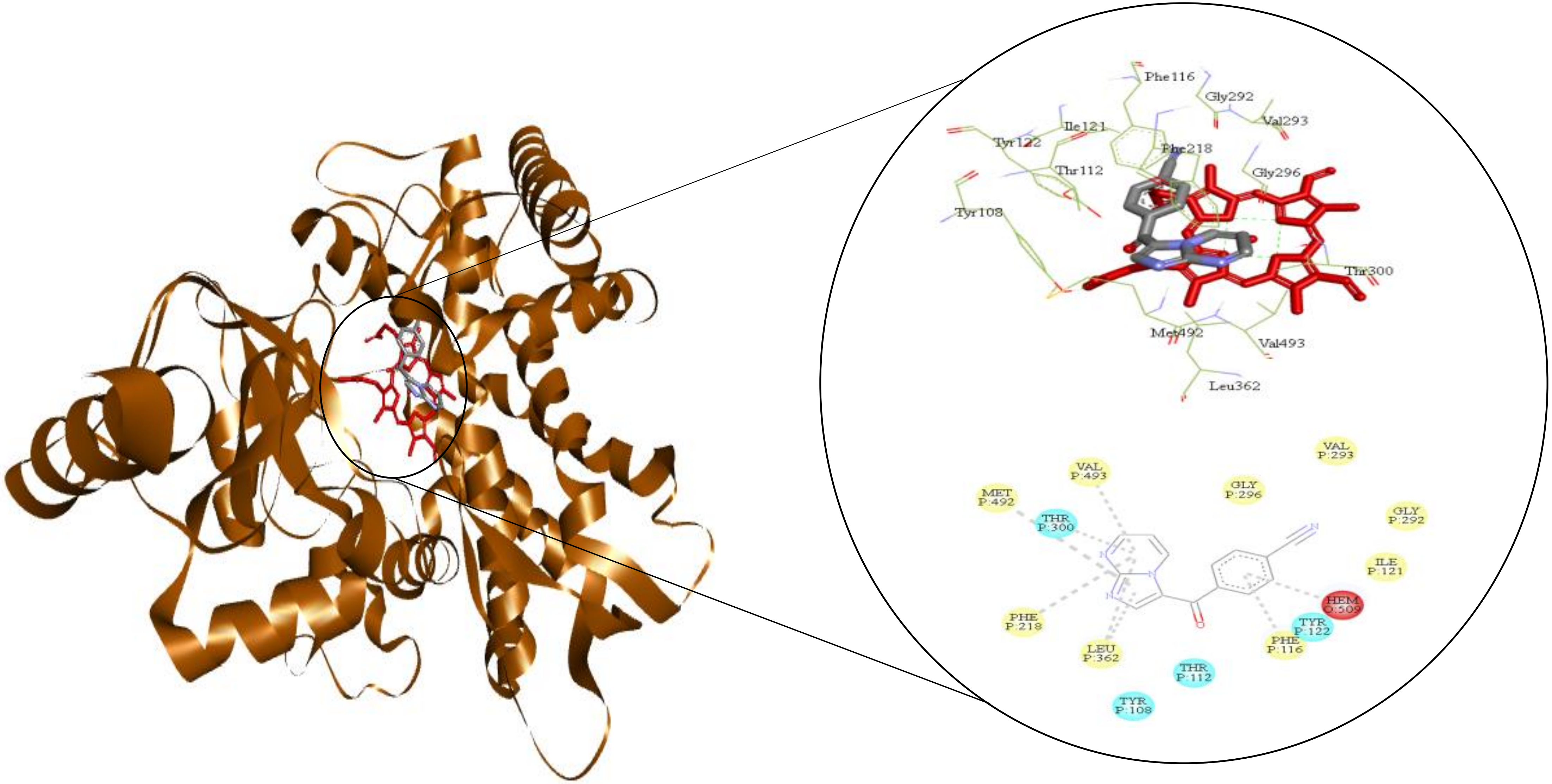

Figure S94: Schematic representation of the interactions of 4f with $CYP51_{Cke.}$

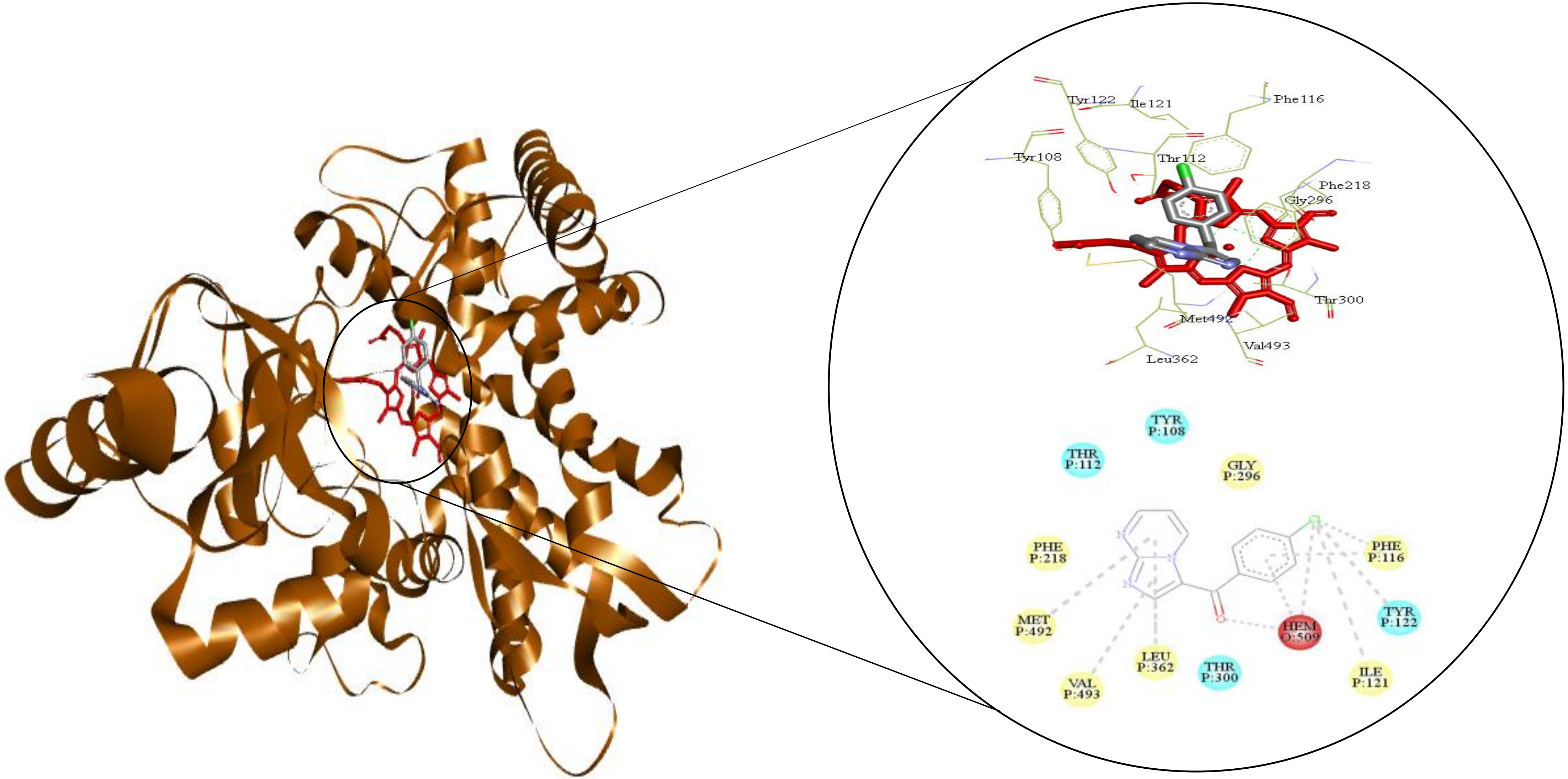

Figure S95: Schematic representation of the interactions of 4i with $CYP51_{Cke.}$

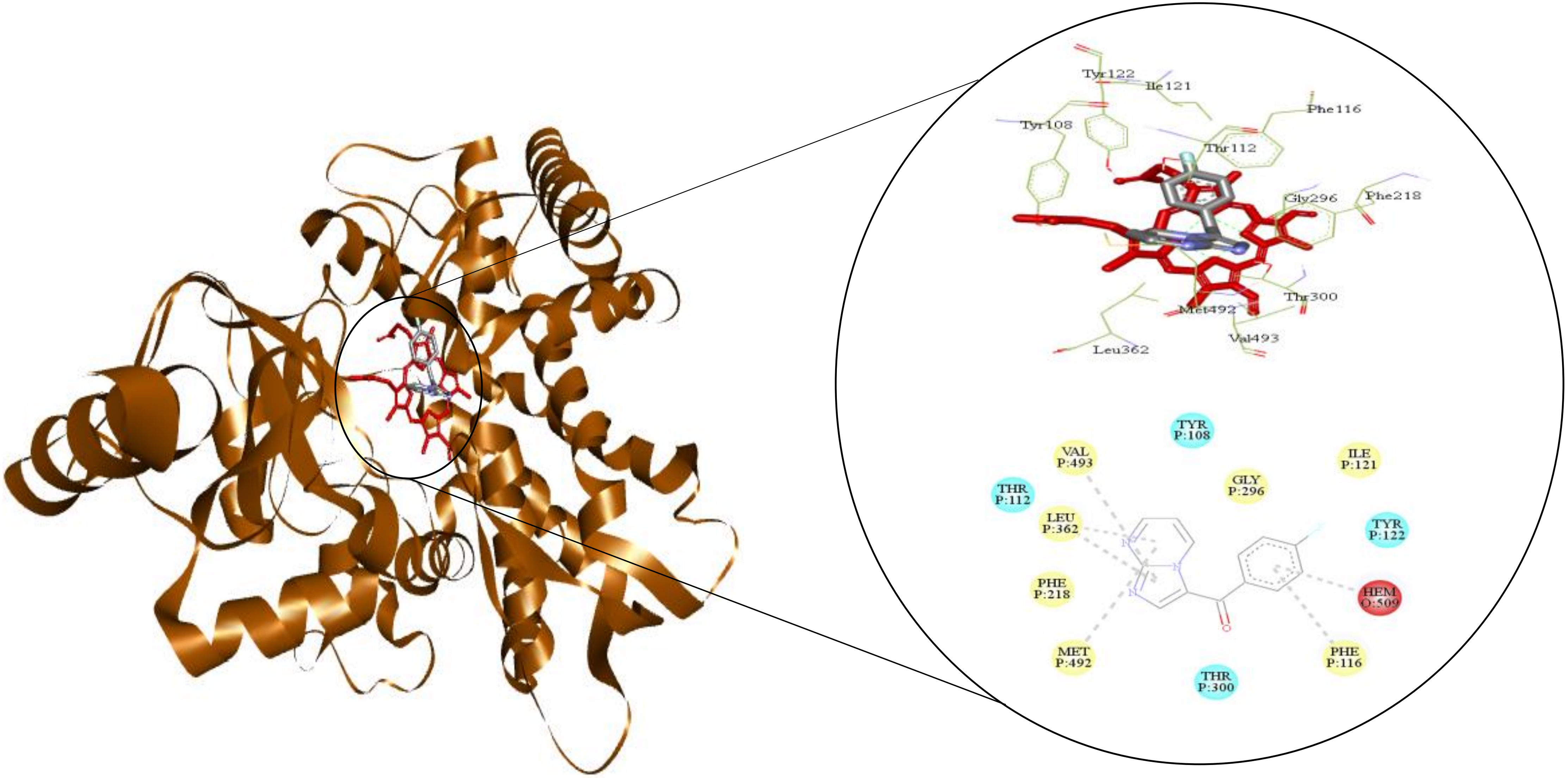

Figure S96: Schematic representation of the interactions of 4j with $CYP51_{Cke.}$

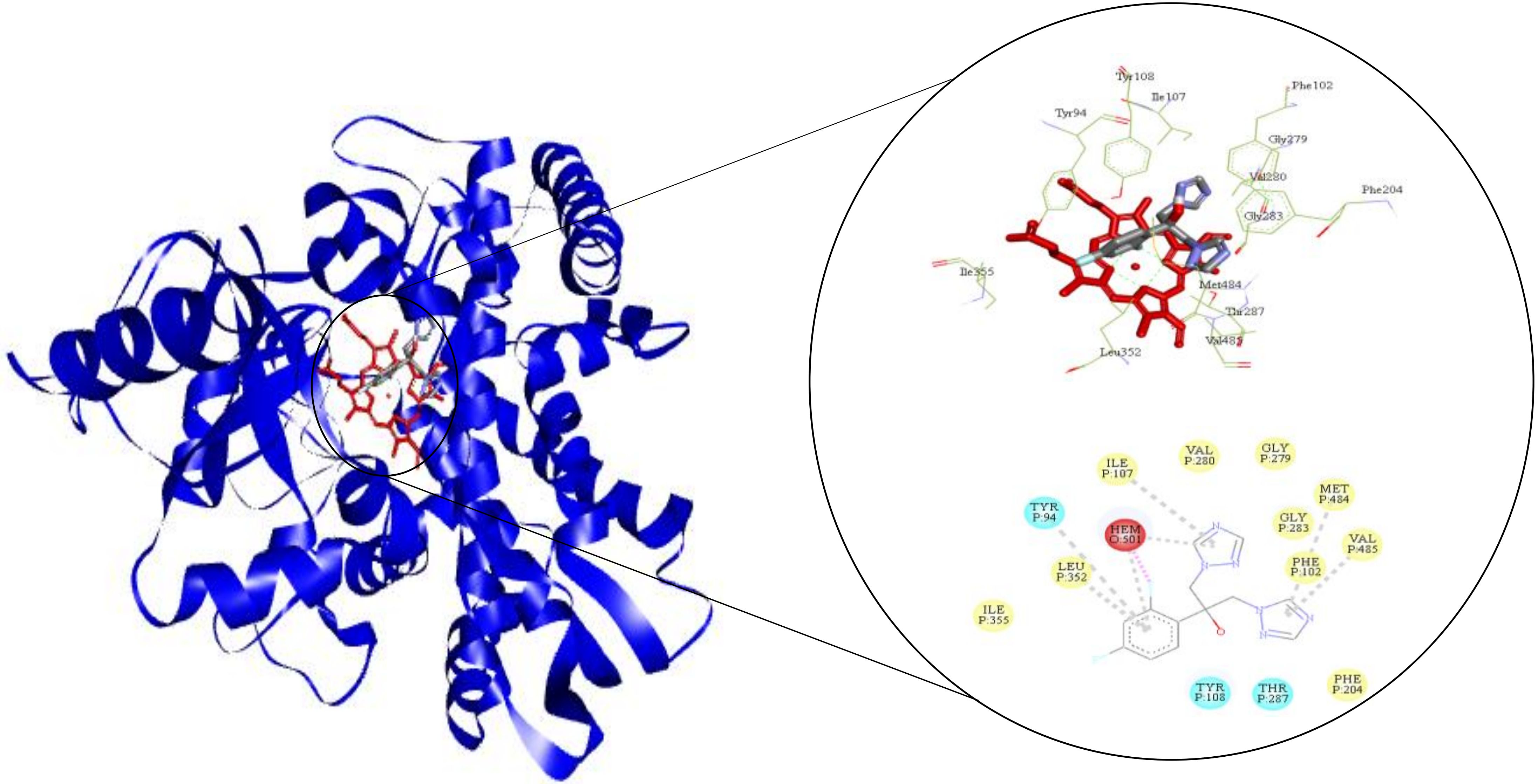

Figure S97: Schematic representation of the interactions of fluconazole with $CYP51_{Ct.}$

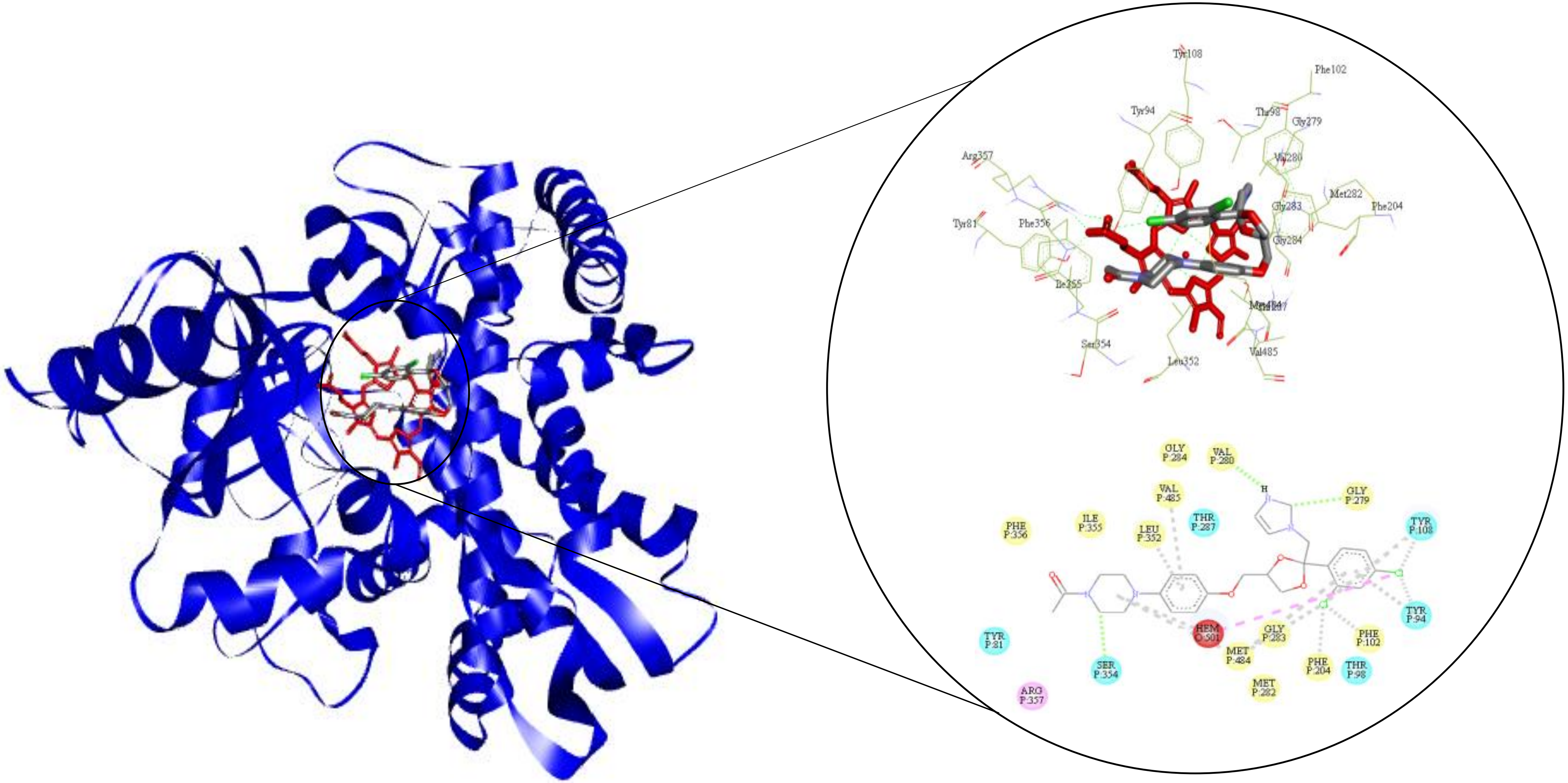

Figure S98: Schematic representation of the interactions of ketoconazole with $CYP51_{Ct.}$

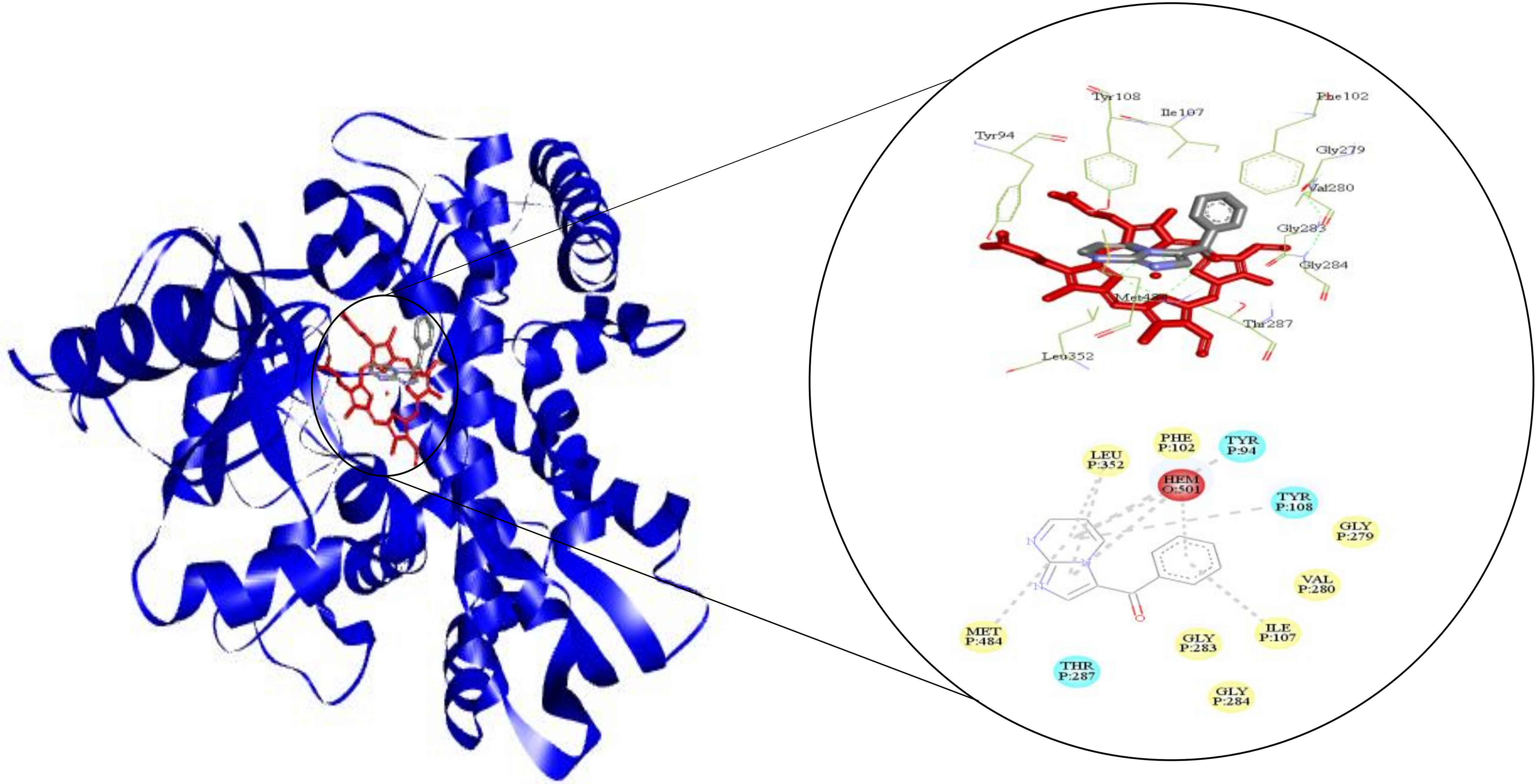

Figure S99: Schematic representation of the interactions of 4a with $CYP51_{Ct.}$

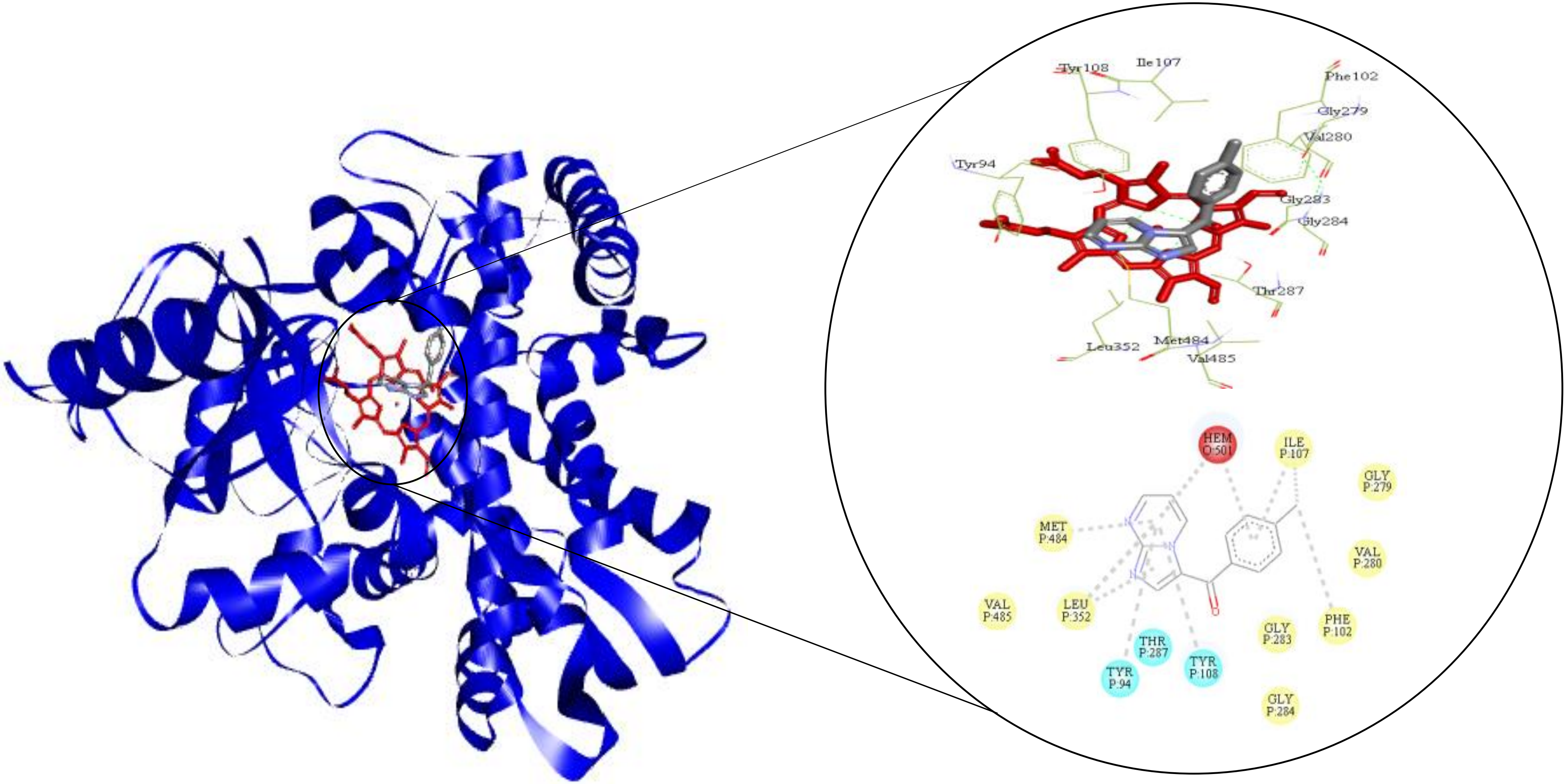

Figure S100: Schematic representation of the interactions of 4d with $CYP51_{Ct.}$

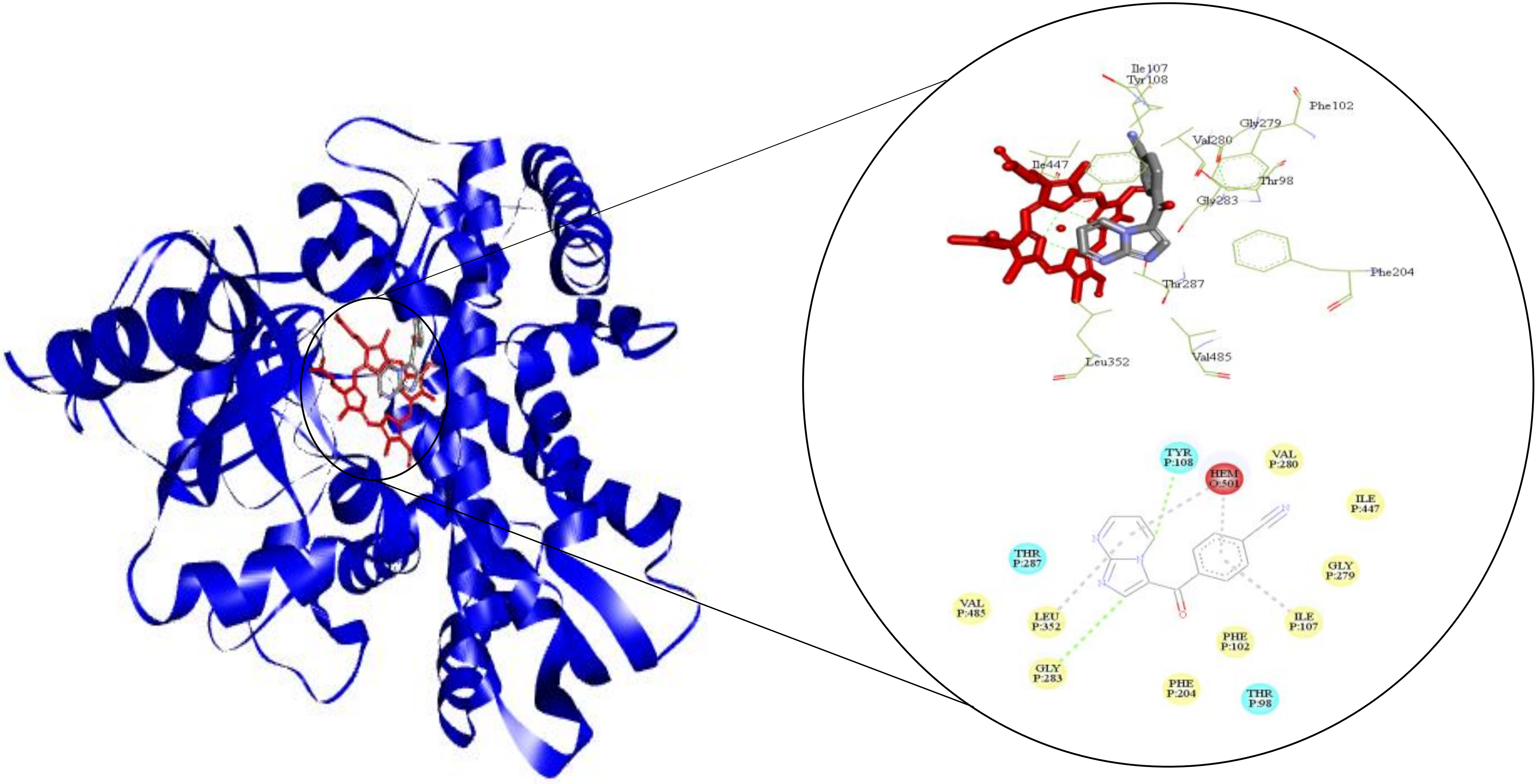

Figure S101: Schematic representation of the interactions of 4f with $CYP51_{Ct.}$

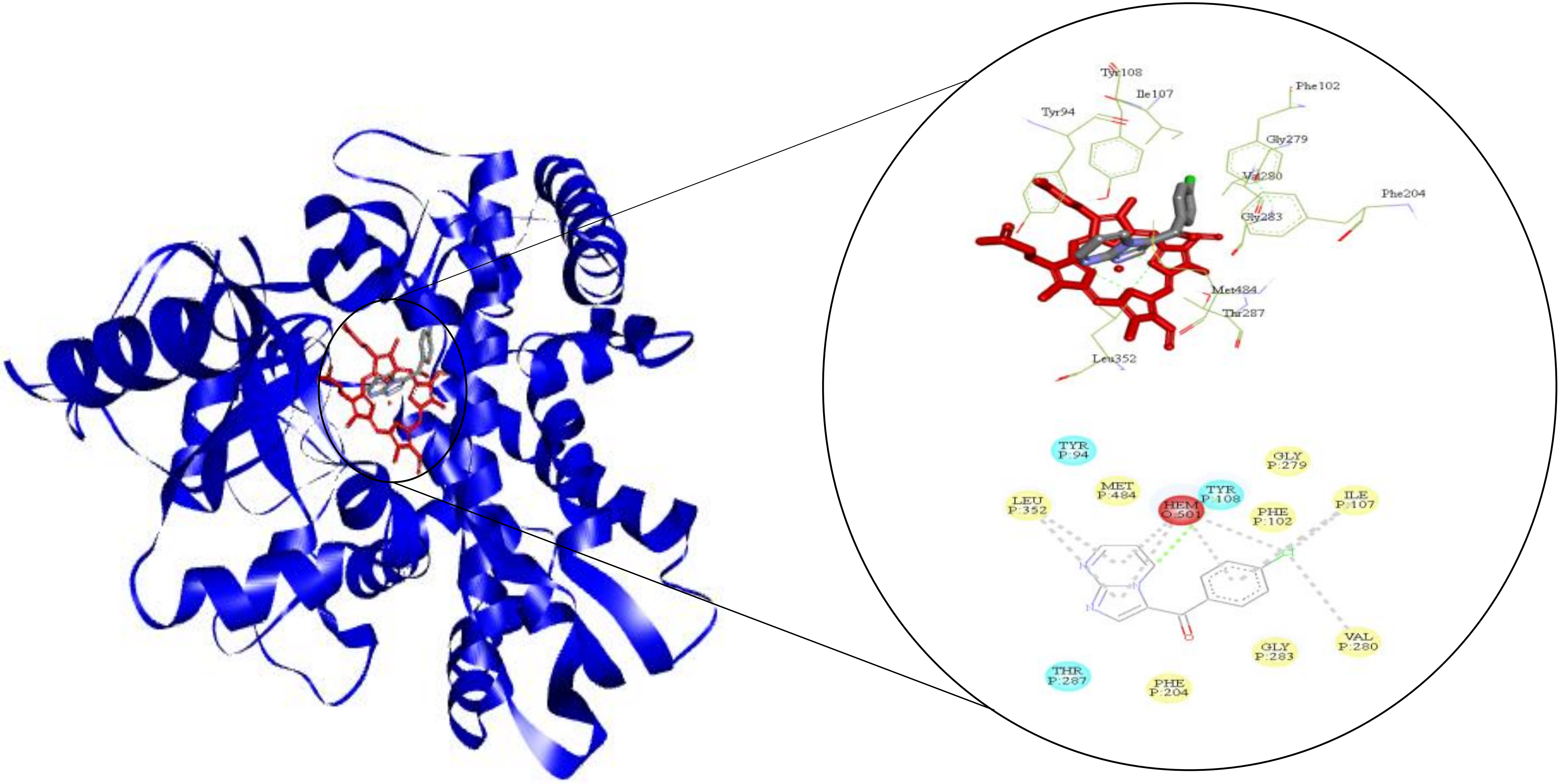

Figure S102: Schematic representation of the interactions of 4i with $CYP51_{Ct.}$

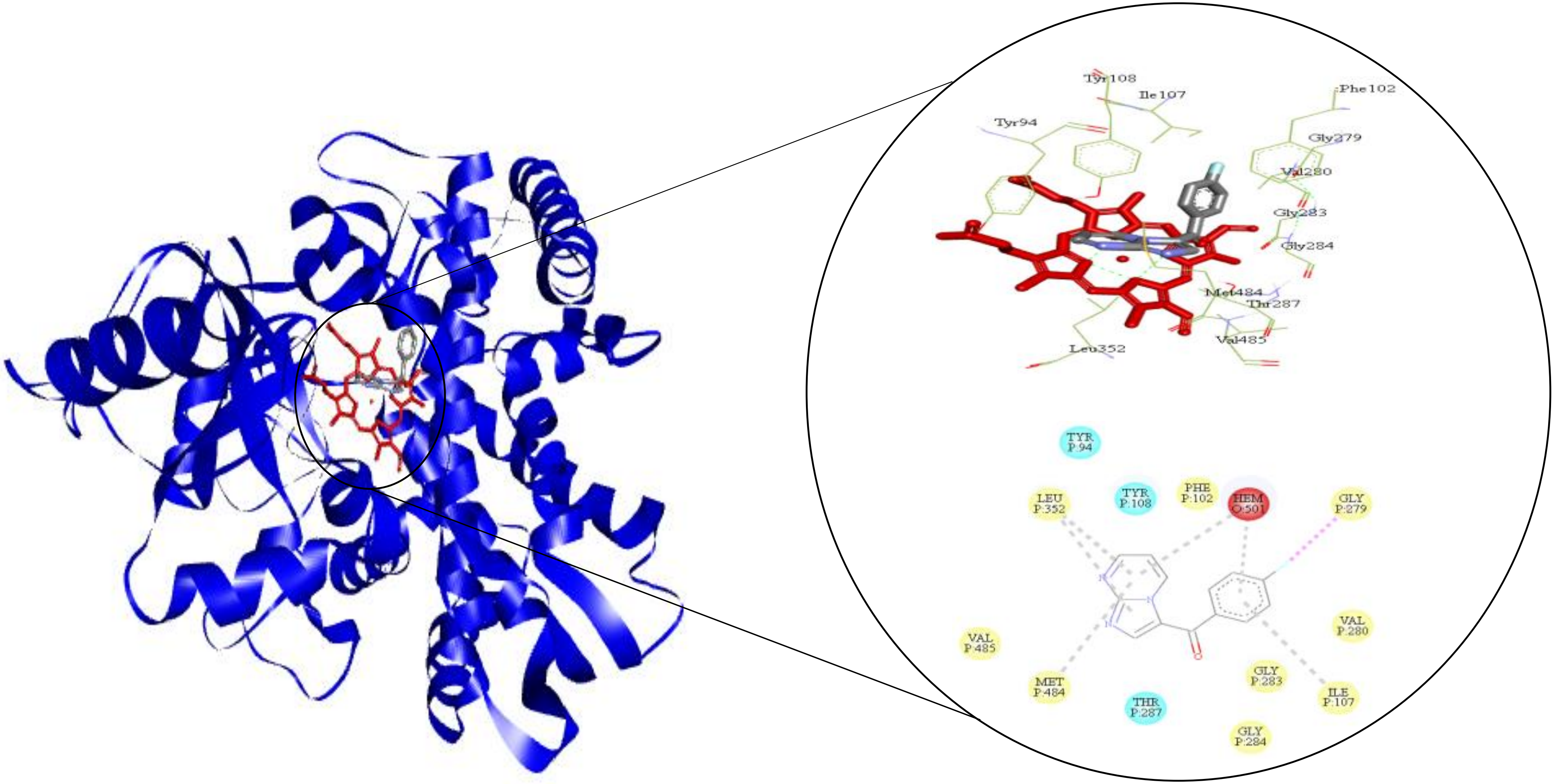

Figure S103: Schematic representation of the interactions of 4j with $CYP51_{Ct.}$
